# Synthesis and Reactivity of N‐Heterocyclic Carbene‐Phosphinidene Manganese Complexes

**DOI:** 10.1002/chem.202500997

**Published:** 2025-04-03

**Authors:** Dustin Bockhardt, Thomas Bannenberg, Matthias Tamm

**Affiliations:** ^1^ Institut für Anorganische und Analytische Chemie Technische Universität Braunschweig Hagenring 30 38106 Braunschweig Germany

**Keywords:** manganese, N‐heterocyclic carbenes, phosphinidenes, selenium, tellurium

## Abstract

The reaction of manganese pentacarbonyl bromide with the N‐heterocyclic carbene‐phosphinidene adduct (IDipp)PSiMe_3_ afforded the tetracarbonyl complex [(IDipp)PMn(CO)_4_] (**1**) with the release of Me_3_SiBr and CO (IDipp = 1,3‐bis(2,6‐diisopropylphenyl)‐imidazolin‐2‐ylidene). X‐ray crystallographic analysis revealed a trigonal‐bipyramidal coordination geometry with a short Mn─P bond, indicative of double bond character. CO substitution with phosphines, NHCs, and isocyanides yielded complexes [(IDipp)PMnL(CO)_3_] (**2**–**5**) in which the ligands (L) occupy axial positions. Kinetic studies and DFT calculations support an associative substitution mechanism, analogous to the nitrosyl complex [Mn(NO)(CO)_4_], demonstrating the ability of (NHC)P and NO ligands to modulate their electronic properties as either one‐ or three‐electron donors. Complex **1** also underwent phosphinidene transfer and chalcogenation reactions along the manganese‐phosphorus bond, resulting in metalladiphosphiranes (**6**, **7**) and metallaphospha‐chalcogeniranes (**8**, **9**) with three‐membered MnPP and MnPE (E = Se, Te) rings.

## Graphical abstract

The N‐heterocyclic carbene‐phosphinidene (NHCP) manganese complex [(NHC)PMn(CO)_4_] undergoes CO substitution reactions via an associative mechanism akin to the nitrosyl complex [Mn(NO)(CO)_4_], with the (NHC)P ligand, switching between three‐electron and one‐electron donor modes. Phosphinidene transfer and chalcogenation reactions along the Mn–P double bond afford metalladiphosphiranes and metallaphosphachalcogeniranes with three‐membered MnPE rings.

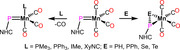

## 1 Introduction

**Figure 1:**
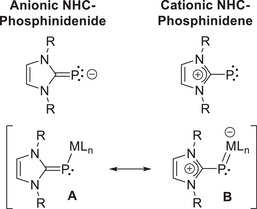
Presentation of (NHC)P ligands and resonance structures of (NHC)P‐metal complexes.

N‐heterocyclic carbene‐phosphorus (NHCP) compounds have emerged as an important class of main‐group species, where NHC ligands stabilize the phosphorus atom in diverse oxidation states and coordination environments.^[^ ^1^ ^]^ Notably, low‐valent phosphorus species, such as NHC‐phosphinidene adducts (NHC)PR, have garnered attention as strongly and inversely polarized phosphaalkenes,^[^ ^2^ ^]^ a property that can be attributed to the ability of the imidazole ring to effectively stabilize a positive charge.^[^ ^3^ ^]^ Expanding on this concept, transition metal complexes of the type [(NHC)PML_n_] feature phosphorus(I) ligands, which can be formally described either as anionic NHC‐phosphinidenides or cationic NHC‐phosphinidenes. These complexes can develop strong and covalent metal‐phosphorus π‐interactions, as illustrated by the resonance between the canonical forms **A** and **B** (Figure 1).^[^ ^4^, ^5^, ^6^, ^7^, ^8^ ^]^

**Figure 2:**
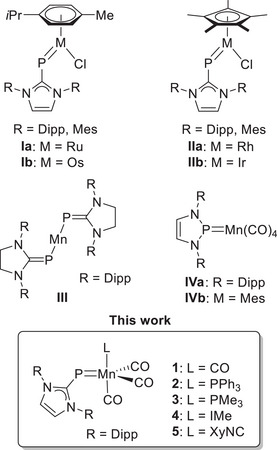
Selected transition metal (NHC)P and NHP complexes.

Representative (NHC)P‐metal complexes are shown in Figure 2. Naturally, the metal centers in the half‐sandwich complexes **I** and **II** attain an 18‐electron configuration only when a metal‐phosphorus double bond is considered, in agreement with both experimental and theoretical studies.^[^ ^4^, ^5^ ^]^ Related cationic complexes, in which the chlorido ligands in **I** and **II** are replaced by neutral ligands (CO, PR_3_, NHC),^[^ ^6^ ^]^ further emphasize the analogy to nucleophilic phosphinidene complexes of the type [RP═ML_n_] containing identical complex fragments.^[^ ^9^ ^]^ This similarity in electronic structure and bonding is also reflected in the comparable ^31^P NMR chemical shifts observed for (NHC)P metal complexes, which appear in the deshielded 300–600 ppm range. Catalytic applications of complexes **I** and **II** were also investigated, and the ruthenium complex **Ia** (R = Mes) proved to be a very efficient catalyst for the hydroboration of nitriles, esters, and amides, demonstrating the potential of (NHC)P ligands to serve as novel ancillary phosphorus ligands in homogeneous catalysis.^[^ ^7^ ^]^ Moreover, an extension to complexes with terminal (NHC)As ligands was also achieved.^[^ ^8^ ^]^

Numerous main‐group metal and metalloid complexes with terminal (NHC)P ligands have also been described in recent years, including those with K,^[^ ^10^ ^]^ Rb,^[^ ^10^ ^]^ Al,^[^ ^11^ ^]^ Ga,^[^ ^12^ ^]^ Si,^[^ ^4^, ^13^, ^14^, ^15^, ^16^ ^]^ Ge,^[^ ^14^, ^17^, ^18^, ^19^, ^20^ ^]^ Sn,^[^ ^13^, ^17^, ^18^, ^19^ ^]^ Pb,^[^ ^17^ ^]^ As,^[^ ^21^, ^22^, ^23^, ^24^ ^]^ Sb,^[^ ^21^ ^]^ Bi,^[^ ^21^ ^]^ and Hg.^[^ ^25^ ^]^ In contrast, 3d transition metal complexes with terminal (NHC)P ligands are virtually unknown, with the only reported example being the unusual homoleptic, open‐shell manganese complex **III** (Figure 2), This complex was unexpectedly formed from the reaction of the manganese diamide [Mn{N(SiMe_3_)_2_}_2_] with the potassium salt [(SIDipp)PK].^[^ ^26^ ^]^ **III** is a high‐spin Mn(II) complex that is stabilized through intramolecular arene‐metal interactions. Other structurally characterized 3d metal complexes contain bridging (NHC)P ligands, which were obtained via template synthesis. These are limited to a divanadium^[^ ^27^ ^]^ and two dinickel species.^[^ ^28^, ^29^ ^]^

Regardless of the charge assignment in the aforementioned complexes, the mesomeric forms **A** and **B** illustrate the ability of (NHC)P ligands to modulate their donor properties, acting as either one‐ or three‐electron donors according to the neutral electron counting convention – a characteristic they share, in principle, with the nitrosyl (NO) ligand.^[^ ^30^, ^31^, ^32^, ^33^ ^]^ Consequently, replacing NO in known nitrosyl complexes of the type [M(NO)(CO)_n_] (M = V, n = 5; M = Mn, n = 4; M = Co, n = 3) with (NHC)P should yield complexes with similar structural features and reactivity. To validate this concept and given the well‐documented reactivity of the manganese‐nitrosyl complex [Mn(NO)(CO)_4_],^[^ ^34^ ^]^ we aimed to synthesize the analogous [(NHC)PMn(CO)_4_] complex **I** (Figure 2) and present herein its characterization, as well as its reactivity toward CO substitution and along the manganese‐phosphorus bond.

In this context, it is important to note that phosphorus(III) ligands in (R_2_P)ML_n_ complexes can similarly be classified as either phosphenium or phosphido complexes based on their electronic structure: a pyramidal phosphorus center corresponds to a one‐electron donor, whereas a planar phosphorus atom indicates a three‐electron donor bonding situation.^[^ ^35^, ^36^ ^]^ However, while a few [(R_2_P)Mn(CO)_4_] exist, they predominantly form dinuclear species with phosphido bridges.^[^ ^37^ ^]^ An exception is the class of N‐heterocyclic phosphenium (NHP) complexes of the type [(NHP)M(CO)_n_], including the manganese species [(NHP)Mn(CO)_4_] (**VI**, Figure 2).^[^ ^38^, ^39^, ^40^, ^41^ ^]^ These complexes are typically synthesized from the corresponding chlorides (NHP)Cl via reaction with carbonyl metallates [M(CO)_n+1_]^−^ (M = V, Mn, Co).^[^ ^38^, ^39^, ^40^, ^41^, ^42^, ^43^ ^]^

## 2 Results and Discussion

### 2.1 Synthesis and Characterization of Manganese (NHC)P Carbonyls

**Scheme 1:**
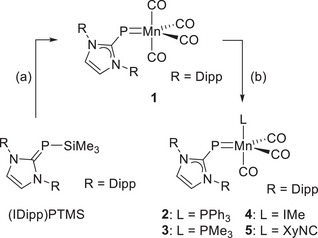
Synthesis of NHC‐phosphinidene complexes **1**–**5**; (a) Mn(CO)_5_Br, toluene, rt, Δ*p*, –CO, –Me_3_SiBr; (b) L, toluene, rt, –CO.

Addition of (IDipp)PSiMe_3_ to a stirred suspension of manganese pentacarbonyl bromide, [Mn(CO)_5_Br], in toluene resulted in a slow color change from orange to dark brown (Scheme 1). Stirring overnight at room temperature under slightly reduced pressure to facilitate the elimination of one equivalent of carbon monoxide resulted in a dark green solution. After removal of solvent and Me_3_SiBr, crystallization from a THF solution layered with *n*‐hexane and subsequent washing with *n*‐pentane, the NHC‐phosphinidene manganese tetracarbonyl complex **1** was isolated as a green, air‐ and moisture‐sensitive solid in good yield (69%). **1** can be stored at −40 °C for several months but readily decomposes in coordinating or chlorinated solvents within a few days at room temperature.

**Figure 3:**
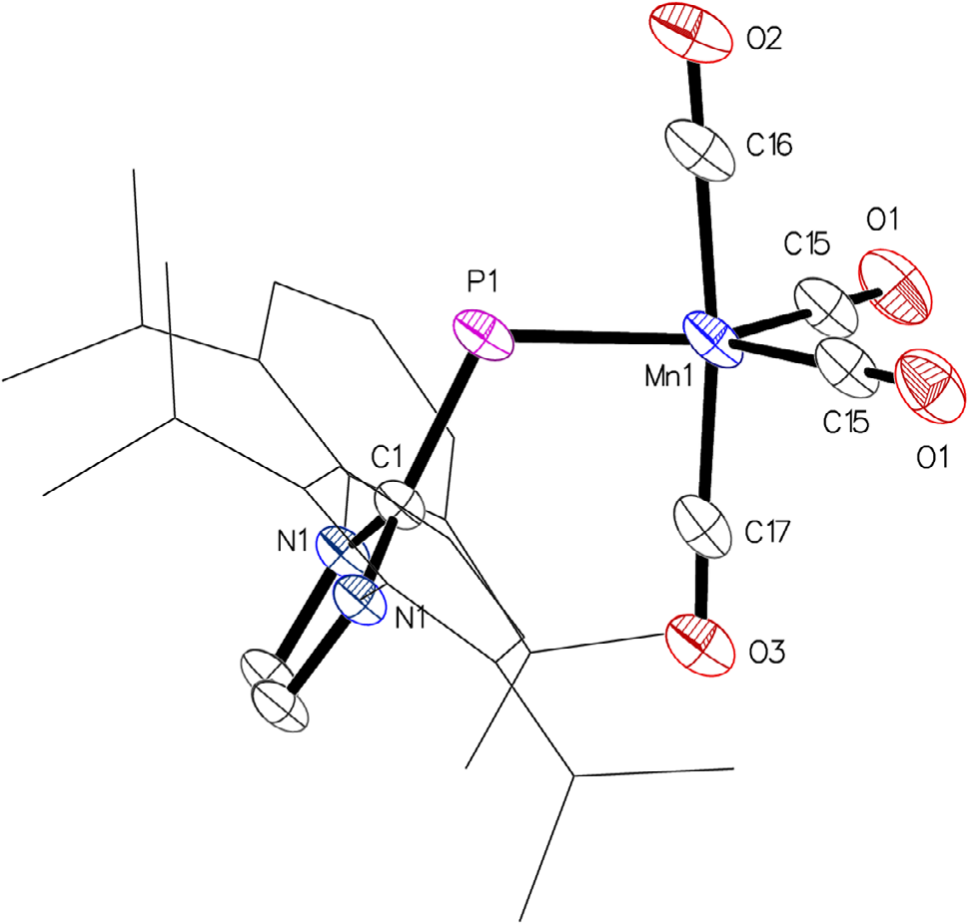
Molecular structure of complex **1** in **1**•*n*‐pentane with thermal displacement parameters drawn at the 50% probability level. Symmetrically equivalent atoms were generated using the following transformation: (x, ½‐y, z). Hydrogen atoms and the solvent molecule were omitted for clarity. Dipp groups are displayed as wireframes. Selected interatomic distances and angles are listed in Table 1.

Storage of a solution of **1** in a toluene/*n*‐pentane mixture at −40 °C resulted in the formation of brown single crystals of **1**•pentane suitable for X‐ray diffraction (XRD) analysis, the resulting molecular structure is presented in Figure 3. Complex **1** crystallized in the monoclinic space group *P2*_1_/*m*, with half a molecule in the asymmetric unit. The manganese atom is pentacoordinate, revealing the loss of one CO ligand, and its geometry is best described as distorted trigonal‐bipyramidal, with the phosphorus atom and the carbon atoms C15/C15’ occupying equatorial positions. The distortion arises primarily from the larger P─Mn─C15/C15’ angle of 130.84(10)° compared to the significantly smaller C15─Mn─C15’ angle of 98.3(2)°, while the C16─Mn─C17 angle of 173.0(2)° involving the axial carbonyl ligands is close to linearity. The Mn─P─C1 angle is 114.06(12)°, consistent with the typical bent coordination mode of phosphinidene ligands. Due to crystallographic *C*_s_ symmetry, the imidazole plane is perfectly perpendicular to the Mn─P─C1 plane, which leads to polarization and elongation of the P─C1 bond, increasing from 1.752(1) Å in (IDipp)PH to 1.822(3) Å in **1**.^[^ ^4^, ^24^ ^]^ Accordingly, a short Mn─P bond length of 2.1904(11) Å is observed, which is significantly shorter than that of the homoleptic paramagnetic (NHC)P complex **III** (2.4206(6) Å)^[^ ^26^ ^]^ and slightly longer than that of the phosphinidene complex [Cp(CO)_2_Mn═PN(Mes*)B(*t*Bu)Cl] (2.1222(8) Å).^[^ ^44^ ^]^ Even shorter Mn─P bond lengths were found for NHC‐phosphenium complexes, such as 2.0625(11) Å in **IVa** ^[^ ^39^ ^]^ and 2.0631(3) Å in **IVb**,^[^ ^40^ ^]^ where a manganese‐phosphorus double bond has been assigned. Other phosphenium complexes of the type [RR'P═Mn(CO)_4_] also exhibit slightly shorter Mn─P bond lengths, consistent with their classification as P(III) species rather than P(I) species like **1**.^[^ ^45^, ^46^, ^47^, ^48^ ^]^

Complex **1** gives rise to one signal in the ^31^P{^1^H} NMR spectrum, characterized by a strongly deshielded chemical shift of 627.5 ppm (in CD_2_Cl_2_). This value aligns with previously reported NHC‐phosphinidene complexes of Ru, Os, Rh, and Ir such as **I** and **II** (353.3–596.9 ppm).^[^ ^4^, ^5^, ^6^, ^7^ ^]^ Notably, this chemical shift is similar to those observed for middle to late 3d transition metal complexes containing neutral amino‐ or arylphosphinidene moieties, which typically show downfield shifts greater than 850 ppm. Examples include complexes [Cp(L)Co═PMes*] (L = PPh_3_, *δ* ^31^P = 867 ppm; L = CO, *δ* ^31^P = 1047 ppm),^[^ ^49^ ^]^ [Cp*(CO)_2_Fe═PN*i*Pr_2_][AlCl_4_] (*δ* ^31^P = 965 ppm),^[^ ^50^ ^]^ [(PPh_3_)(CO)_3_Co═PN*i*Pr_2_][AlCl_4_] (*δ* ^31^P = 861.2 ppm),^[^ ^51^ ^]^ and [(dtbpe)Ni═P(dmp)] (dtbpe = 1,2‐(di‐*tert*‐butylphosphino)ethylene, dmp = 2,6‐dimesitylphenyl, *δ* ^31^P = 970 ppm).^[^ ^52^ ^]^

The pentacoordinate manganese atom in **1** attains an 18‐valence‐electron configuration only if the Mn─P bond is regarded as a double bond, with the (NHC)P ligand acting as a three‐electron donor according to the neutral counting convention. Therefore, the reactivity of **1** in carbonyl substitution reactions may resemble that of the nitrosyl‐tetracarbonyl complex [Mn(NO)(CO)_4_], which undergoes substitution via an associative mechanism to form complexes of the type [(ON)MnL(CO)_3_].^[^ ^34^ ^]^ Indeed, the addition of phosphines (PR_3_, R = Ph, Me), the N‐heterocyclic carbene 1,3,4,5‐tetramethylimidazolin‐2‐ylidene (IMe), or 2,6‐dimethylphenyl isocyanide (XyNC) to a solution of **1** in toluene caused an immediate darkening of the solution, with a slight color shift to a distinct green (L = PR_3_) or yellow (L = IMe, XyNC). After removal of the solvent and washing with *n*‐pentane, the complexes **2**–**5** were obtained as pale dark green to brown solids (Scheme 1).

**Figure 4:**
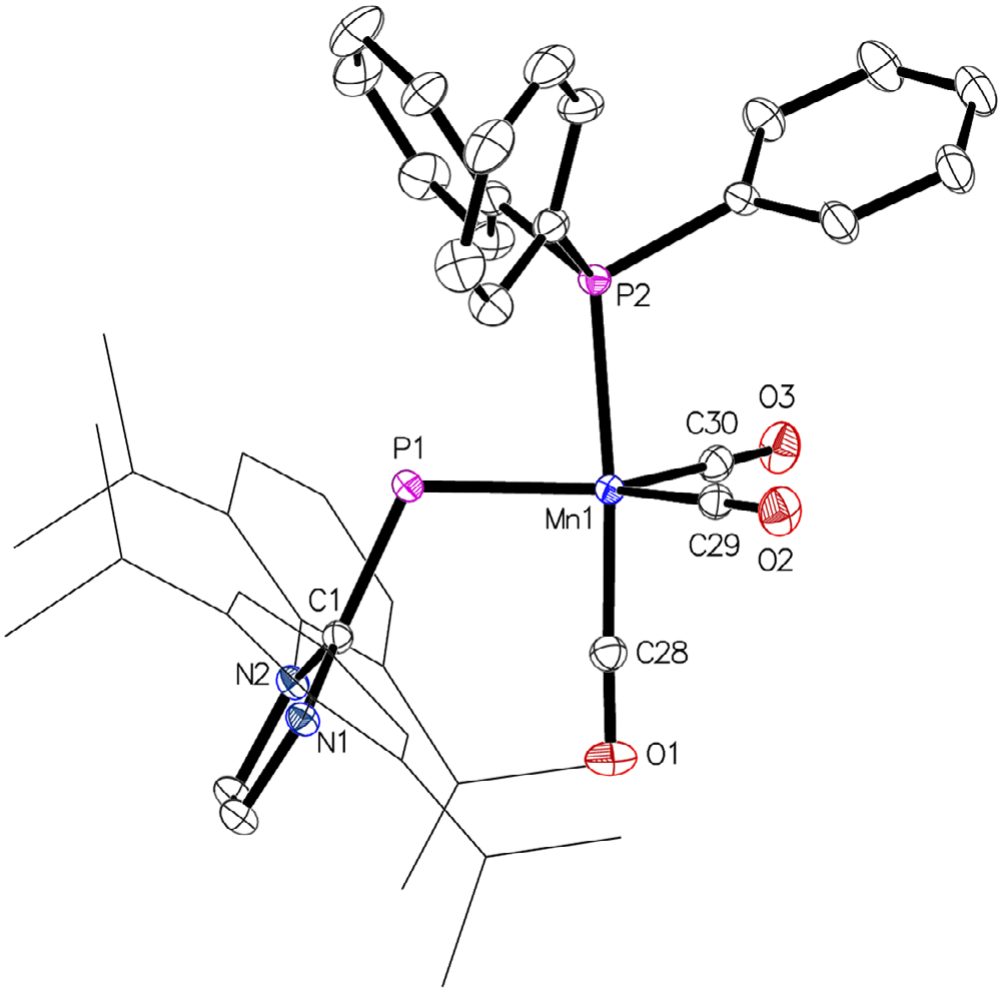
Molecular structure of complex 2 with thermal displacement parameters drawn at the 50% probability level. Hydrogen atoms were omitted for clarity. Dipp groups are displayed as wireframes. Selected interatomic distances and angles are listed in Table 1.

Crystals suitable for XRD analysis were obtained by vapor diffusion of *n*‐hexane into C_6_D_6_ solutions of **2** (L = PPh_3_), **3** (L = PMe_3_), and **4** (L = IMe), or by layering a benzene solution of **5** (L = XyNC) with *n*‐hexane at room temperature. The molecular structure of **2** is presented in Figure 4, while the structures of **3**–**5** are provided in the Supporting Information (Figures S120–S122). Following the substitution of a CO ligand, the manganese centers in all complexes once again adopt distorted trigonal‐bipyramidal coordination geometries, with the newly introduced ligands each occupying an axial position. Overall, the structural characteristics of **2**–**5** are analogous to those of **1**, with uniformly short Mn–P bond lengths that exhibit slight variation: 2.1805(3) Å in **2**, 2.1939(5) Å in **3**, 2.2136(8) Å in **4**, and 2.1679(9)/2.1673(9) Å in the two crystallographically independent molecules of **5** (Table 1).

**Table 1:** Comparison of selected spectroscopic, structural and computational data for the NHC‐phosphinidene Mn carbonyls [{(IDipp)P}(L)Mn(CO)_3_)] **1**–**5**.

		Mn─P [Å]		Mn─L [Å]		P─C1 [Å]			
Complex (L)	*δ* ^31^P^[^ ^a^ ^]^ [ppm] (*J*_P,P_ [Hz])	Exp.	Calc. (WBI)	Exp.	Calc. (WBI)	Exp.	Calc. (WBI)	Mn─P─C1 [°]	P─Mn─L [°]
**1** (CO)	627.5	2.1901 (11)	2.220 (1.36)	1.848 (4)	1.855 (1.07)	1.822 (3)	1.830 (1.03)	114.05 (12)	84.16 (15)
**2** (PPh_3_)	570.6 (67), 69.2 (65)	2.1805 (3)	2.212 (1.37)	2.2873 (2)	2.348 (0.72)	1.8123 (8)	1.810 (1.07)	113.47 (3)	84.777 (8)
**3** (PMe_3_)	531.9 (60), 17.6 (57)	2.1939 (5)	2.221 (1.38)	2.2965 (5)	2.326 (0.75)	1.8169 (17)	1.813 (1.07)	113.83 (5)	83.932 (17)
**4** (IMe)	461.9	2.2136 (8)	2.239 (1.32)	2.064 (3)	2.046 (0.71)	1.815 (3)	1.814 (1.08)	112.81 (9)	81.75 (9)
**5** (XyNC)^[^ ^b^ ^]^	564.3	2.1679 (9)/ 2.1673 (9)	2.228 (1.32)	1.861 (3)/ 1.874 (3)	1.887 (0.97)	1.743 (2)/ 1.740 (2)	1.824 (1.04)	112.93 (9)/ 112.75 (9)	79.50 (8)/ 79.56 (8)

^[a]^ in CD_2_Cl_2_;

^[b]^ Two crystallographically independent molecules in the asymmetric unit.

The ^31^P{^1^H} spectra of the products revealed the disappearance of the characteristic low‐field signal of complex **1** (627.5 ppm) and the appearance of new, upfield‐shifted signals at 570.6 ppm for **2** (d, ^2^ *J*_P,P_ = 67 Hz), 531.9 ppm for **3** (d, ^2^ *J*_P,P_ = 60 Hz), 461.9 ppm for **4** and 564.2 ppm for **5**, which were assigned to the (NHC)P moiety (Table 1). For complexes **2** and **3**, the second phosphorus atom gives rise to a doublet at 69.2 ppm (^2^ *J*_P,P_ = 65 Hz) and 17.6 ppm (^2^ *J*_P,P_ = 57 Hz), respectively, due to coupling with the phosphorus atom of the phosphinidene moiety. The replacement of a carbonyl ligand with a weaker π‐accepting ligand exerts a pronounced shielding effect on the (NHC)P phosphorus atom, which can be rationalized by a polarization of the Mn–P π‐bond, increasing the electron density on the P atom. Similar to complex **1**, complexes **3**–**5** exhibit hindered rotation around the P1─C1 bond in solution at room temperature, as evidenced by significant broadening of the ^1^H NMR signals of the isopropyl groups. In contrast, complex **2**, which features the sterically demanding PPh_3_ ligand, shows resolved but considerably broadened signals of the isopropyl groups even at room temperature. Cooling the NMR sample to −13 °C (Figure S24) reveals the expected pattern, consisting of two quartets of quartets, which appear as a multiplet, along with four doublets (^3^ *J*_H,H_ = 6.7, 6.8, 6.8, 6.6 Hz). The ^13^C{^1^H} NMR spectra show signals for two distinct carbonyl ligands in complexes **2** (226.9/235.3 ppm), **3** (227.1/233.8 ppm), and **5** (225.5/233.7 ppm), whereas only one carbonyl signal (230.9 ppm) could be observed for complex **4**.

### 2.2 Chemical Bonding Analysis and Mechanistic Studies

To investigate the bonding situation in complexes **1**–**5**, their geometries were optimized using the density functional theory (DFT) method B97‐D at the 6–311G(d,p) level of theory for all atoms except Mn, for which the Stuttgart RSC 1997 ECP basis set ECP10MDF was used, followed by natural bond orbital (NBO) analysis. Full computational details and contour plots of selected NBOs are provided in the Supporting Information. The calculated structural parameters are generally consistent with those determined in the solid state, except for the isocyanide complex **5**, which exhibits an unexpectedly short Mn─P bond in the crystal structure (Table 1). On average, the Mn─P bond lengths are approximately 2.22 Å. The corresponding Wiberg bond indices (WBI) range from 1.32 to 1.38, similar to those calculated for the half‐sandwich complexes **I** and **II** (Figure 4),^[^ ^5^, ^6^ ^]^ supporting the assignment of a polarized, yet covalent Mn─P double bond. Accordingly, an NBO associated with a manganese‐phosphorus π‐interaction is identified for each complex, with an approximate 60/40 distribution of electron density between the phosphorus and manganese atoms, respectively.

**Figure 5:**
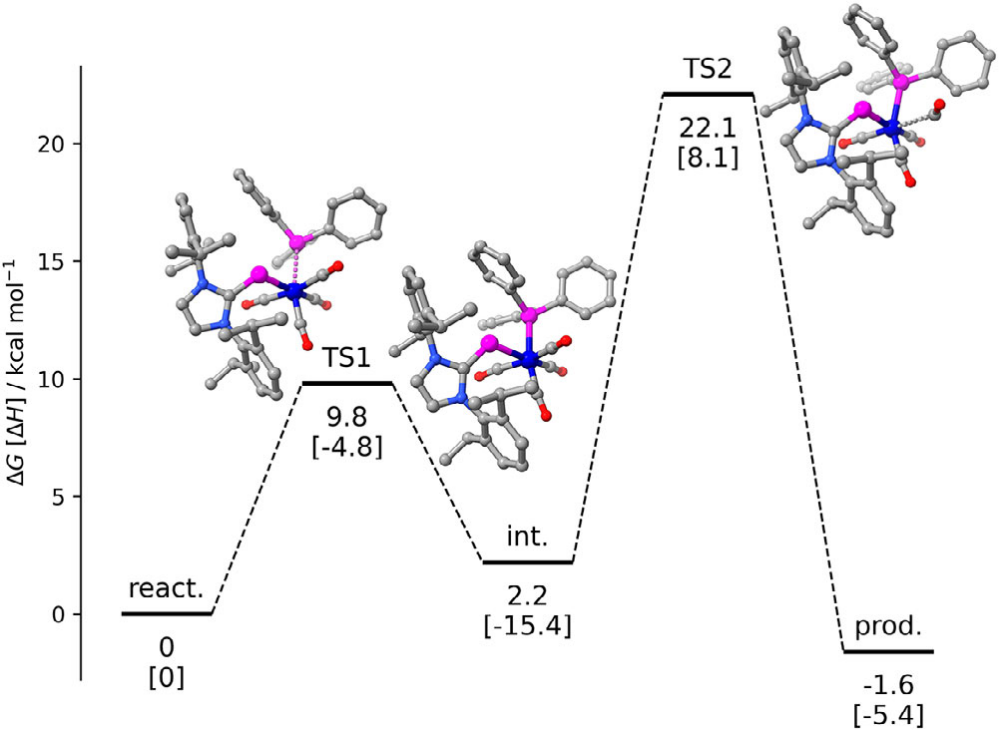
Calculated energy profile for the carbonyl substitution reaction at complex **1** with PPh_3_ (B97‐D // Mn: ECP10MDF/6‐311(d,p)).

To investigate the mechanism of carbonyl substitution, the energy profiles for the reactions of complex **1** with PMe_3_ and PPh_3_ were calculated using intrinsic reaction coordinate (IRC) scans. Since both reactions exhibit comparable energy profiles, only the profile for the reaction with PPh_3_ is shown in Figure 5, while the corresponding profile for PMe_3_ is provided in the Supporting Information (Figure S133). Similar to carbonyl substitution reactions in [Mn(NO)(CO)_4_],^[^ ^34^, ^53^, ^54^ ^]^ the reactions proceed via an association‐dissociation pathway, with the overall product formation being only slightly exergonic, i.e., *ΔG*_298K_(PMe_3_) = −0.4 kcal mol^−1^, *ΔG*_298K_(PPh_3_) = −1.6 kcal mol^−1^. Considering the accuracy of the computational method, these reactions can be considered as approximately thermoneutral, with the primary driving force being the irreversible release of carbon monoxide.

The first transition state (TS1) corresponds to the association of the phosphine ligand, proceeding with relatively low free energy barriers of *ΔG*_298K_(PMe_3_) = 4.1 kcal mol^−1^ and *ΔG*_298K_(PPh_3_) = 9.8 kcal mol^−1^. The subsequent formation of the hexacoordinate intermediate (int.) is slightly exergonic for PMe_3_ (*ΔG*_298K_ = −1.6 kcal mol^−1^) and slightly endergonic for PPh_3_ (*ΔG*_298K_ = 2.2 kcal mol^−1^). Phosphine coordination induces a significant elongation of the manganese‐phosphorus bonds to the (NHC)P ligand, increasing from 2.220 Å in **1** to 2.549 and 2.530 Å in the PMe_3_ and PPh_3_ adducts, respectively. Concurrently, the P–C1 bond lengths decrease from 1.83 Å to approximately 1.78 Å in both adducts. These structural changes suggest a change in the electronic nature of the (NHC)P ligand from a three‐electron to a one‐electron donor, consistent with a shift from mesomeric structure **A** to **B** (see above, Figure 1). Finally, CO dissociation constitutes the rate determining step, proceeding via the second transition state (TS2) with barriers of *ΔG*_298K_(PMe_3_) = 19.9 kcal mol^−1^ and *ΔG*_298K_(PPh_3_) = 22.1 kcal mol^−1^.

To validate the proposed mechanism, the reaction of complex **1** with PPh_3_ in THF was investigated by IR spectroscopy. The experiments were conducted under pseudo‐first‐order conditions, following Basolo's protocol,^[^ ^34^, ^55^ ^]^ with ligand concentrations exceeding a tenfold excess relative to complex **1**. The absorbance (*A*) was monitored over time (*t*), and isosbestic points were observed at 1897 and 1957 cm^−1^, indicating a clean reaction without detectable side products (Table S13). Full experimental details are provided in the Supporting Information. Plots of ln(|*A*_∞_−*A*_t_|) versus *t* yielded good linear fits over the first 2.5 h from which the apparent rate constant *k’* was determined (Table S14).^[^ ^56^ ^]^ To differentiate between first‐ or second‐order kinetic models, the mean apparent rate constants (Table S15) were plotted against the ligand concentration. A linear correlation was observed, and the data were fitted to estimate a second‐order rate constant of *k =* 1.08(5)·10^−4^ L mol^−1^ s^−1^ (Figure S115). Using the Eyring equation,^[^ ^57^ ^]^ the free activation energy was determined to be *Δ* *G* ^‡^ = 22.5 kcal mol^−1^, which aligns perfectly with the calculated barrier for TS2 (*vide supra*). It should be noted that the application of a polarized continuum solvent model (SMD) with THF as a solvent provided a slightly higher barrier of 25.0 kcal mol^−1^ (Figure S126), which is still in very good agreement with the experimental findings.

For comparison, the substitution of one CO ligand with PPh_3_ in [Mn(NO)(CO)_4_] exhibits a second‐order rate constant of *k* = 2.2·10^−3^ L mol^−1^ s^−1^ determined at 50 °C in *p*‐xylene.^[^ ^34^ ^]^ This affords an activation barrier of *Δ* *G* ^‡^ = 22.9 kcal mol^−1^, confirming that the substitution reactions in **1** and [Mn(NO)(CO)_4_] follow a similar reaction mechanism. In contrast, CO substitution with PPh_3_ in [(*η* ^3^‐allyl)Mn(CO)_4_] follows first‐order kinetics at a slower rate (*k*_1_ = 2.80·10^−4^ L mol^−1^ s^−1^ at 45 °C),^[^ ^58^ ^]^ resulting in a barrier of *Δ* *G* ^‡^ = 23.8 kcal mol^−1^. In this system, the rate‐determining step involves CO dissociation and formation of the coordinatively unsaturated intermediate [(*η* ^3^‐allyl)Mn(CO)_3_], which has been characterized spectroscopically only at low temperatures in argon or nitrogen matrices.^[^ ^59^ ^]^

### 2.3 Phosphinidene and Chalcogen Transfer Reactions

**Scheme 2:**
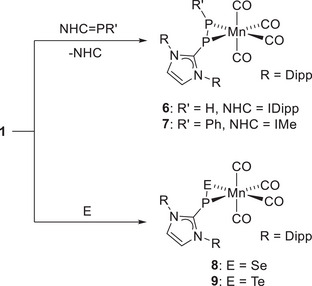
Synthesis of manganadiphosphiranes **6** and **7**, manganaphospha‐selenirane **8**, and manganaphosphatellurirane **9**.

The reactivity of complex **1** with H_2_ was investigated by stirring a toluene solution under an H_2_ atmosphere. We hypothesized that heterolytic cleavage of the H─H bond could occur across the Mn─P double bond, leading to the formation of the hydrido complex [{(IDipp)PH}MnH(CO)_4_]. Similar reactivity had been envisaged for the NHP complexes **IV**, however, the preparation of [(NHPH}MnH(CO)_4_] proceeded stepwise by subsequent hydride and proton transfer^[^ ^38^, ^39^ ^]^ or by photochemical activation.^[^ ^41^ ^]^ After prolonged exposure (48 h) and repeated exchanges of the H_2_ atmosphere (10 times), the dark green color of the solution disappeared, suggesting the conversion of the starting material into a species lacking the Mn─P double bond. Subsequent solvent removal and analysis by ^31^P{^1^H} NMR spectroscopy confirmed this transformation, as the low‐field signal associated with the (NHC)P unit was no longer observed. Instead, multiple new signals appeared in the range of 50 to −250 ppm, indicating the formation of a complex mixture of phosphorus‐containing species. In the ^1^H NMR spectrum, high‐field signals around −6 ppm provided evidence for the formation of manganese‐hydride species. Notably, a doublet of doublets at −0.30 ppm (*J*_H,P_ = 134 Hz, 26 Hz) drew particular attention, as it had been previously observed as an impurity during the synthesis of complex **1**. Corresponding ^31^P NMR signals at −85.6 ppm (^1^ *J*_P,P_ = 279 Hz, *J*_P,H_ = 26 Hz) and −208.9 ppm (^1^ *J*_P,P_ = 279 Hz, *J*_P,H_ = 134 Hz) were detected, consistent with the presence of two distinct phosphorus nuclei coupled to a single hydrogen atom, as expected for metalladiphosphirane complex **6** (Scheme 2).

**Figure 6:**
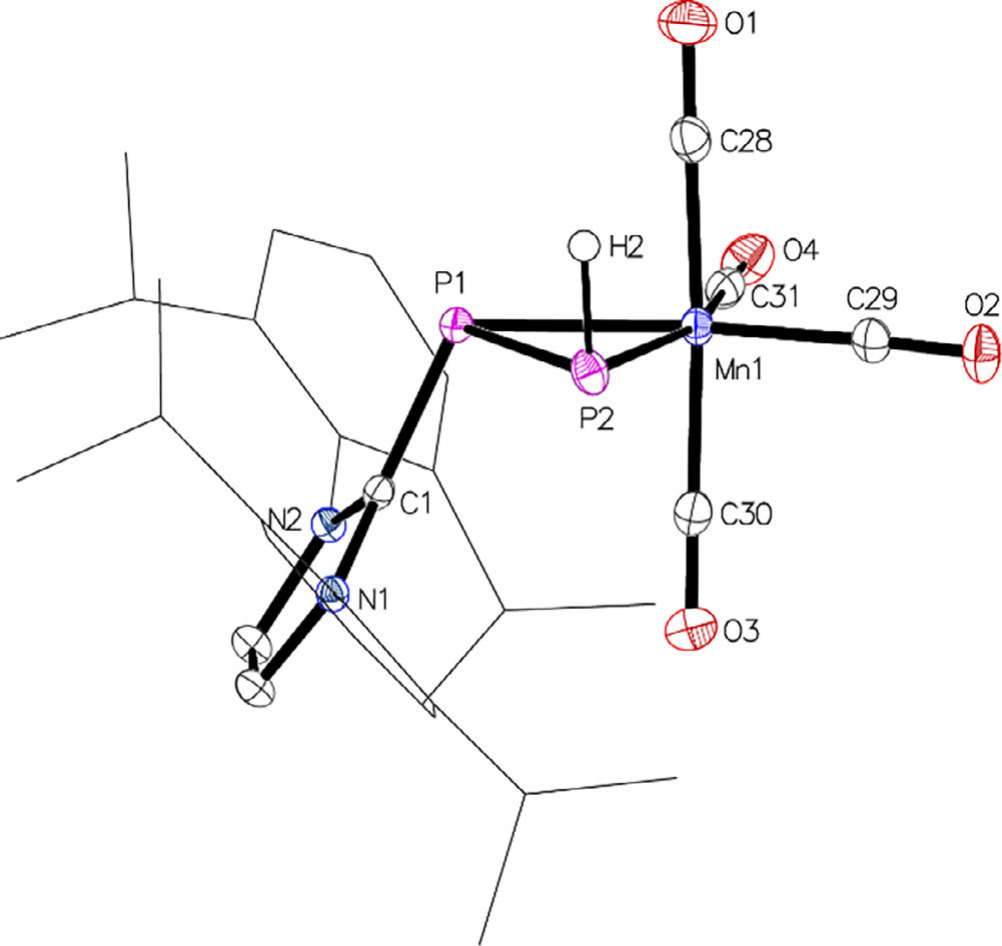
Molecular structure of complex 6 with thermal displacement parameters drawn at the 50% probability level. Hydrogen atoms, disordered isopropyl groups, and the solvent molecule were omitted for clarity. Dipp groups are displayed as wireframes. Selected interatomic distances and angles are listed in Table 2.

Vapor diffusion of *n*‐hexane into the C_6_D_6_ NMR solution yielded pale yellow crystals of [{(IDipp)PPH}Mn(CO)_4_] (**6**, Figure 6) and fewer yellow crystals of [(IDipp)H][μ^2^‐{(IDipp)PPH}Mn_2_(CO)_8_] (Figure S123), both suitable for single‐crystal X‐ray diffraction. The latter consists of an imidazolium cation and a dimanganate counteranion. The formation of these complexes indicates H_2_ activation, accompanied by PH transfer across the Mn─P double bond in **1**. To validate this assumption, complex **1** was reacted with (IDipp)PH in toluene, resulting in an orange solution after 16 h as the green color disappeared. Filtration over alumina to remove the free NHC followed by solvent removal afforded complex **6** as an orange solid in 44 % yield. Extending this protocol to (IMe)PPh,^[^ ^60^, ^61^ ^]^ containing a phenylphosphinidene fragment, gave complex [{(IDipp)PP(Ph)}Mn(CO)_4_] (**7**) as a yellow solid in 63 % yield (Scheme 2, top). In this context, it should be noted that PPh transfer onto organic substrates has been previously described for zinc complexes of a related NHC‐phosphinidene.^[^ ^62^ ^]^

NMR analysis of complex **6** revealed the characteristic ^1^H and ^31^P NMR signals described above, with ^31^P NMR chemical shifts of −85.6 and −208.9 ppm (^1^ *J*_P,P_ = 279 Hz). Likewise, its PPh congener **7** displays two doublets at −84.8 and −98.2 ppm, however, with a significantly larger coupling constant (^1^ *J*_P,P_ = 341 Hz). The molecular structure of **7** was also established by X‐ray diffraction analysis (see Supporting Information, Figure S124). Pertinent structural data of **6** and **7** are summarized in Table 2. In both complexes, the manganese atoms reside in pseudo‐octahedral environments, forming MnP_2_ rings with P1─P2 bond lengths of 2.1612(4) Å (**6**) and 2.1486(2) Å (**7**), within the range found for metal‐bound diphosphenes.^[^ ^63^ ^]^ A similar “diphospha‐manganacyclopropane” structural motif appears in related phosphino‐phosphinidene species [(R_2_PPR’)Mn(CO)_4_],^[^ ^64^, ^65^ ^]^ such as [(*i*Pr_2_PP*^t^* Bu)Mn(CO)_4_], which features a P1─P2 bond lengths of 2.122(2) Å.^[^ ^65^ ^]^ The classification of **6** and **7** as diphosphene complexes with (NHC)P═PR‐*κ* ^2^ *P*,*P* (R = H, Ph) ligands is supported by comparison with the cationic iron complex [{(^Cl^IDipp)PPDipp}Fe(CO)_4_]OTf (^Cl^IDipp = 4,5‐dichloro‐1,3‐di(2,6‐diisopropylphenyl)imidazolin‐2‐ylidene). This compound, synthesized from Fe(CO)_5_ and the cationic diphosphene (^Cl^IDipp)PP(Dipp), has a P1─P2 bond length of 2.1598(10) Å, almost identical with those in **6** and **7**, and exhibits ^31^P NMR chemical shifts of ─8.8 and ─41.7 ppm with ^1^ *J*_P,P_ = 389 Hz.^[^ ^66^ ^]^

**Table 2:** Comparison of selected structural and spectroscopic data for the Mn carbonyls [{(IDipp)P = E}Mn(CO)_4_] **6**–**9**.

Complex	E	Mn─P1 [Å]	Mn─E [Å]	P1─E [Å]	P1─C1 [Å]	Mn─E─P1 [Å]	Mn─P1─E [Å]	*δ* ^31^P{^1^H} [ppm] (^1^ *J*_P,E_ [Hz])	*δ* E{^1^H} [ppm] (^1^ *J*_E,P_ [Hz])
**6**	PH	2.3806 (3)	2.4146 (6)	2.1612 (4)	1.8323 (10)	62.412 (16)	66.022 (16)	−85.6 (279),^[^ ^[a]^, ^[b]^ ^]^	−208.9 (279)^[^ ^[a]^, ^[b]^ ^]^
**7**	PPh	2.3779 (2)	2.4111 (5)	2.1486 (2)	1.8274 (4)	62.555 (14)	64.136 (13)	−84.8 (341)^[^ ^[a]^, ^[b]^ ^]^	−98.2 (341)^[^ ^[a]^, ^[b]^ ^]^
**8**	Se	2.3691 (4)	2.5103 (8)	2.2094 (4)	1.8433 (12)	59.86 (2)	66.39 (2)	−27.4 (360)^[^ ^[c]^, ^[d]^ ^]^	−648 (359)^[^ ^[c]^, ^[d]^ ^]^
**9**	Te	2.3844 (9)	2.6843 (9)	2.4311 (8)	1.841 (3)	55.30 (3)	67.75 (3)	−54.0 (686)^[^ ^[c]^, ^[e]^ ^]^	−1181 (701)^[^ ^[c]^, ^[e]^ ^]^

^[a]^ in C_6_D_6_;

^[b]^ E = ^31^P;

^[c]^ in THF‐d_8_;

^[d]^ E = ^77^Se;

^[e]^ E = ^125^Te.

To further investigate the reactivity of the Mn─P double bond in **1**, the complex was treated with grey selenium or tellurium in THF, affording orange or red solutions after stirring for 3 h or 16 h, respectively. Following solvent removal, the residues were extracted with toluene, filtered, and evaporated to isolate complexes **8** and **9** in moderate to good yields (**8**: E = Se, orange solid, 62%; **9**: E = Te, red solid, 84%; Scheme 2, bottom). NMR spectroscopic characterization confirmed the formation of metallaphosphaselenirane‐ and tellurirane‐species, as evidenced by signals at −27.4 ppm (**8**) and −54.0 ppm (**9**) in the ^31^P{^1^H} NMR spectra, with the expected ^77^Se (^1^ *J*_P,Se_ = 360 Hz) and ^125^Te (^1^ *J*_P,Te_ = 686 Hz) satellite couplings. Accordingly, doublets appear in the respective ^77^Se{^1^H} or ^125^Te{^1^H} NMR spectrum at −648 ppm (^1^ *J*_Se,P_ = 359 Hz) and −1181 ppm (^1^ *J*_Te,P_ = 701 Hz).

**Figure 7:**
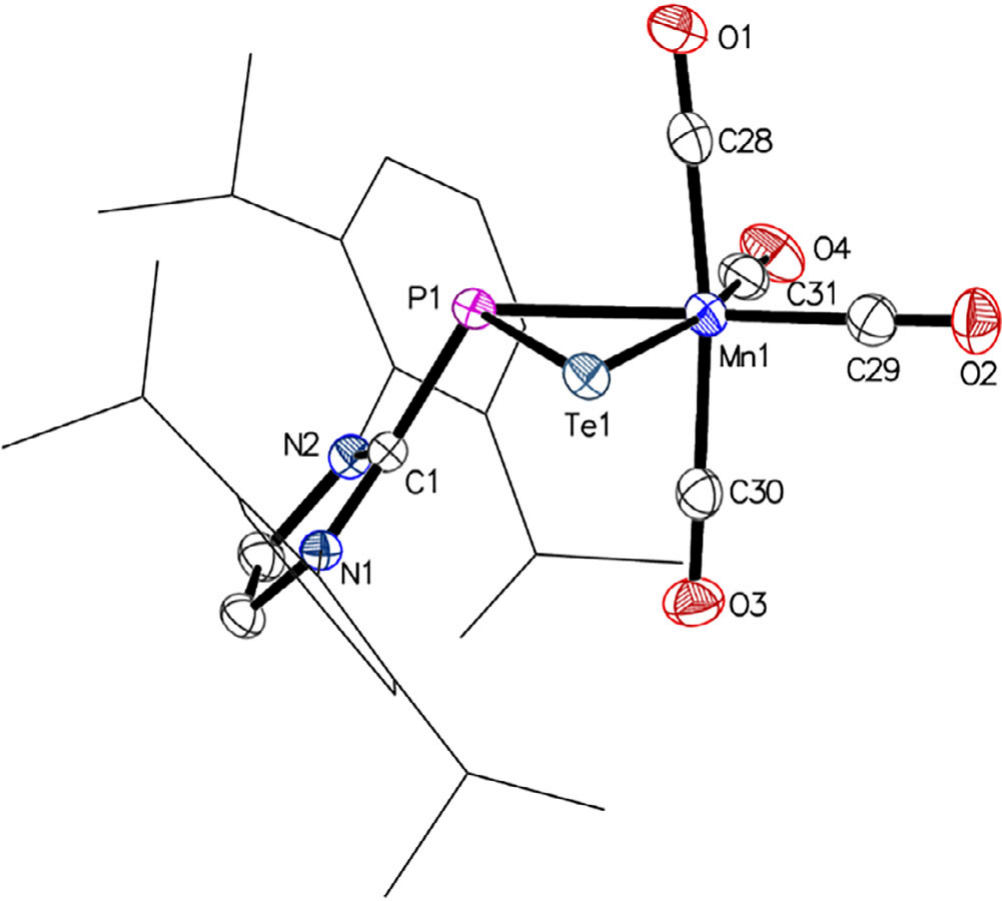
Molecular structure of complex 9 with thermal displacement parameters drawn at the 50% probability level. Hydrogen atoms and the second molecule of the asymmetric unit were omitted for clarity. Dipp groups are displayed as wireframes. Selected interatomic distances and angles Selected interatomic distances and angles are listed in Table 2.

Vapor diffusion of *n*‐hexane into solutions of complexes **8** and **9** in C_6_D_6_ resulted in the formation of orange or red crystals suitable for single‐crystal X‐ray diffraction analysis. Both complexes crystallize in the space group *P*2_1_/*n* with similar lattice parameters. In both cases, one of the two molecules in the asymmetric unit, which resemble enantiomers, shows small (7 % or 11 %) disorder of the [EMn(CO)_4_] moiety (E = Se, Te), corresponding to the other respective enantiomer. The molecular structures of **8** (Figure S113) and **9** (Figure 7) confirm the formation of three‐membered MnPE rings, in which the Mn─P1─E angles of about 65° represent the largest angles (Table 2). Compared to isolobal complexes **6** and **7**, the Mn−E and P1−E bond lengths are longer than the corresponding Mn–P2 and P1–P2 distances, increasing from 2.5103(3) and 2.2094(4) Å in **8** (E = Se) to 2.6843(4) and 2.4311(8) Å in **9** (E = Te) due to the larger van der Waals radii of the chalcogens.^[^ ^67^ ^]^ In contrast, the Mn−P1 distances of 2.3691(4) Å in **8** and 2.3844(9) Å in **9** change only marginally (Table 2).

In the same vein as complexes **6** and **7**, which can be classified as diphosphene complexes with cationic (NHC)P═PR‐*κ* ^2^ *P*,*P* ligands, the ligands in **8** and **9** can be conceived as cationic (NHC)P═E‐*κ* ^2^ *P*,*E* (E = Se, Te) ligands and thus *formally* as NHC‐stabilized heavy nitrosyl analogs.^[^ ^16^, ^18^ ^]^ Similar structural motifs are extremely rare for selenium and unknown for tellurium, with the closest example being [(R_2_PSe)Mn(CO)_4_], which, however, contains a tetracoordinated phosphorus atom and therefore exhibits distinctly different structural and spectroscopic features.^[^ ^68^ ^]^ Other examples include molybdenum and tungsten species with MPSe three‐membered rings, which can be synthesized in a similar fashion to complex **8** through the reaction of the phosphenium complexes [Cp(CO)_2_M═P(Ph){N(SiMe_3_)_2_}] (M = Mo, W) with elemental selenium.^[^ ^69^ ^]^ Similar reactivity is also observed for bulky diphosphenes, RP═PR, which react with elemental selenium and tellurium to form selena‐ and telluradiphosphiranes, respectively.^[^ ^70^, ^71^, ^72^, ^73^, ^74^ ^]^

## 3 Conclusions

The N‐heterocyclic carbene‐phosphinidene adduct (IDipp)PSiMe_3_ has proven to be an efficient (IDipp)P transfer reagent, allowing the synthesis of a series of pentacoordinate manganese carbonyl complexes of the type [(IDipp)PMnL(CO)_3_] (**1**–**5**, L = CO, PPh_3_, PMe_3_, IMe, XyNC). These complexes are rare examples of 3d metal NHC‐phosphinidene species. All complexes exhibit trigonal‐bipyramidal structures and attain an 18‐valence electron configuration, with the (IDipp)P ligand acting as a three‐electron donor according to the neutral electron counting convention. CO substitution in the tetracarbonyl complex [(IDipp)PMn(CO)_4_] (**1**) likely proceeds via an associative mechanism, involving an octahedral intermediate [(IDipp)PMnL(CO)_4_] and subsequent CO release. These findings highlight the ability of (NHC)P ligands to modulate their electronic properties, switching between three‐electron and one‐electron donor modes and adjusting the metal‐phosphorus π‐interaction.

The presence of a covalent yet polarized metal‐phosphorus double bond opens exciting possibilities for small molecule activation. However, reactions of **1** with dihydrogen reveal partial cleavage of the NHC‐P bond and subsequent PH transfer. This reactivity has been successfully exploited in the synthesis of metalladiphosphiranes (**6**, **7**) and metallaphosphachalcogeni‐ranes (**8**, **9**) via targeted phosphinidene and chalcogen transfer, respectively.

The concept of identifying isolable 3d metal (NHC)P complexes can be readily extended, with nitrosyl complexes serving as a leitmotif. Consequently, a series of neutral and anionic (NHC)P complexes [(NHC)PM(CO)_n_] (M = V, n = 5; M = Mn, n = 4; M = Co, n = 3) and [(NHC)PM(CO)_n_]^–^ (M = Cr, n = 4; M = Fe, n = 3; Ni, n = 2] should be accessible. The overarching goal is to apply such systems in homogenous catalysis and further exploit the potential of (NHC)P ligand as novel phosphorus(I) ancillary ligands.^[^ ^7^ ^]^

## Conflicts of interest

The authors declare no conflict of interest.

## Supporting information



Supporting Information

## Data Availability

The data that support the findings of this study are available in the supplementary material of this article.

## References

[1] K. Schwedtmann , G. Zanoni , J. J. Weigand , Chem. Asian J. 2018, 13, 1388. 29573181 10.1002/asia.201800199

[2] T. Krachko , J. C. Slootweg , Eur. J. Inorg. Chem. 2018, 2018, 2734.

[3] A. Doddi , M. Peters , M. Tamm , Chem. Rev. 2019, 119, 6994. 30983327 10.1021/acs.chemrev.8b00791

[4] A. Doddi , D. Bockfeld , T. Bannenberg , P. G. Jones , M. Tamm , Angew. Chem., Int. Ed. 2014, 53, 13568. 10.1002/anie.201408354 25287885

[5] M. Peters , A. Doddi , T. Bannenberg , M. Freytag , P. G. Jones , M. Tamm , Inorg. Chem. 2017, 56, 10785. 28829597 10.1021/acs.inorgchem.7b01798

[6] A. Doddi , D. Bockfeld , T. Bannenberg , M. Tamm , Chem.‐Eur. J. 2020, 26, 14878. 32721063 10.1002/chem.202003099 PMC7756676

[7] J. Bhattacharjee , D. Bockfeld , M. Tamm , J. Org. Chem. 2022, 87, 1098. 35007063 10.1021/acs.joc.1c02377

[8] A. Doddi , T. Bannenberg , D. Bockfeld , M. Tamm , Z. Anorg. Allg. Chem. 2023, 649.

[9] H. Aktaş , J. C. Slootweg , K. Lammertsma , Angew. Chem., Int. Ed. 2010, 49, 2102. 10.1002/anie.200905689 20157897

[10] H. M. Gottschling , M. Balmer , R.‐M. Richter , C. von Hänisch , Z. Anorg. Allg. Chem. 2023, 649.

[11] O. Lemp , M. Balmer , K. Reiter , F. Weigend , C. von Hänisch , Chem. Commun. 2017, 53, 7620. 10.1039/c7cc04422d 28639632

[12] M. K. Sharma , H. M. Weinert , C. Wölper , S. Schulz , Chem.‐Eur. J. 2024, 30, e202400110. 38235843 10.1002/chem.202400110

[13] M. Balmer , C. von Hänisch , Z. Anorg. Allg. Chem. 2018, 644, 1143.

[14] Y. Wang , T. Szilvási , S. Yao , M. Driess , Nat. Chem. 2020, 12, 801. 32807885 10.1038/s41557-020-0518-0

[15] J. Bhattacharjee , M. Peters , D. Bockfeld , M. Tamm , Chem.‐Eur. J. 2021, 27, 5913. 33555047 10.1002/chem.202100482 PMC8048956

[16] M. E. Doleschal , A. Kostenko , J. Y. Liu , S. Inoue , Nat. Chem. 2024, 16,. 10.1038/s41557-024-01618-6 PMC11611736 39256544

[17] M. Balmer , Y. J. Franzke , F. Weigend , C. von Hänisch , Chem.‐Eur. J. 2020, 26, 192. 31702848 10.1002/chem.201905061 PMC6972534

[18] V. Nesterov , R. Baierl , F. Hanusch , A. E. Ferao , S. Inoue , J. Am. Chem. Soc. 2019, 141, 14576. 31476856 10.1021/jacs.9b08741

[19] Z. Li , X. Chen , Y. Li , C.‐Y. Su , H. Grützmacher , Chem. Commun. 2016, 52, 11343. 10.1039/c6cc05916c 27545980

[20] H. Chen , Y. Chen , T. Li , D. Wang , L. Xu , G. Tan , Inorg. Chem. 2023, 62, 20906. 38095884 10.1021/acs.inorgchem.3c03353

[21] M. Balmer , H. Gottschling , C. von Hänisch , Chem. Commun. 2018, 54, 2659. 10.1039/c8cc00899j 29479606

[22] L. P. Ho , M.‐K. Zaretzke , T. Bannenberg , M. Tamm , Chem. Commun. 2019, 55, 10709. 10.1039/c9cc05739k 31429453

[23] A. Doddi , D. Bockfeld , M.‐K. Zaretzke , T. Bannenberg , M. Tamm , Chem.‐Eur. J. 2019, 25, 13119. 31433085 10.1002/chem.201903795 PMC6856684

[24] A. M. Tondreau , Z. Benkő , J. R. Harmer , H. Grützmacher , Chem. Sci. 2014, 5, 1545.

[25] M. Bispinghoff , A. M. Tondreau , H. Grützmacher , C. A. Faradji , P. G. Pringle , Dalton Trans. 2016, 45, 5999. 26122315 10.1039/c5dt01741f

[26] R. Weller , M. Balmer , C. von Hänisch , C. G. Werncke , Dalton Trans. 2022, 51, 1765. 35013743 10.1039/d1dt03805b

[27] M. Piesch , S. Reichl , M. Seidl , G. Balázs , M. Scheer , Angew. Chem., Int. Ed. 2019, 58, 16563. 10.1002/anie.201908397 31573128

[28] G. Hierlmeier , A. Hinz , R. Wolf , J. M. Goicoechea , Angew. Chem., Int. Ed. 2018, 57, 431. 10.1002/anie.201710582 29152826

[29] R. A. Schulz , U. S. Karaca , M. Diefenbach , N. J. A. Werthmann , S. Dechert , M. M. Hansmann , M. C. Holthausen , F. Meyer , Chem.‐Eur. J. 2024, e202404095. 39584492 10.1002/chem.202404095

[30] G. B. Richter‐Addo , P. Legzdins , Metal nitrosyls, Oxford University Press, New York, NY, 1992.

[31] D. Michael , P. Mingos , D. J. Sherman , in ** *Advances in Inorganic Chemistry* ** (Ed.: A. G. Sykes), Academic Press, 1989, pp. 293–377.

[32] T. W. Hayton , P. Legzdins , W. B. Sharp , Chem. Rev. 2002, 102, 935. 11942784 10.1021/cr000074t

[33] J. H. Enemark , R. D. Feltham , Coord. Chem. Rev. 1974, 13, 339.

[34] H. Wawersik , F. Basolo , J. Am. Chem. Soc. 1967, 89, 4626.

[35] L. Rosenberg , Coord. Chem. Rev. 2012, 256, 606.

[36] A. G. Bakhoda , Inorg. Chem. 2025, 64, 1930. 39829228 10.1021/acs.inorgchem.4c04673

[37] D. C. Lacy , P. C. Abhyankar , Chem.‐Eur. J. 2023, 29, e202300518. 37078974 10.1002/chem.202300518

[38] M. Gediga , S. H. Schlindwein , J. Bender , M. Nieger , D. Gudat , Angew. Chem., Int. Ed. 2017, 56, 15718. 10.1002/anie.201709015 28980421

[39] M. Gediga , C. M. Feil , S. H. Schlindwein , J. Bender , M. Nieger , D. Gudat , Chem.‐Eur. J. 2017, 23, 11560. 28560855 10.1002/chem.201701442

[40] M. Papendick , C. M. Feil , M. Nieger , D. Gudat , Z. Anorg. Allg. Chem. 2018, 644, 1006.

[41] M. Papendick , D. Gudat , Chem.‐Eur. J. 2023, 29, e202302525. 37650872 10.1002/chem.202302525

[42] M. Papendick , N. Birchall , M. Nieger , D. Gudat , Organometallics 2024, 43.

[43] S. Burck , J. Daniels , T. Gans‐Eichler , D. Gudat , K. Nättinen , M. Nieger , Z. Anorg. Allg. Chem. 2005, 631, 1403.

[44] H. Braunschweig , J. O. C. Jimenez‐Halla , K. Radacki , R. Shang , Angew. Chem., Int. Ed. 2016, 55, 12673. 10.1002/anie.201603548 27621216

[45] O. J. Scherer , E. Franke , J. Kaub , Angew. Chem. Int. Ed. Engl. 1986, 25, 96.

[46] H. Lang , M. Leise , C. Emmerich , J. Organomet. Chem. 1991, 418, C9.

[47] S. Weller , S. H. Schlindwein , C. M. Feil , Z. Kelemen , D. Buzsáki , L. Nyulászi , S. Isenberg , R. Pietschnig , M. Nieger , D. Gudat , Organometallics 2019, 38, 4717. 10.1039/c9dt00892f 30984960

[48] D. A. DuBois , E. N. Duesler , R. T. Paine , Inorg. Chem. 1985, 24, 3.

[49] A. T. Termaten , H. Aktas , M. Schakel , A. W. Ehlers , M. Lutz , A. L. Spek , K. Lammertsma , Organometallics 2003, 22, 1827.

[50] B. T. Sterenberg , K. A. Udachin , A. J. Carty , Organometallics 2003, 22, 3927.

[51] J. Sánchez‐Nieves , B. T. Sterenberg , K. A. Udachin , A. J. Carty , J. Am. Chem. Soc. 2003, 125, 2404. 12603123 10.1021/ja028303b

[52] R. Melenkivitz , D. J. Mindiola , G. L. Hillhouse , J. Am. Chem. Soc. 2002, 124, 3846. 11942818 10.1021/ja017787t

[53] S. Niu , M. B. Hall , Chem. Rev. 2000, 100, 353. 11749240 10.1021/cr980404y

[54] J. A. S. Howell , P. M. Burkinshaw , Chem. Rev. 1983, 83, 557.

[55] Q. Z. Shi , T. G. Richmond , W. C. Trogler , F. Basolo , J. Am. Chem. Soc. 1984, 106, 71.

[56] J. R. Chipperfield , J. Organomet. Chem. 1989, 363, 253.

[57] K. J. Laldler , M. C. King , J. Phys. Chem. 1983, 87, 2657.

[58] G. T. Palmer , F. Basolo , J. Am. Chem. Soc. 1985, 107, 3122.

[59] R. B. Hitam , K. A. Mahmoud , A. J. Rest , J. Organomet. Chem. 1985, 291, 321.

[60] A. J. Arduengo , III, H. V. R. Dias , J. C. Calabrese , Chem. Lett. 1997, 143.

[61] D. Bockfeld , T. Bannenberg , P. G. Jones , M. Tamm , Eur. J. Inorg. Chem. 2017, 2017, 3452.

[62] T. Krachko , M. Bispinghoff , A. M. Tondreau , D. Stein , M. Baker , A. W. Ehlers , J. C. Slootweg , H. Grützmacher , Angew. Chem., Int. Ed. 2017, 56, 7948. 10.1002/anie.201703672 28505382

[63] L. Weber , Chem. Rev. 1992, 92, 1839.

[64] E. Lindner , E. Ossig , M. Darmuth , J. Organomet. Chem. 1989, 379, 107.

[65] E. Lindner , M. Darmuth , H. A. Mayer , R. Fawzi , C. Maichle , M. Steimann , Chem. Ber. 1993, 126, 23.

[66] C. Taube , J. Fidelius , K. Schwedtmann , C. Ziegler , F. Kreuter , L. Loots , L. J. Barbour , R. Tonner‐Zech , R. Wolf , J. J. Weigand , Angew. Chem., Int. Ed. 2023, 62, e202306706. 10.1002/anie.202306706 37671442

[67] S. S. Batsanov , Inorg. Mater. 2001, 37, 871.

[68] E. Lindner , E. Bosch , R. Fawzi , M. Steimann , H. A. Mayer , K. Gierling , Chem. Ber. 1996, 129, 945.

[69] H.‐U. Reisacher , W. F. McNamara , E. N. Duesler , R. T. Paine , Organometallics 1997, 16, 449.

[70] D. Rottschäfer , M. K. Sharma , B. Neumann , H.‐G. Stammler , D. M. Andrada , R. S. Ghadwal , Chem.‐Eur. J. 2019, 25, 8127. 31038217 10.1002/chem.201901204

[71] T. Sasamori , Y. Suzuki , M. Sakagami , H. Miyake , N. Tokitoh , Chem. Lett. 2014, 43, 1464.

[72] T. Sasamori , N. Takeda , N. Tokitoh , J. Phys. Org. Chem. 2003, 16, 450.

[73] A. Tsurusaki , N. Nagahora , T. Sasamori , K. Matsuda , Y. Kanemitsu , Y. Watanabe , Y. Hosoi , Y. Furukawa , N. Tokitoh , Bull. Chem. Soc. Jpn. 2010, 83, 456.

[74] H. Voelker , U. Pieper , H. W. Roesky , G. M. Sheldrick , Z. Naturforsch. B 1993, 49, 255.

