# Supplementary material for: Synthesis and Reactivity of N‐Heterocyclic Carbene‐Phosphinidene Manganese Complexes
Source: Chemistry. 2025 Apr 3;31(25):e202500997. doi: 10.1002/chem.202500997 (PMC12057602; doi:10.1002/chem.202500997)
Supplement: Supplementary file 1 — Supporting Information [file CHEM-31-e202500997-s001.pdf]

Supporting Information

Synthesis and Reactivity of N-heterocyclic Carbene-Phosphinidene Manganese Complexes

Dustin Bockhardt, Thomas Bannenberg, and Matthias Tamm*

*Institut für Anorganische und Analytische Chemie, Technische Universität Braunschweig, Hagenring 30, 38106 Braunschweig, Germany; Tel.: +495313915309; E-mail: m.tamm@tu-bs.de

Table of Contents

S1. General 1

S2. Experimental Procedures and Analysis Data 2

S2.1. Synthesis of (IDipp)PMn(CO)$_4$ 2

S2.2. Synthesis of (IDipp)PMn(PPh$_3$)(CO)$_3$ 3

S2.3. Synthesis of (IDipp)PMn(PMe$_3$)(CO)$_3$ 4

S2.4. Synthesis of (IDipp)PMn(IMe)(CO)$_3$ 5

S2.5. Synthesis of (IDipp)PMn(XyNC)(CO)$_3$ 6

S2.6. Synthesis of {(IDipp)P=PH}Mn(CO)$_4$ 7

S2.7. Synthesis of {(IDipp)P=PPh}Mn(CO)$_4$ 8

S2.8. Synthesis of {(IDipp)P=Se}Mn(CO)$_4$ 9

S2.9. Synthesis of {(IDipp)P=Te}Mn(CO)$_4$ 10

S3. NMR Spectra 12

S3.1. (IDipp)PMn(CO)$_4$ 12

S3.2. (IDipp)PMn(PPh$_3$)(CO)$_3$ 21

S3.3. (IDipp)PMn(PMe3)(CO)3 29

S3.4. (IDipp)PMn(IMe)(CO)3 34

S3.5. (IDipp)PMn(XyNC)(CO)$_3$ 38

S3.6. {(IDipp)P=PH}Mn(CO)4 42

S3.7. {(IDipp)P=PPh}Mn(CO)$_4$ 47

S3.8. {(IDipp)P=Se}Mn(CO)$_4$ 52

S3.9. {(IDipp)P=Te}Mn(CO)$_4$ 57

S4. Preliminary Reactivity Studies with Dihydrogen 61
S5. Investigation of the CO Substitution Reaction with PPh_3 at Variable Temperatures 63
S6. IR Spectra 67
S7. UV/Vis Spectra 72
S8. Single Crystal X-Ray Diffraction Analysis Details 77
S8.1. General 77
S8.2. Refinement and data handling special details are listed in the crystal data and structure refinement tables (Crystal Data and Structure Refinement Tables 77
S8.3. Crystal Data and Structure Refinement Tables 78
S8.4. Molecular Structures and Structural Details 88
S9. Kinetic Studies 93
S9.1. Theory 93
S9.2. Experiments 94
S10. Computational Details 107
S10.1. General Information and Energies of Optimized Structures 107
S10.2. NBO Analysis 110
S11. References 115

S1. General

Unless otherwise noted, all reactions have been performed under dry argon atmosphere in a Glove Box (MBraun 200B Eco) or using a high vacuum line with common Schlenk techniques. Elevated temperatures were achieved by a silicone oil bath, whereas low temperatures were provided by an isopropanol cooling bath.

$(CO)_5MnBr$,[1] (IDipp)$PSiMe_3$,[2] (IDipp)PH,[2] (IMe)PPh,[3] PMe_3[4,5] and IMe[6] were synthesized by literature procedures. All other starting materials were obtained from commercial sources (Sigma-Aldrich, Alfa-Aesar, Roth, TCI, abcr, VWR or Fisher Chemical and used without further purification.

Filtration media Celite® 545 and aluminum oxide (neutral, Brockmann I, for chromatography, 50–200 m, 60Å) were purchased from Acros Organics and dried at 120 °C at least 24 h prior to use.

Solvents were dried either over Na/benzophenone, CaH_2 (chlorinated solvents) and distilled or dried by a SPS (solvent purification system) from MBraun with subsequent degassing (ultrasonic bath) and afterwards stored under dry argon atmosphere over molecular sieves (3–5 Å).

NMR spectra were recorded with Bruker AV III-400 (400 MHz), Bruker AV III-HD500 (500 MHz) and Bruker AV II-600 (600 MHz) spectrometers. The chemical shifts are given in parts per million (δ; ppm) relative to residual solvent ^1H signals (δ = 7.16 (C_6D_6), 3.58 (THF-d_8) ppm) or to the integral standard tetramethylsilane (δ = 0.00 ppm) when using CD_2Cl_2, or to the ^{13}C resonance of the solvents (δ = 128.1 (C_6D_6), 67.2 (THF-d_8), 53.8 (CD_2Cl_2) ppm). All other spectra were calibrated using external references (^{31}P: 85% $H_3PO_{4(aq)}$, ^{77}Se: Me_2Se, ^{125}Te: Me_2Te). Coupling constants (*J*) are reported in Hertz (Hz) and splitting patterns are indicated as s (singlet), d (doublet), t (triplet), m (multiplet), sept (septet), br (broad) and s_{br} (broad singlet). The number of protons attached to each carbon atom was determined by ^{13}C-DEPT135 experiments. The assignment of signals was supported by 2D experiments (^1H,^1H COSY; ^1H,^{13}C HSQC; ^1H,^{13}C HMBC). ^{15}N chemical shifts were determined by ^1H,^{15}N HMBC experiments. Data handling and visualization was done using TopSpin[7] by Bruker. Temperatures were determined experimentally by measurement of CH_3OH in CH_3OD.

Elemental analyses were carried out on a Vario Micro Cube System.

UV/Vis spectra were recorded in THF solution on a Varian Cary 50 Scan spectrophotometer.

IR spectra were recorded with a Bruker Vertex 70 spectrometer either in THF solutions in a cuvette of an approximately 1 mm optical path length equipped with NaCl windows or equipped with a Pike Technologies MIRacle attenuated total reflectance (ATR) unit (ZnSe crystal) with neat samples.

UV/Vis, IR data were manipulated using Python 3.12 with the libraries NumPy[8], Pandas[9,10], SciPy[11], Uncertainties[12] and plotted with Matplotlib.[13]

S2. Experimental Procedures and Analysis Data

S2.1. Synthesis of (IDipp)PMn(CO)4

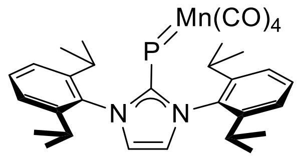

In a Schlenk flask, $Mn(CO)_5Br$ (167 mg, 0.609 mmol, 1.00 eq.) was mixed with toluene (5 mL) resulting in an orange suspension. (IDipp)$PSiMe_3$ (300 mg, 0.609 mmol, 1.00 eq.), dissolved in toluene (15 mL, yellow), was added at room temperature. A few minutes after the addition, the solution began to darken, resulting in a yellow-green solution after approximately 30 minutes. The reaction mixture was allowed to stir at room temperature for further 16 h with slightly reduced pressure (vacuum was pulled onto the flask until the solution started bubbling vigorously, then the flask was sealed). The solvent was removed under reduced pressure leaving behind a dark green residue which was dissolved in THF (5 mL), layered with *n*-hexane (20 mL) and then stored at −27 °C for at least 24 h resulting in the formation of dark green crystals. The solution was decanted with a syringe, the product washed with *n*-pentane (2 x 5 mL) and dried under high vacuum. (IDipp)$PMn(CO)_4$ was obtained as a dark green solid (248 mg, 0.422 mmol, 69 %).

Single crystals suitable for x-ray diffraction analysis could be grown from a saturated of solution (IDipp)$PMn(CO)_4$ in a toluene/*n*-pentane mixture at −40 °C.

^{1}H NMR (500 MHz, CD_2Cl_2, 298 K): δ = 1.17 (12H, d, $^{3}J_{H,H}$ = 5.9 Hz, CH(C$\underline{H}_3$)$_2$), 1.29 (12H, d, $^{3}J_{H,H}$ = 6.7 Hz, CH(C$\underline{H}_3$)$_2$), 2.65–3.50 (4H, m ("s$_{br}$"), C$\underline{H}$(CH$_3$)$_2$), 7.25 (4H, d, $^{3}J_{H,H}$ = 7.8 Hz, *m*-C$\underline{H}_{Dipp}$), 7.42 (2H, t, $^{3}J_{H,H}$ = 7.8 Hz, *p*-C$\underline{H}_{Dipp}$), 7.44 (2H, s, C$\underline{H}_{Imidazolin}$) ppm.

^{1}H NMR (400 MHz, CD_2Cl_2, 215 K): δ = 1.06 (6H, d, $^{3}J_{H,H}$ = 6.8 Hz, CH(C$\underline{H}_3$)$_2$), 1.24 (6H, d, $^{3}J_{H,H}$ = 6.7 Hz, CH(C$\underline{H}_3$)$_2$), 1.30 (6H, d, $^{3}J_{H,H}$ = 6.7 Hz, CH(C$\underline{H}_3$)$_2$), 1.34 (6H, d, $^{3}J_{H,H}$ = 6.6 Hz, CH(C$\underline{H}_3$)$_2$), 2.55–2.70 (2H, m ("sept", $^{3}J_{H,H}$ = 6.7 Hz), C$\underline{H}$(CH$_3$)$_2$), 3.29–3.42 (2H, m ("sept", $^{3}J_{H,H}$ = 6.6 Hz), C$\underline{H}$(CH$_3$)$_2$), 7.24 (2H, dd, $^{3}J_{H,H}$ = 7.7 Hz, $^{4}J_{H,H}$ = 1.0 Hz, *m*-C$\underline{H}_{Dipp}$), 7.34 (2H, dd, $^{3}J_{H,H}$ = 7.8 Hz, $^{4}J_{H,H}$ = 1.0 Hz, *m*-C$\underline{H}_{Dipp}$), 7.49 (2H, t, $^{3}J_{H,H}$ = 7.8 Hz, *p*-C$\underline{H}_{Dipp}$), 7.51 (2H, s, C$\underline{H}_{Imidazolin}$) ppm.

^{1}H NMR (300 MHz, C_6D_6, 298 K): δ = 0.98 (12H, d, $^{3}J_{H,H}$ = 6.85 Hz, CH(C$\underline{H}_3$)$_2$), 1.36 (12H, d, $^{3}J_{H,H}$ = 6.58 Hz, CH(C$\underline{H}_3$)$_2$), 2.65–3.50 (4H, m ("s$_{br}$"), C$\underline{H}$(CH$_3$)$_2$), 6.64 (2H, s, C$\underline{H}_{Imidazolin}$), 6.94 (4H, d, $^{3}J_{H,H}$ = 7.6 Hz, *m*-C$\underline{H}_{Dipp}$), 7.05 (2H, t, $^{3}J_{H,H}$ = 7.7 Hz, *p*-C$\underline{H}_{Dipp}$) ppm.

^{13}C{^{1}H} NMR (125 MHz, CD_2Cl_2, 298 K): δ = 22.8 (s, CH($\underline{C}$H$_3$)$_2$), 26.0 (s, CH($\underline{C}$H$_3$)$_2$), 29.0 (s, $\underline{C}$H(CH$_3$)$_2$), 125.0 (s, *m*-$\underline{C}$H$_{Dipp}$), 125.6 (s, $\underline{C}$H$_{Imidazolin}$), 131.1 (s, *p*-$\underline{C}$H$_{Dipp}$), 133.1 (s, $\underline{C}$-N), 146.3 (s, $\underline{C}$-*i*Pr), 186.9 (d, $^{1}J_{P,C}$ = 156 Hz, N$\underline{C}$N), 222.8 (s$_{br}$, $\underline{C}$O$_{ax}$), 232.4 (s, $\underline{C}$O$_{eq}$) ppm.

^{31}P{^{1}H} NMR (202.5 MHz, CD_2Cl_2, 298 K): δ = 627.5 (s) ppm.

^{31}P{^{1}H} NMR (121.5 MHz, C_6D_6, 298 K): δ = 616.7 (s) ppm.

^{31}P{^{1}H} NMR (162 MHz, toluene-*d*$_8$, 298 K): δ = 617.2 (s) ppm.

^{1}H,^{15}N HMBC (51 MHz, CD_2Cl_2, 298 K): $\delta_N = -202$ ppm.

IR (THF): $\tilde{\nu}$ = 2029 *v*(C-O), 1948 *v*(C-O), 1931 *v*(C-O), 1915 *v*(C-O) cm^{-1}.

IR (ZnSe-ATR): $\tilde{\nu}$ = 2027 *v*(C-O), 1934 *v*(C-O), 1907 *v*(C-O), 1892 *v*(C-O) cm^{-1}.

UV/Vis (THF): $\lambda_{\max}$ = 356, 646 nm.

Elemental analysis (%) calcd. for $C_{31}H_{36}MnN_2O_4P$ (586.55 g mol^{-1}): C 63.48, H 6.19, N 4.78; found: C 63.64, H 6.22, N 4.71.

S2.2. Synthesis of (IDipp)PMn(PPh$_3$)(CO)$_3$

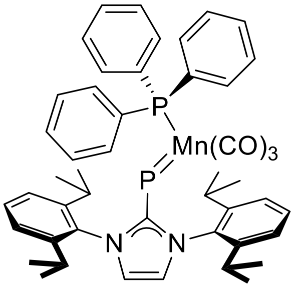

In a Schlenk flask, (IDipp)PMn(CO)$_4$ (50 mg, 0.085 mmol, 1.00 eq.) was dissolved in toluene (3 mL), resulting in a dark green solution. Triphenylphosphine (22 mg, 0.085 mmol, 1.00 eq.) was dissolved in toluene (2 mL, colorless) and, with strong stirring, added dropwise to the green solution at room temperature. Stirring was continued for 16 h and afterwards all volatiles were removed under reduced pressure. The dark green residue was washed with *n*-pentane (3x 1 mL), dried under high vacuum and extracted with toluene (3 mL). After filtration through a piece of tissue, the solvent was removed under reduced pressure. (IDipp)P(PPh$_3$)Mn(CO)$_3$ was obtained as a green solid (42 mg, 0.051 mmol, 60 %).

Single crystals suitable for x-ray diffraction analysis could be grown by vapor diffusion of *n*-hexane into a saturated solution of (IDipp)PMn(PPh$_3$)(CO)$_3$ in C_6D_6 at room temperature.

^{1}H NMR (500 MHz, CD_2Cl_2, 298 K): δ = 0.48–0.82 (6H, m (“s$_{br}$”), CH(C*H*$_3$)$_2$), 0.82–1.06 (6H, m (“s$_{br}$”), CH(C*H*$_3$)$_2$), 1.15–1.35 (6H, m (“s$_{br}$”), CH(C*H*$_3$)$_2$), 1.35–1.59 (6H, m (“s$_{br}$”), CH(C*H*$_3$)$_2$), 2.26–2.66 (2H, m (“s$_{br}$”), C*H*(CH$_3$)$_2$), 3.31–3.77 (2H, m (“s$_{br}$”), C*H*(CH$_3$)$_2$), 7.08–7.29 (19H, m (“2x td; 1x tq”), *m*-C*H*$_{Dipp}$, *o*-C*H*$_{Phen}$, *m*-C*H*$_{Phen}$, *p*-C*H*$_{Phen}$), 7.34 (2H, s, C*H*$_{Imidazolin}$), 7.46 (2H, t, $^{3}J_{H,H}$ = 7.8 Hz, *p*-C*H*$_{Dipp}$) ppm.

^{1}H NMR (400 MHz, CD_2Cl_2, 260 K): δ = 0.66 (6H, d, $^{3}J_{H,H}$ = 6.7 Hz, CH(C*H*$_3$)$_2$), 0.93 (6H, d, $^{3}J_{H,H}$ = 6.8 Hz, CH(C*H*$_3$)$_2$), 1.29 (6H, d, $^{3}J_{H,H}$ = 6.8 Hz, CH(C*H*$_3$)$_2$), 1.43 (6H, d, $^{3}J_{H,H}$ = 6.6 Hz, CH(C*H*$_3$)$_2$), 2.39–2.52 (2H, m (“quint”), C*H*(CH$_3$)$_2$), 3.48–3.63 (2H, m (“quint”), C*H*(CH$_3$)$_2$), 7.12–7.37 (19H, m, *m*-C*H*$_{Dipp}$, *o*-C*H*$_{Phen}$, *m*-C*H*$_{Phen}$, *p*-C*H*$_{Phen}$), 7.37 (2H, s, C*H*$_{Imidazolin}$), 7.49 (2H, t, $^{3}J_{H,H}$ = 7.7 Hz, *p*-C*H*$_{Dipp}$) ppm.

^{1}H NMR (300 MHz, C_6D_6, 298 K): δ = 0.60–1.20 (18H, m (“3 x s$_{br}$”), CH(C*H*$_3$)$_2$), 0.82–1.06 1.45–1.83 (6H, m (“s$_{br}$”), CH(C*H*$_3$)$_2$), 2.40–2.74 (2H, m (“s$_{br}$”), C*H*(CH$_3$)$_2$), 3.65–3.92 (2H, m (“s$_{br}$”), 6.69 (2H, s, C*H*$_{Imidazolin}$), C*H*(CH$_3$)$_2$), 6.92–7.09 (13H, m), 7.20 (2H, t, $^{3}J_{H,H}$ = 7.8 Hz, *p*-C*H*$_{Dipp}$), 7.50–7.60 (6H, m (“tt”)) ppm.

^{13}C{^{1}H} NMR (125 MHz, CD_2Cl_2, 298 K): δ = 21.5–23.8 (m, CH(*C*H$_3$)$_2$), 25.0–27.0 (m, CH(*C*H$_3$)$_2$), 28.2–29.7 (m, *C*H(CH$_3$)$_2$), 124.4 (s, *C*H$_{Imidazolin}$), 125.6 (s$_{br}$, *m*-*C*H$_{Dipp}$), 127.7 (d, $J_{C,P}$ = 9.1 Hz, *C*H$_{Phen}$), 129.3 (d, $J_{C,P}$ = 1.8 Hz, *C*H$_{Phen}$), 130.2 (s, *p*-*C*H$_{Dipp}$), 133.9 (s, *C*-N),

135.1 (d, $J_{C,P}$ = 10.1 Hz, $\underline{C}H_{Phen}$), 136.9 (d, $J_{C,P}$ = 36.7 Hz, $\underline{C}H_{Phen}$), 146.2–147.4 (m, $\underline{C}$-*i*Pr), 191.5 (dd, $^1J_{C,P}$ = 145 Hz, $^2J_{C,P}$ = 39 Hz, N$\underline{C}$N), 226.9 (s$_{br}$, $\underline{C}O_{eq}$), 235.3 (s$_{br}$, $\underline{C}O_{ax}$) ppm.

^{31}P{^{1}H} NMR (202.5 MHz, CD_2Cl_2, 298 K): δ = 69.2 (d, $^2J_{P,P}$ = 65 Hz, $\underline{P}Ph_3$), 570.6 (d, $^2J_{P,P}$ = 67 Hz, IDipp-$\underline{P}$) ppm.

^{31}P{^{1}H} NMR (121.5 MHz, C_6D_6, 298 K): δ = 70.0 (d, $^2J_{P,P}$ = 65 Hz, $\underline{P}Ph_3$), 549.6 (d, $^2J_{P,P}$ = 68 Hz, IDipp-$\underline{P}$) ppm.

^{31}P{^{1}H} NMR (162 MHz, toluene-*d*8, 298 K): δ = 70.4 (d, $^2J_{P,P}$ = 67 Hz, $\underline{P}Ph_3$), 547.5 (d, $^2J_{P,P}$ = 68 Hz, IDipp-$\underline{P}$) ppm.

^{1}H,^{15}N HMBC (51 MHz, CD_2Cl_2, 298 K): δ_N = −205 ppm.

IR (THF): $\tilde{\nu}$ = 1963 *ν*(C-O), 1882 *ν*(C-O), 1871 *ν*(C-O) cm^{-1}.

UV/Vis (THF): λ_{max} = 285, 382, 635 nm.

Elemental analysis (%) calcd. for $C_{31}H_{36}MnN_2O_4P$ (820.83 g mol^{-1}): C 70.24, H 6.26, N 3.41; found: C 70.66, H 6.226, N 3.13.

S2.3. Synthesis of (IDipp)PMn(PMe3)(CO)3

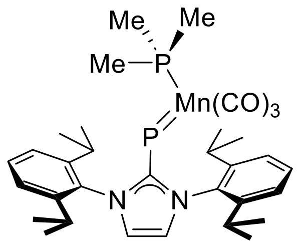

In a Schlenk flask, (IDipp)PMn(CO)4 (50 mg, 0.085 mmol, 1.00 eq.) was dissolved in toluene (3 mL), resulting in a dark green solution. Trimethylphosphine (0.102 mL, 1M in toluene, 0.085 mmol, 1.00 eq.) was diluted with toluene (1 mL, colorless) and, with strong stirring, added dropwise to the green solution at room temperature. The addition resulted immediately in a slight yellow tint of the reaction mixture. Stirring was continued for 3 h and afterwards all volatiles were removed under reduced pressure. The dark green residue was washed with *n*-hexane (1 mL), dried under high vacuum and extracted with toluene (3 mL). After filtration through a piece of tissue, the solvent was removed under reduced pressure, resulting in the formation of black crystals of (IDipp)P(PMe3)Mn(CO)3 (30 mg, 0.047 mmol, 55 %).

Single crystals suitable for x-ray diffraction analysis could be grown by vapor diffusion of *n*-hexane into a saturated solution of (IDipp)PMn(PMe3)(CO)3 in C_6D_6 at room temperature.

^{1}H NMR (500 MHz, CD_2Cl_2, 298 K): δ = 1.07 (12H, d, $^3J_{H,H}$ = 6.9 Hz, CH(C$\underline{H}_3$)2), 1.22 (12H, d, $^3J_{H,H}$ = 6.8 Hz, CH(C$\underline{H}_3$)2), 1.34 (9H, d, $^2J_{H,P}$ = 8.1 Hz, P(C$\underline{H}_3$)3), 2.92–3.16 (4H, m ("s$_{br}$"), C$\underline{H}$(CH3)2), 7.13 (4H, d, $^3J_{H,H}$ = 7.8 Hz, *m*-C$\underline{H}_{Dipp}$), 7.21 (2H, s, C$\underline{H}_{Imidazolin}$), 7.42 (2H, t, $^3J_{H,H}$ = 7.8 Hz, *p*-C$\underline{H}_{Dipp}$) ppm.

^{1}H NMR (300 MHz, C_6D_6, 298 K): δ = 1.06 (12H, d, $^3J_{H,H}$ = 6.8 Hz, CH(C$\underline{H}_3$)2), 1.24 (12H, d, $^3J_{H,H}$ = 8.0 Hz, CH(C$\underline{H}_3$)2), 1.50 (9H, d, $^2J_{H,P}$ = 6.6 Hz, P(C$\underline{H}_3$)3), 3.15–3.45 (4H, m ("s$_{br}$"), C$\underline{H}$(CH3)2), 6.66 (2H, s, C$\underline{H}_{Imidazolin}$), 7.13 (6H, *m*-C$\underline{H}_{Dipp}$, *p*-C$\underline{H}_{Dipp}$) ppm.

^{13}C{^{1}H} NMR (125 MHz, CD_2Cl_2, 298 K): δ = 19.6 (dd, $^{1}J_{C,P}$ = 26 Hz, $^{2}J_{C,P}$ = 10 Hz, P($\underline{C}H_3)_3$), 22.9 (s, CH($\underline{C}H_3)_2$), 25.9 (s, CH($\underline{C}H_3)_2$), 28.9 (s, $\underline{C}H(CH_3)_2$), 124.3 (s, $\underline{C}H_{Imidazolin}$), 124.6 (s, *m*-$\underline{C}H_{Dipp}$), 130.4 (s, *p*-$\underline{C}H_{Dipp}$), 134.4 (s, $\underline{C}$-N), 146.7 (s, $\underline{C}$-*i*Pr), 190.6 (dd, $^{1}J_{C,P}$ = 146 Hz, $^{2}J_{C,P}$ = 40 Hz, N$\underline{C}$N), 227.1 (s_{br}, $\underline{C}O_{eq}$), 233.8 (s, $\underline{C}O_{ax}$) ppm.

^{31}P{^{1}H} NMR (202.5 MHz, CD_2Cl_2, 298 K): δ = 17.6 (d, $^{2}J_{P,P}$ = 57 Hz, $\underline{P}Me_3$), 531.9 (d, $^{2}J_{P,P}$ = 60 Hz, IDipp-$\underline{P}$) ppm.

^{31}P NMR (202.5 MHz, CD_2Cl_2, 298 K): δ = 17.6 (m ("d, $^{2}J_{P,P}$ = 54 Hz"), $\underline{P}Me_3$), 531.9 (d, $^{2}J_{P,P}$ = 60 Hz, IDipp-$\underline{P}$) ppm.

^{31}P{^{1}H} NMR (121.5 MHz, C_6D_6, 298 K): δ = 17.7 (d, $^{2}J_{P,P}$ = 58 Hz), $\underline{P}Me_3$), 516.0 (d, $^{2}J_{P,P}$ = 58 Hz, IDipp-$\underline{P}$) ppm.

^{1}H,^{15}N HMBC (51 MHz, CD_2Cl_2, 298 K): δ_N = −204 ppm.

IR (THF): $\tilde{\nu}$ = 1961 ν(C-O), 1879 ν(C-O), 1861 ν(C-O) cm^{-1}.

UV/Vis (THF): λ_{max} = 287, 375, 613 nm.

Elemental analysis (%) calcd. for $C_{33}H_{36}MnN_2O_4P$ (634.62 g mol^{-1}): C 62.46, H 7.15, N 4.41; found: C 60.90[1], H 7.11, N 3.93.

S2.4. Synthesis of (IDipp)PMn(IMe)(CO)$_3$

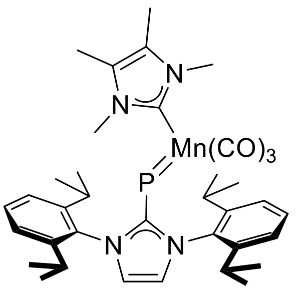

In a Schlenk flask, (IDipp)PMn(CO)$_4$ (50 mg, 0.085 mmol, 1.00 eq.) was dissolved in toluene (3 mL), resulting in a dark green solution. Tetramethylimidazolin-2-yliden (13 mg, 0.085 mmol, 1.00 eq.) was dissolved in toluene (1 mL) and, with strong stirring, added dropwise to the green solution at room temperature. The addition resulted immediately in a slight yellow tint of the reaction mixture and after addition of half of the reactant the solution turned dark yellow-orange. Stirring was continued for 2 h and afterwards all volatiles were removed under reduced pressure. The dark green residue was washed with *n*-hexane (2 x 1.5 mL), dried under high vacuum and extracted with toluene (3 x 1 mL). After filtration through a piece of tissue, the solvent was removed under reduced pressure. The dark solid was suspended three times in *n*-pentane (1 mL) and subsequently dried under reduced pressure to remove residual toluene. (IDipp)P(IMe)Mn(CO)$_3$ was obtained as a dark green-brown solid (44 mg, 0.064 mmol, 76 %).

Single crystals suitable for x-ray diffraction analysis could be grown by vapor diffusion of *n*-hexane into a saturated solution of (IDipp)PMn(IMe)(CO)$_3$ in C_6D_6 at room temperature.

^{1}H NMR (400 MHz, CD_2Cl_2, 298 K): δ = 1.06 (12H, d, $^{3}J_{H,H}$ = 6.1 Hz, CH($C\underline{H}_3)_2$), 1.13–1.31 (12H, m ("s_{br}"), CH($C\underline{H}_3)_2$), 1.98 (6H, s, $CC\underline{H}_3$), 2.84–3.32 (4H, m ("s_{br}"), $C\underline{H}(CH_3)_2$), 3.09

[1] A carbon percentage too low is observed likely due to carbide formation.

(6H, s, NC$\underline{H}_3$), 7.11–7.16 (4H, m ("d, $^3J_{H,H}$ = 7.8 Hz"), *m*-C$\underline{H}_{Dipp}$), 7.19 (2H, s, C$\underline{H}_{Imidazolin}$), 7.27 (2H, t, $^3J_{H,H}$ = 7.7 Hz, *p*-C$\underline{H}_{Dipp}$) ppm.

^{1}H NMR (300 MHz, C_6D_6, 298 K): δ = 0.99–1.16 (12H, m ("s$_{br}$"), CH(C$\underline{H}_3$)$_2$), 1.28 (6H, s, CC$\underline{H}_3$), 1.33–1.73 (12H, m ("s$_{br}$"), CH(C$\underline{H}_3$)$_2$), 3.09 (6H, s, NC$\underline{H}_3$), 6.70 (2H, s, C$\underline{H}_{Imidazolin}$), 6.97–7.14 (6H, m, *m*-C$\underline{H}_{Dipp}$, *p*-C$\underline{H}_{Dipp}$) ppm.[2]

^{13}C{^{1}H} NMR (101 MHz, CD_2Cl_2, 298 K): δ = 9.6 (s, C$\underline{C}$H$_3$), 22.8 (s, CH($\underline{C}$H$_3$)$_2$), 25.8 (s, CH($\underline{C}$H$_3$)$_2$), 28.7 (s, $\underline{C}$H(CH$_3$)$_2$), 36.0 (s, N$\underline{C}$H$_3$), 123.6 (s, $\underline{C}$H$_{Imidazolin}$), 124.3 (s, *m*-$\underline{C}$H$_{Dipp}$), 125.6 (s, $\underline{C}$CH$_3$), 130.0 (s, *p*-$\underline{C}$H$_{Dipp}$), 134.6 (s, $\underline{C}$-N), 146.8 (s, $\underline{C}$-*i*Pr), 190.9 (d, $^1J_{P,C}$ = 149 Hz, N$\underline{C}$N$_{Dipp}$), 197.2 (d, $^1J_{P,C}$ = 9.8 Hz, N$\underline{C}$N$_{IMe}$), 230.9 (s$_{br}$, $\underline{C}$O) ppm.

^{31}P{^{1}H} NMR (162 MHz, CD_2Cl_2, 298 K): δ = 461.9 (s) ppm.

^{31}P{^{1}H} NMR (121.5 MHz, C_6D_6, 298 K): δ = 442.3 (s) ppm.

^{1}H,^{15}N HMBC (41 MHz, CD_2Cl_2, 298 K): δ_N = −200 ($\underline{N}$-Me), −204 ($\underline{N}$-Dipp) ppm.

IR (THF): $\tilde{\nu}$ = 1952 *ν*(C-O), 1859 *ν*(C-O), 1852 *ν*(C-O) cm^{-1}.

UV/Vis (THF): λ_{max} = 301, 390, 606 nm.

Elemental analysis (%) calcd. for $C_{37}H_{48}MnN_4O_3P$ (682.73 g mol^{-1}): C 65.09, H 7.09, N 8.21; found: C 63.85[3], H 6.96, N 7.85.

S2.5. Synthesis of (IDipp)PMn(XyNC)(CO)$_3$

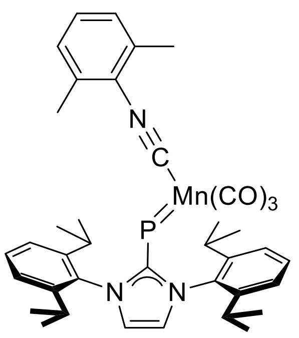

In a Schlenk flask, (IDipp)PMn(CO)$_4$ (50 mg, 0.085 mmol, 1.00 eq.) was dissolved in toluene (5 mL), resulting in a dark green solution. (2,5-Dimethylphenyl)isocyanide (11 mg, 0.085 mmol, 1.00 eq.) was dissolved in toluene (4 mL) and afterwards added dropwise with strong stirring. An immediate color change occurred, giving the dark green solution a yellow tint. The reaction mixture was stirred for 2 h at room temperature. Then, the solvent was removed under reduced pressure. The black residue was crystallized from benzene (1 mL) layered with *n*-hexane (3 mL) at room temperature. Dark green crystals formed after 3 days. The solution was decanted, the crystals washed with a minimal amount of *n*-pentane (0.5 mL) and dried under high vacuum to give {IDipp)P}Mn(XyNC)(CO)$_3$ as a green solid (33 mg, 0.048 mmol, 56 %).

Single crystals suitable for x-ray diffraction analysis could be grown by diffusion of *n*-hexane into a saturated solution of {IDipp)P}Mn(XyNC)(CO)$_3$ in benzene at room temperature.

^{1}H NMR (500 MHz, CD_2Cl_2, 298 K): δ = 1.17 (12H, d, $^3J_{H,H}$ = 4.9 Hz, CH(C$\underline{H}_3$)$_2$), 1.31 (12H, d, $^3J_{H,H}$ = 7.0 Hz, CH(C$\underline{H}_3$)$_2$), 2.44 (6H, s, CC$\underline{H}_3$), 2.86–3.40 (4H, m ("s$_{br}$"), C$\underline{H}$(CH$_3$)$_2$), 7.02–

[2] The signal of C$\underline{H}$(CH$_3$)$_2$ is not observed probably because of strong broadening.
[3] A carbon percentage too low is observed likely due to carbide formation.

7.10 (3H, m, C$\underline{H}_{Xy}$), 7.20–7.28 (4H, m ("d"), *m*-C$\underline{H}_{Dipp}$), 7.35 (2H, s, C$\underline{H}_{Imidazolin}$), 7.37–7.44 (2H, m ("t"), *p*-C$\underline{H}_{Dipp}$) ppm.

^{1}H NMR (300 MHz, C_6D_6, 298 K): δ = 1.06 (12H, d, $^3J_{H,H}$ = 6.8 Hz, CH(C$\underline{H}_3$)$_2$), 1.49 (12H, d, $^3J_{H,H}$ = 5.7 Hz, CH(C$\underline{H}_3$)$_2$), 2.18 (6H, s, CC$\underline{H}_3$), 3.02–3.60 (4H, m ("s_{br}"), C$\underline{H}$(CH$_3$)$_2$), 6.56–6.63 (2H, m ("d"), *m*-C$\underline{H}_{Xy}$), 6.65–6.72 (1H, m ("t"), *p*-C$\underline{H}_{Xy}$), 6.69 (2H, s, C$\underline{H}_{Imidazolin}$), 6.97–7.11 (6H, m, *m*-C$\underline{H}_{Dipp}$, *p*-C$\underline{H}_{Dipp}$) ppm.

^{13}C{^{1}H} NMR (125 MHz, CD_2Cl_2, 298 K): δ = 19.1 (s, C$\underline{C}$H$_3$), 22.9 (s, CH($\underline{C}$H$_3$)$_2$), 25.9 (s, CH($\underline{C}$H$_3$)$_2$), 28.9 (s, $\underline{C}$H(CH$_3$)$_2$), 124.8 (s, *m*-$\underline{C}$H$_{Dipp}$; s, $\underline{C}$H$_{Imidazolin}$), 127.6 (s, *p*-$\underline{C}$H$_{Xy}$), 127.9 (s, *m*-$\underline{C}$H$_{Xy}$), 129.2 (s, $\underline{C}_{Xy}$-N/$\underline{C}_{Xy}$-Me), 130.7 (s, *p*-$\underline{C}$H$_{Dipp}$), 133.8 (s, $\underline{C}_{Dipp}$-N), 135.0 (s, $\underline{C}_{Xy}$-Me/$\underline{C}$-$_{Xy}$-N), 146.6 (s, $\underline{C}$-*i*Pr), 186.2 (N-$\underline{C}_{Xy}$), 189.3 (d, $^1J_{P,C}$ = 152 Hz, N$\underline{C}$N), 225.5 (s_{br}, $\underline{C}$O$_{ax}$), 233.7 (s_{br}, $\underline{C}$O$_{eq}$) ppm.

^{31}P{^{1}H} NMR (202.5 MHz, CD_2Cl_2, 298 K): δ = 564.2 (s) ppm.

^{31}P{^{1}H} NMR (121.5 MHz, C_6D_6, 298 K): δ = 554.5 (s) ppm.

^{1}H,^{15}N HMBC (51 MHz, CD_2Cl_2, 298 K): δ_N = −203 ppm.

IR (THF): $\tilde{\nu}$ = 2096 ν(C-N), 1965 ν(C-O), 1902 ν(C-O), 1890 ν(C-O) cm^{-1}.

UV/Vis (THF): λ_{max} = 374, 478, 642 nm.

Elemental analysis (%) calcd. for $C_{39}H_{45}MnN_3O_3P$ (689.72 g mol^{-1}): C 67.92, H 6.58, N 6.09; found: C 67.43, H 6.50, N 5.82.

S2.6. Synthesis of {(IDipp)P=PH}Mn(CO)$_4$

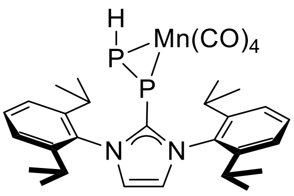

In a Schlenk flask, (IDipp)PMn(CO)$_4$ (50 mg, 0.085 mmol, 1.00 eq.) was dissolved in toluene (5 mL). To this green solution IDippPH (36 mg, 0.085 mmol, 1.00 eq.), dissolved in toluene (5 mL, yellow), was added at room temperature, resulting in a light green solution. The reaction mixture was stirred at room temperature for 16 h, followed by filtration over aluminum oxide (neutral) and the removal of the solvent under high vacuum resulting in an orange solid. The residue was dissolved in toluene (1 mL) and layered with *n*-hexane (5 mL). Storage over 48 h resulted in the formation of orange crystals. The solution was decanted with a syringe and the crystals dried under reduced pressure. {(IDipp)P=PH}Mn(CO)$_4$ was isolated as an orange solid (23 mg, 0.037 mmol, 44 %).

Single crystals suitable for x-ray diffraction analysis could be grown by vapor diffusion of *n*-hexane into a saturated solution of {(IDipp)P=PH}Mn(CO)$_4$ in C_6D_6 at room temperature.

^{1}H NMR (600 MHz, C_6D_6, 298 K): δ = –0.30 (1H, dd, J_{H,P_A} = 134 Hz, J_{H,P_X} = 26 Hz, P$\underline{H}$), 0.92 (6H, d, $^3J_{H,H}$ = 6.9 Hz, CH(C$\underline{H}_3$)$_2$), 0.93 (6H, d, $^3J_{H,H}$ = 6.8 Hz, CH(C$\underline{H}_3$)$_2$), 1.46 (6H, d, $^3J_{H,H}$ = 6.7 Hz, CH(C$\underline{H}_3$)$_2$), 1.48 (6H, d, $^3J_{H,H}$ = 6.7 Hz, CH(C$\underline{H}_3$)$_2$), 2.73–2.83 (4H, m ("sept"), C$\underline{H}$(CH$_3$)$_2$), 6.33 (2H, s, C$\underline{H}_{Imidazolin}$), 7.11 (2H, dd, $^3J_{H,H}$ = 7.7 Hz, $^4J_{H,H}$ = 1.4 Hz,

m-C*H*$_{Dipp}$), 7.18 (2H, dd, $^3J_{H,H}$ = 7.8 Hz, $^4J_{H,H}$ = 1.4 Hz, *m*-C*H*$_{Dipp}$), 7.30 (2H, t, $^3J_{H,H}$ = 7.8 Hz, *p*-C*H*$_{Dipp}$) ppm.

$^{13}C\{^1H\}$ NMR (151 MHz, C_6D_6, 298 K): δ = 22.5 (dd, $J_{C,P}$ = 4 Hz, $J_{C,P}$ = 4 Hz, CH(*C*H$_3$)$_2$), 22.8 (d, $J_{C,P}$ = 2 Hz, CH(*C*H$_3$)$_2$), 25.9 (s, CH(*C*H$_3$)$_2$), 26.2 (s$_{br}$, CH(*C*H$_3$)$_2$), 29.2 (s, *C*H(CH$_3$)$_2$), 29.5 (d, $J_{C,P}$ = 7 Hz, *C*H(CH$_3$)$_2$), 124.3 (d, $J_{C,P}$ = 4 Hz, *C*H$_{Imidazolin}$), 125.2 (s, *m*-*C*H$_{Dipp}$), 125.2 (s, *m*-*C*H$_{Dipp}$), 131.6 (s, *p*-*C*H$_{Dipp}$), 133.1 (s$_{br}$, *C*-N), 145.1 (s, *C*-*i*Pr), 171.4 (dd, $J_{P,C}$ = 133 Hz, $J_{P,C}$ = 13 Hz, N*C*N), 218.5 (s$_{br}$, *C*O), 221.7 (s$_{br}$, *C*O), 222.7 (s$_{br}$, *C*O), 225.3 (s$_{br}$, *C*O) ppm.

$^{31}P\{^1H\}$ NMR (202.5 MHz, C_6D_6, 298 K): AX spin system: δ = –85.6 (d, $^1J_{P,P}$ = 279 Hz, *P*$_A$), –208.9 (d, $^1J_{P,P}$ = 279 Hz, *P*$_X$) ppm.

^{31}P NMR (202.5 MHz, C_6D_6, 298 K): AMX spin system: δ = –85.6 (d, $^1J_{P,P}$ = 279 Hz, $J_{P_A,H}$ = 26 Hz, *P*$_A$), –208.9 (d, $^1J_{P,P}$ = 279 Hz, $J_{P_X,H}$ = 134 Hz, *P*$_X$) ppm.

$^1H,^{15}N$ HMBC (61 MHz, C_6D_6, 298 K): δ_N = −191 ppm.

IR (THF): $\tilde{\nu}$ = 2029 *ν*(C-O), 1952 *ν*(C-O), 1936 *ν*(C-O), 1913 *ν*(C-O) cm^{-1}.

UV/Vis (THF): λ_{max} = 328 nm.

Elemental analysis (%) calcd. for $C_{31}H_{37}MnN_2O_4P_2$ (618.53 g mol^{-1}): C 60.20, H 6.03, N 4.53; found: C 60.31, H 6.01, N 4.39.

S2.7. Synthesis of {(IDipp)P=PPh}Mn(CO)$_4$

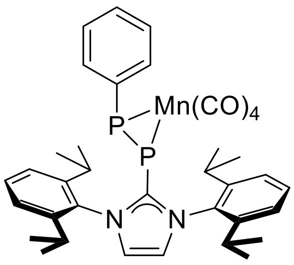

In a Schlenk flask, (IDipp)PMn(CO)$_4$ (20 mg, 0.034 mmol, 1.00 eq.) was dissolved in toluene (2 mL). To this green solution IMePPh (8 mg, 0.034 mmol, 1.00 eq.), suspended in toluene (4 mL, yellow), was added at room temperature, resulting in a dark yellow-orange solution. After 2 h, the reaction mixture had turned light yellow. Stirring was continued at room temperature for 16 h, followed by filtration over aluminum oxide (neutral) and the removal of the solvent under high vacuum resulting in a yellow solid (15 mg, 0.022 mmol, 63 %)

Single crystals suitable for x-ray diffraction analysis could be grown by vapor diffusion of *n*-hexane into a saturated solution of {(IDipp)P=PPh}Mn(CO)$_4$ in C_6D_6 at room temperature.

^1H NMR (600 MHz, C_6D_6, 298 K): δ = 0.82 (6H, d, $^3J_{H,H}$ = 7.2 Hz, CH(C*H*$_3$)$_2$), 0.84 (6H, d, $^3J_{H,H}$ = 7.0 Hz, CH(C*H*$_3$)$_2$), 1.43 (6H, d, $^3J_{H,H}$ = 6.7 Hz, CH(C*H*$_3$)$_2$), 1.47 (6H, d, $^3J_{H,H}$ = 6.7 Hz, CH(C*H*$_3$)$_2$), 2.66–2.75 (2H, m (“sept”), 2.77–2.86 (2H, m (“sept”), C*H*(CH$_3$)$_2$), 6.26 (2H, s, C*H*$_{Imidazolin}$), 6.70–6.73 (1H, m (“tt”), *p*-C*H*$_{Ph}$), 6.77–6.81 (2H, m (“t”), *m*-C*H*$_{Ph}$), 7.00 (2H, dd, $^3J_{H,H}$ = 7.7 Hz, $^4J_{H,H}$ = 1.4 Hz, *m*-C*H*$_{Dipp}$), 7.05 (2H, dd, $^3J_{H,H}$ = 7.8 Hz, $^4J_{H,H}$ = 1.4 Hz, *m*-C*H*$_{Dipp}$), 7.11 (2H, t, $^3J_{H,H}$ = 7.7 Hz, *p*-C*H*$_{Dipp}$, 7.32–7.36 (2H, m (“t”), *o*-C*H*$_{Ph}$) ppm.

^{13}**C{^{1}H} NMR** (151 MHz, C_6D_6, 298 K): δ = 22.3–22.5 (m, ("t$_{br}$"), CH(C̲H$_3$)$_2$), 22.7 (s, CH(C̲H$_3$)$_2$), 26.0 (s, CH(C̲H$_3$)$_2$), 26.4 (s, CH(C̲H$_3$)$_2$), 29.2 (s, C̲H(CH$_3$)$_2$), 29.6 (d, $J_{C,P}$ = 6 Hz, C̲H(CH$_3$)$_2$), 124.3 (d, $J_{C,P}$ = 3 Hz, C̲H$_{Imidazolin}$), 125.1 (s, *m*-C̲H$_{Dipp}$), 125.2 (s, *m*-C̲H$_{Dipp}$), 126.1(s, *p*-C̲H$_{Ph}$), 127.5 (d, $J_{C,P}$ = 2 Hz, *m*-C̲H$_{Ph}$), 131.6 (s, *p*-C̲H$_{Dipp}$), 133.1–133.4 (m, C̲-N, *o*-C̲H$_{Ph}$), 144.7 (s, C̲-*i*Pr), 146.5 (s, C̲-*i*Pr), 146.6 (dd, $J_{P,C}$ = 61 Hz, $J_{P,C}$ = 11 Hz, PC̲), 170.8 (dd, $J_{P,C}$ = 131 Hz, $J_{P,C}$ = 11 Hz, NC̲N), 216.2 (s$_{br}$, C̲O), 220.2 (s$_{br}$, C̲O), 221.7 (s$_{br}$, C̲O), 225.6 (s$_{br}$, C̲O) ppm.

^{31}**P{^{1}H} NMR** (202.5 MHz, C_6D_6, 298 K): AX spin system: δ = –84.8 (d, $^{1}J_{P,P}$ = 341 Hz, P̲$_A$), –98.2 (d, $^{1}J_{P,P}$ = 341 Hz, P̲$_X$) ppm.

^{31}**P NMR** (202.5 MHz, C_6D_6, 298 K): AMX spin system: δ = –84.8 (d, $^{1}J_{P,P}$ = 341 Hz, P̲$_A$), –98.2 (d, $^{1}J_{P,P}$ = 341 Hz, P̲$_X$)[4] ppm.

^{1}**H,**^{15}**N HMBC** (61 MHz, C_6D_6, 298 K): δ_N = −192 ppm.

IR (THF): $\tilde{\nu}$ = 2031 *ν*(C-O), 1954 *ν*(C-O), 1938 *ν*(C-O), 1911 *ν*(C-O) cm^{-1}.

UV/Vis (THF): λ_{max} = 294 nm.

Elemental analysis (%) calcd. for $C_{37}H_{41}MnN_2O_4P_2$ (694.63 g mol^{-1}): C 63.98, H 5.95, N 4.03; found: C 63.85, H 5.95, N 3.92.

S2.8. Synthesis of {(IDipp)P=Se}Mn(CO)$_4$

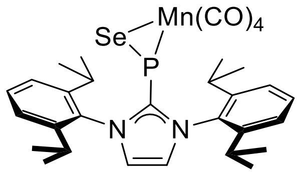

(IDipp)PMn(CO)$_4$ (50 mg, 0.085 mmol, 1.0 eq.) was dissolved in THF (3 mL) resulting in a dark green solution. Solid grey selenium (7 mg, 0.085 mmol, 1.0 eq.) was added suspended in THF (3 mL) and added in one portion to the stirred solution at room temperature. The reaction mixture was stirred for 3 h at rt. After the color had changed from dark green to red, the solvent was removed under reduced pressure and the residue extracted with toluene (3 mL). The extract was filtered through a small piece of tissue. After removal of the solvent and drying under reduced pressure {(IDipp)P=Se}Mn(CO)$_4$ could be obtained as an orange solid (35 mg, 0.053 mmol, 62%).

Single crystals suitable for x-ray diffraction analysis could be grown by vapor diffusion of *n*-hexane into a solution of {(IDipp)P=Se}Mn(CO)$_4$ in C_6D_6 at room temperature.

^{1}**H NMR** (400 MHz, THF-d$_8$, 298 K): δ = 1.01 (6H, d, $^{3}J_{H,H}$ = 7.0 Hz, CH(C*H̲*$_3$)$_2$), 1.07 (6H, d, $^{3}J_{H,H}$ = 7.0 Hz, CH(C*H̲*$_3$)$_2$), 1.29 (6H, d, $^{3}J_{H,H}$ = 6.5 Hz, CH(C*H̲*$_3$)$_2$), 1.39 (6H, d, $^{3}J_{H,H}$ = 6.8 Hz, CH(C*H̲*$_3$)$_2$), 2.64–2.80 (4H, m, C*H̲*(CH$_3$)$_2$), 7.26 (4H, d, $^{3}J_{H,H}$ = 7.7 Hz, *m*-C*H̲*$_{Dipp}$), 7.42 (4H, d, $^{3}J_{H,H}$ = 7.6 Hz, *m*-C*H̲*$_{Dipp}$), 7.56 (2H, t, $^{3}J_{H,H}$ = 7.7 Hz, *p*-C*H̲*$_{Dipp}$), 7.53 (2H, s, C*H̲*$_{Imidazolin}$) ppm.

[4] Slight broadening of this signal is observed. Small "shoulders" could be interpreted as a triplet (coupling to the *o*-CH groups of the P-bound phenyl ring).

^{13}C{^{1}H} NMR (101 MHz, THF-d$_8$, 298 K): δ = 22.5 (d, $J_{P,C}$ = 1 Hz, CH($\underline{C}$H$_3$)$_2$), 22.9 (d, $J_{P,C}$ = 4 Hz, CH($\underline{C}$H$_3$)$_2$), 25.7 (s, CH($\underline{C}$H$_3$)$_2$), 26.1 (s, CH($\underline{C}$H$_3$)$_2$), 29.7 (s, $\underline{C}$H(CH$_3$)$_2$), 29.9 (s, $\underline{C}$H(CH$_3$)$_2$), 30.0 (s, $\underline{C}$H(CH$_3$)$_2$), 125.2 (s, *m*-$\underline{C}$H$_{Dipp}$), 125.4 (s, *m*-$\underline{C}$H$_{Dipp}$), 126.4 (s, $\underline{C}$H$_{Imidazolin}$), 126.5 (s, $\underline{C}$H$_{Imidazolin}$), 131.7 (s, *p*-$\underline{C}$H$_{Dipp}$), 133.7 (s, $\underline{C}$-N), 145.6 (s, $\underline{C}$-*i*Pr), 146.8 (s, $\underline{C}$-*i*Pr), 169.0 (d, $^{1}J_{P,C}$ = 169 Hz, N$\underline{C}$N), 214.4 (s$_{br}$, $\underline{C}$O), 217.5 (s$_{br}$, $\underline{C}$O), 221.3 (s$_{br}$, $\underline{C}$O), 224.8 (s$_{br}$, $\underline{C}$O) ppm.

^{31}P{^{1}H} NMR (162 MHz, THF-d$_8$, 298 K): δ = –27.4 (s; satellites: d, $^{1}J_{P,Se}$ = 360 Hz; d, $^{1}J_{P,C}$ = 169 Hz) ppm.

^{77}Se NMR (76 MHz, THF-d$_8$, 298 K): δ = –648 (d, $^{1}J_{Se,P}$ = 359 Hz) ppm.

IR (THF): $\tilde{\nu}$ = 2042 ν(C-O), 1956 ν(C-O), 1917 ν(C-O) cm^{-1}.

UV/Vis (THF): λ_{max} = 297, 447 nm.

Elemental analysis (%) calcd. for $C_{31}H_{36}MnN_2O_4PSe$ (665.52 g mol^{-1}): C 55.95, H 5.45, N 4.21; found: C 56.13, H 5.36, N 3.88.

S2.9. Synthesis of {(IDipp)P=Te}Mn(CO)$_4$

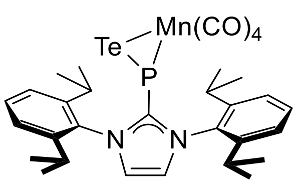

(IDipp)PMn(CO)$_4$ (50 mg, 0.085 mmol, 1.0 eq.) was dissolved in THF (5 mL) resulting in a dark green solution. Solid Tellurium (11 mg, 0.085 mmol, 1.0 eq.) was suspended in THF (5 mL) and added in one portion to the stirred solution at room temperature. The reaction mixture was stirred for 16 h at rt. After the color changed from dark green to red, the solvent was removed under reduced pressure and the residue extracted with toluene (4 mL). The extract was filtered through a small piece of tissue. After removal of the solvent and drying under reduced pressure {(IDipp)P=Te}Mn(CO)$_4$ was obtained as a red solid (51 mg, 0.071 mmol, 84%).

Single crystals suitable for x-ray diffraction analysis can be grown by vapor diffusion of *n*-hexane into a solution of {(IDipp)P=Te}Mn(CO)$_4$ in C_6D_6 at room temperature.

^{1}H NMR (400 MHz, THF-d$_8$, 298 K): δ = 1.12 (6H, d, $^{3}J_{H,H}$ = 6.8 Hz, CH(C$\underline{H}_3$)$_2$), 1.17 (6H, d, $^{3}J_{H,H}$ = 6.8 Hz, CH(C$\underline{H}_3$)$_2$), 1.40 (6H, d, $^{3}J_{H,H}$ = 6.5 Hz, CH(C$\underline{H}_3$)$_2$), 1.49 (6H, d, $^{3}J_{H,H}$ = 6.7 Hz, CH(C$\underline{H}_3$)$_2$), 2.72–2.90 (4H, m, C$\underline{H}$(CH$_3$)$_2$), 7.38 (4H, dd, $^{3}J_{H,H}$ = 7.8 Hz, $^{4}J_{H,H}$ = 1.1 Hz, *m*-C$\underline{H}_{Dipp}$), 7.42 (4H, dd, $^{3}J_{H,H}$ = 7.8 Hz, $^{4}J_{H,H}$ = 1.1 Hz, *m*-C$\underline{H}_{Dipp}$), 7.56 (2H, t, $^{3}J_{H,H}$ = 7.8 Hz, *p*-C$\underline{H}_{Dipp}$), 7.67 (2H, s, C$\underline{H}_{Imidazolin}$) ppm.

^{13}C{^{1}H} NMR (101 MHz, THF-d$_8$, 298 K): δ = 22.3 (d, $J_{P,C}$ = 4.0 Hz, CH($\underline{C}$H$_3$)$_2$), 22.8 (d, $J_{P,C}$ = 1.4 Hz, CH($\underline{C}$H$_3$)$_2$), 25.8 (s, CH($\underline{C}$H$_3$)$_2$), 26.0 (s$_{br}$, CH($\underline{C}$H$_3$)$_2$), 29.7 (s, $\underline{C}$H(CH$_3$)$_2$), 29.9 (s, $\underline{C}$H(CH$_3$)$_2$), 30.0 (s, $\underline{C}$H(CH$_3$)$_2$), 125.3 (s, *m*-$\underline{C}$H$_{Dipp}$), 125.5 (s$_{br}$, *m*-$\underline{C}$H$_{Dipp}$), 126.6 (s, $\underline{C}$H$_{Imidazolin}$), 131.8 (s, *p*-$\underline{C}$H$_{Dipp}$), 133.9 (s, $\underline{C}$-N), 146.0 (s, $\underline{C}$-*i*Pr), 147.0 (s, $\underline{C}$-*i*Pr), 167.4 (d, $^{1}J_{P,C}$ = 169 Hz, N$\underline{C}$N), 216.7 (s$_{br}$, $\underline{C}$O), 218.3 (s$_{br}$, $\underline{C}$O), 219.7 (s$_{br}$, $\underline{C}$O), 226.3 (s$_{br}$, $\underline{C}$O) ppm.

$^{31}P\{^{1}H\}$ NMR (162 MHz, THF-d$_8$, 298 K): δ = –54.0 (s; satellites: d, $^{1}J_{\mathrm{P,Te}} = 686$ Hz; d, $^{1}J_{\mathrm{P,C}} = 171$ Hz) ppm.

^{125}Te NMR (126 MHz, THF-d$_8$, 298 K): δ = –1181 (d, $^{1}J_{\mathrm{Te,P}} = 701$ Hz) ppm.

IR (THF): $\tilde{\nu}$ = 2039 *ν*(C-O), 1954 *ν*(C-O), 1919 *ν*(C-O) cm^{-1}.

UV/Vis (THF): λ_{max} = 320, 507 nm.

Elemental analysis (%) calcd. for $C_{31}H_{36}MnN_2O_4PTe$ (714.15 g mol^{-1}): C 52.14, H 5.08, N 3.92; found: C 52.23, H 5.13, N 3.73.

S3. NMR Spectra

S3.1. (IDipp)PMn(CO)4

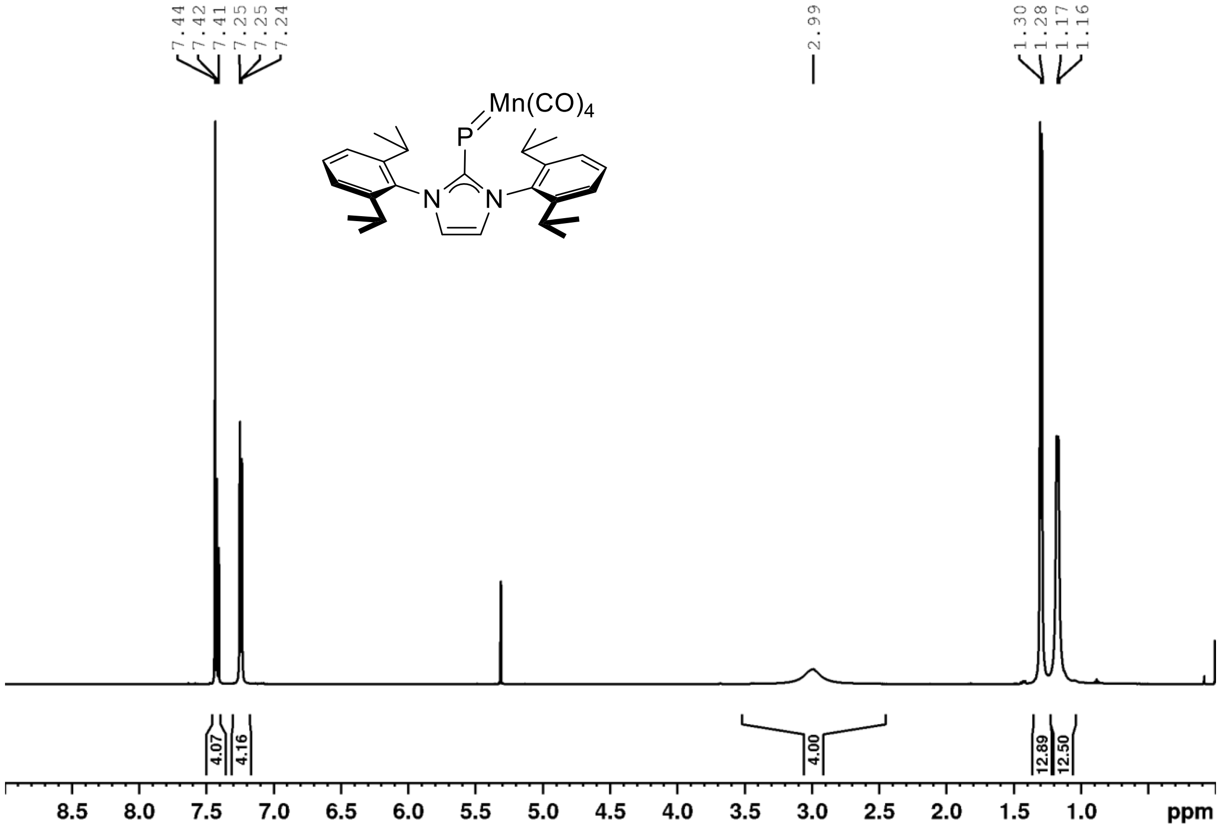

Figure S1: ^{1}H NMR spectrum of (IDipp)PMn(CO)$_4$ (500 MHz, CD_2Cl_2, 298 K, overview).

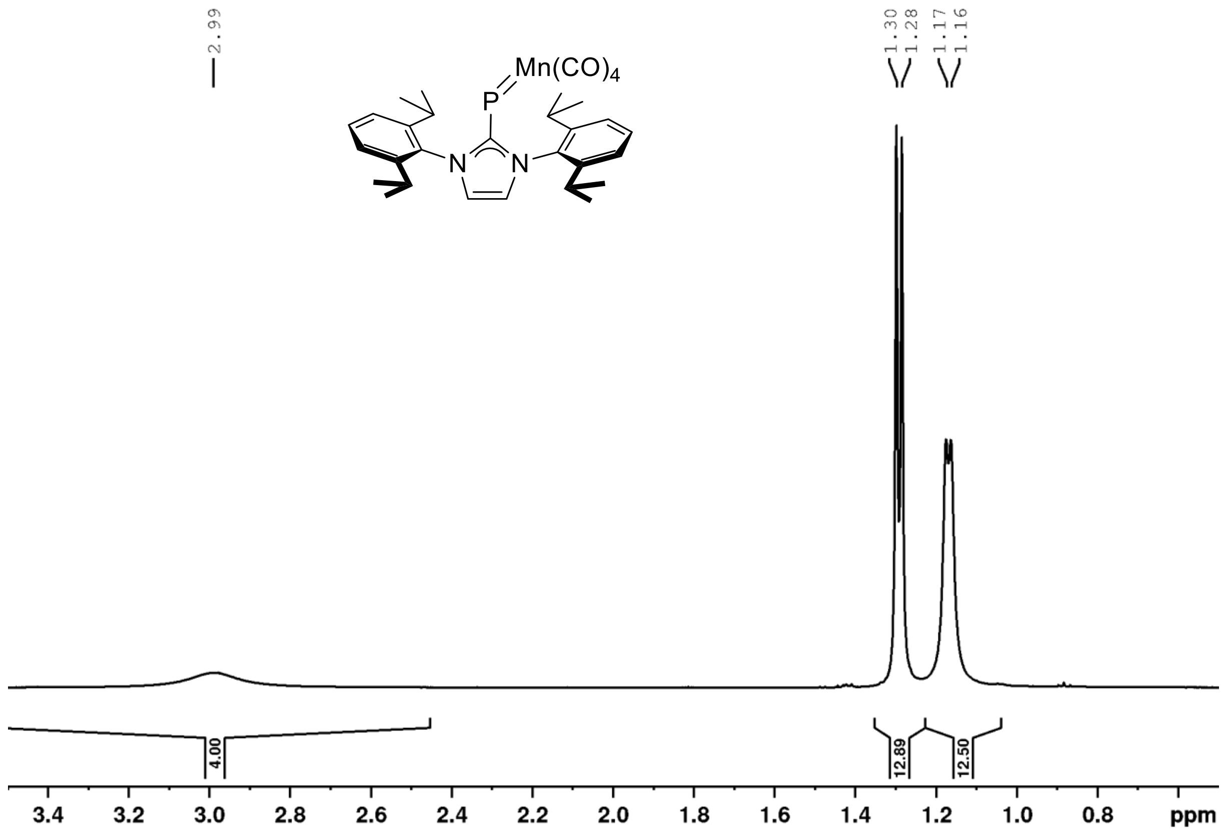

Figure S2: ^{1}H NMR spectrum of (IDipp)PMn(CO)4 (500 MHz, CD_2Cl_2, 298 K, 0.5–3.5 ppm).

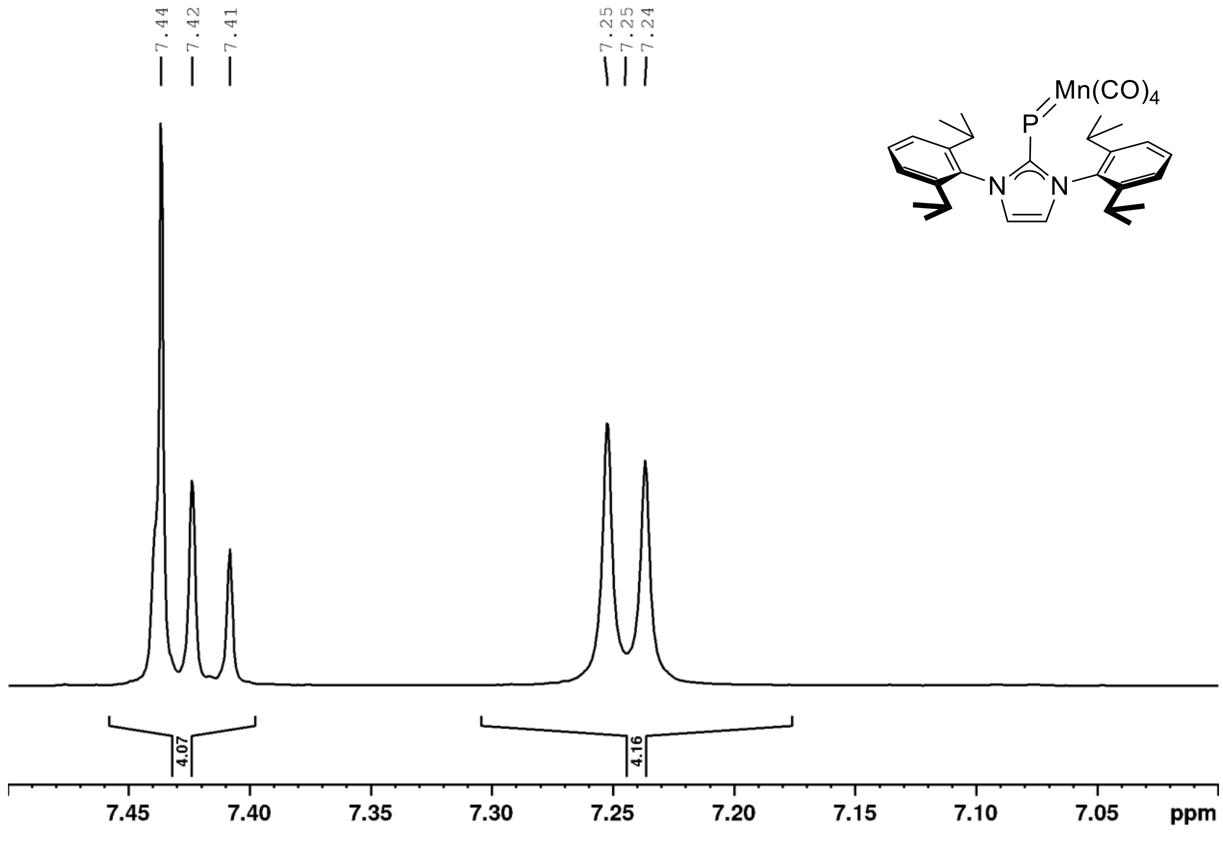

Figure S3: ^{1}H NMR spectrum of (IDipp)PMn(CO)$_4$ (400 MHz, CD_2Cl_2, 298 K, 7.0–7.5 ppm).

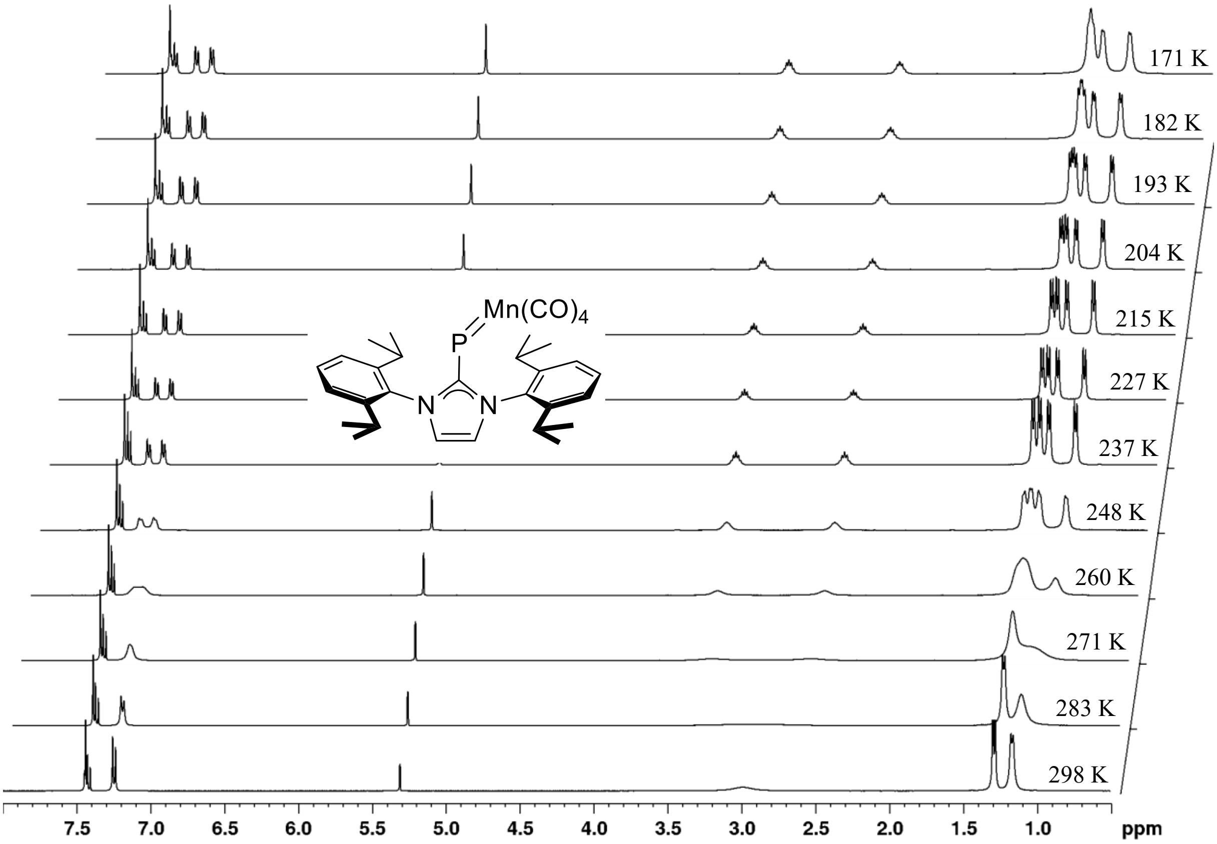

Figure S4: ^{1}H NMR spectrum of (IDipp)PMn(CO)$_4$ (400 MHz, CD_2Cl_2, 298–171 K, overview).

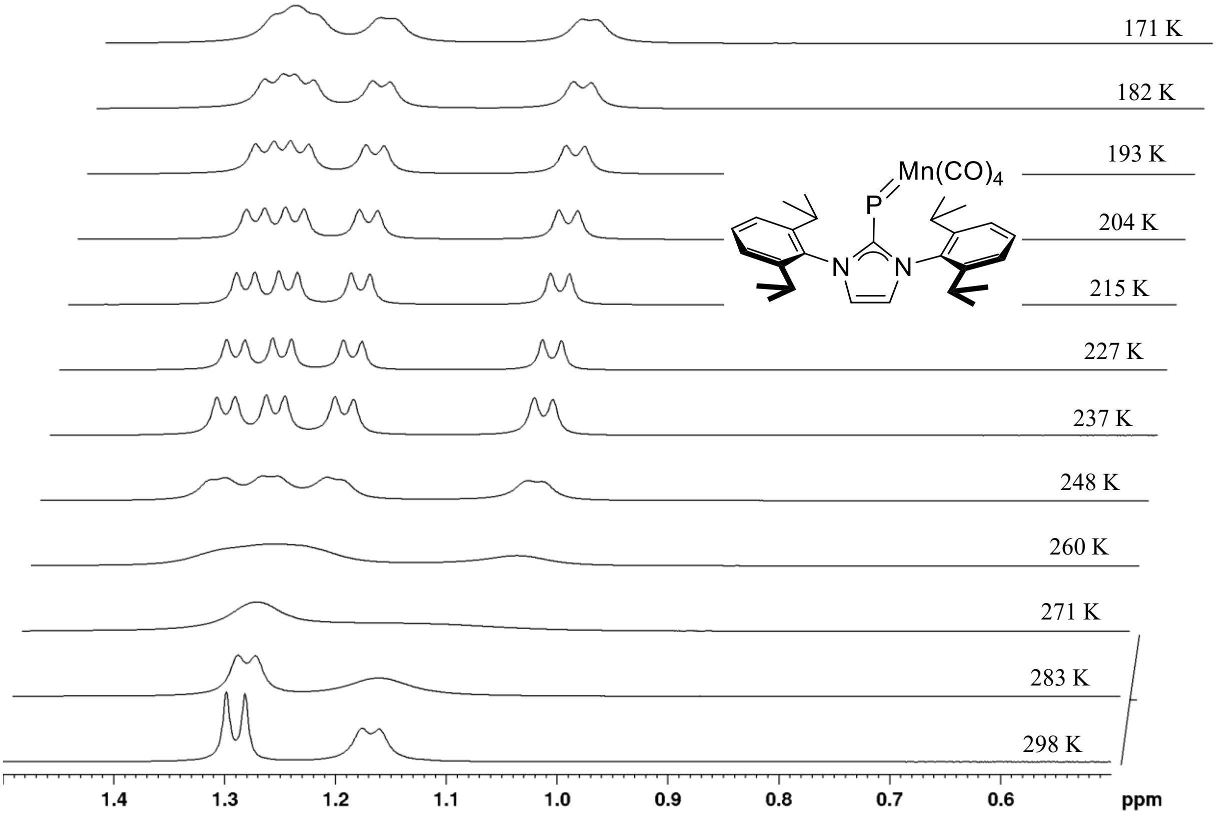

Figure S5: ^{1}H NMR spectrum of (IDipp)PMn(CO)$_4$ (400 MHz, CD_2Cl_2, 298–171 K, 0.5–1.5 ppm).

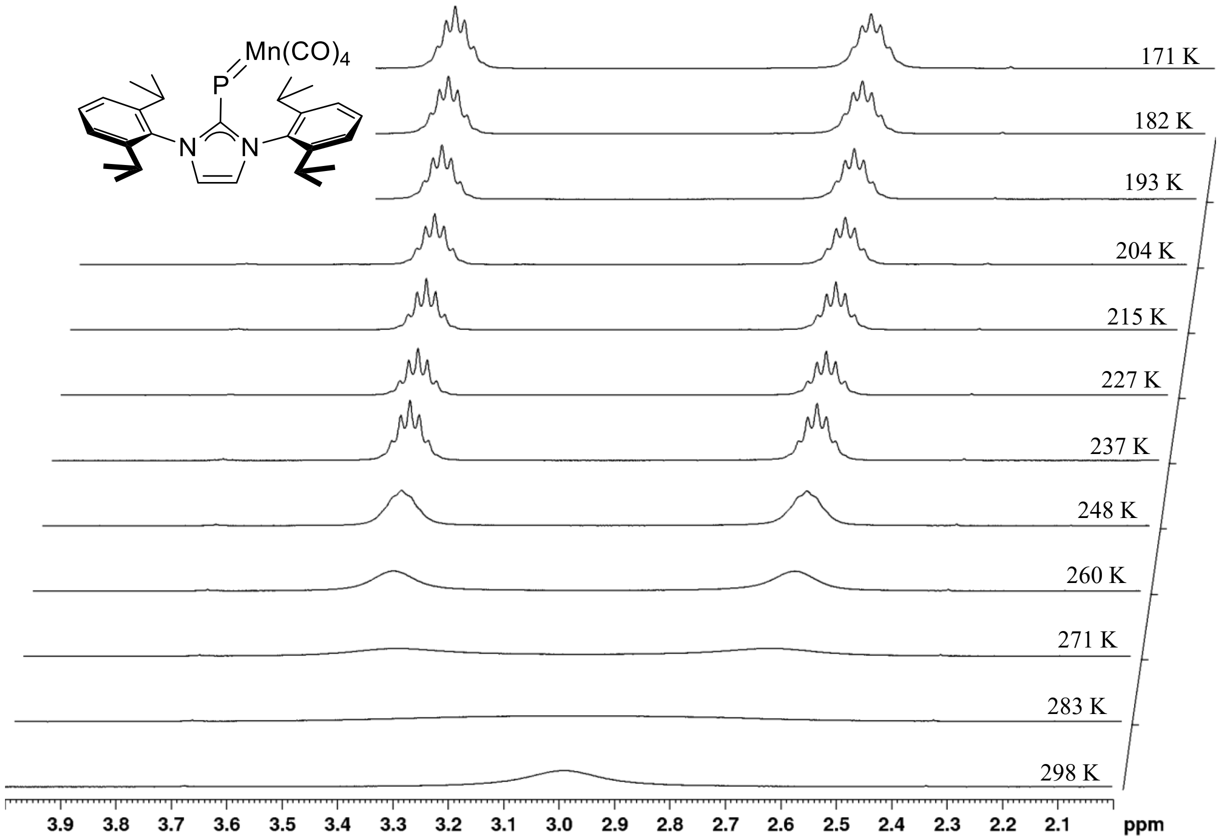

Figure S6: ^{1}H NMR spectrum of (IDipp)PMn(CO)4 (400 MHz, CD_2Cl_2, 298–171 K, 2.0–4.0 ppm).

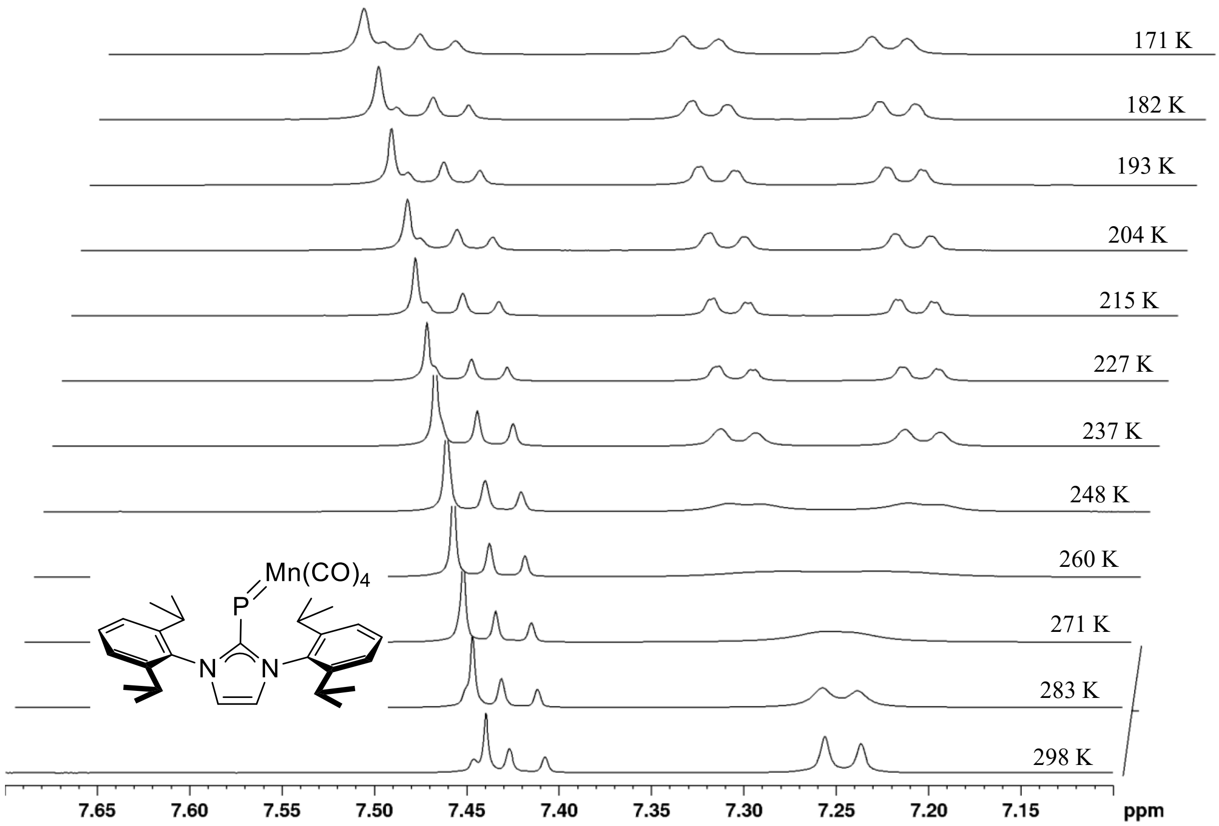

Figure S7: ^{1}H NMR spectrum of (IDipp)PMn(CO)4 (400 MHz, CD_2Cl_2, 298–171 K, 7.1–7.7 ppm).

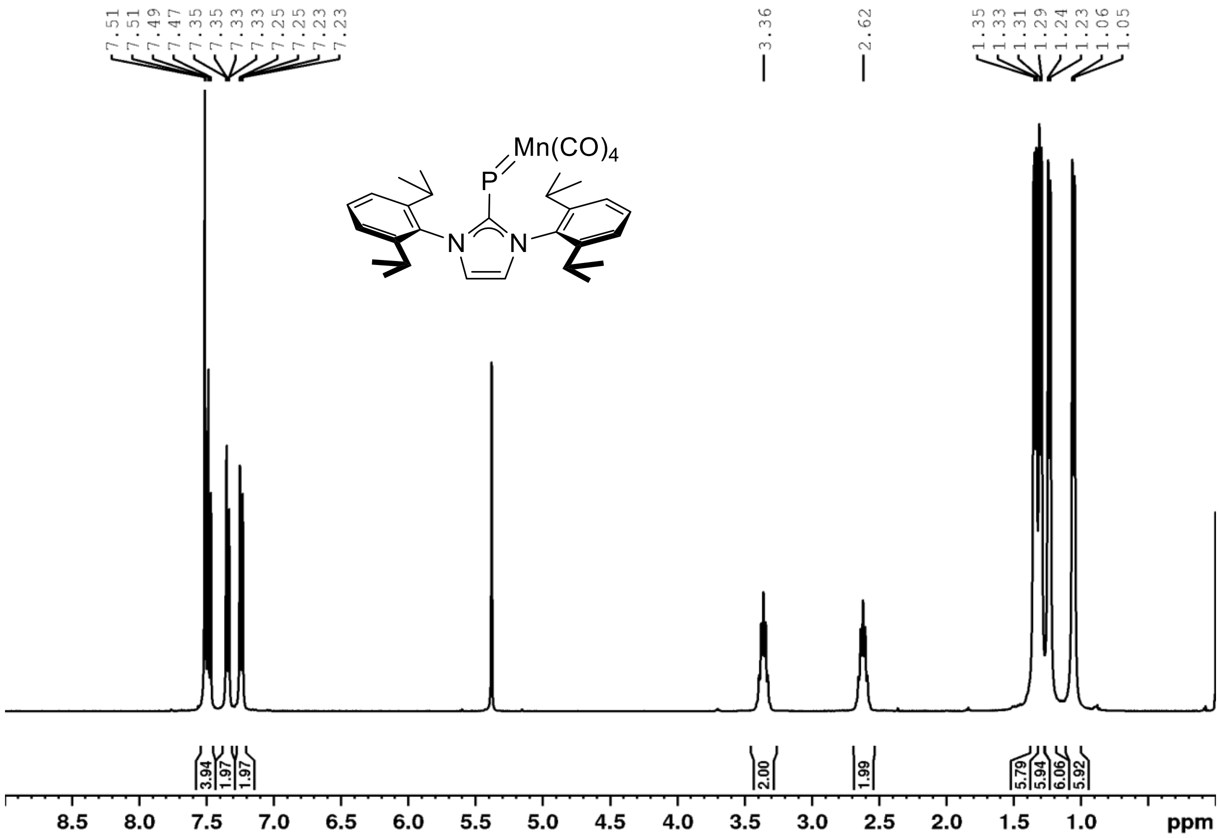

Figure S8: S9: ^{1}H NMR spectrum of (IDipp)PMn(CO)$_4$ (400 MHz, CD_2Cl_2, 215 K, overview).

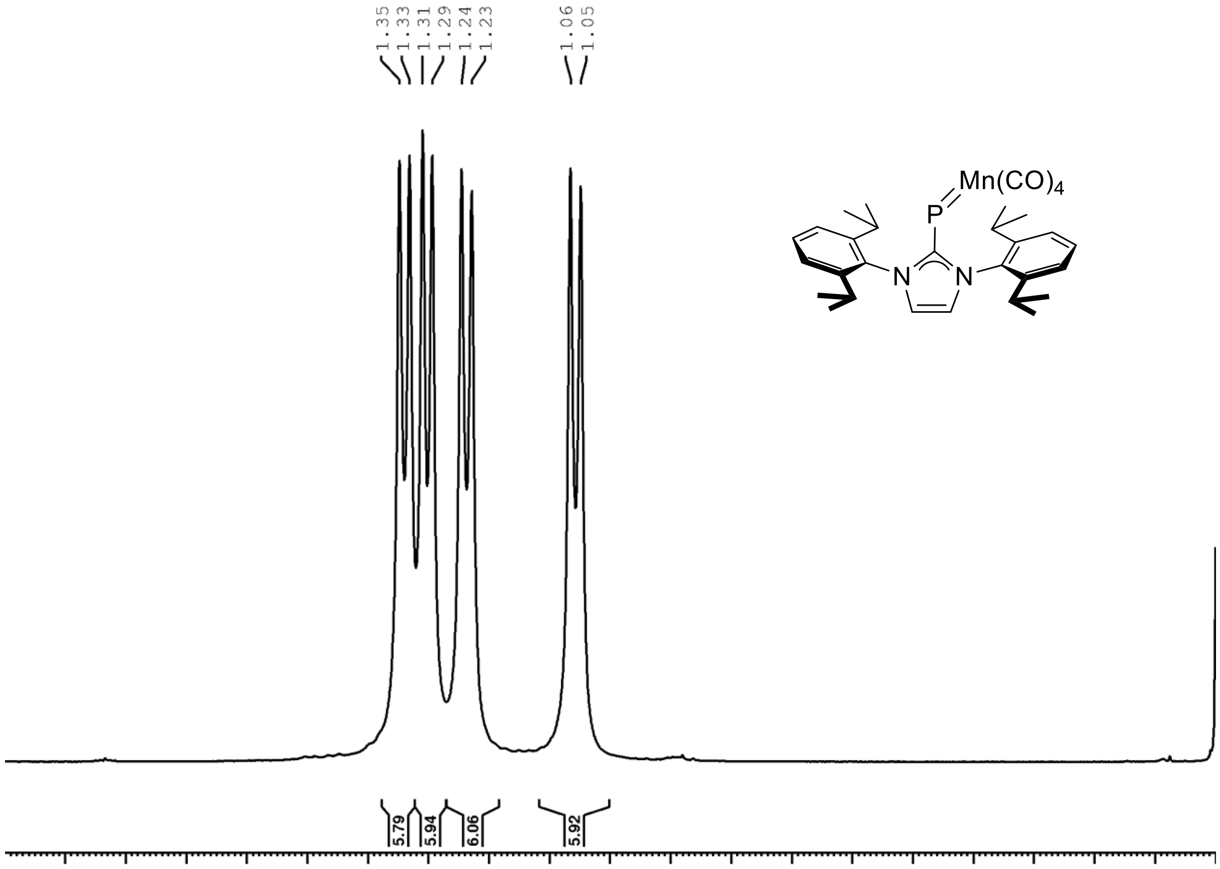

Figure S10: ^{1}H NMR spectrum of (IDipp)PMn(CO)4 (400 MHz, CD_2Cl_2, 215 K, 0.0–2.0 ppm).

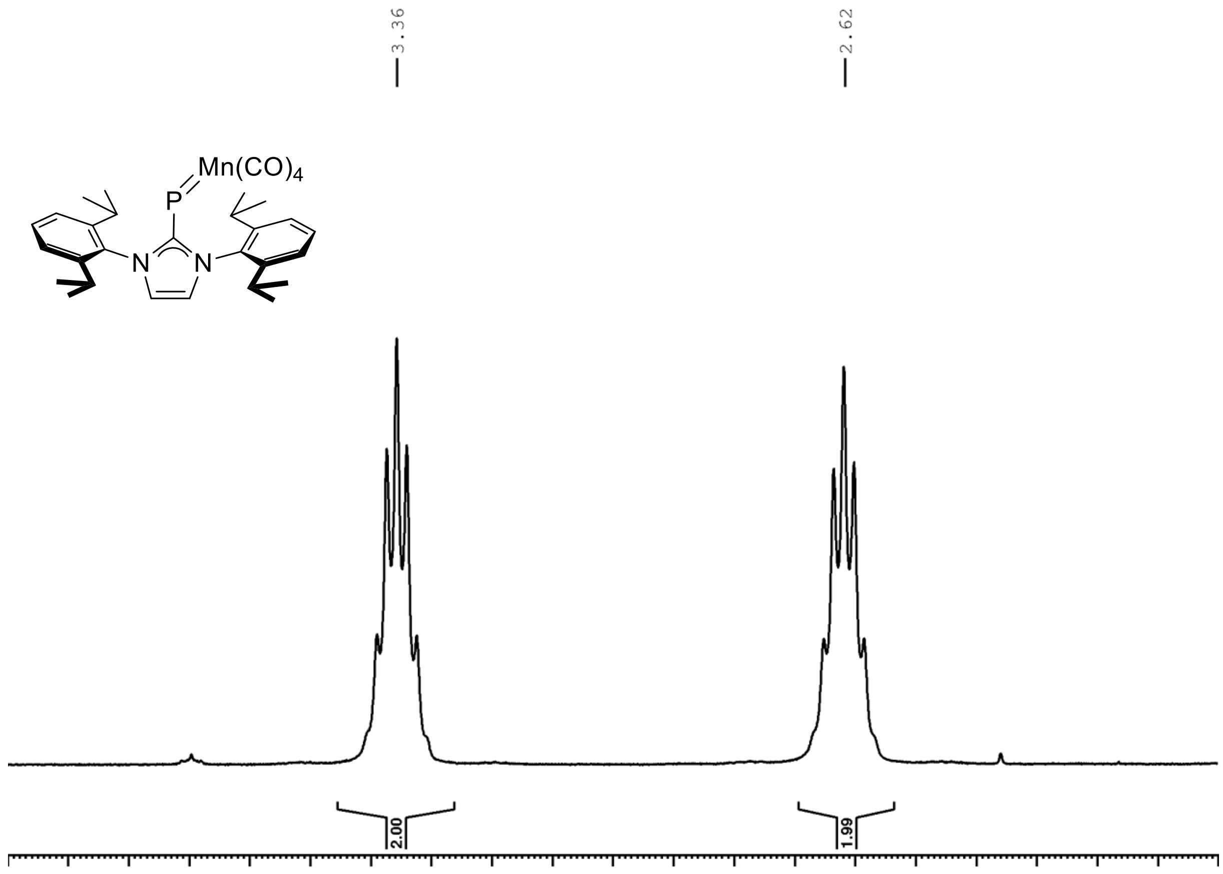

Figure S11: ^{1}H NMR spectrum of (IDipp)PMn(CO)4 (400 MHz, CD_2Cl_2, 215 K, 2.0–4.0 ppm).

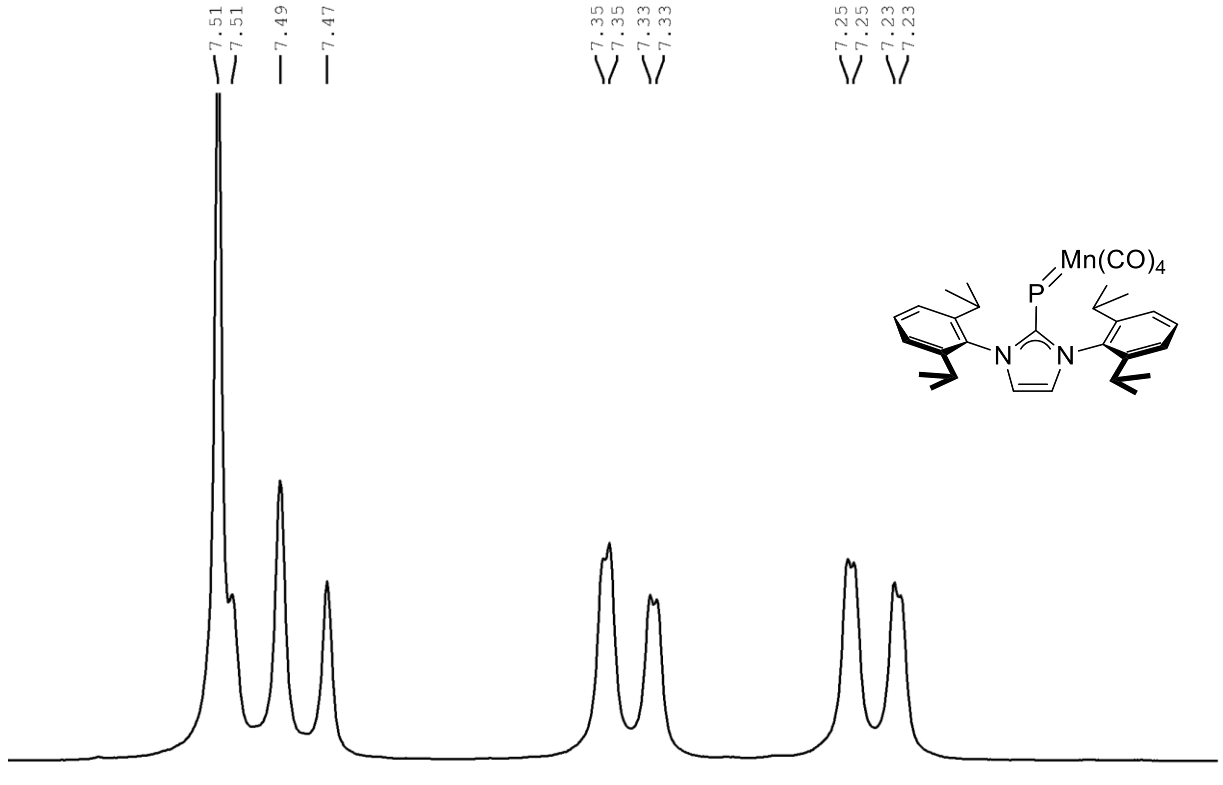

Figure S12: ^{1}H NMR spectrum of (IDipp)PMn(CO)4 (400 MHz, CD_2Cl_2, 215 K, 7.1–7.6 ppm).

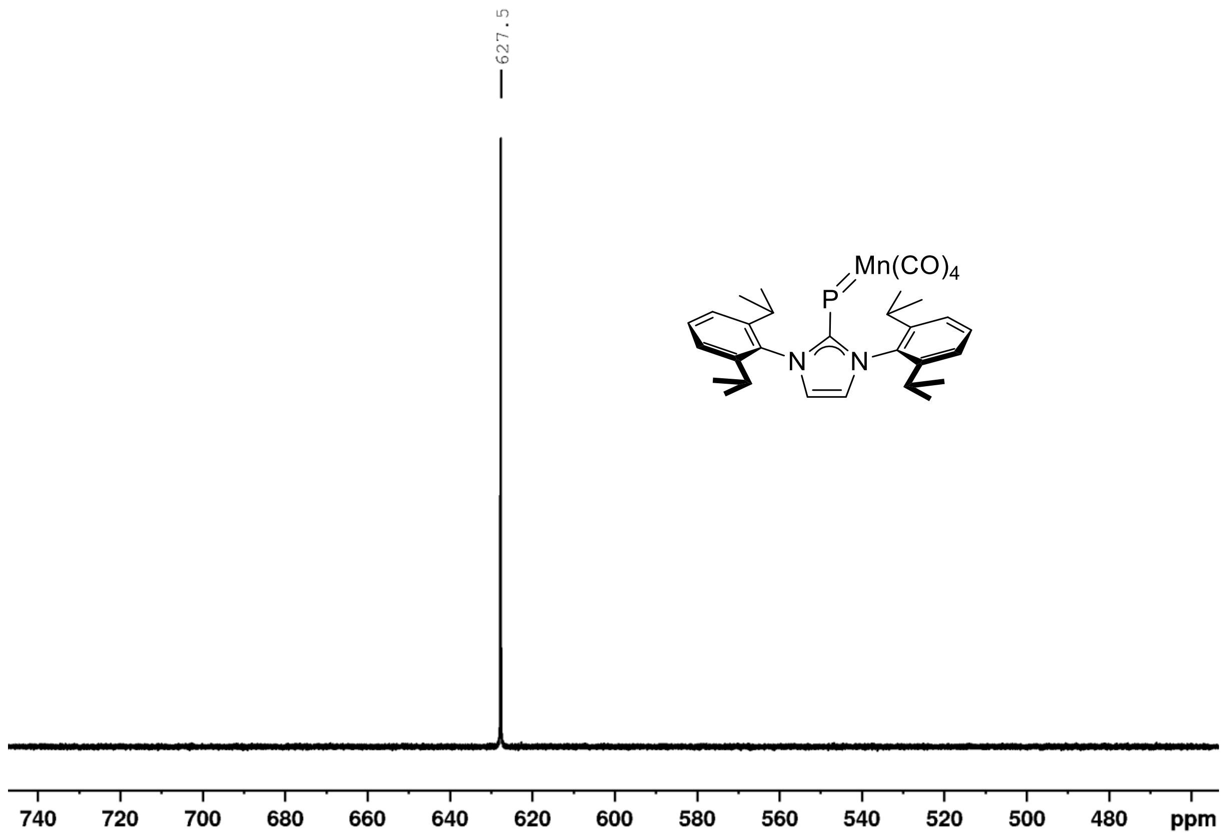

Figure S13: $^{31}P\{^1H\}$ NMR spectrum of (IDipp)PMn(CO)4 (202.5 MHz, CD_2Cl_2, 298 K).

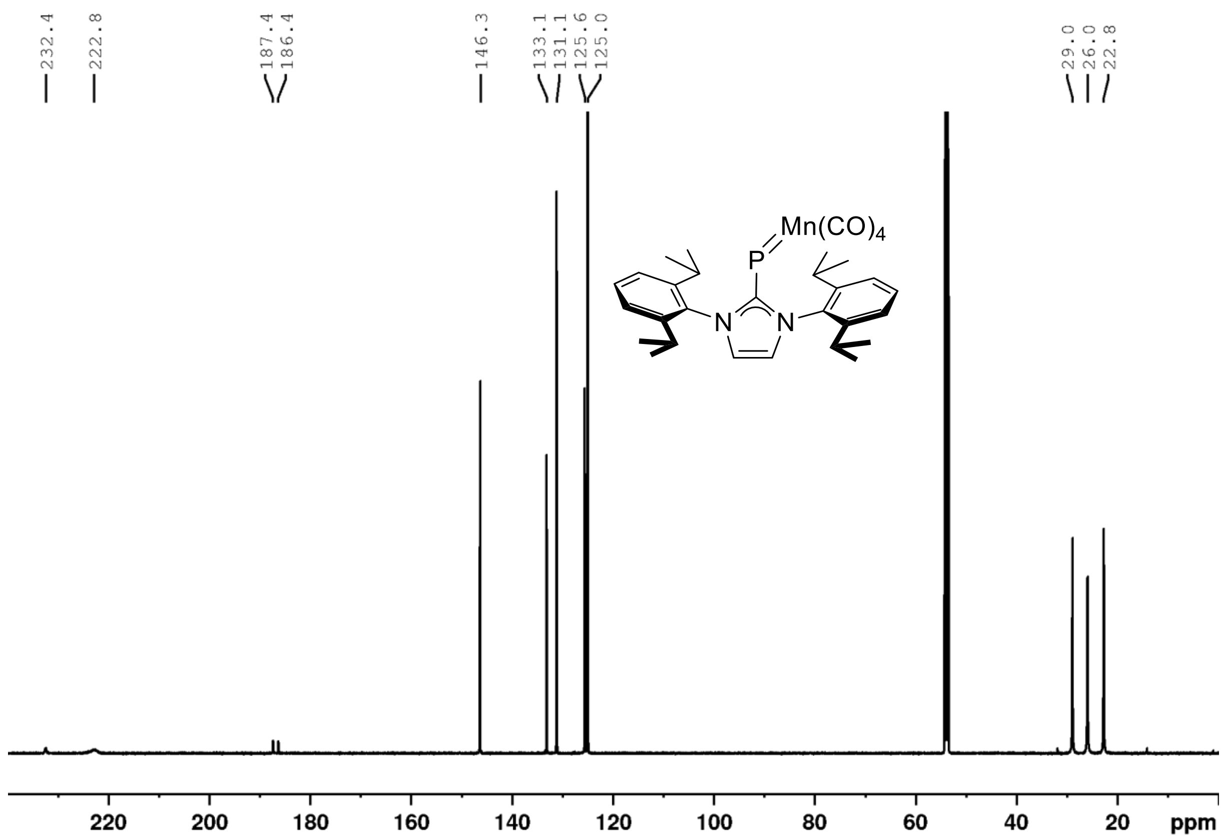

Figure S14: $^{13}C\{^1H\}$ NMR spectrum of (IDipp)PMn(CO)4 (101 MHz, CD_2Cl_2, 298 K, overview).

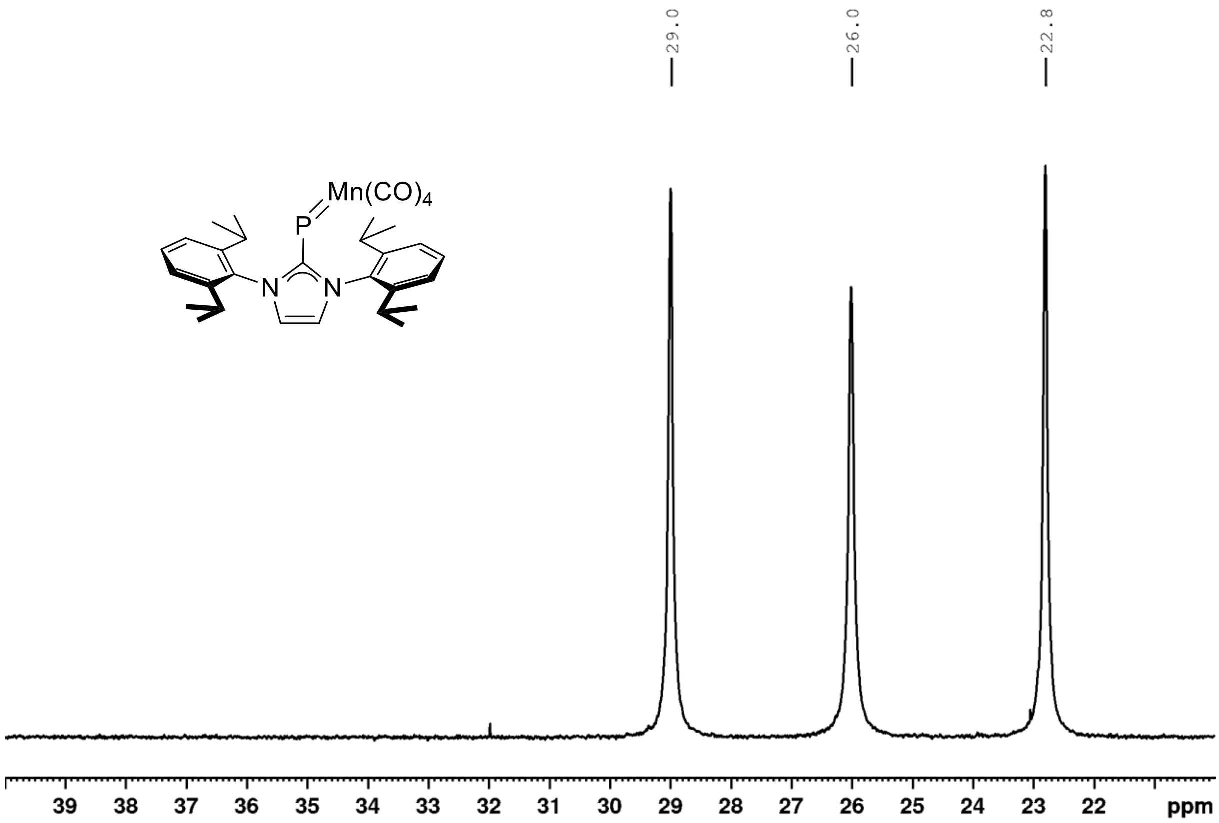

Figure S15: ${}^{13}C\{{}^{1}H\}$ NMR spectrum of (IDipp)PMn(CO)4 (101 MHz, CD_2Cl_2, 298 K, 20–40 ppm).

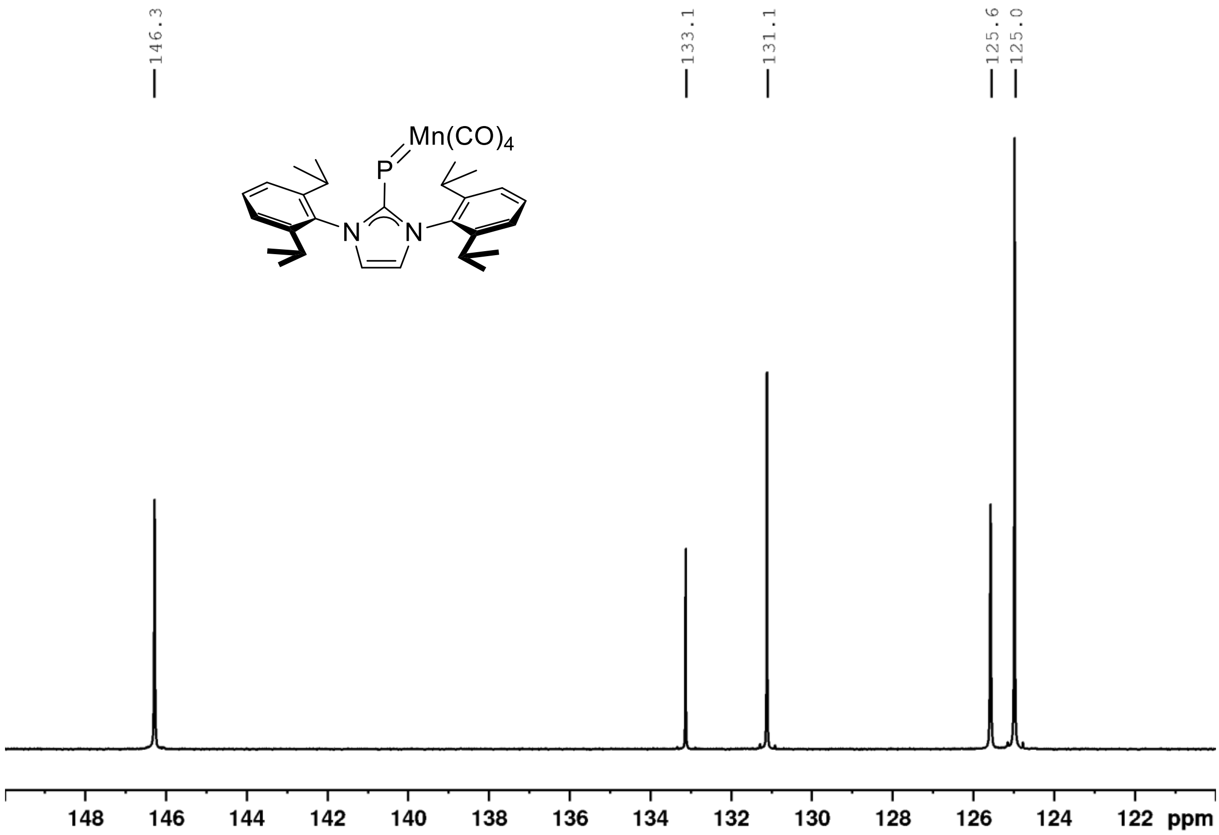

Figure S16: ${}^{13}C\{{}^{1}H\}$ NMR spectrum of (IDipp)PMn(CO)4 (101 MHz, CD_2Cl_2, 298 K, 120–150 ppm).

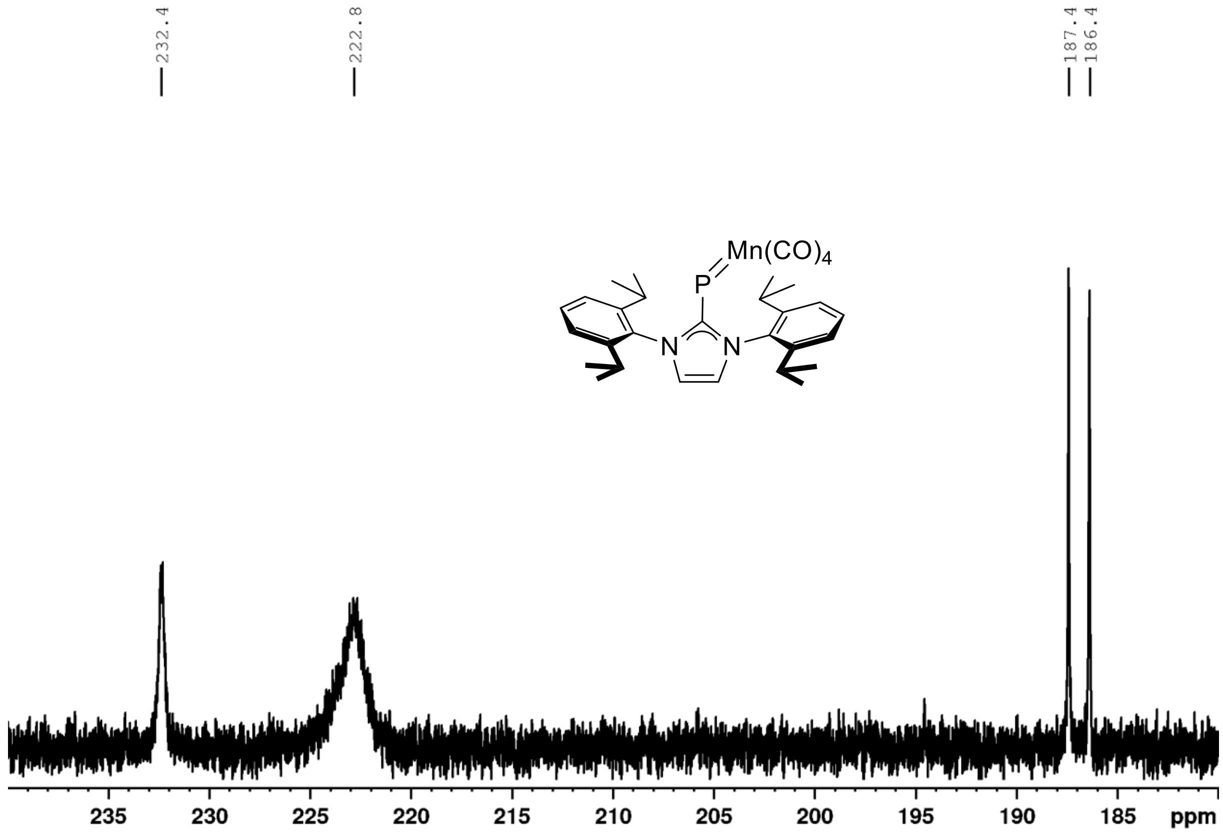

Figure S17: $^{13}C\{^1H\}$ NMR spectrum of (IDipp)PMn(CO)$_4$ (101 MHz, CD_2Cl_2, 298 K, 180–240 ppm).

S3.2. (IDipp)PMn(PPh3)(CO)3

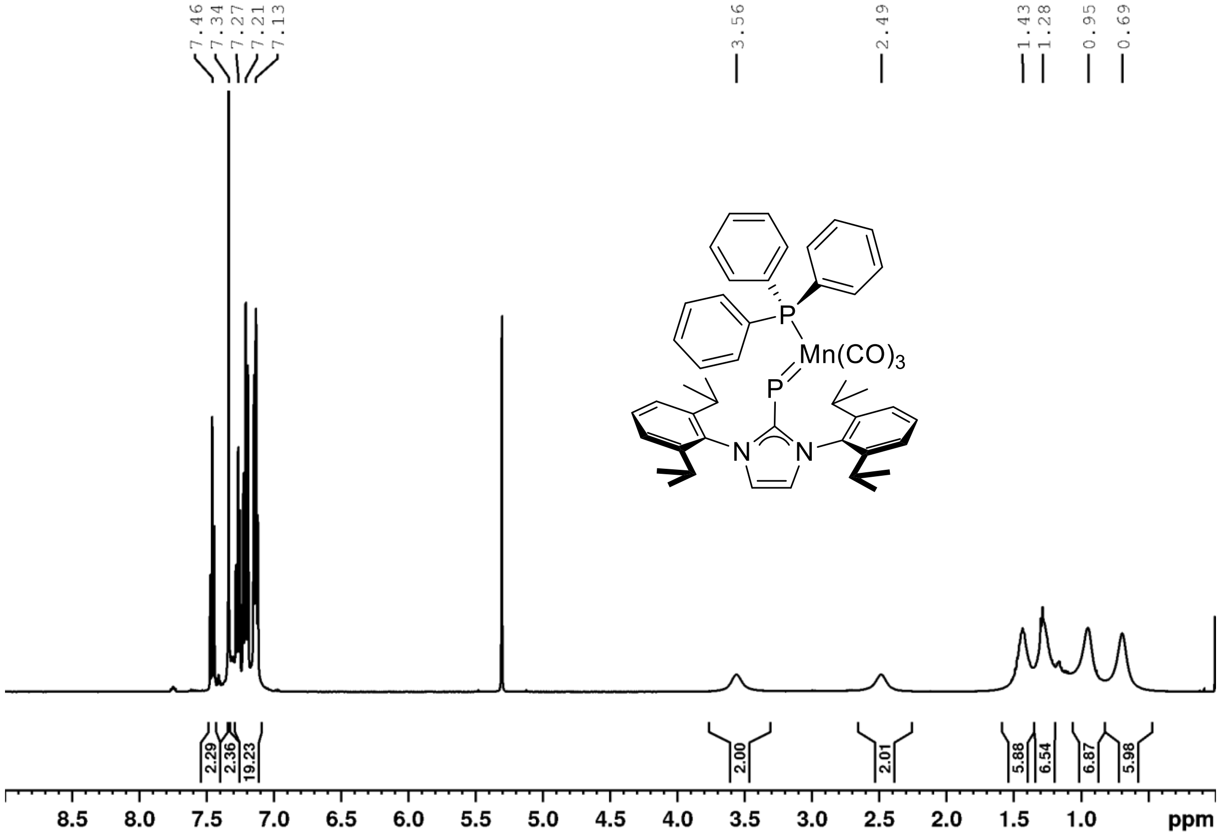

Figure S18: ^{1}H NMR spectrum of (IDipp)P(PPh3)Mn(CO)3 (500 MHz, CD_2Cl_2, 298 K, overview).

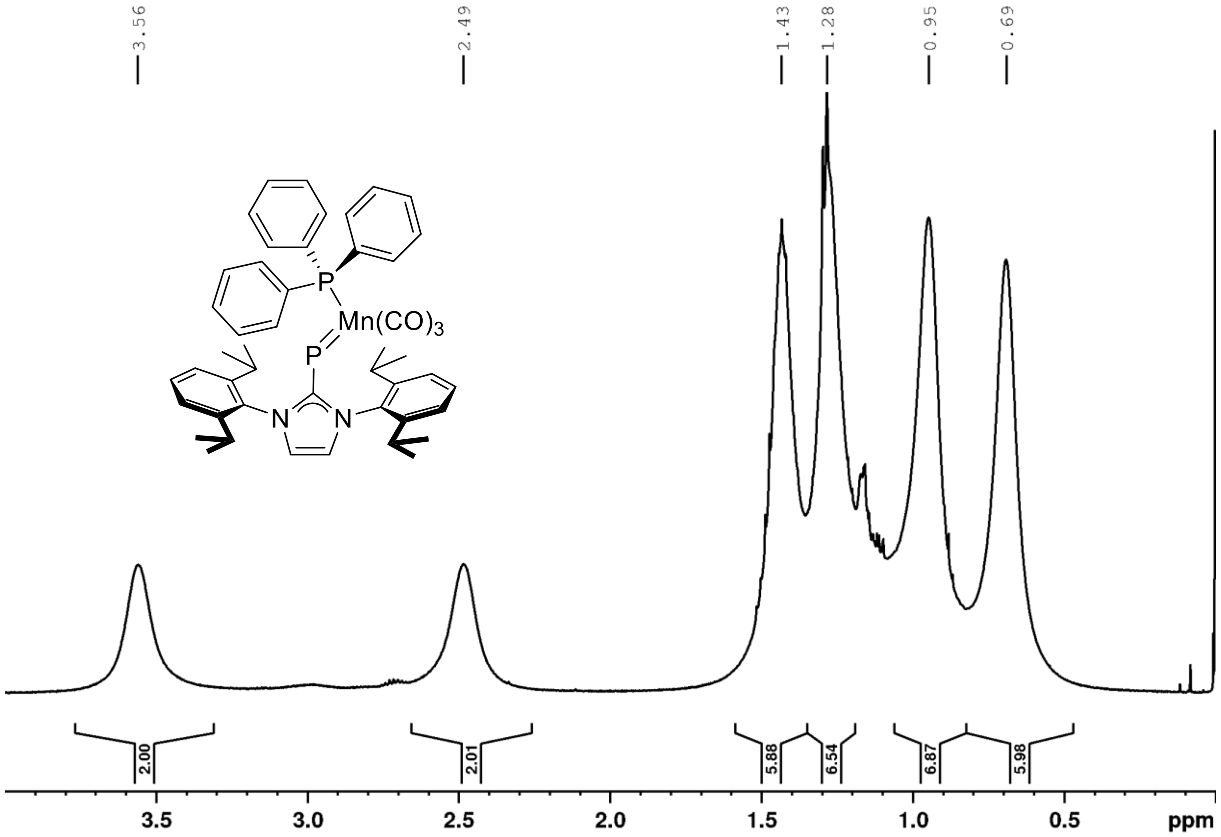

Figure S19: ^{1}H NMR spectrum of (IDipp)P(PPh3)Mn(CO)3 (500 MHz, CD_2Cl_2, 298 K, 0.0–4.0 ppm).

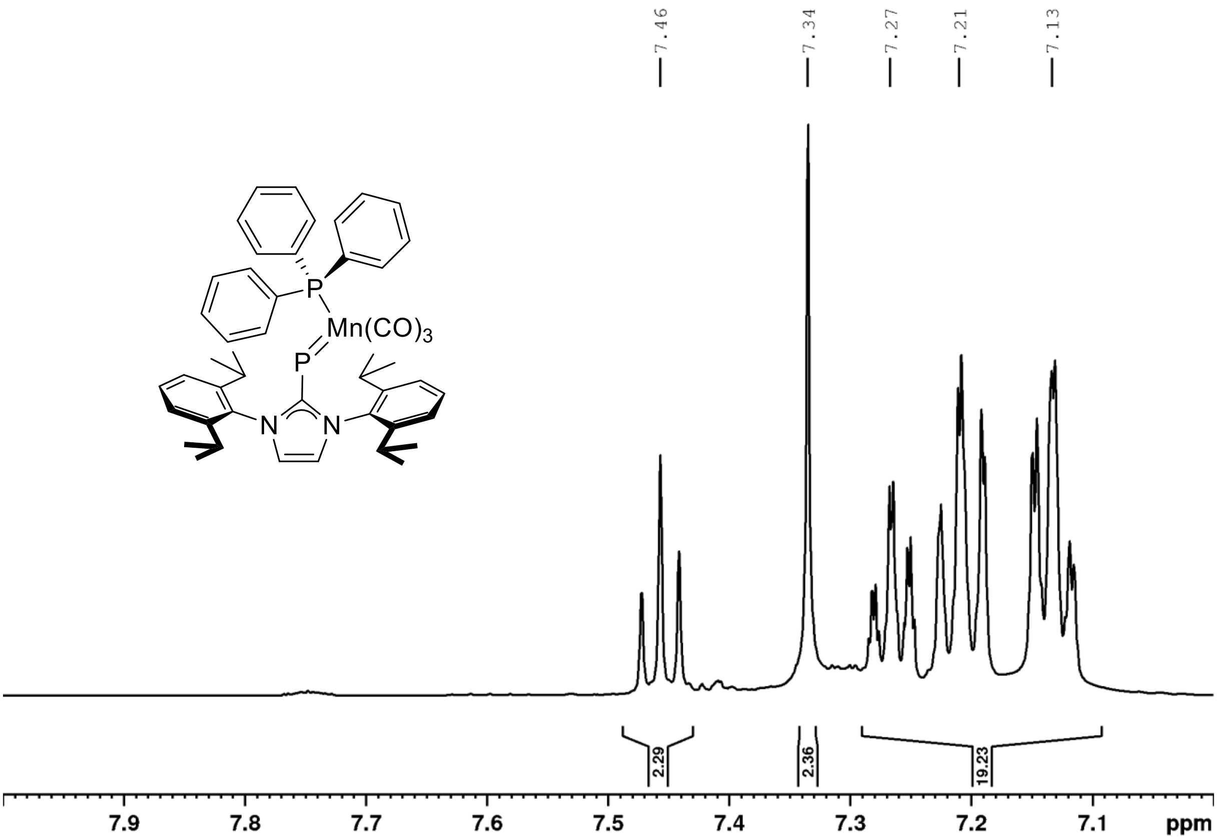

Figure S20: ^{1}H NMR spectrum of (IDipp)P(PPh3)Mn(CO)3 (500 MHz, CD_2Cl_2, 298 K, 7.0–8.0 ppm).

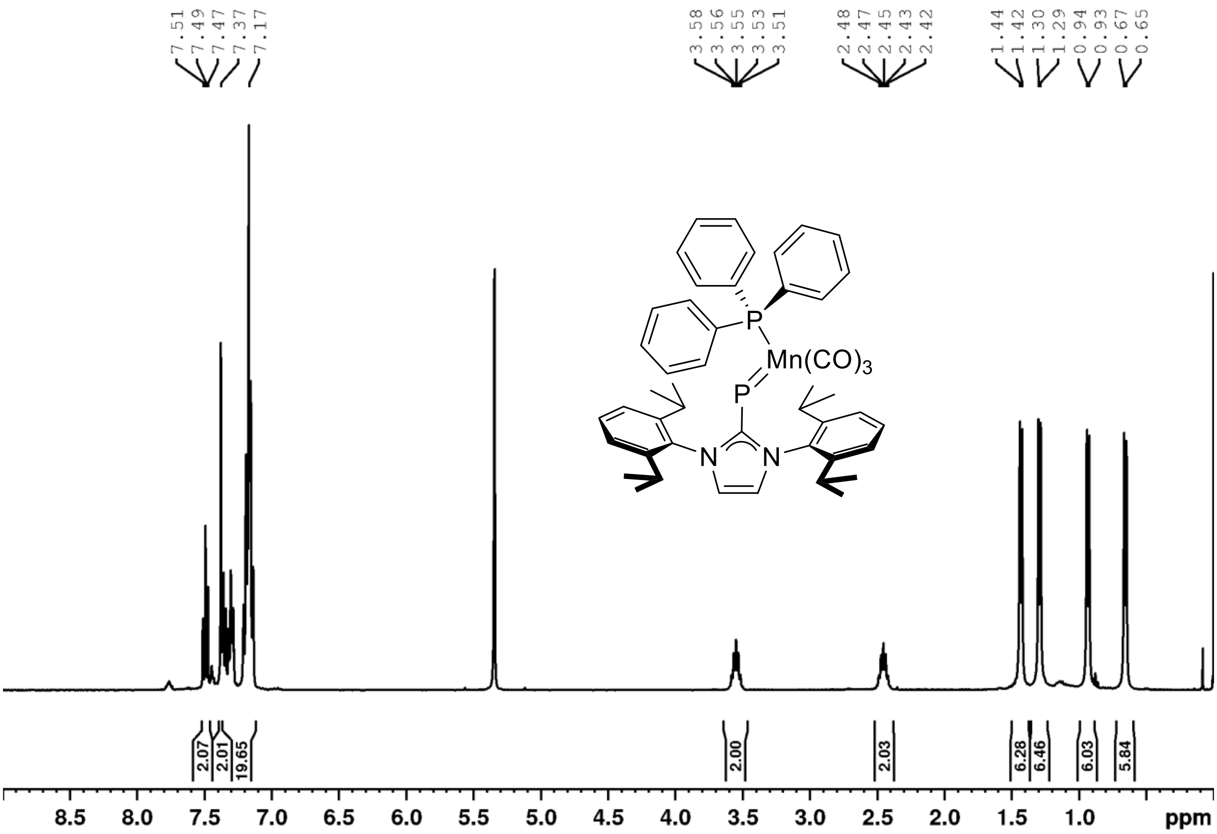

Figure S21: ^{1}H NMR spectrum of (IDipp)P(PPh3)Mn(CO)3 (400 MHz, CD_2Cl_2, 260 K, overview).

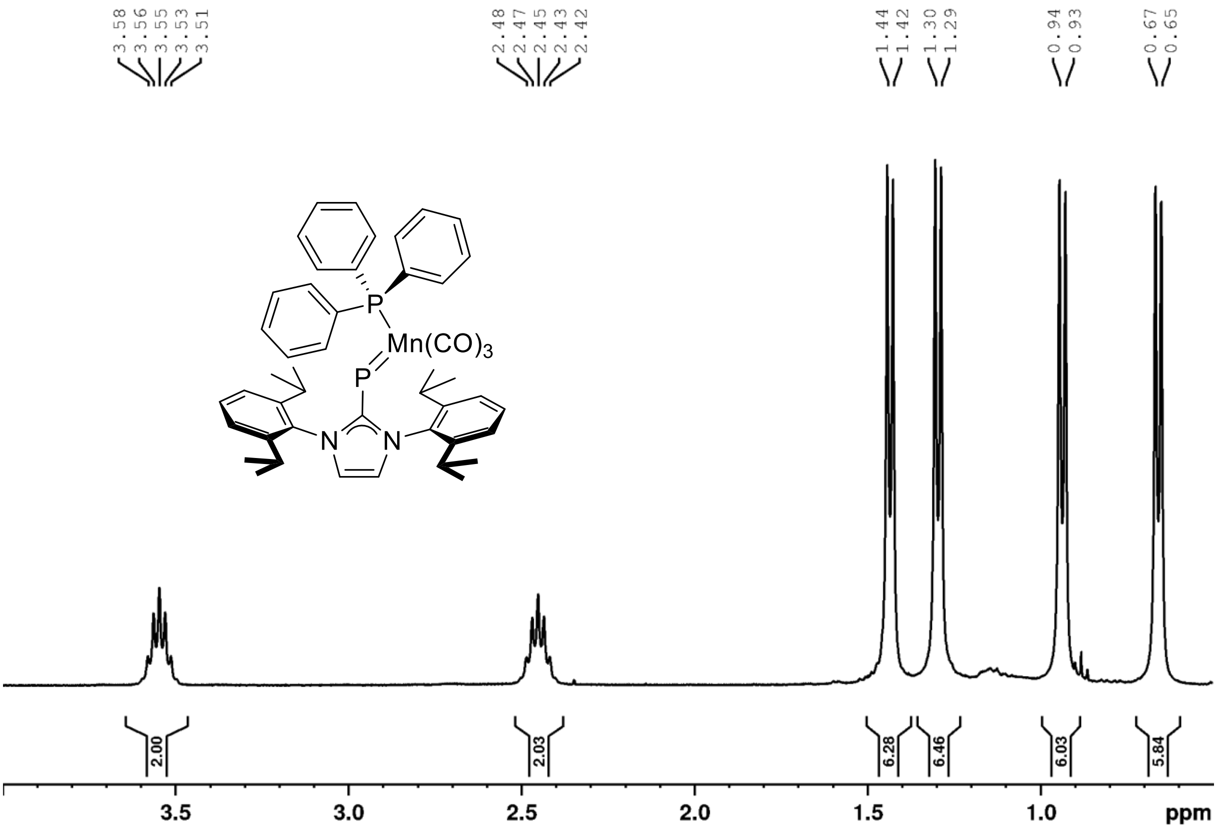

Figure S22: ^{1}H NMR spectrum of (IDipp)P(PPh$_3$)Mn(CO)$_3$ (400 MHz, CD_2Cl_2, 260 K, 0.5–4.0 ppm).

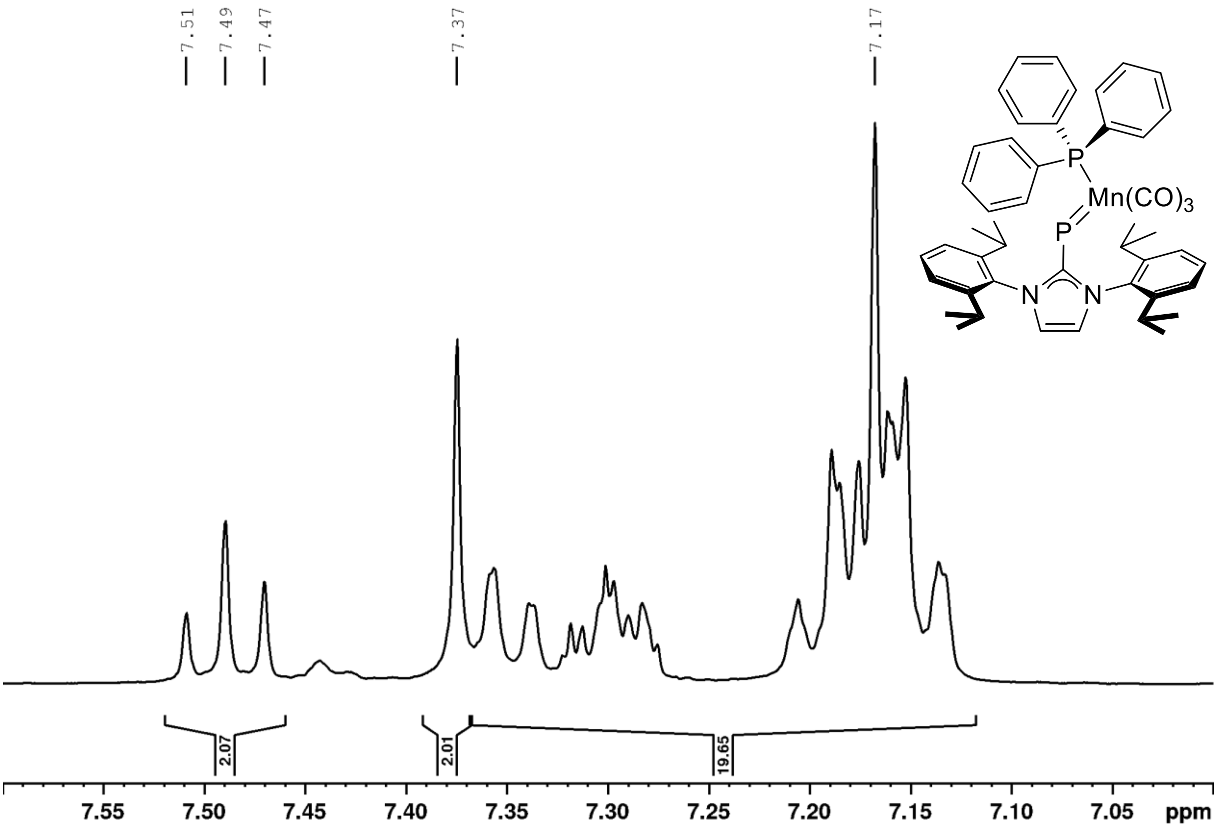

Figure S23: ^{1}H NMR spectrum of (IDipp)P(PPh$_3$)Mn(CO)$_3$ (400 MHz, CD_2Cl_2, 260 K, 7.0–7.6 ppm).

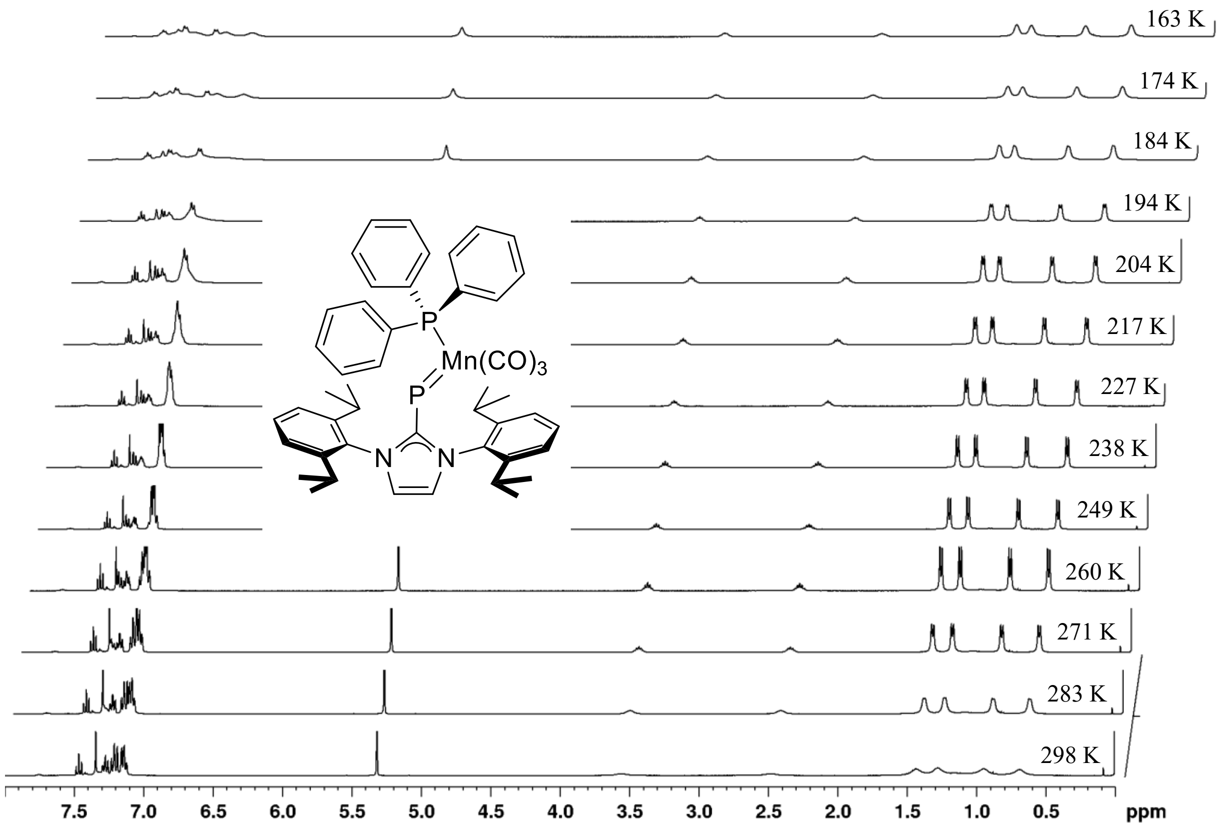

Figure S24: ^{1}H NMR spectrum of (IDipp)P(PPh$_3$)Mn(CO)$_3$ (400 MHz, CD_2Cl_2, 298–171 K, overview).

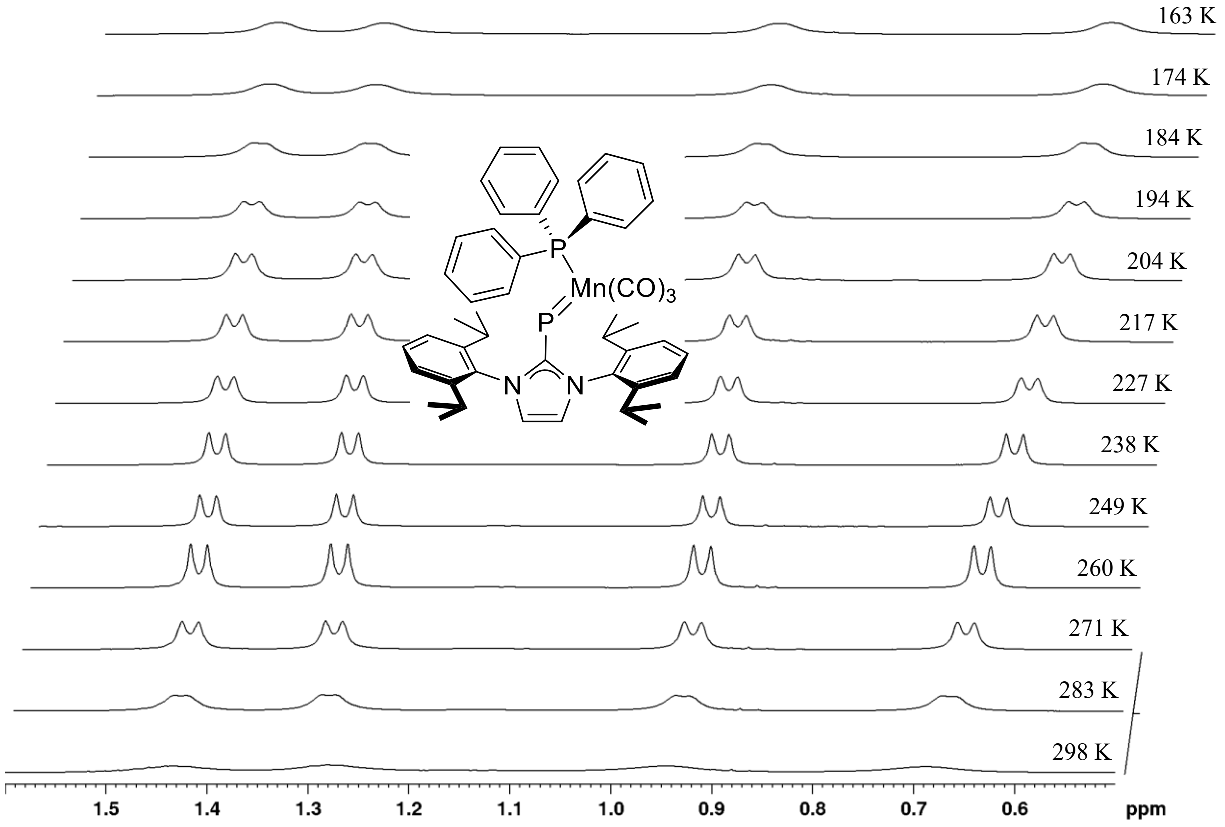

Figure S25: ^{1}H NMR spectrum of (IDipp)P(PPh$_3$)Mn(CO)$_3$ (400 MHz, CD_2Cl_2, 298–171 K, 0.5–1.6 ppm).

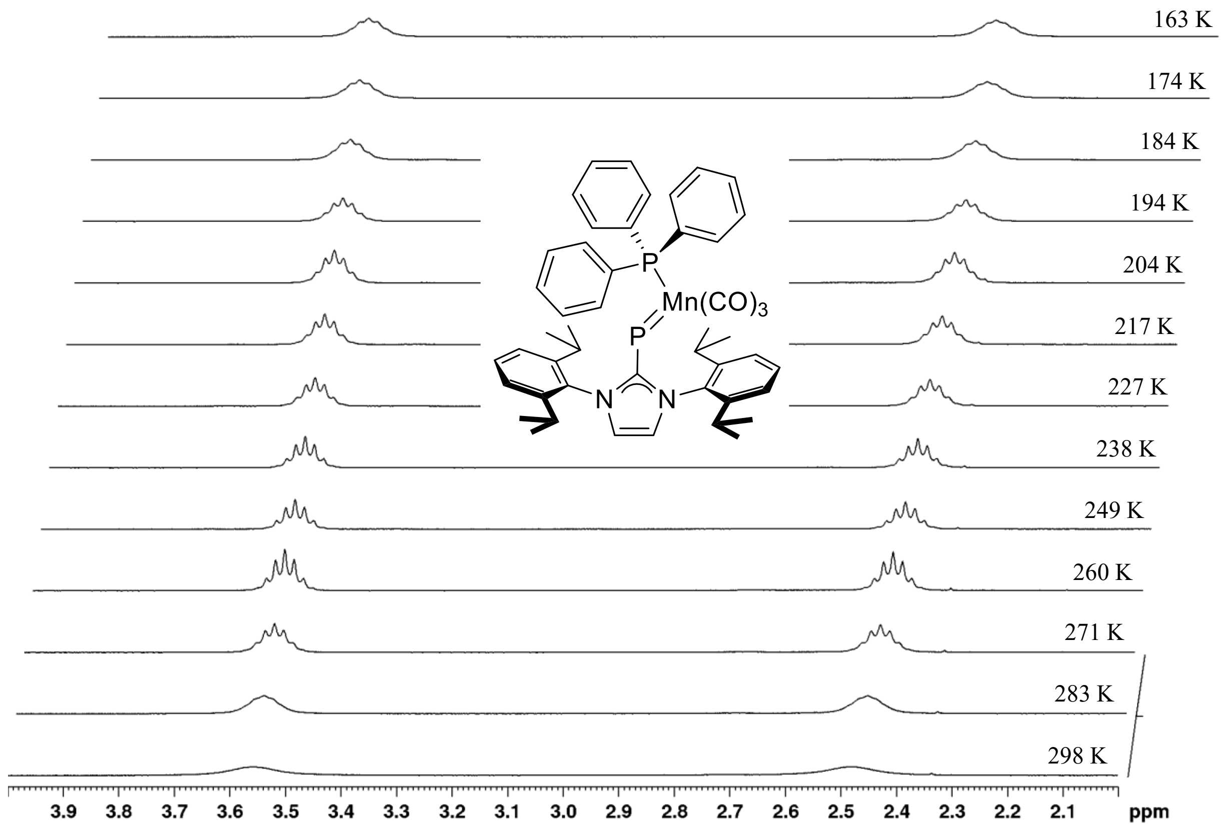

Figure S26: ^{1}H NMR spectrum of (IDipp)P(PPh3)Mn(CO)3 (400 MHz, CD_2Cl_2, 298–171 K, 2.0–4.0 ppm).

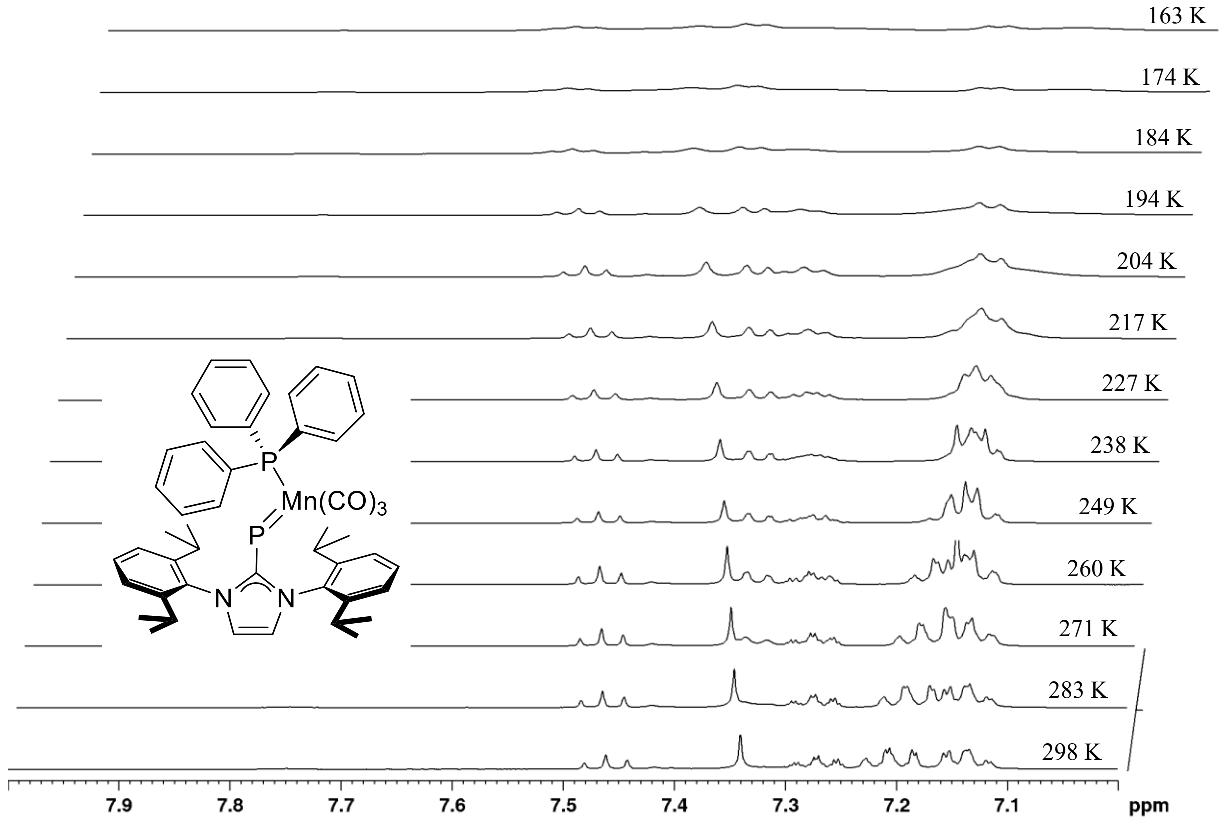

Figure S27: ^{1}H NMR spectrum of (IDipp)P(PPh3)Mn(CO)3 (400 MHz, CD_2Cl_2, 298–171 K, 7.0–8.0 ppm).

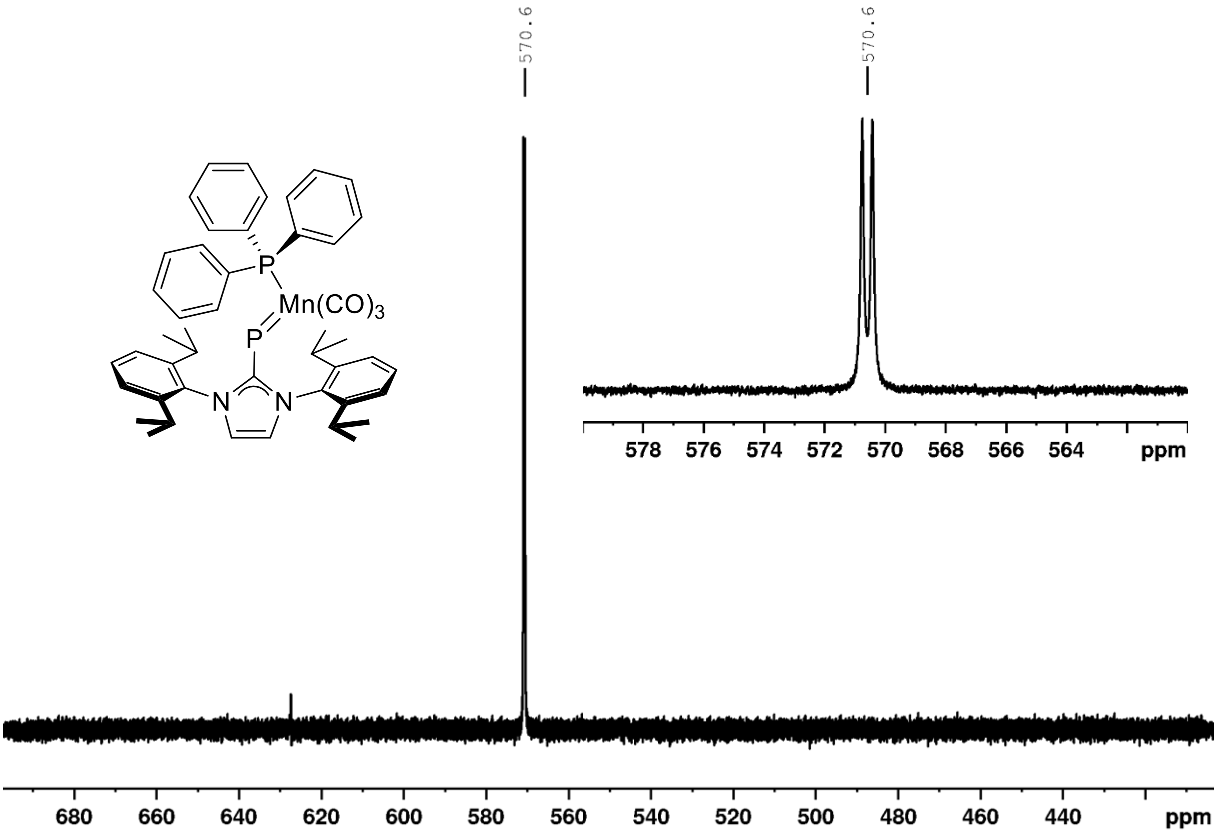

Figure S28: $^{31}P\{^{1}H\}$ NMR spectrum of (IDipp)P(PPh$_3$)Mn(CO)$_3$ (202.5 MHz, CD_2Cl_2, 298 K, 400–700 ppm).

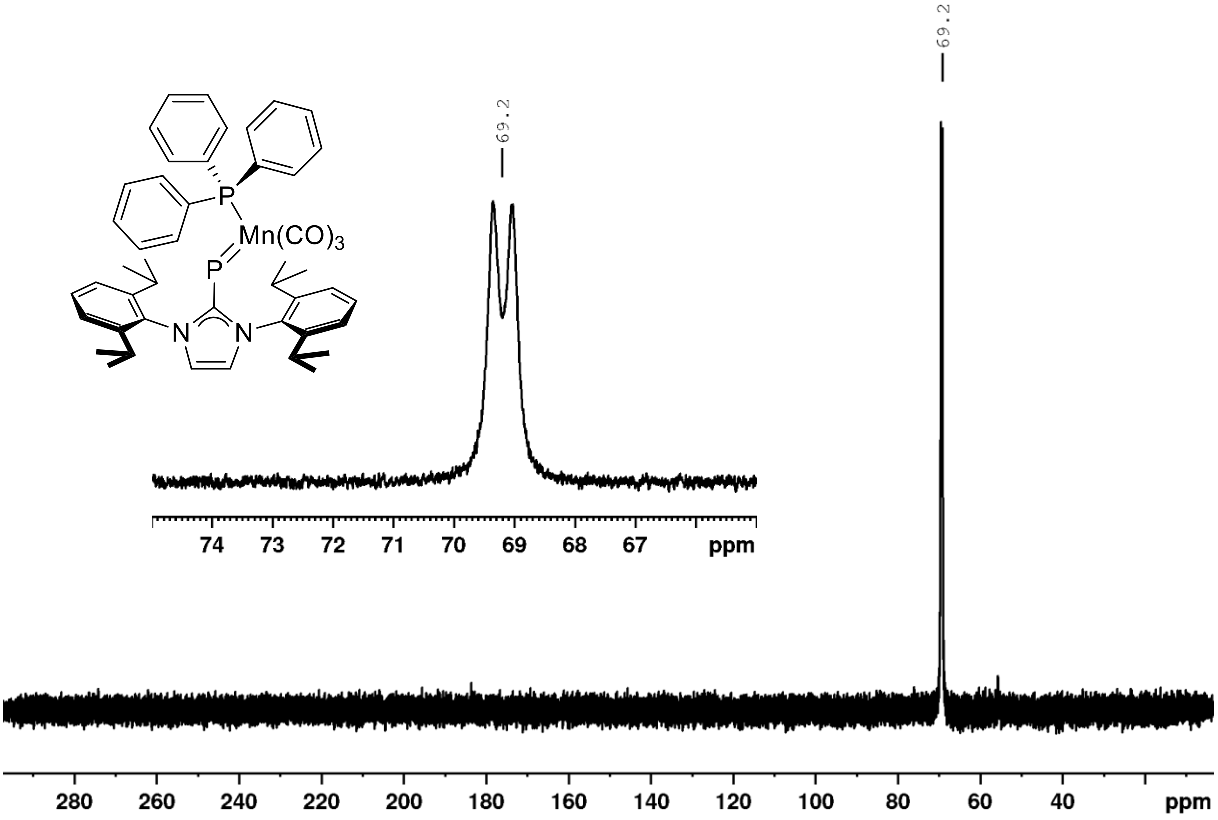

Figure 29: $^{31}P\{^{1}H\}$ NMR spectrum of (IDipp)P(PPh$_3$)Mn(CO)$_3$ (202.5 MHz, CD_2Cl_2, 298 K, , 0–300 ppm).

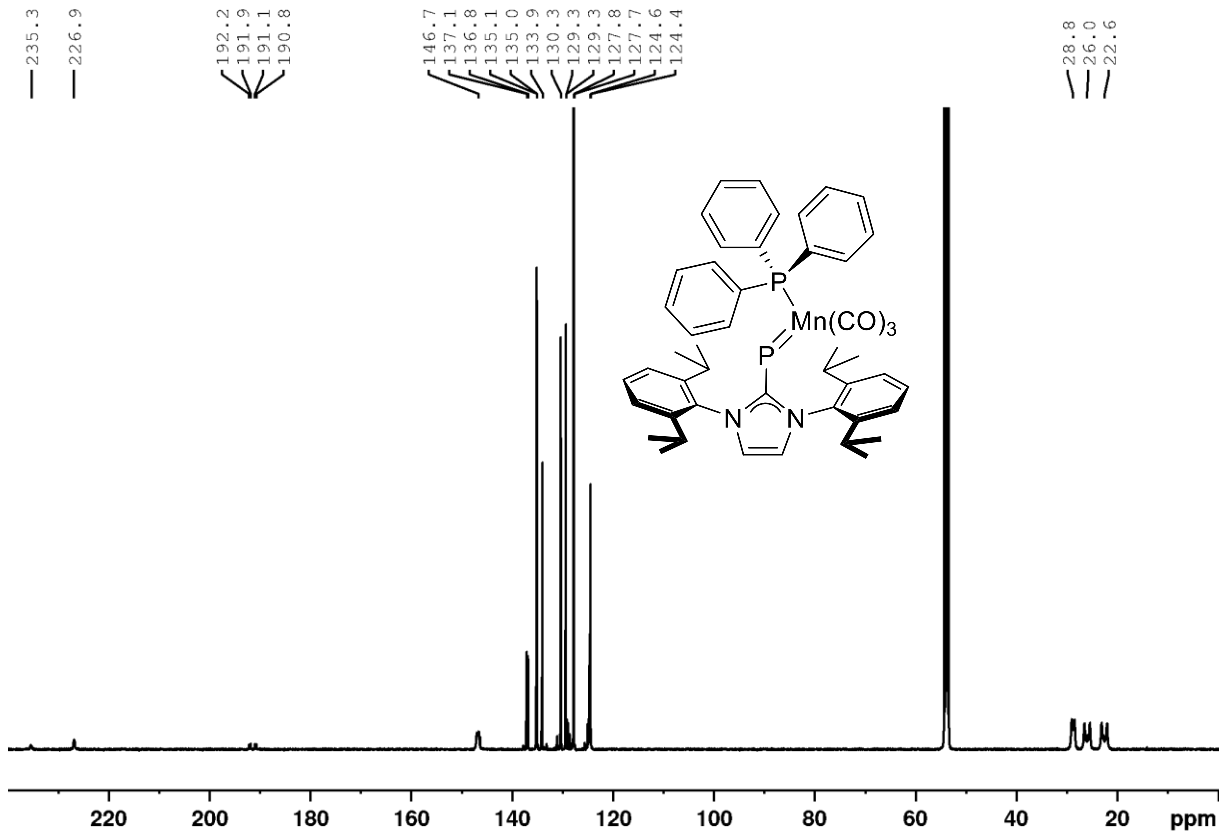

Figure S30: $^{13}C\{^{1}H\}$ NMR spectrum of (IDipp)P(PPh$_3$)Mn(CO)$_3$ (101 MHz, CD_2Cl_2, 298 K, overview).

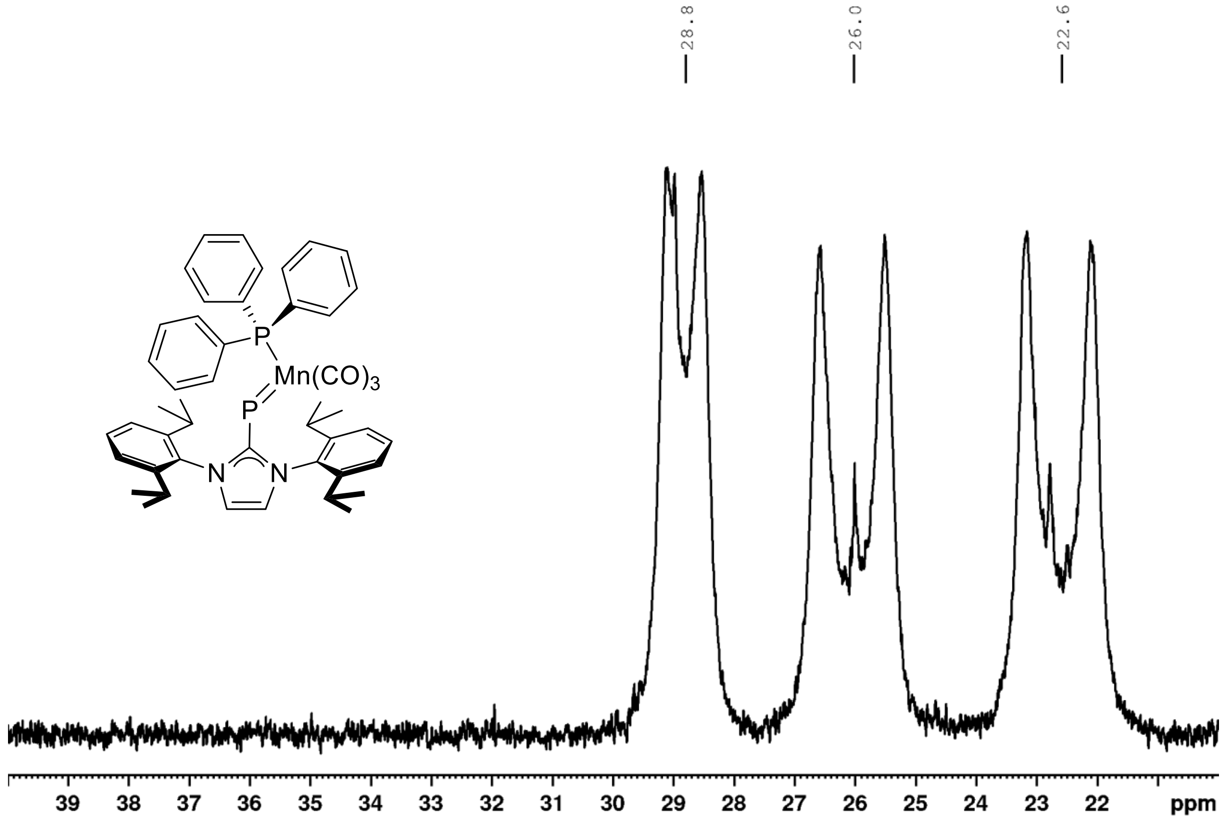

Figure S31: $^{13}C\{^{1}H\}$ NMR spectrum of (IDipp)P(PPh$_3$)Mn(CO)$_3$ (101 MHz, CD_2Cl_2, 298 K, 20–40 ppm).

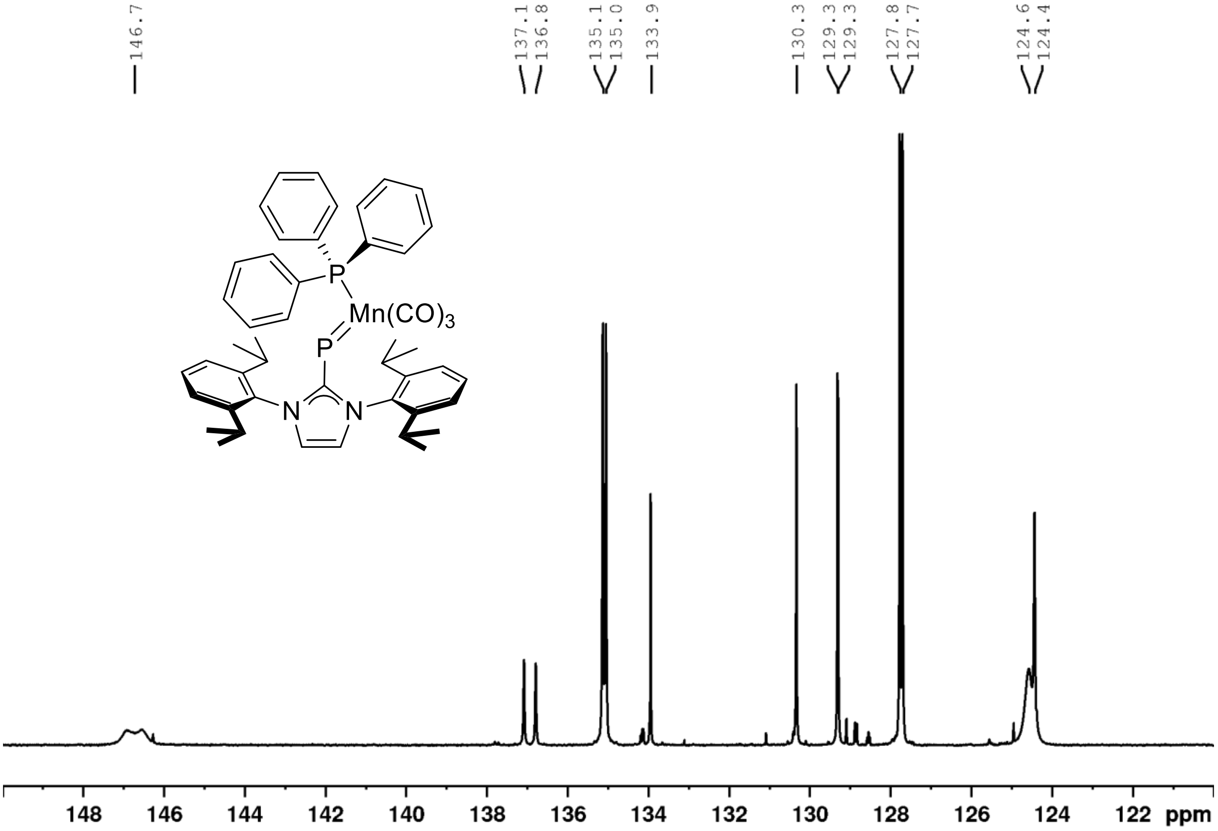

Figure S32: $^{13}C\{^{1}H\}$ NMR spectrum of (IDipp)P(PPh$_3$)Mn(CO)$_3$ (101 MHz, CD_2Cl_2, 298 K, 120–150 ppm).

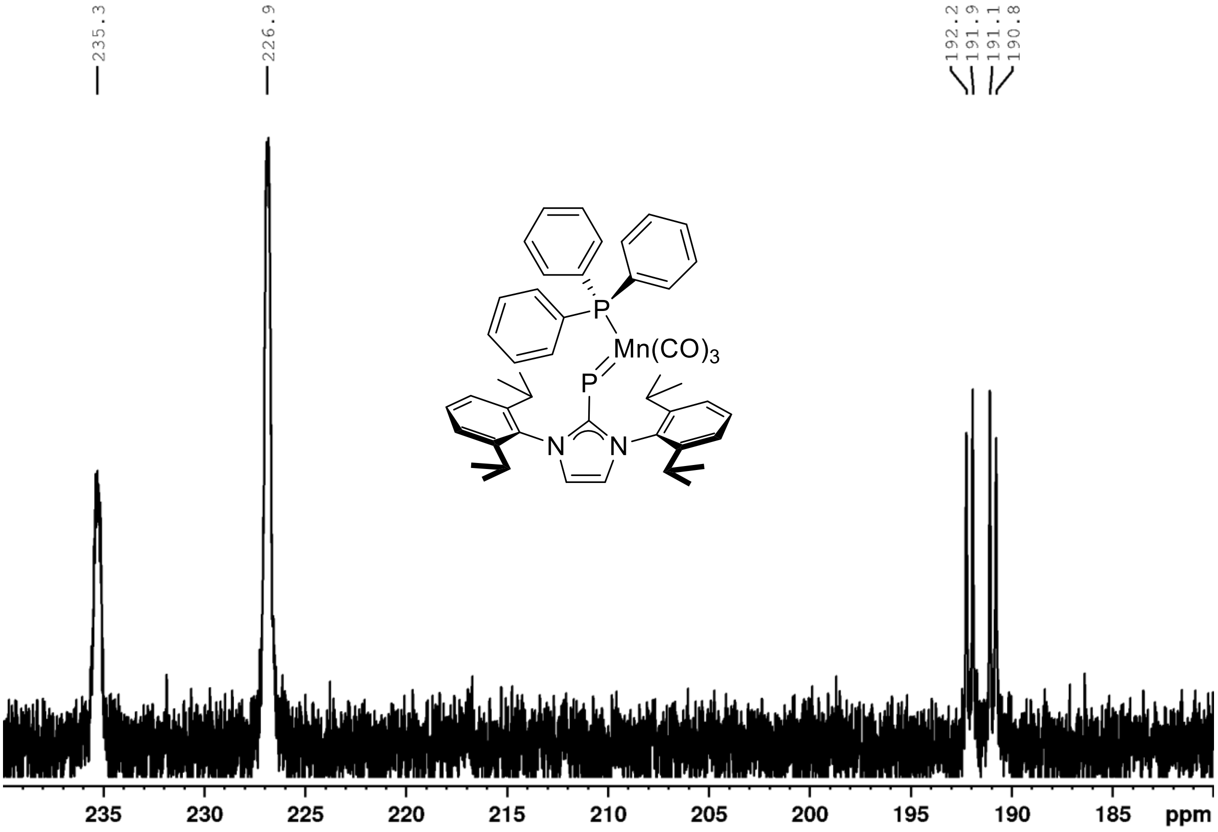

Figure S33: $^{13}C\{^{1}H\}$ NMR spectrum of (IDipp)P(PPh$_3$)Mn(CO)$_3$ (101 MHz, CD_2Cl_2, 298 K, 180–240 ppm).

S3.3. (IDipp)PMn(PMe3)(CO)3

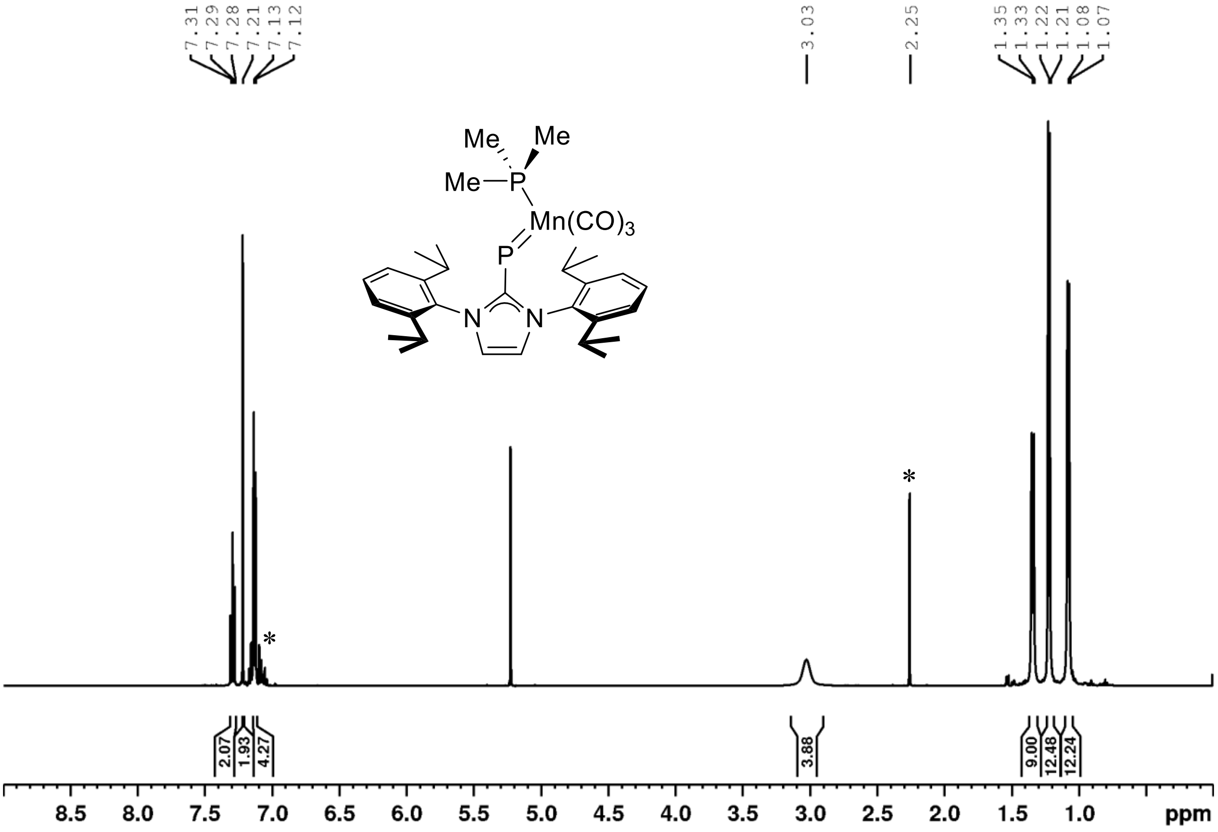

Figure S34: ^{1}H NMR spectrum of (IDipp)P(PMe$_3$)Mn(CO)$_3$ (500 MHz, CD_2Cl_2, 298 K, overview); * = residual toluene.

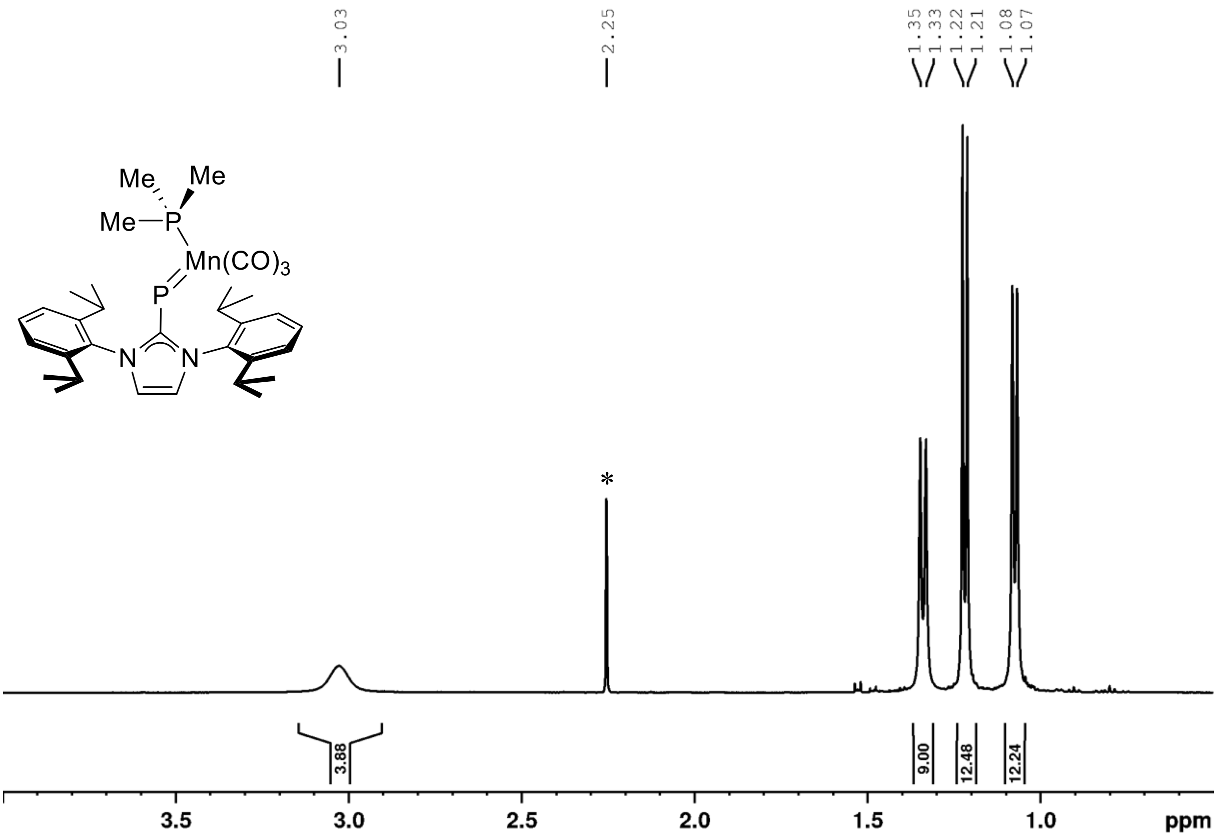

Figure S35: ^{1}H NMR spectrum of (IDipp)P(PMe_3)Mn$(CO)_3$ (500 MHz, CD_2Cl_2, 298 K, 0.5–4.0 ppm); * = residual toluene.

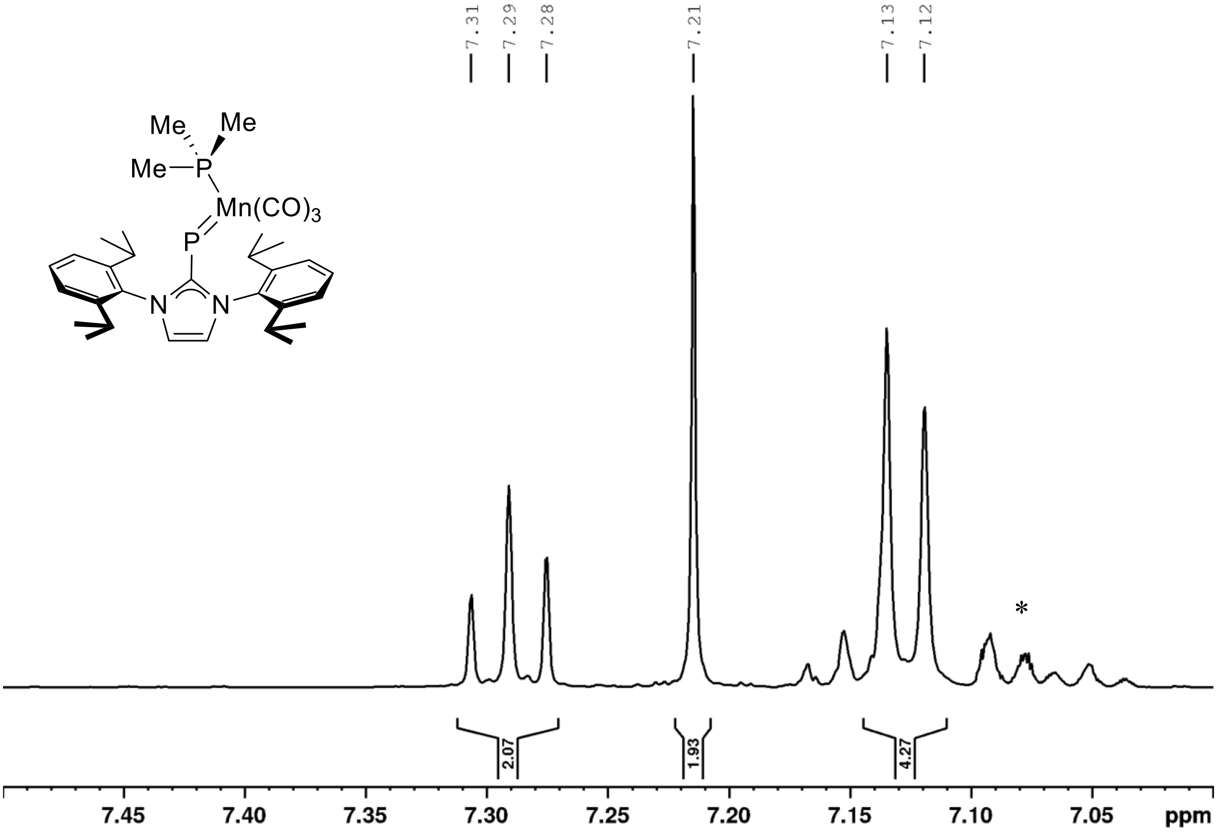

Figure S36: ^{1}H NMR spectrum of (IDipp)P(PMe_3)Mn$(CO)_3$ (500 MHz, CD_2Cl_2, 298 K, 7.0–7.5 ppm); * = residual toluene.

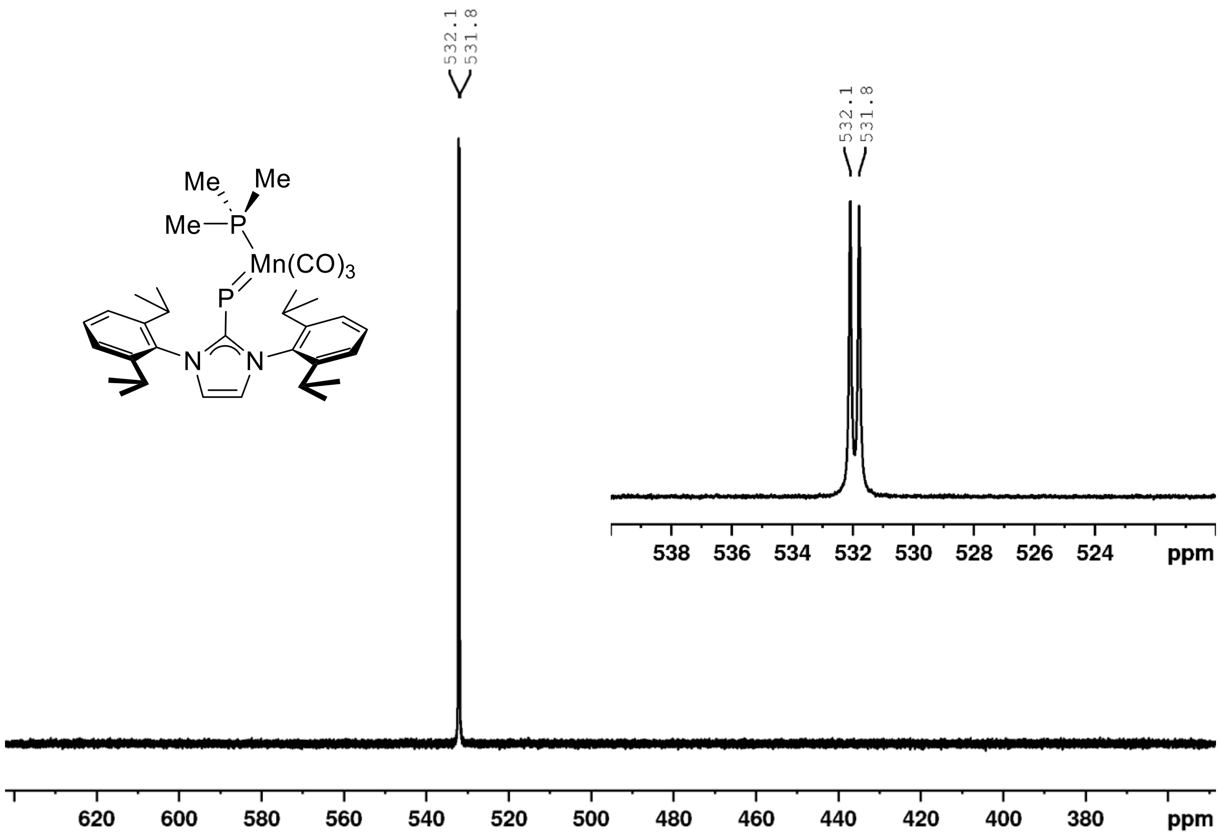

Figure S37: $^{31}P\{^{1}H\}$ NMR spectrum of (IDipp)P(PMe$_3$)Mn(CO)$_3$ (202.5 MHz, CD_2Cl_2, 298 K, 350–640 ppm).

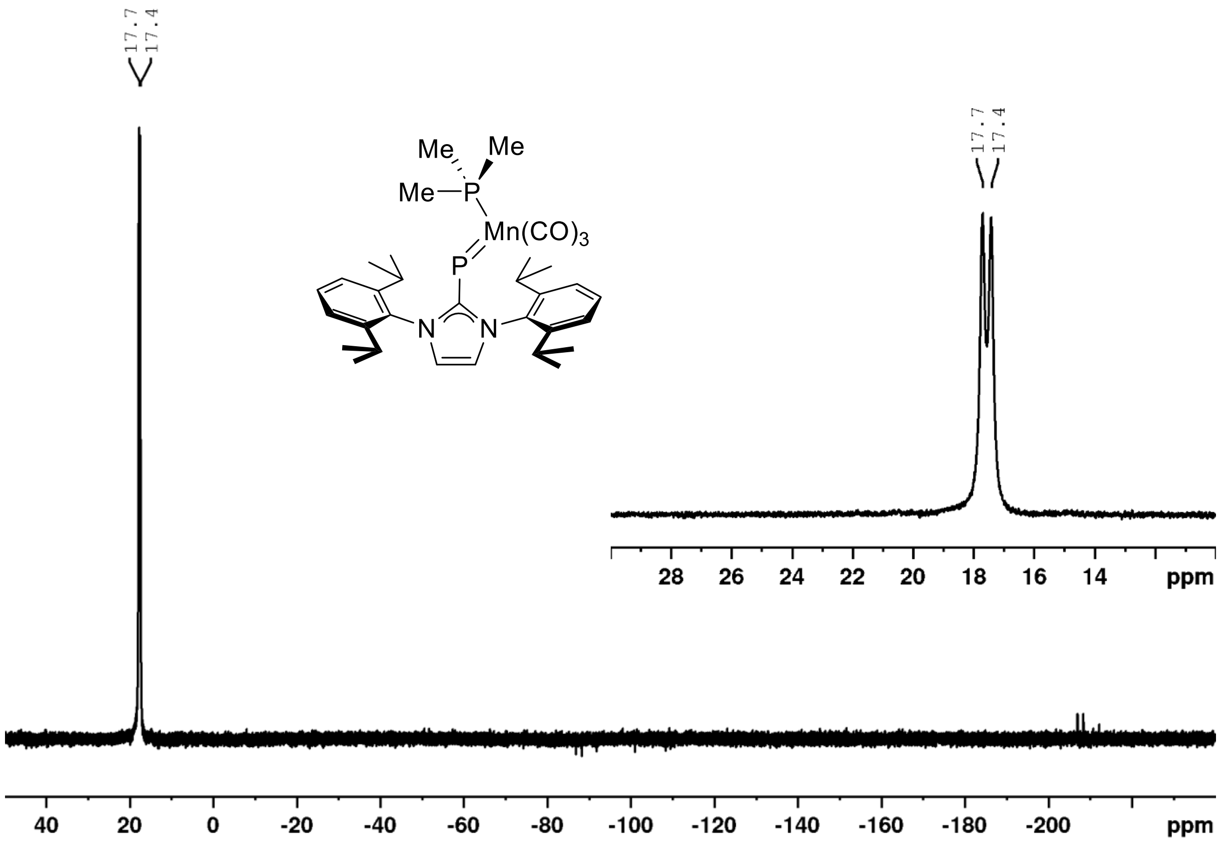

Figure S38: $^{31}P\{^{1}H\}$ NMR spectrum of (IDipp)P(PMe$_3$)Mn(CO)$_3$ (202.5 MHz, CD_2Cl_2, 298 K, –240–50 ppm).

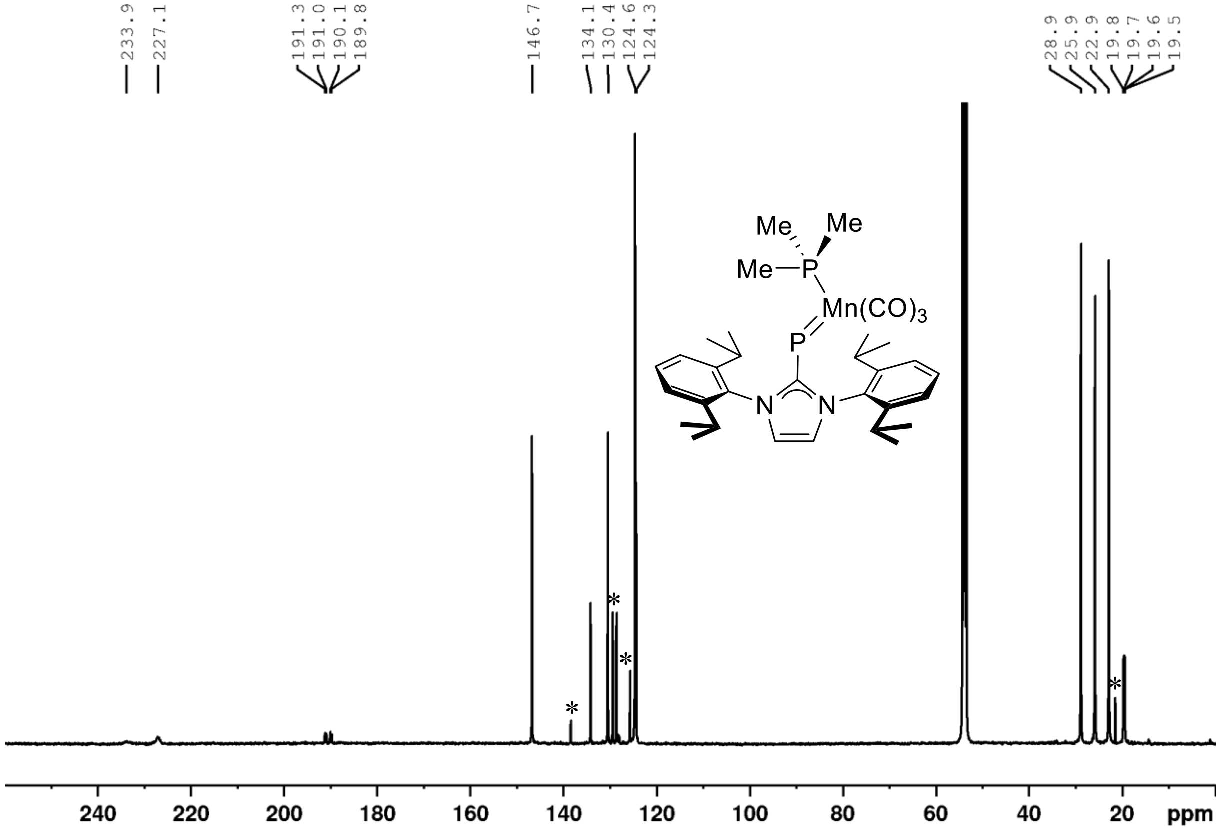

Figure S39: $^{13}C\{^{1}H\}$ NMR spectrum of (IDipp)P(PMe$_3$)Mn(CO)$_3$ (101 MHz, CD_2Cl_2, 298 K, overview); * = residual toluene.

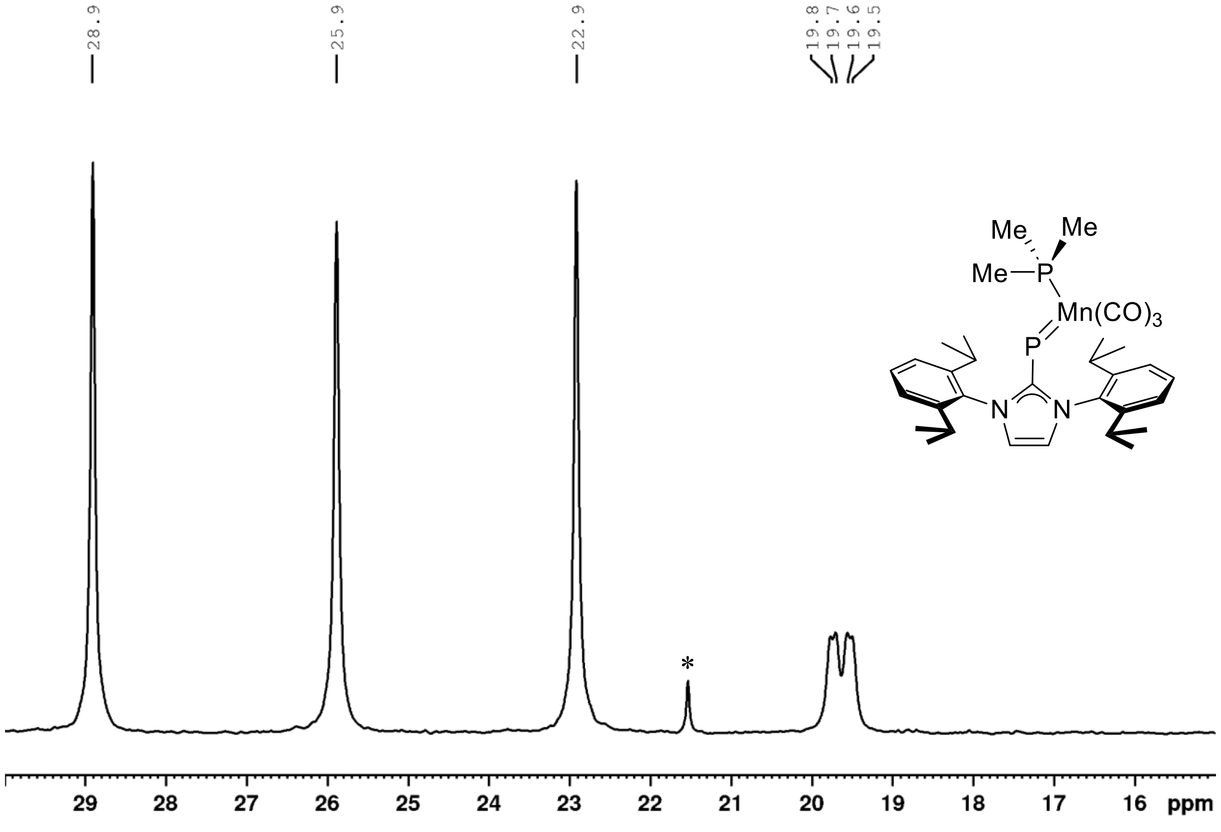

Figure S40: $^{13}C\{^{1}H\}$ NMR spectrum of (IDipp)P(PMe$_3$)Mn(CO)$_3$ (101 MHz, CD_2Cl_2, 298 K, 15–30 ppm); * = residual toluene.

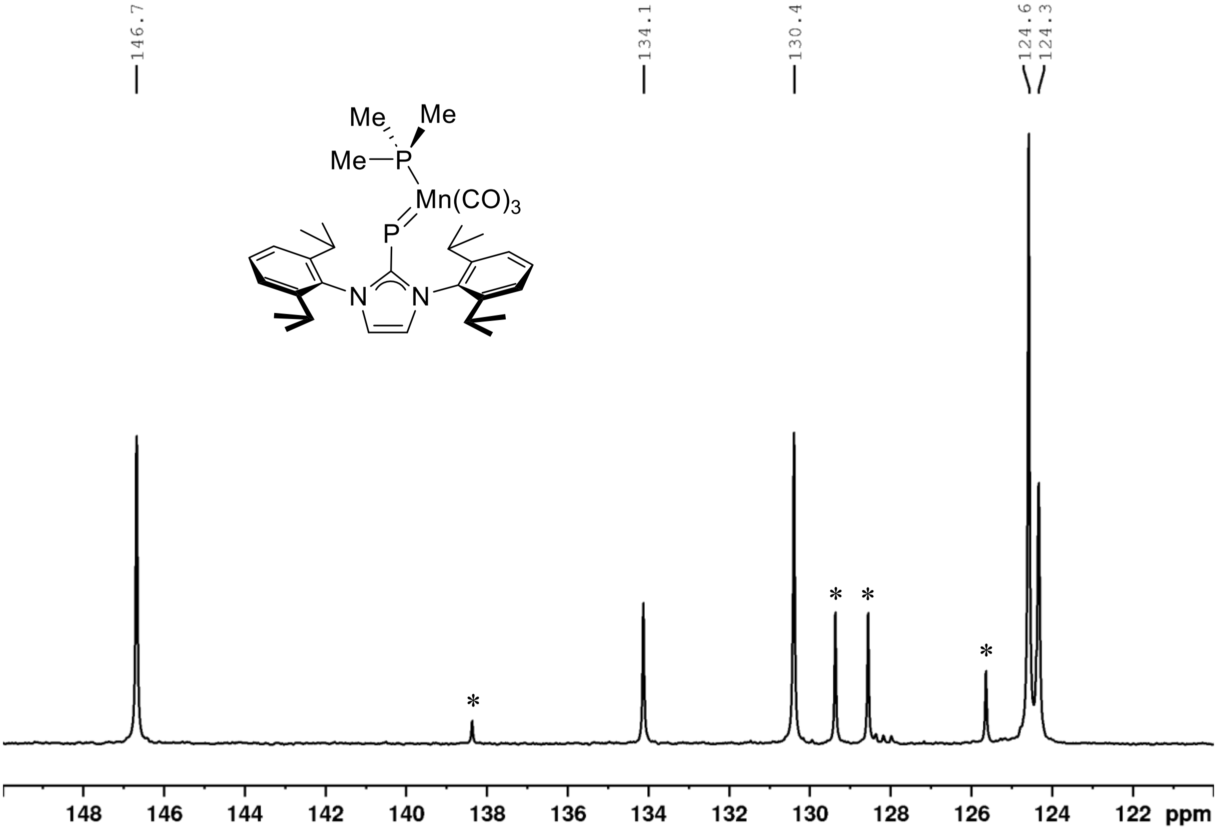

Figure S41: $^{13}C\{^1H\}$ NMR spectrum of (IDipp)P(PMe$_3$)Mn(CO)$_3$ (101 MHz, CD_2Cl_2, 298 K, 120–150 ppm); * = residual toluene.

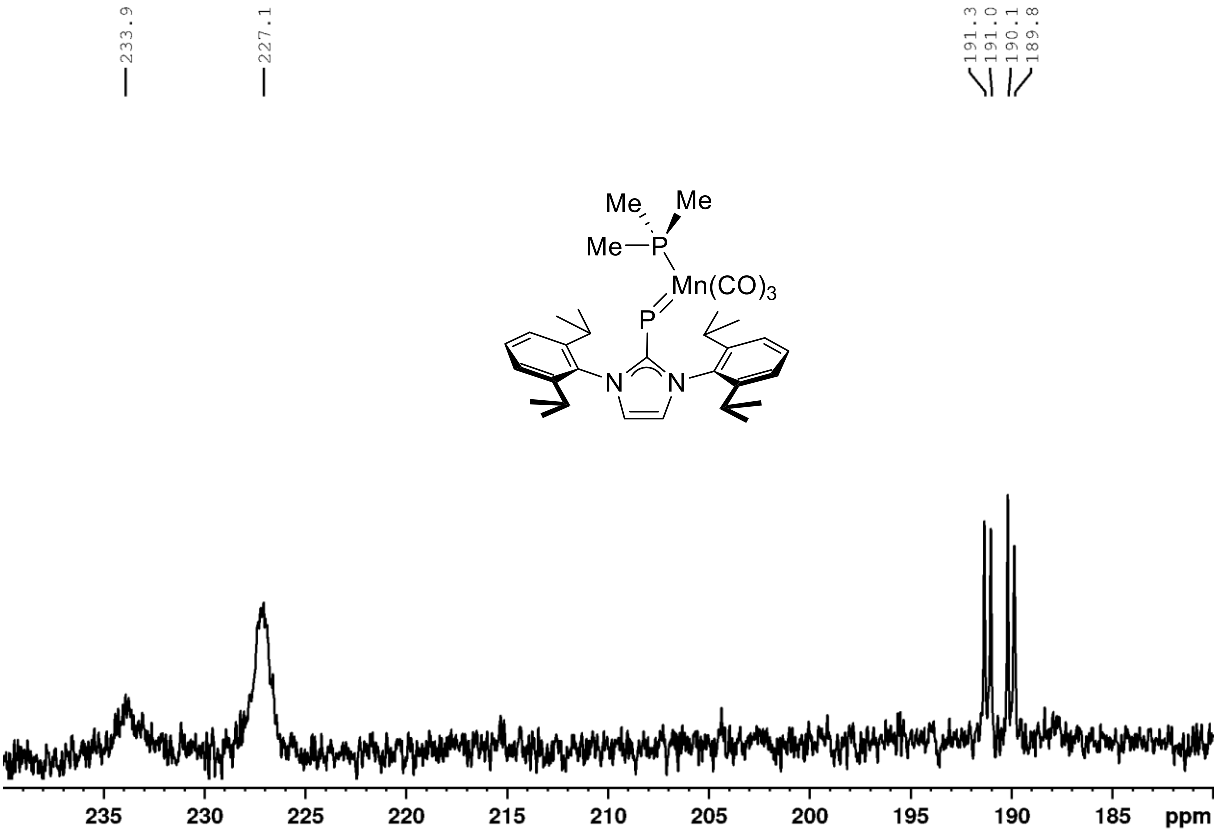

Figure S42: $^{13}C\{^1H\}$ NMR spectrum of (IDipp)P(PMe$_3$)Mn(CO)$_3$ (101 MHz, CD_2Cl_2, 298 K, 180–240 ppm.

S3.4. (IDipp)PMn(IMe)(CO)3

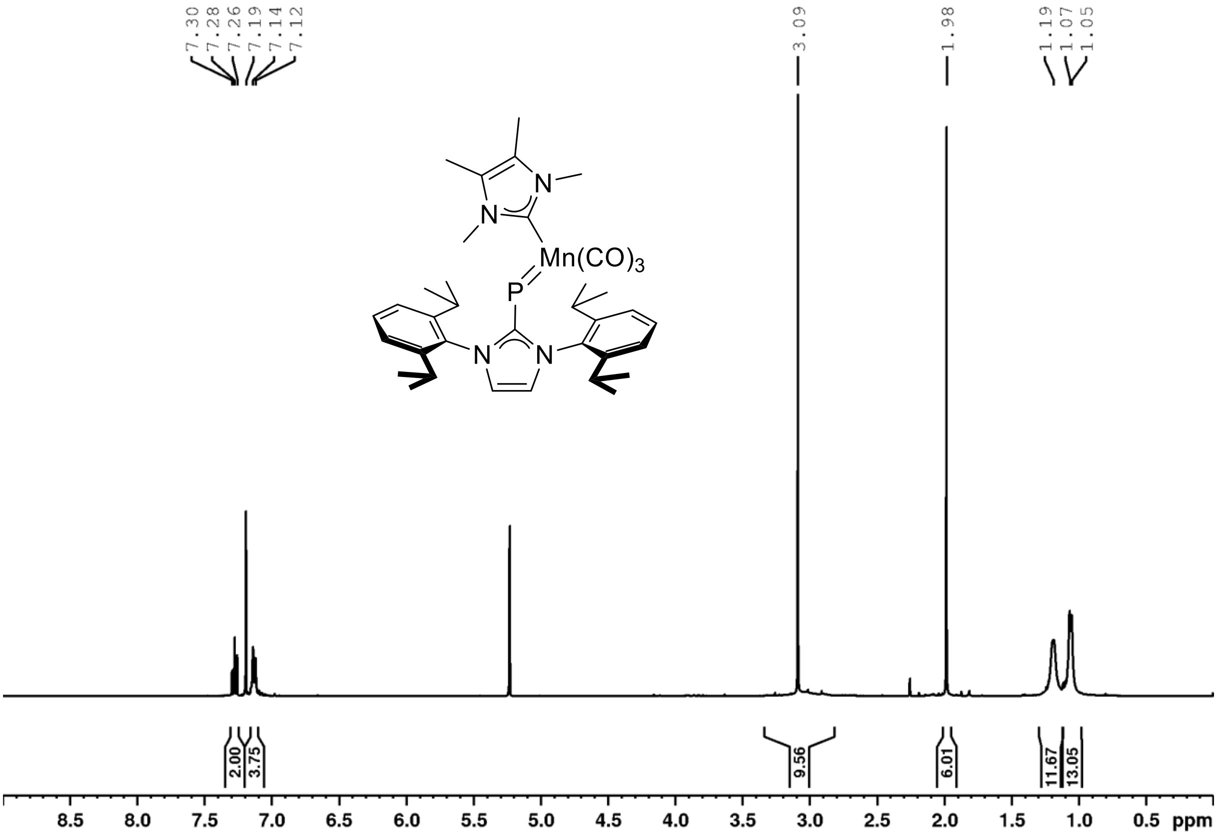

Figure S43: ^{1}H NMR spectrum of (IDipp)P(IMe)Mn(CO)$_3$ (400 MHz, CD_2Cl_2, 298 K, overview).

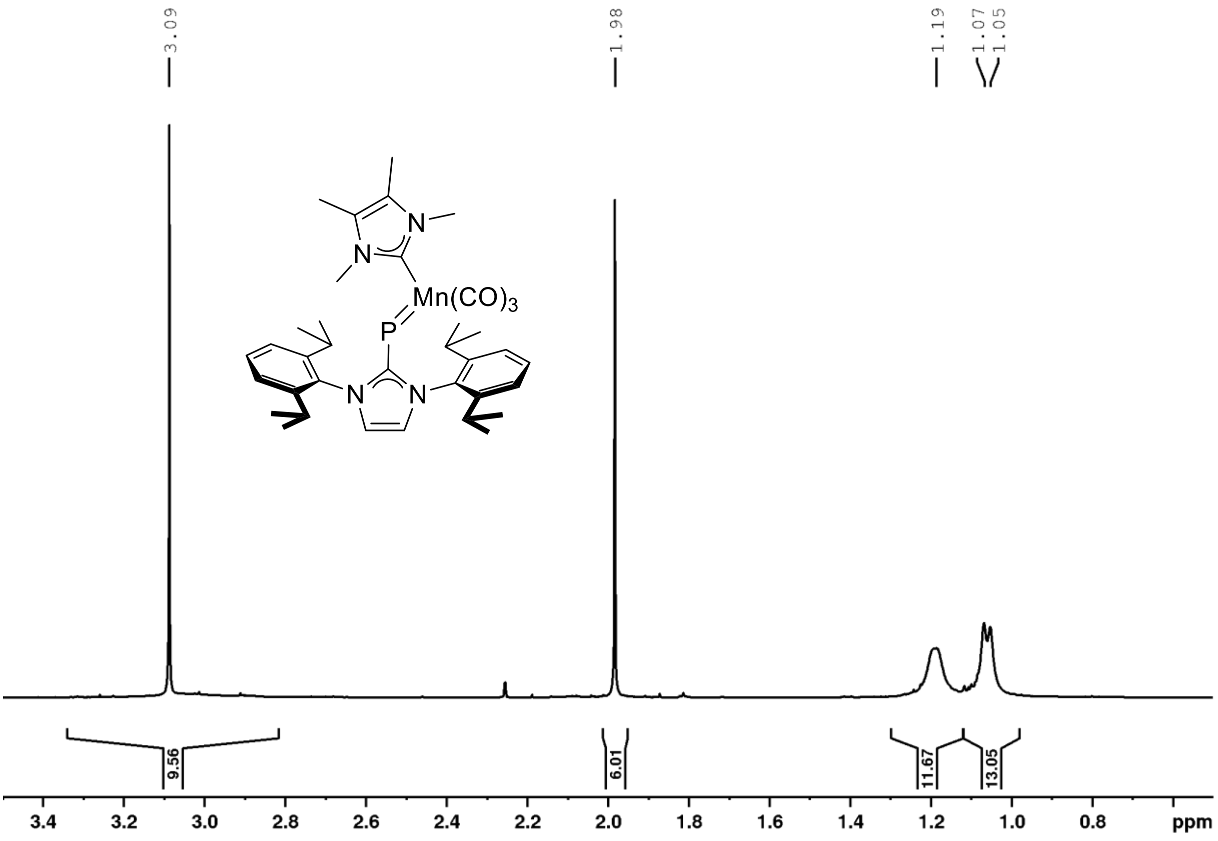

Figure S44: ^{1}H NMR spectrum of (IDipp)P(IMe)Mn(CO)$_3$ (400 MHz, CD_2Cl_2, 298 K, 0.5–3.5 ppm).

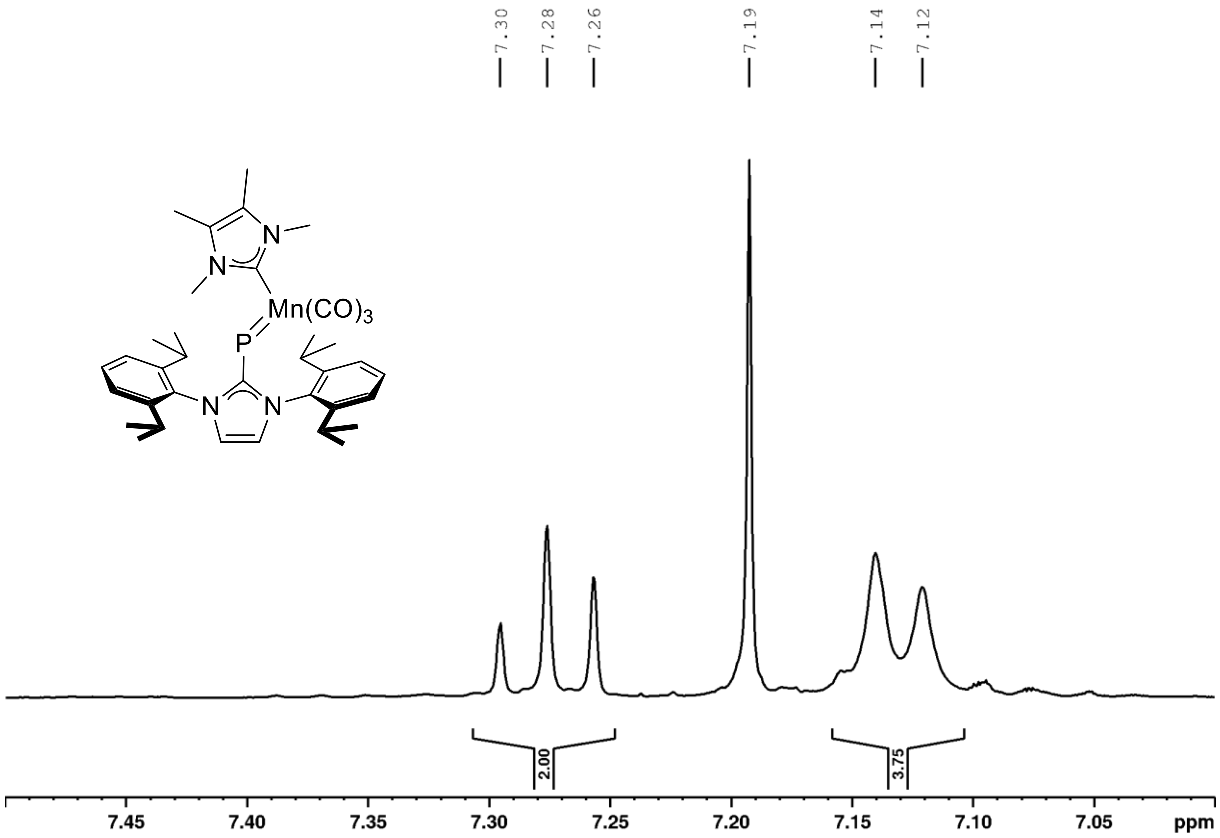

Figure S45: ^{1}H NMR spectrum of (IDipp)P(IMe)Mn(CO)$_3$ (400 MHz, CD_2Cl_2, 298 K, 7.0–7.5 ppm).

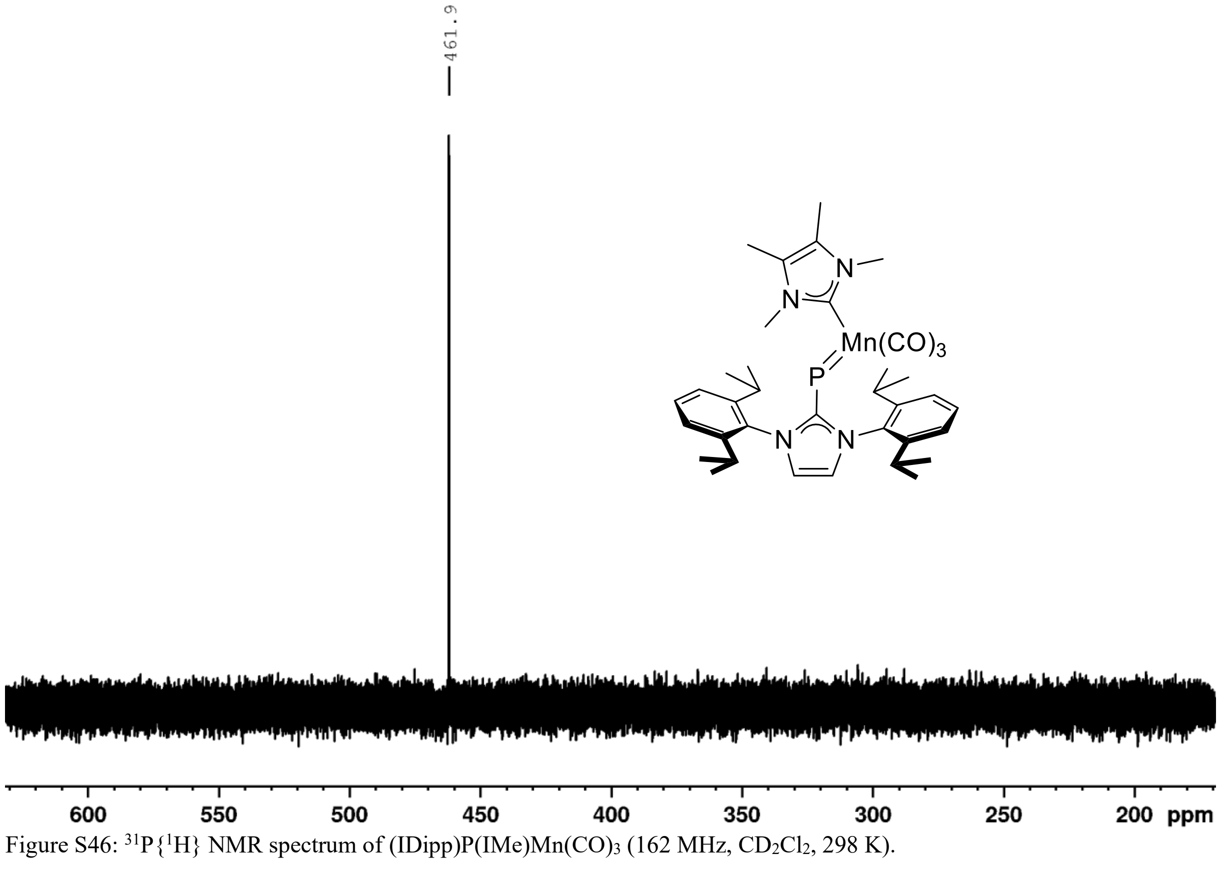

Figure S46: $^{31}P\{^{1}H\}$ NMR spectrum of (IDipp)P(IMe)Mn(CO)$_3$ (162 MHz, CD_2Cl_2, 298 K).

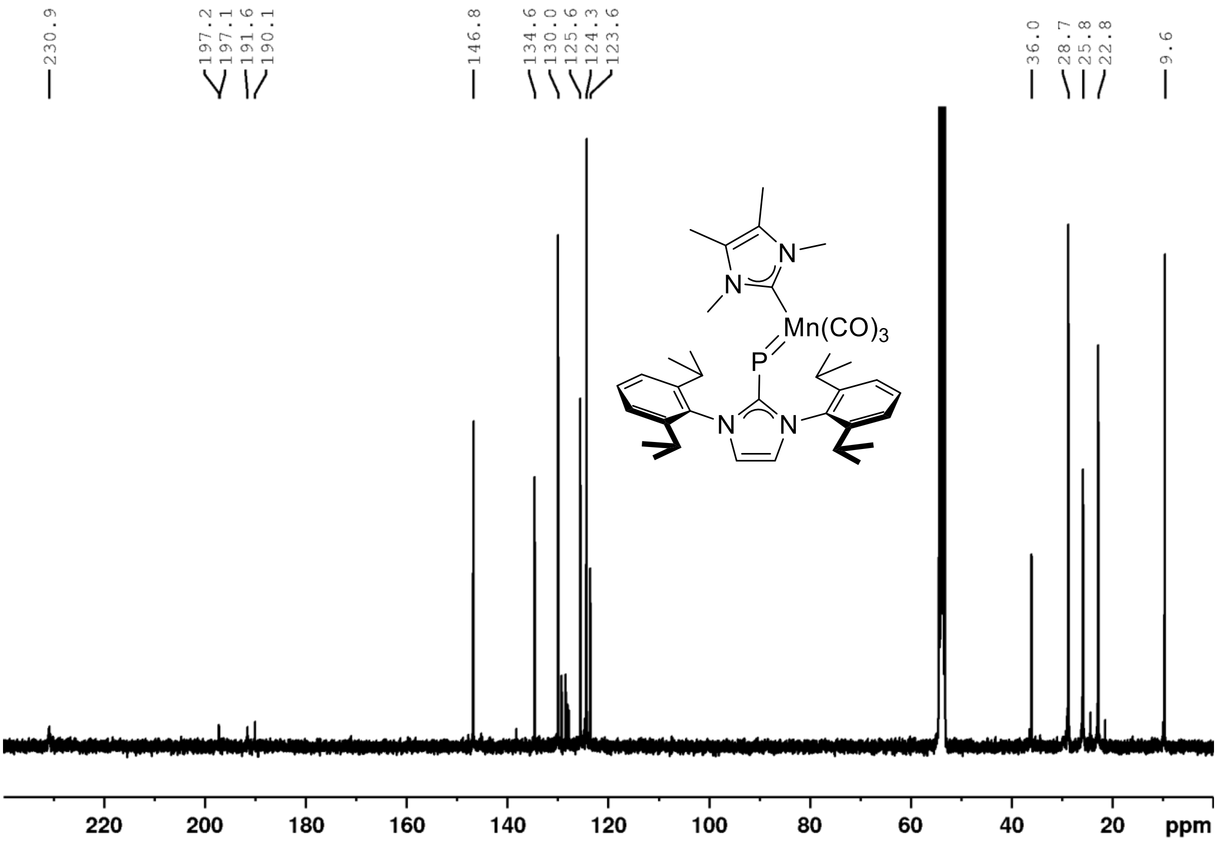

Figure S47: $^{13}C\{^{1}H\}$ NMR spectrum of (IDipp)P(IMe)Mn(CO)$_3$ (101 MHz, CD_2Cl_2, 298 K, overview).

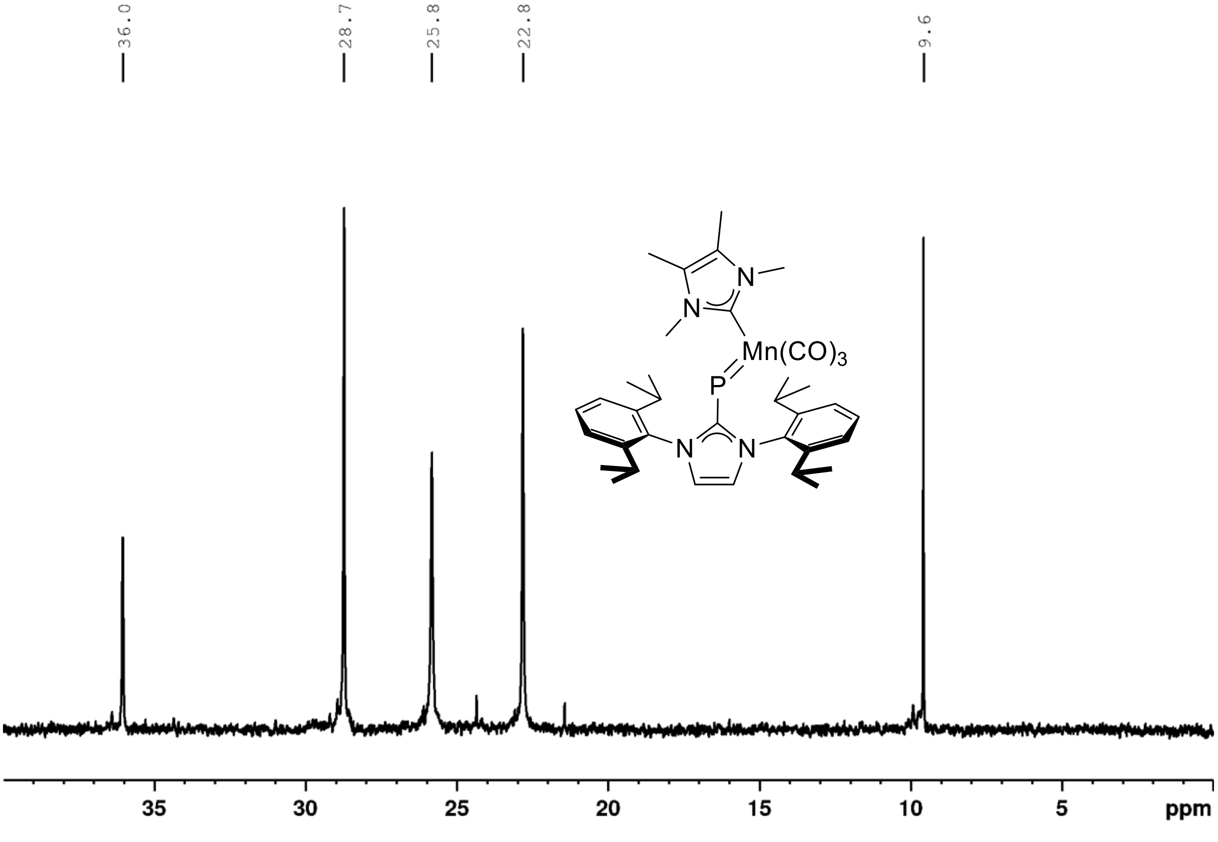

Figure S48: $^{13}C\{^{1}H\}$ NMR spectrum of (IDipp)P(IMe)Mn(CO)$_3$ (101 MHz, CD_2Cl_2, 298 K, 0–40 ppm).

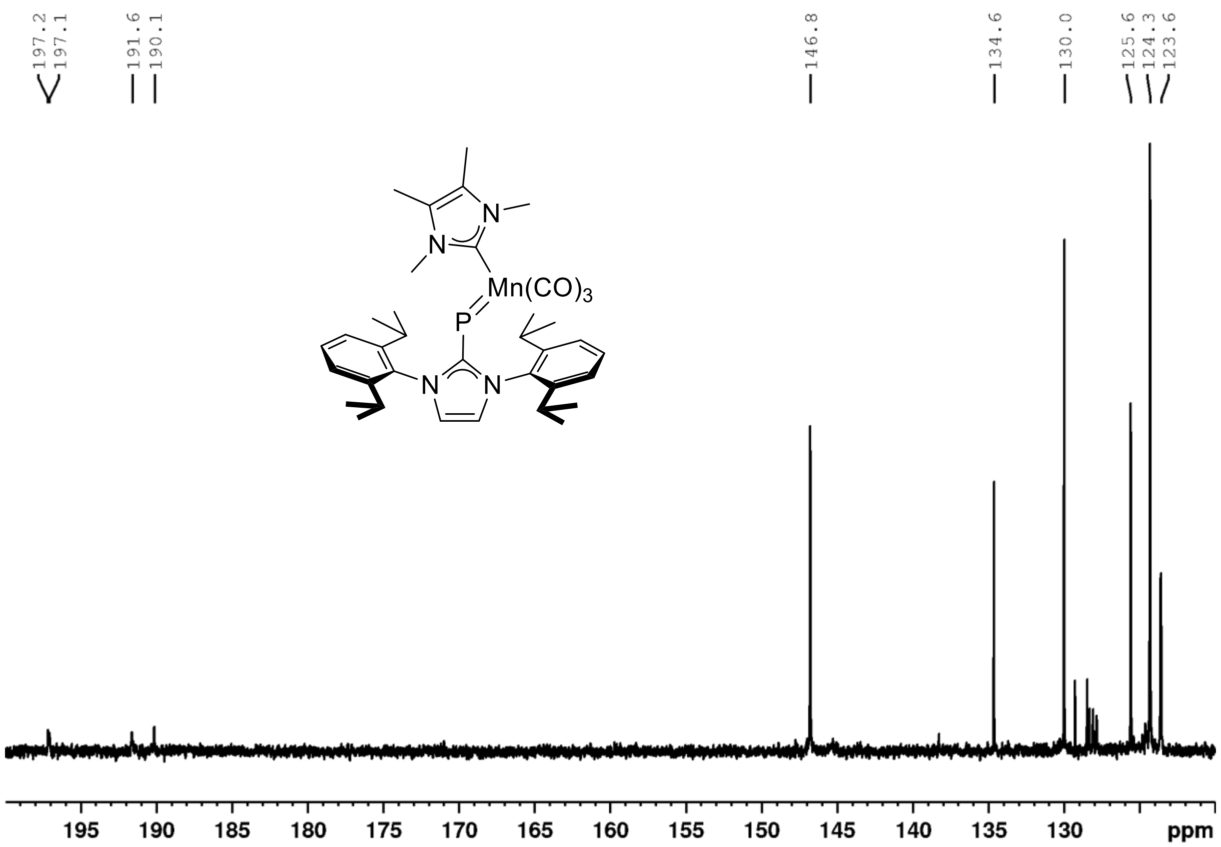

Figure S49: $^{13}C\{^{1}H\}$ NMR spectrum of (IDipp)P(IMe)Mn(CO)3 (101 MHz, CD_2Cl_2, 298 K, 120–200 ppm).

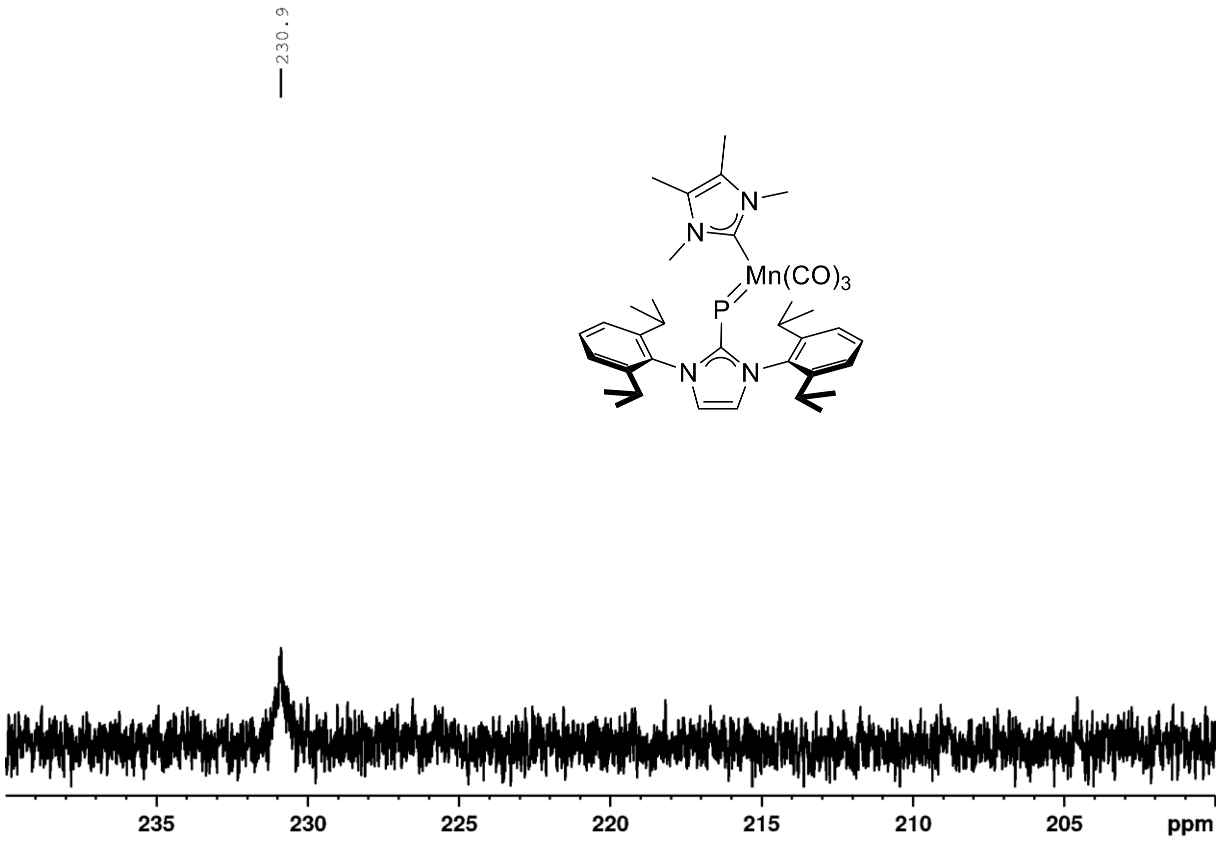

Figure S50: $^{13}C\{^{1}H\}$ NMR spectrum of (IDipp)P(IMe)Mn(CO)3 (101 MHz, CD_2Cl_2, 298 K, 200–240 ppm).

S3.5. (IDipp)PMn(XyNC)(CO)$_3$

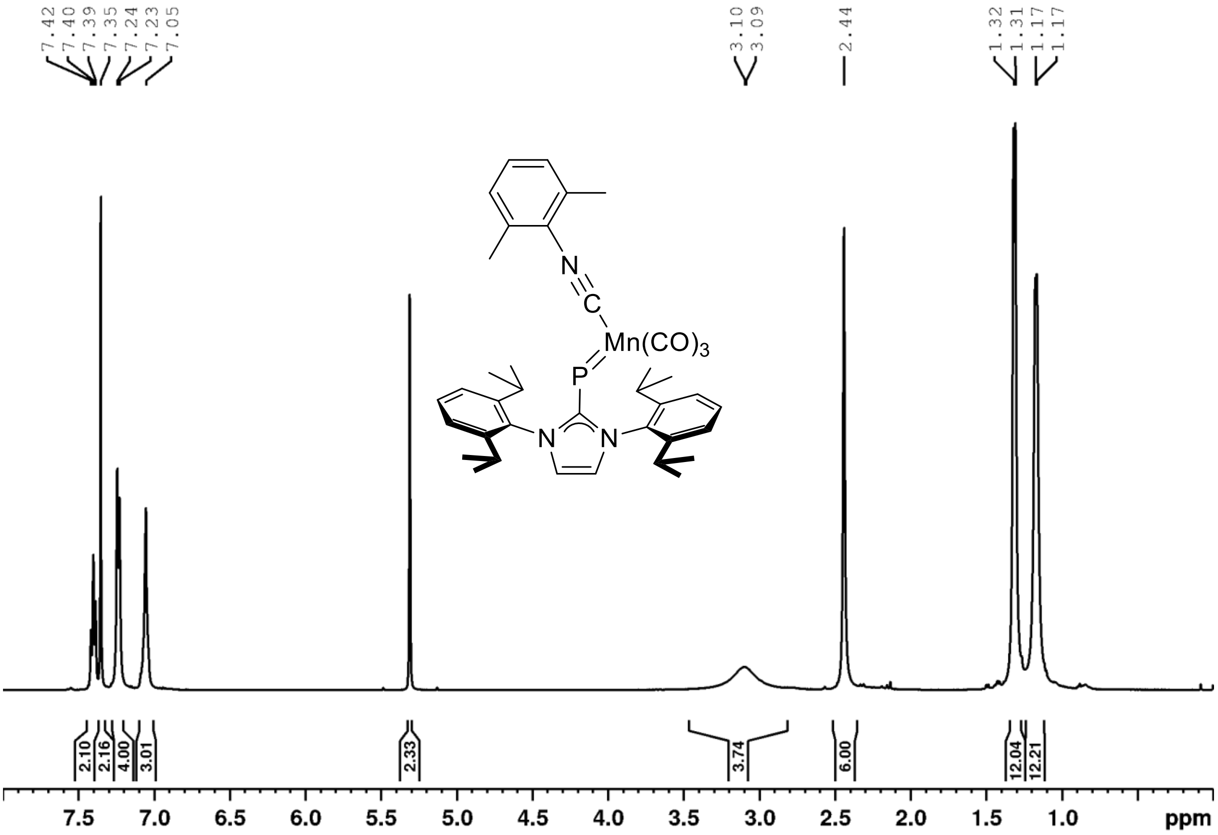

Figure S51: ^{1}H NMR spectrum of (IDipp)P(XyNC)Mn(CO)$_3$ (500 MHz, CD_2Cl_2, 298 K, overview).

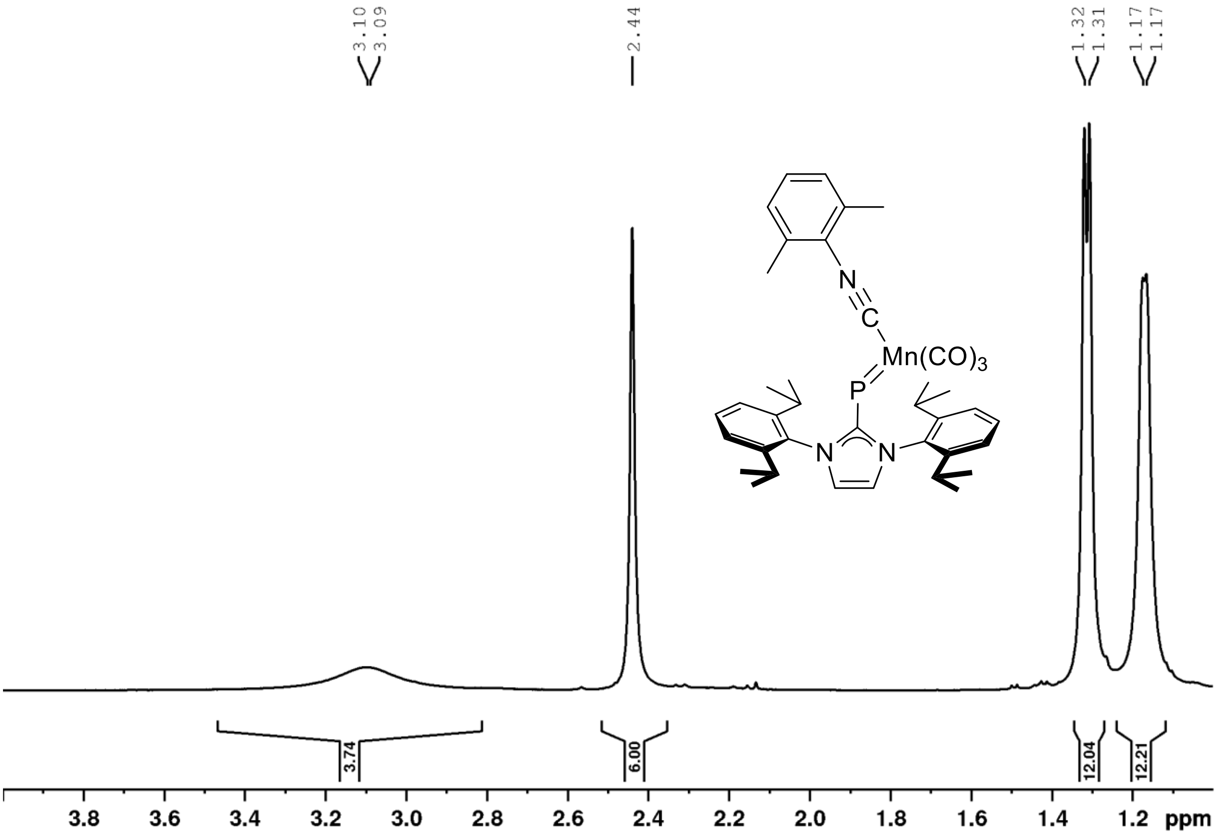

Figure S52: ^{1}H NMR spectrum of (IDipp)P(XyNC)Mn(CO)$_3$ (500 MHz, CD_2Cl_2, 298 K, 1.0–4.0 ppm).

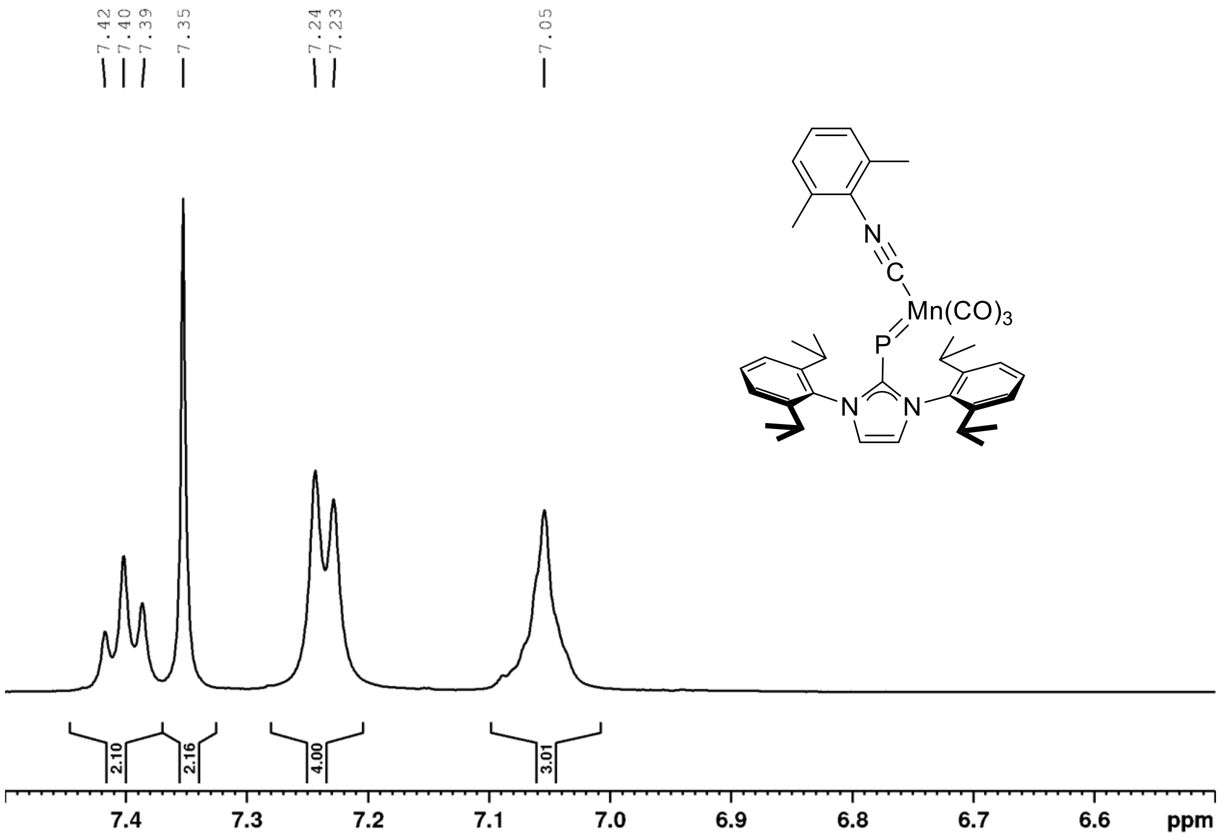

Figure S53: ^{1}H NMR spectrum of (IDipp)P(XyNC)Mn(CO)$_3$ (500 MHz, CD_2Cl_2, 298 K, 6.5–7.5 ppm).

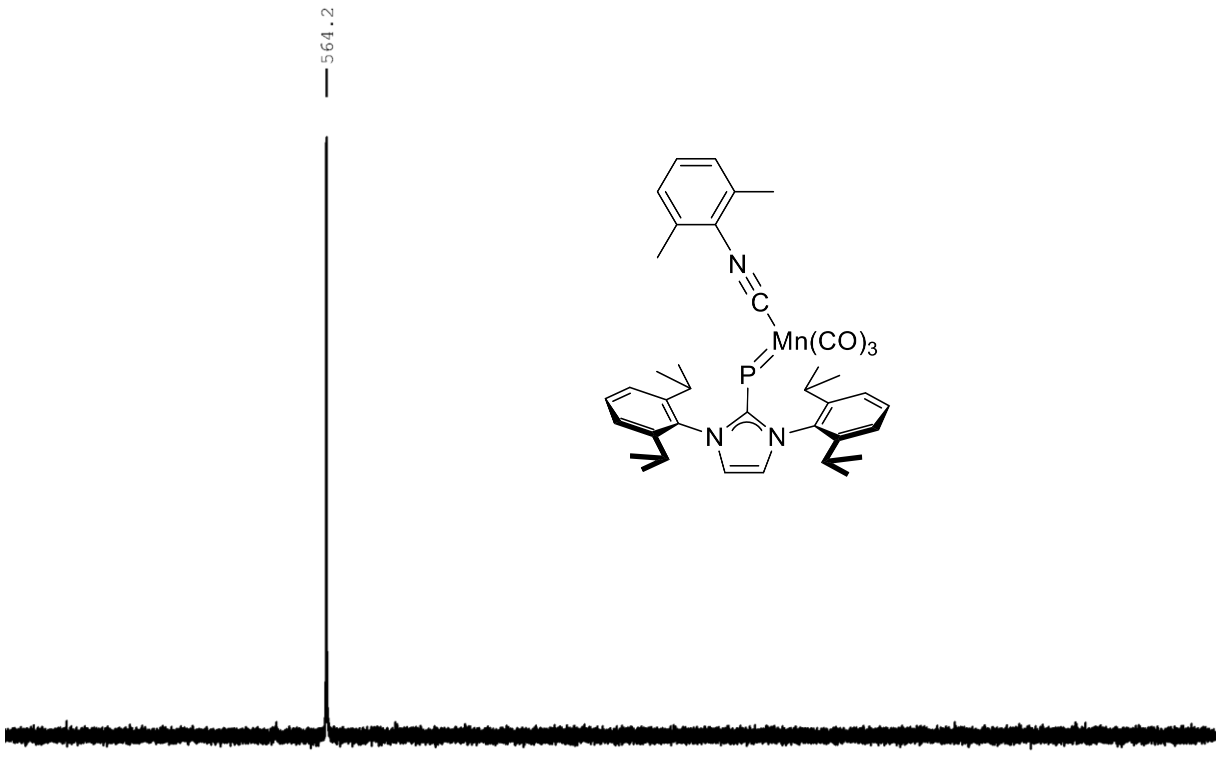

Figure S54: ^{31}P{^{1}H} NMR spectrum of (IDipp)P(XyNC)Mn(CO)$_3$ (202.5 MHz, CD_2Cl_2, 298 K).

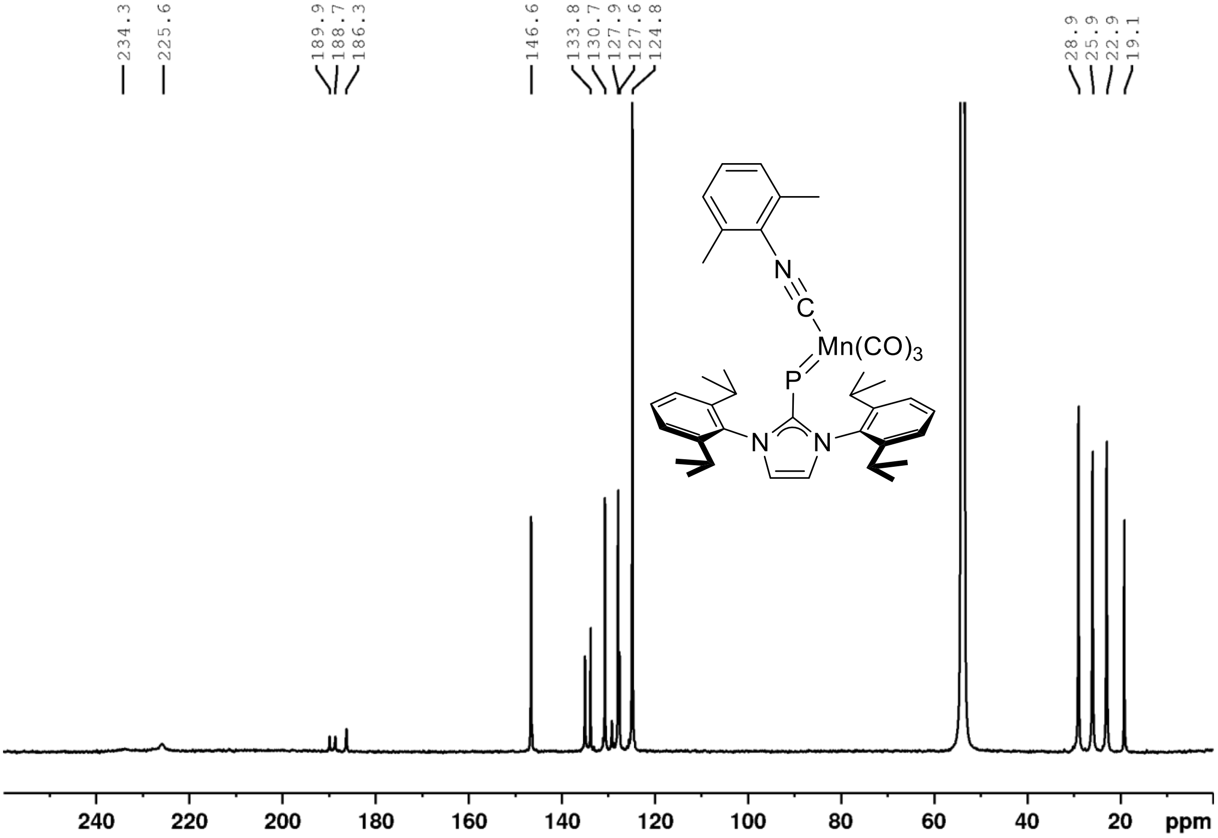

Figure S55: $^{13}C\{^{1}H\}$ NMR spectrum of (IDipp)P(XyNC)Mn(CO)$_3$ (101 MHz, CD_2Cl_2, 298 K, overview).

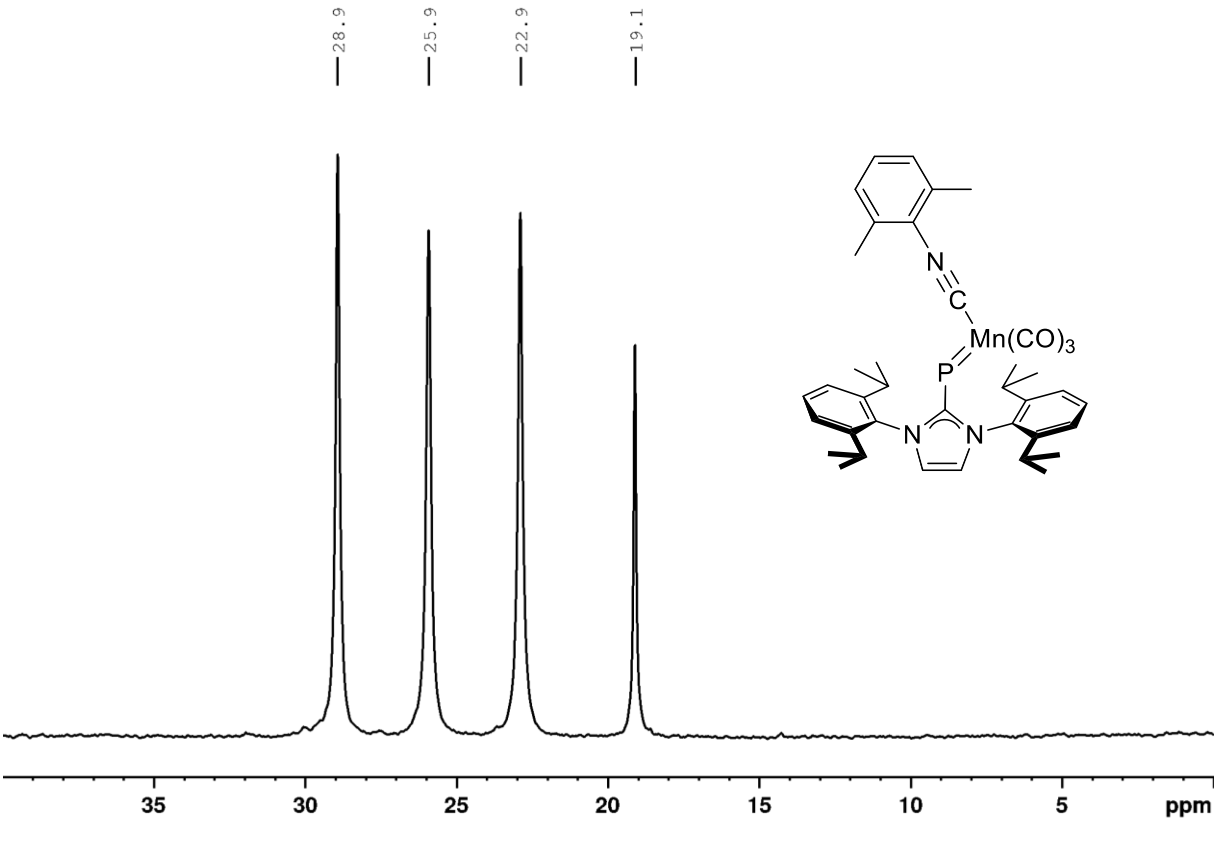

Figure S56: $^{13}C\{^{1}H\}$ NMR spectrum of (IDipp)P(XyNC)Mn(CO)$_3$ (101 MHz, CD_2Cl_2, 298 K, 0–40 ppm).

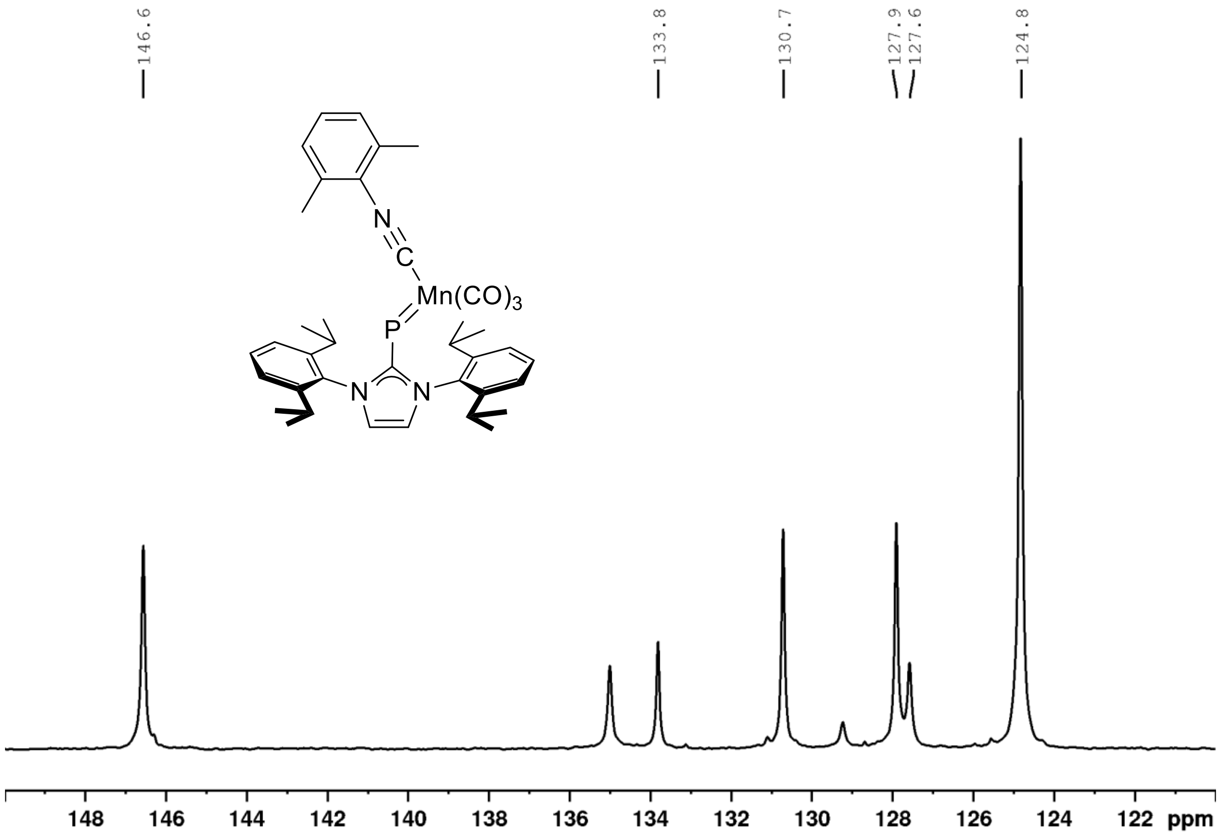

Figure S57: $^{13}C\{^{1}H\}$ NMR spectrum of (IDipp)P(XyNC)Mn(CO)$_3$ (101 MHz, CD_2Cl_2, 298 K, 120–150 ppm).

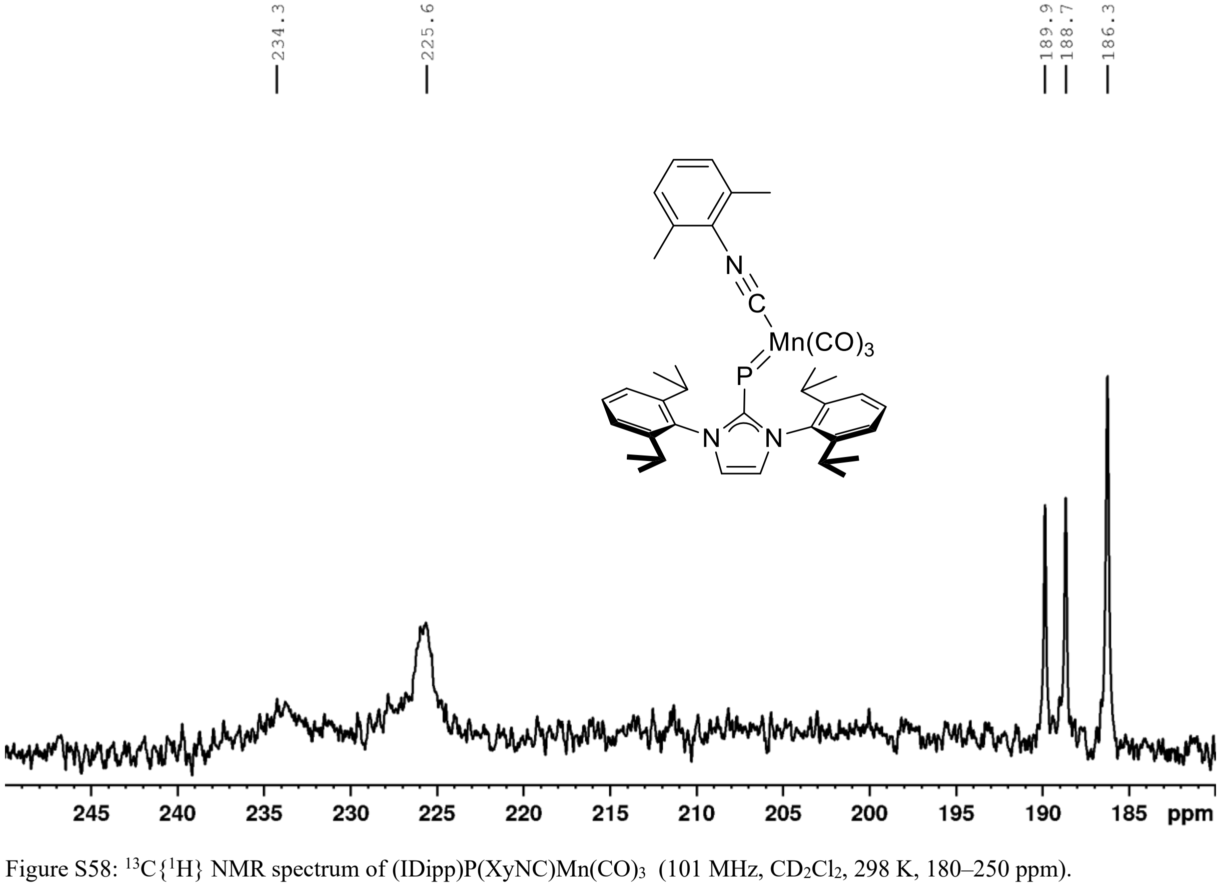

Figure S58: $^{13}C\{^{1}H\}$ NMR spectrum of (IDipp)P(XyNC)Mn(CO)$_3$ (101 MHz, CD_2Cl_2, 298 K, 180–250 ppm).

S3.6. {(IDipp)P=PH}Mn(CO)4

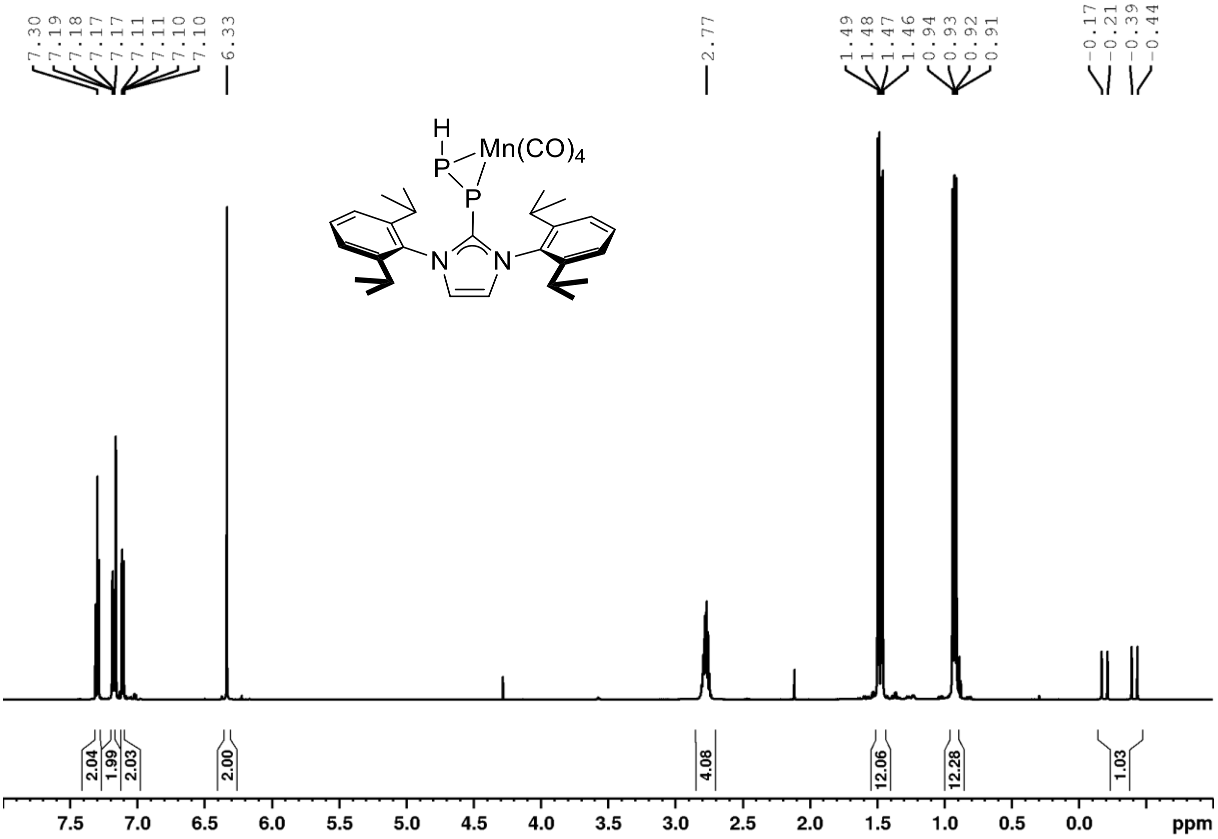

Figure S59: ^{1}H NMR spectrum of {(IDipp)P=PH}Mn(CO)4 (600 MHz, C_6D_6, 298 K, overview).

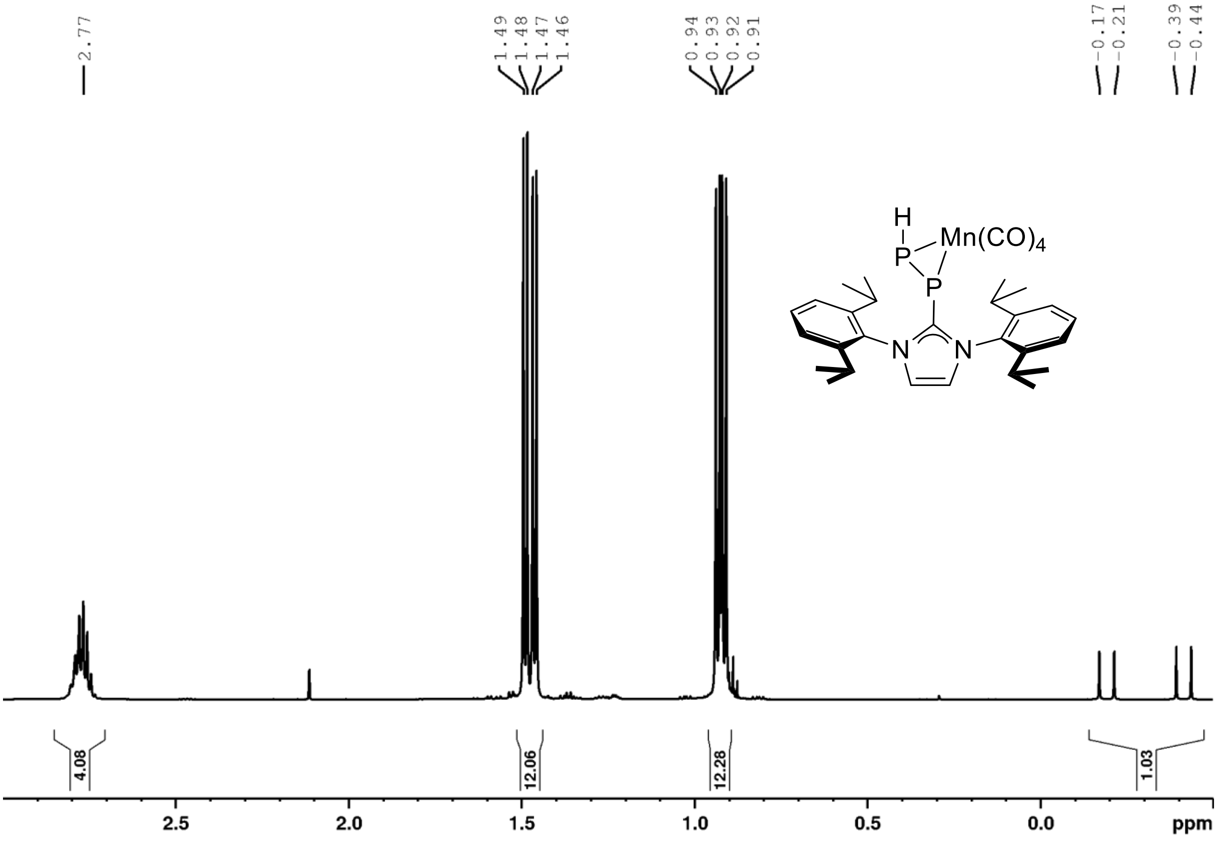

Figure S60: ^{1}H NMR spectrum of {(IDipp)P=PH}Mn(CO)4 (600 MHz, C_6D_6, 298 K, (–0.5)–3.0 ppm).

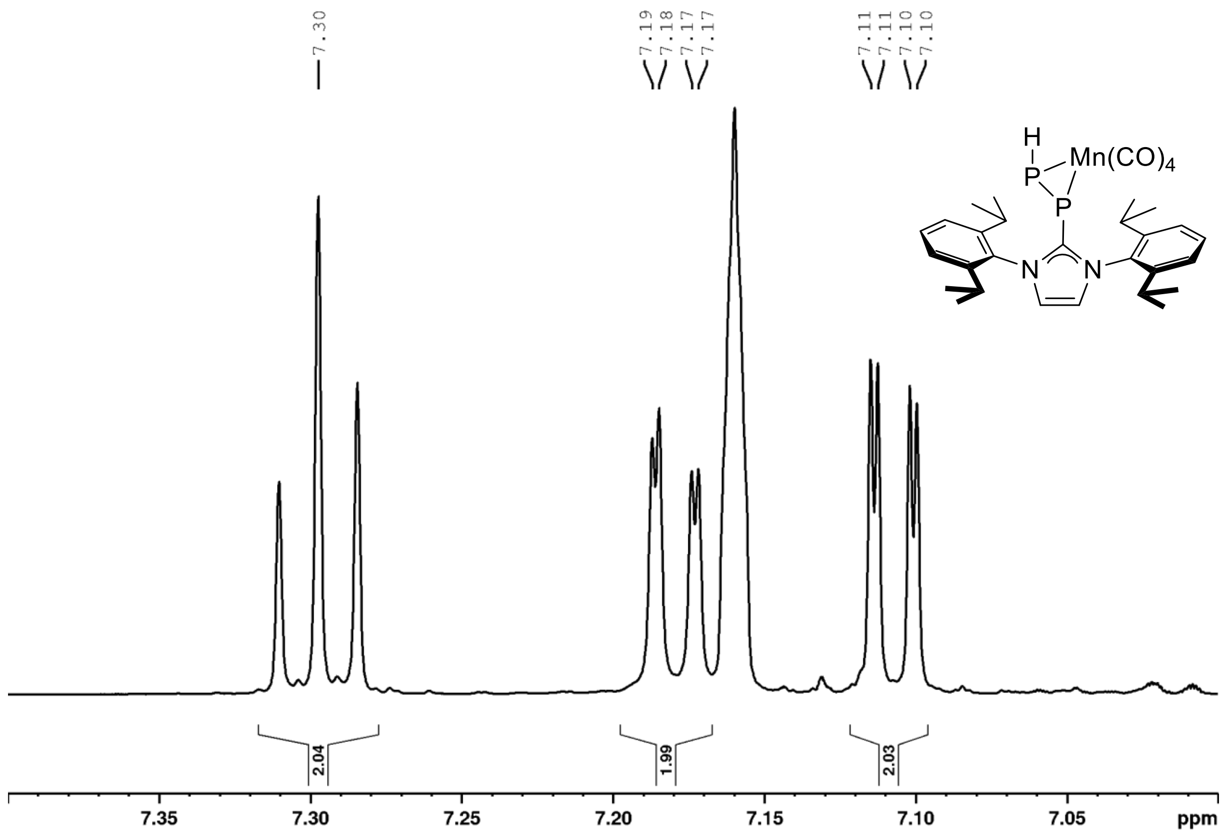

Figure S61: ^{1}H NMR spectrum of {(IDipp)P=PH}Mn(CO)$_4$ (600 MHz, C_6D_6, 298 K, 7.0–7.4 ppm).

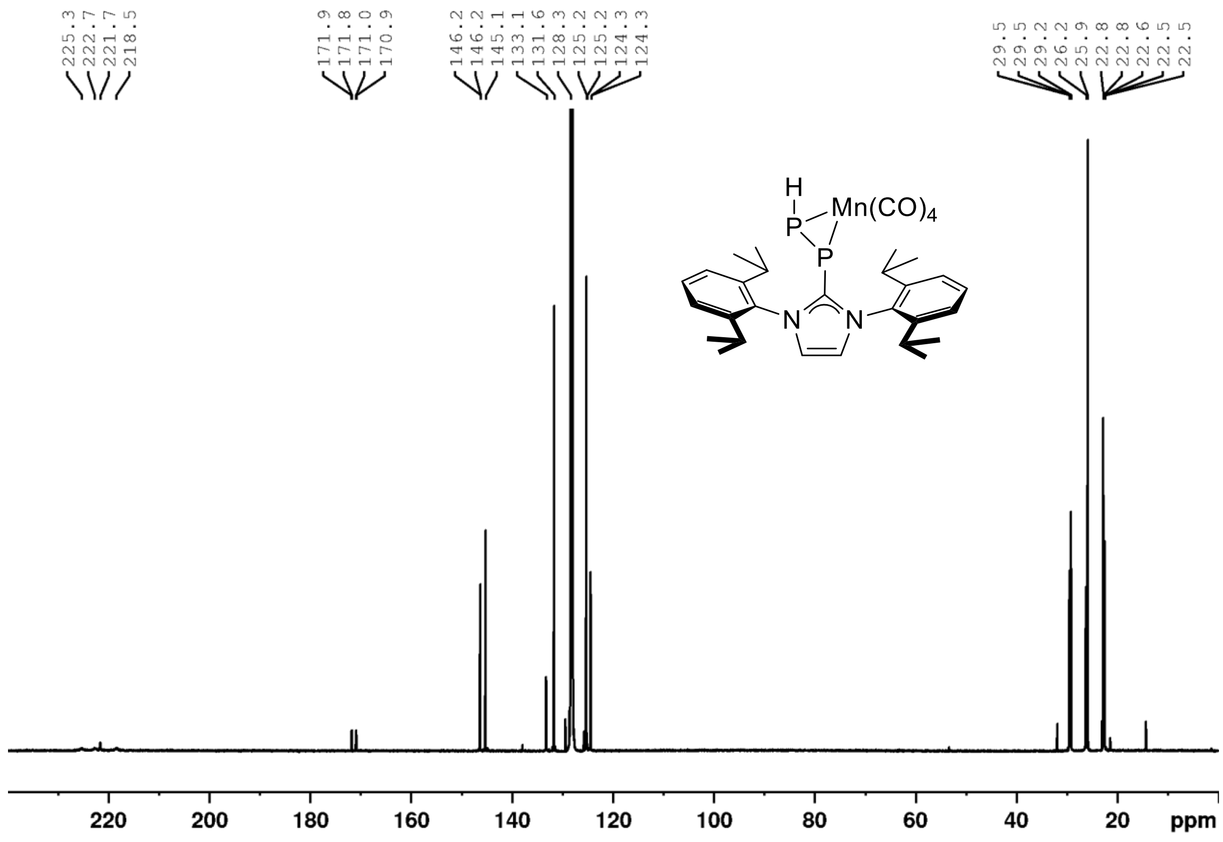

Figure S62: ^{13}C{^{1}H} NMR spectrum of {(IDipp)P=PH}Mn(CO)$_4$ (151 MHz, C_6D_6, 298 K, overview).

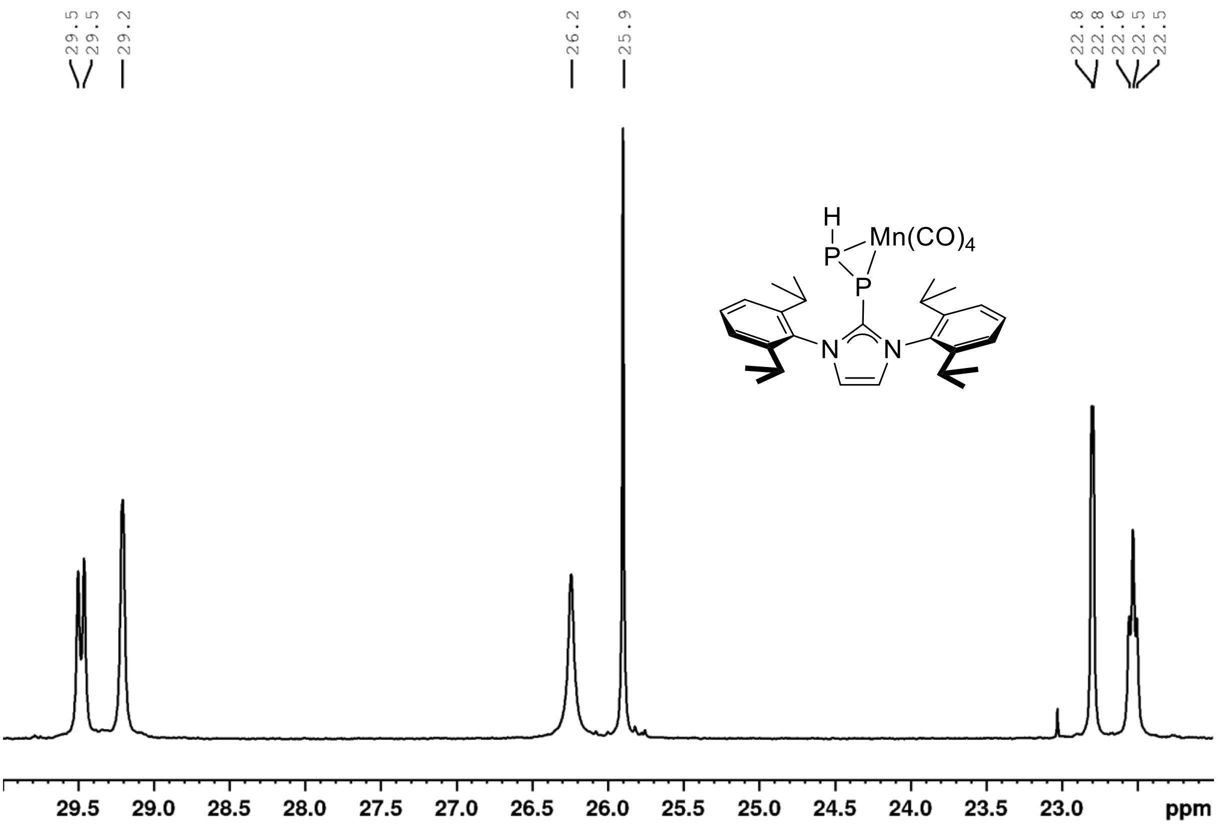

Figure S63: $^{13}C\{^{1}H\}$ NMR spectrum of $\{(IDipp)P{=}PH\}Mn(CO)_4$ (151 MHz, C_6D_6, 298 K, 22.0–30.0 ppm).

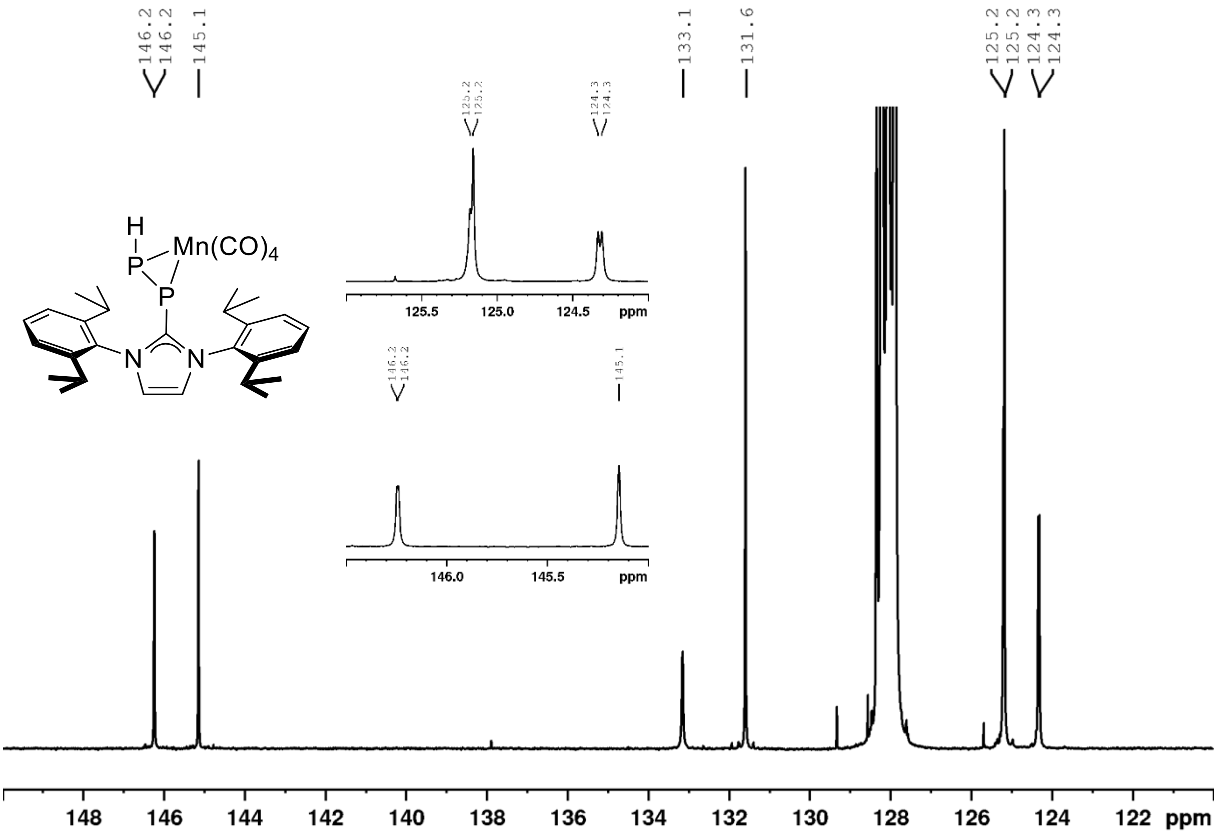

Figure S64: $^{13}C\{^{1}H\}$ NMR spectrum of $\{(IDipp)P{=}PH\}Mn(CO)_4$ (151 MHz, C_6D_6, 298 K, 120.0–150.0 ppm).

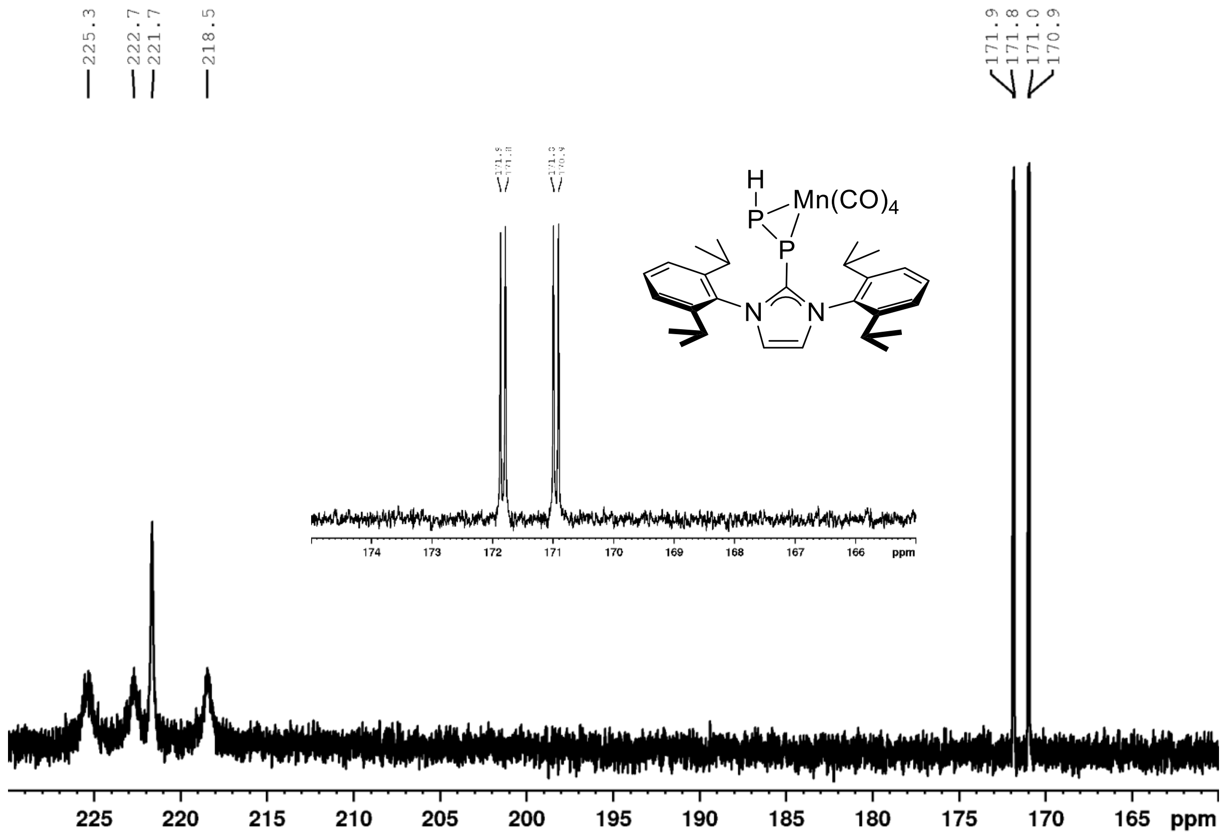

Figure S65: $^{13}C\{^{1}H\}$ NMR spectrum of {(IDipp)P=PH}Mn(CO)$_4$ (151 MHz, C_6D_6, 298 K, 160.0–240.0 ppm).

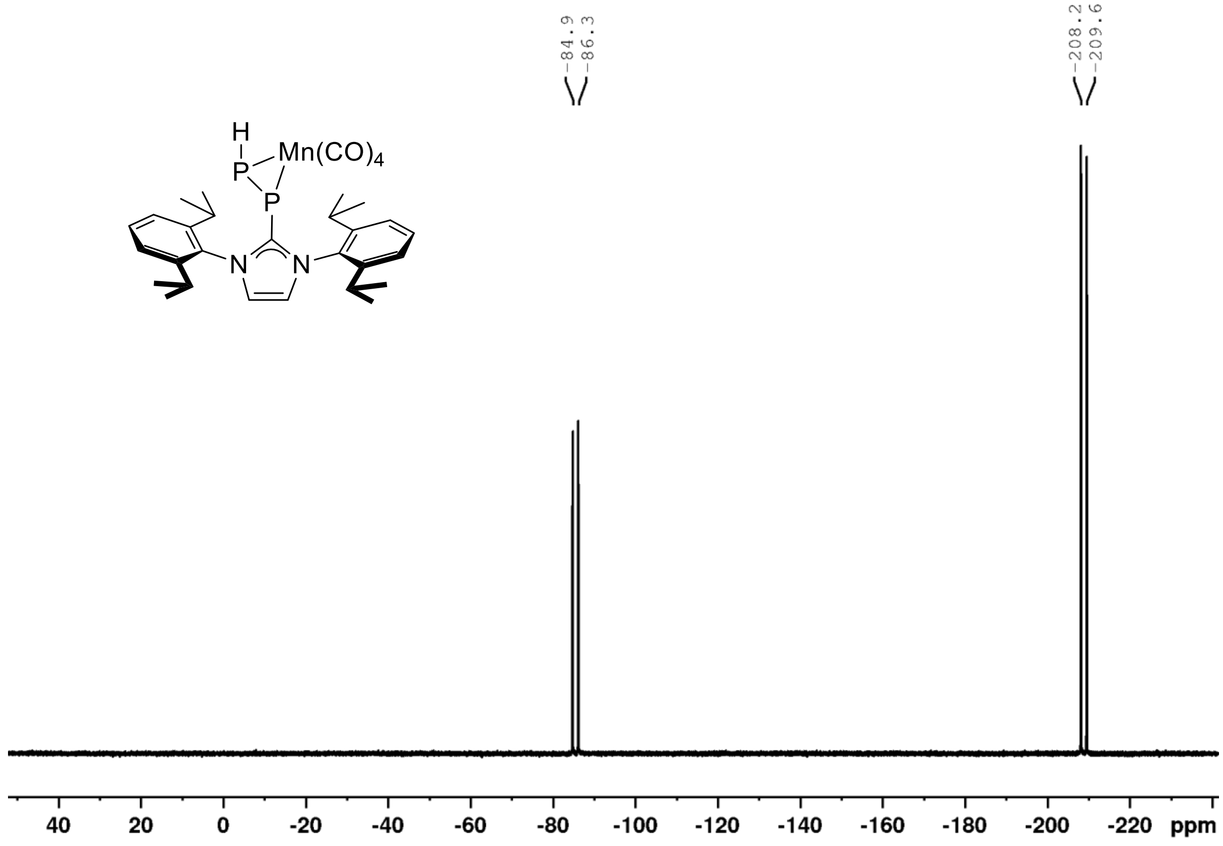

Figure S66: $^{31}P\{^{1}H\}$ NMR spectrum of {(IDipp)P=PH}Mn(CO)$_4$ (205.5 MHz, C_6D_6, 298 K).

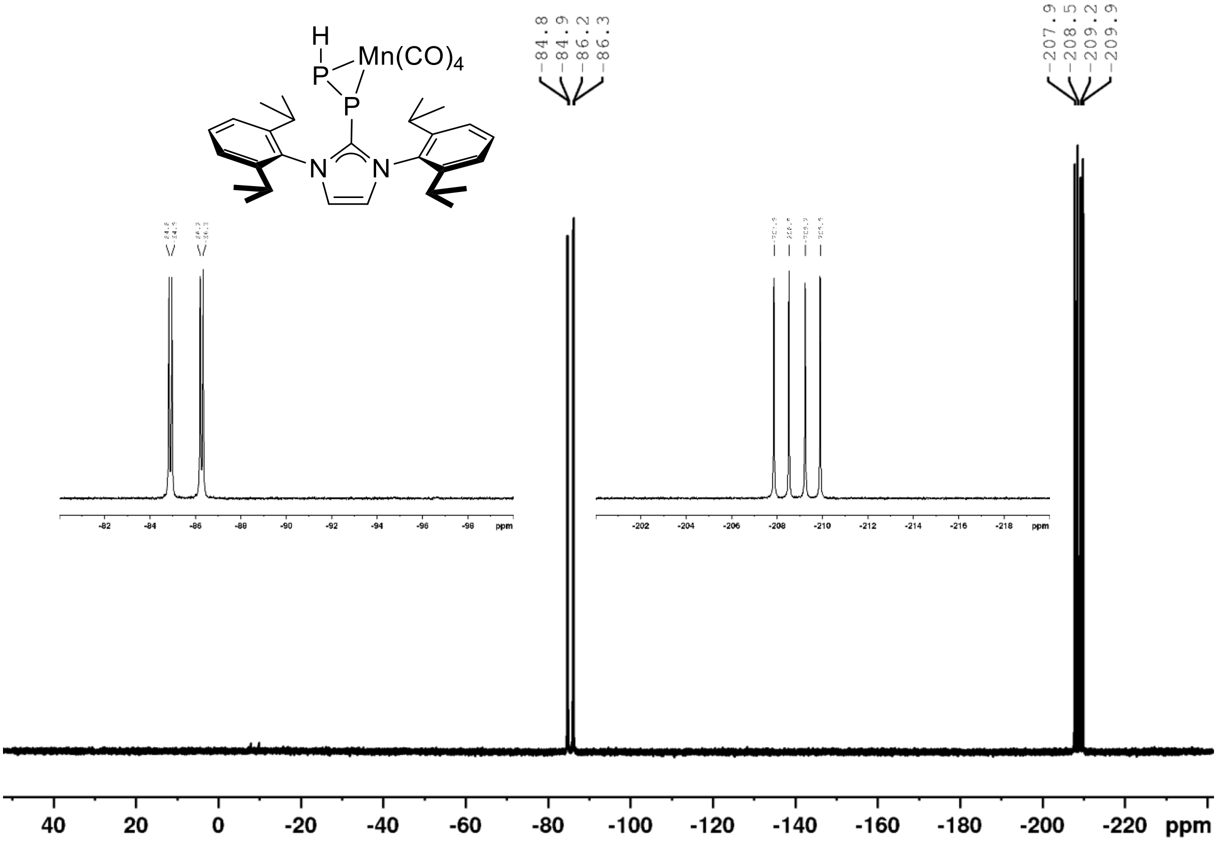

Figure S67: ^{31}P NMR spectrum of {(IDipp)P=PH}Mn(CO)$_4$ (205.5 MHz, C_6D_6, 298 K).

S3.7. {(IDipp)P=PPh}Mn(CO)$_4$

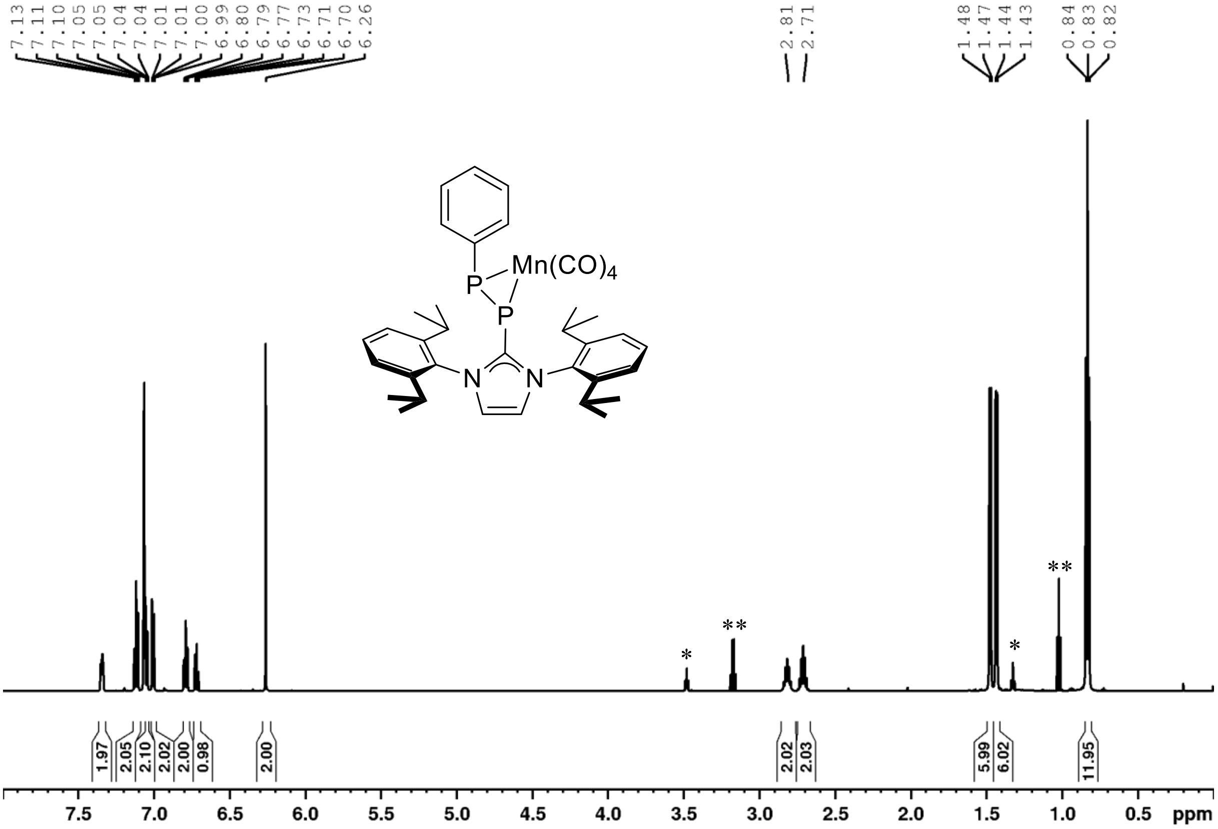

Figure S68: ^{1}H NMR spectrum of {(IDipp)P=PPh}Mn(CO)$_4$ (600 MHz, C_6D_6, 298 K, overview); * = residual THF, ** = residual Et_2O; both residual solvents could be removed by furth drying under high vacuum as can be seen by CHN.

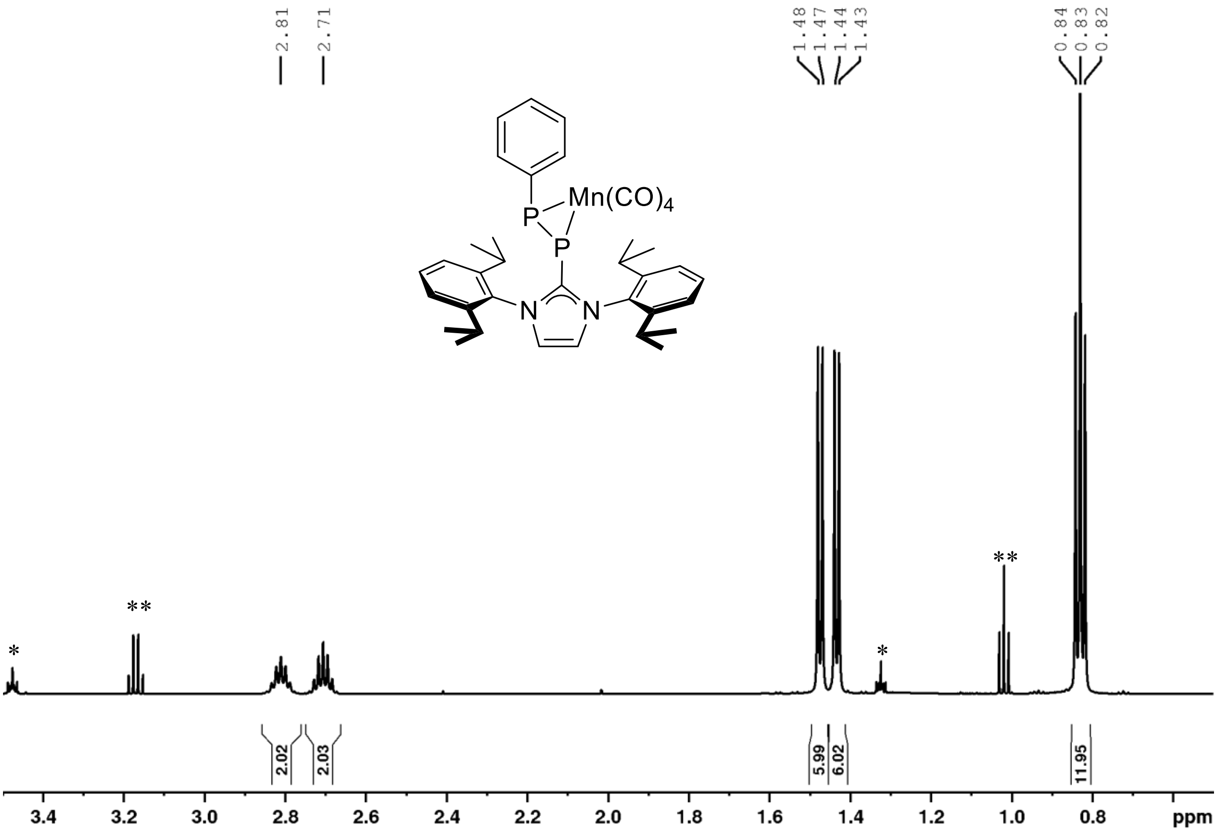

Figure S69: ^{1}H NMR spectrum of {(IDipp)P=PPh}Mn(CO)$_4$ (600 MHz, C_6D_6, 298 K, 0.5–3.5 ppm); * = residual THF, ** = residual Et_2O; both residual solvents could be removed by furth drying under lhigh vacuum as can be seen by CHN.

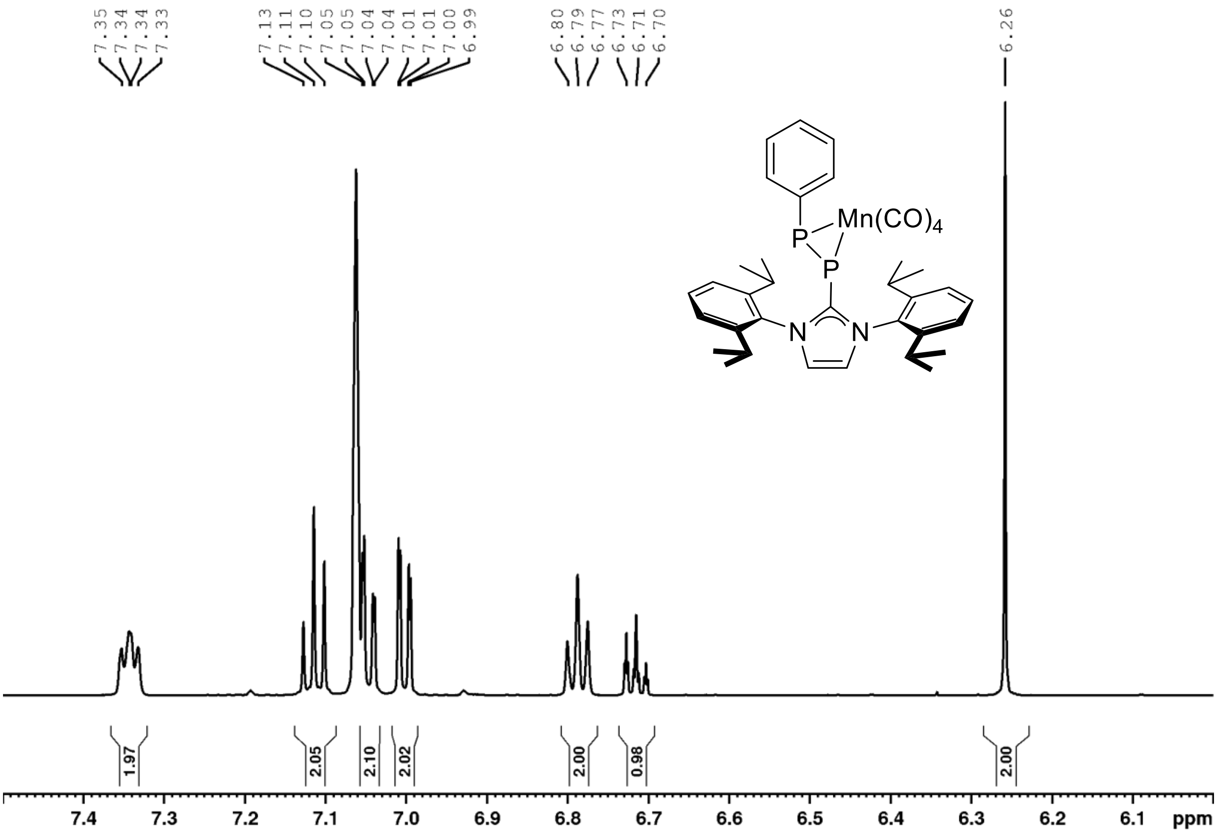

Figure S70: ^{1}H NMR spectrum of {(IDipp)P=PPh}Mn(CO)4 (600 MHz, C_6D_6, 298 K, 6.0–7.5 ppm).

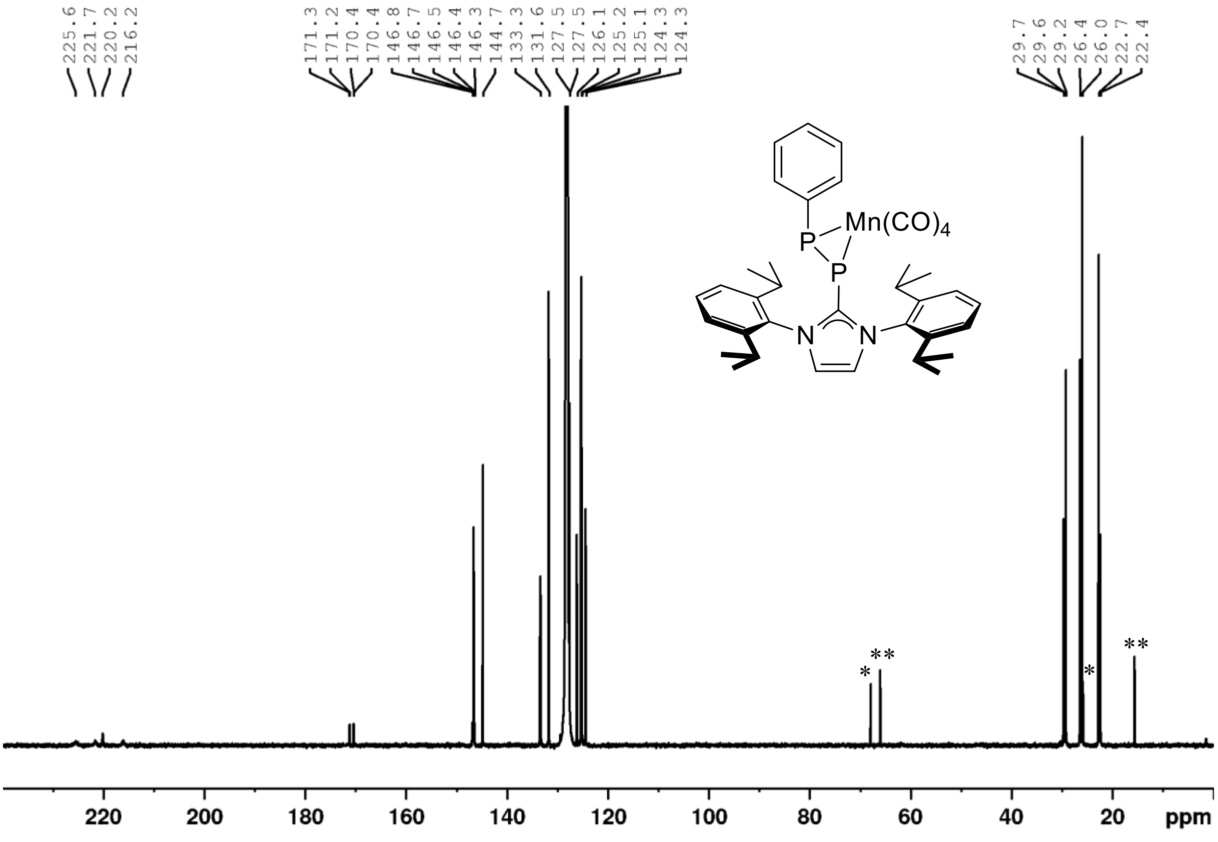

Figure S71: $^{13}C\{^1H\}$ NMR spectrum of {(IDipp)P=PPh}Mn(CO)4 (151 MHz, C_6D_6, 298 K, overview);* = residual THF, ** = residual Et_2O; both residual solvents could be removed by furth drying under lhigh vacuum as can be seen by CHN.

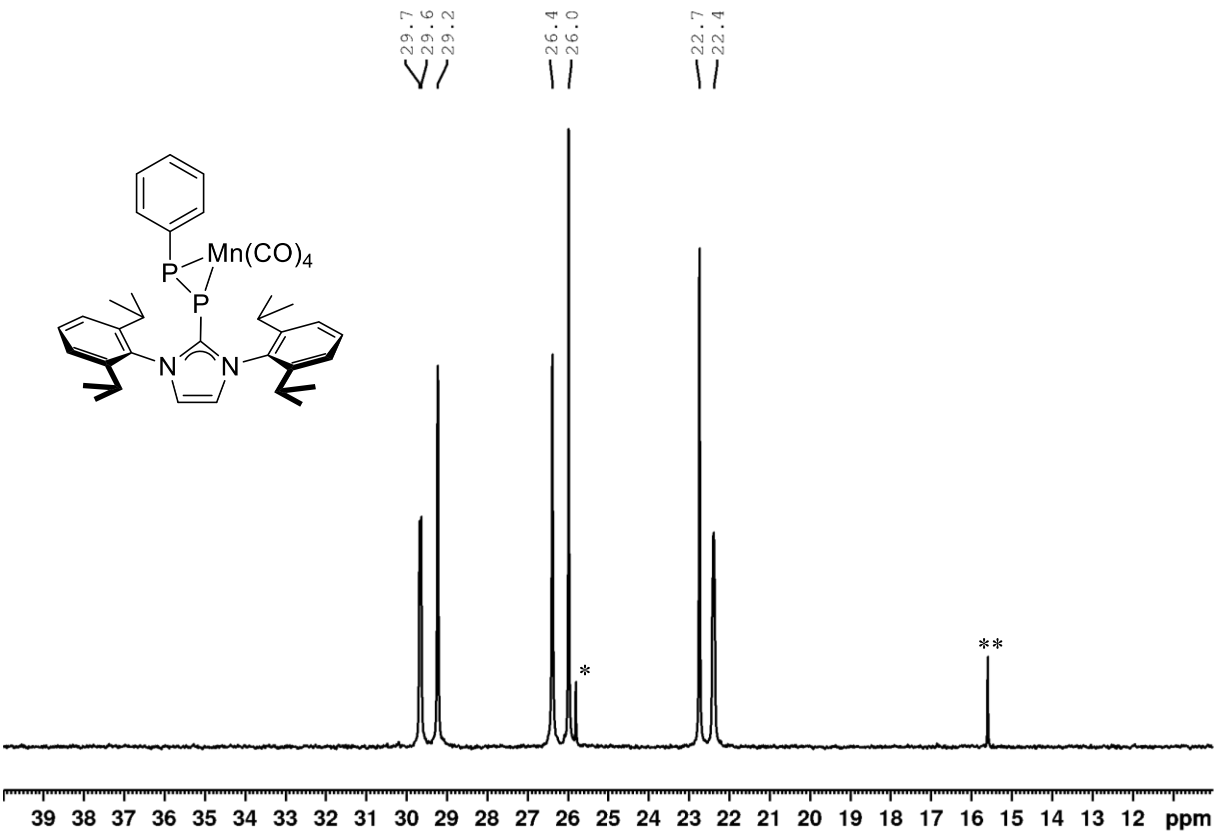

Figure S72: $^{13}C\{^{1}H\}$ NMR spectrum of {(IDipp)P=PPh}Mn$(CO)_4$ (151 MHz, C_6D_6, 298 K, 10–40 ppm);* = residual THF, ** = residual Et_2O; both residual solvents could be removed by furth drying under lhigh vacuum as can be seen by CHN.

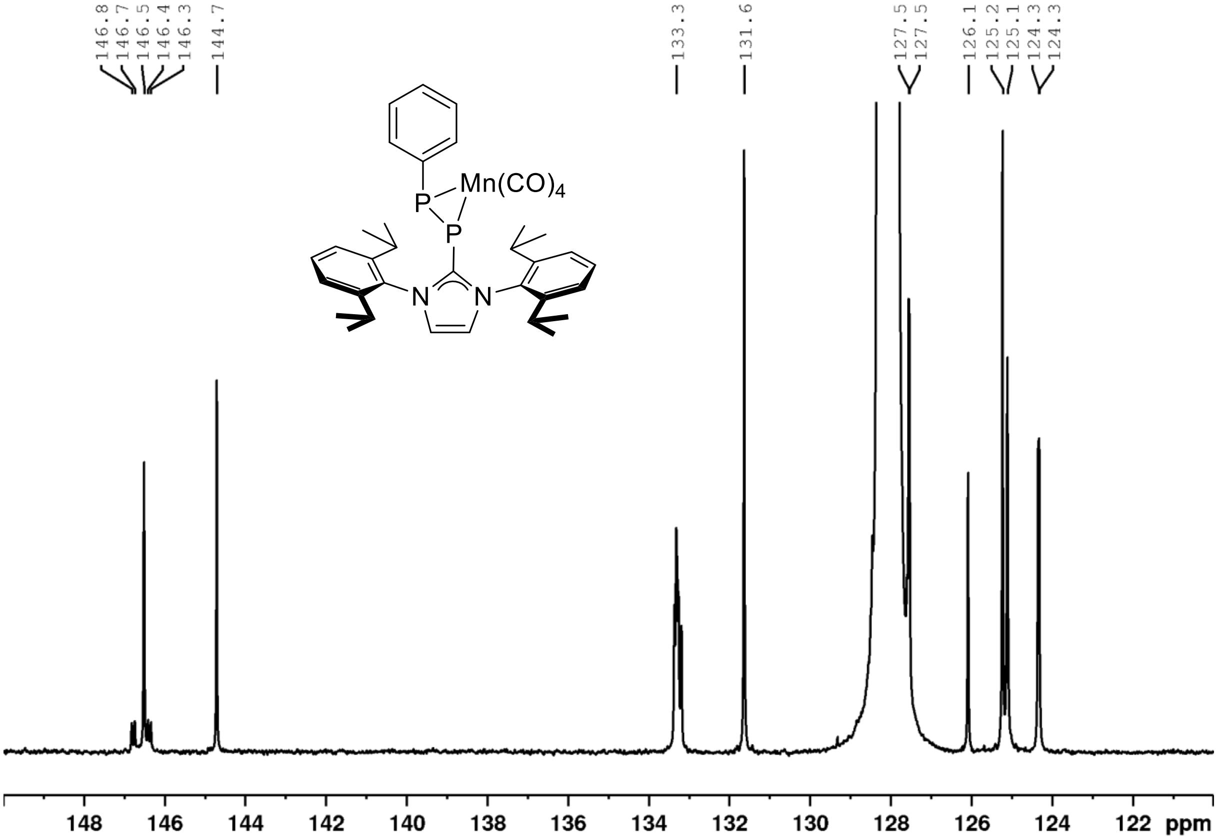

Figure S73: $^{13}C\{^{1}H\}$ NMR spectrum of {(IDipp)P=PPh}Mn$(CO)_4$ (151 MHz, C_6D_6, 298 K, 120–150 ppm).

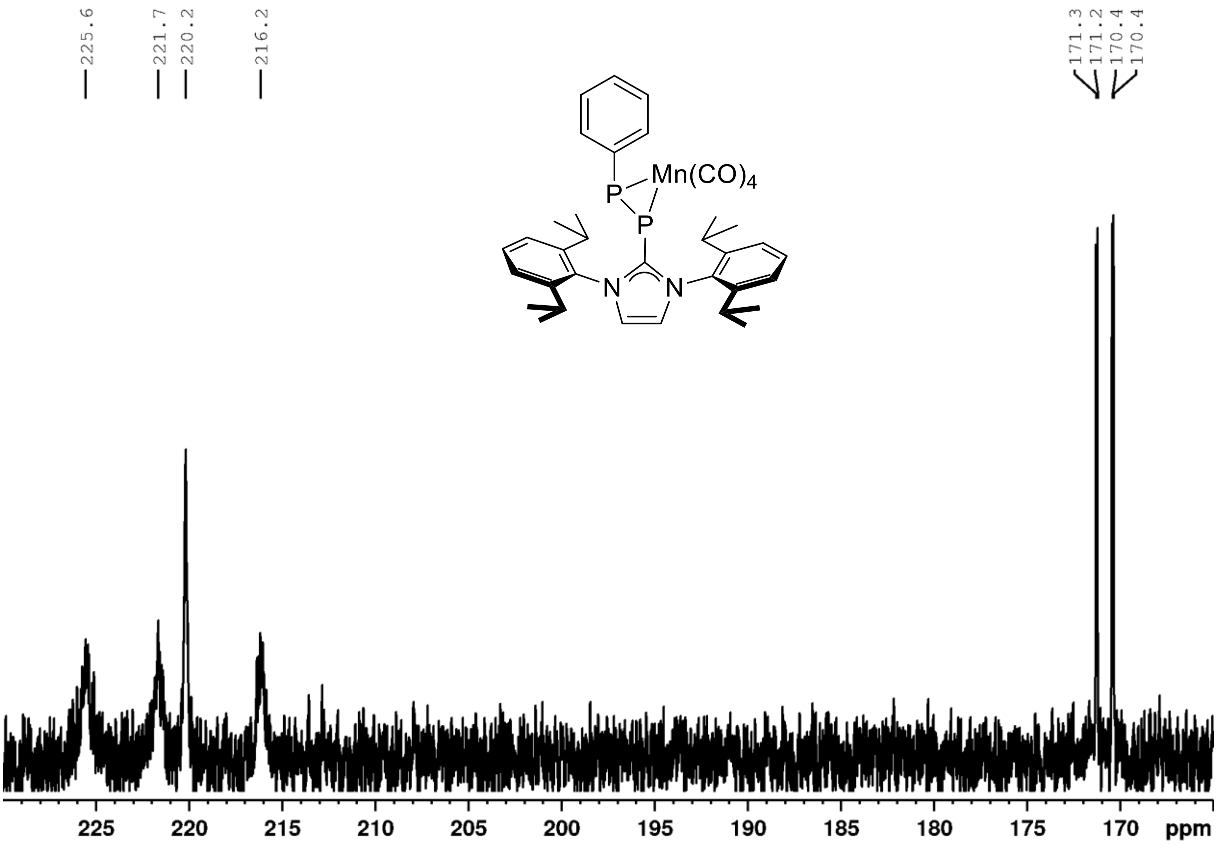

Figure S74: $^{13}C\{^{1}H\}$ NMR spectrum of {(IDipp)P=PPh}Mn(CO)$_4$ (151 MHz, C_6D_6, 298 K, 165–230 ppm).

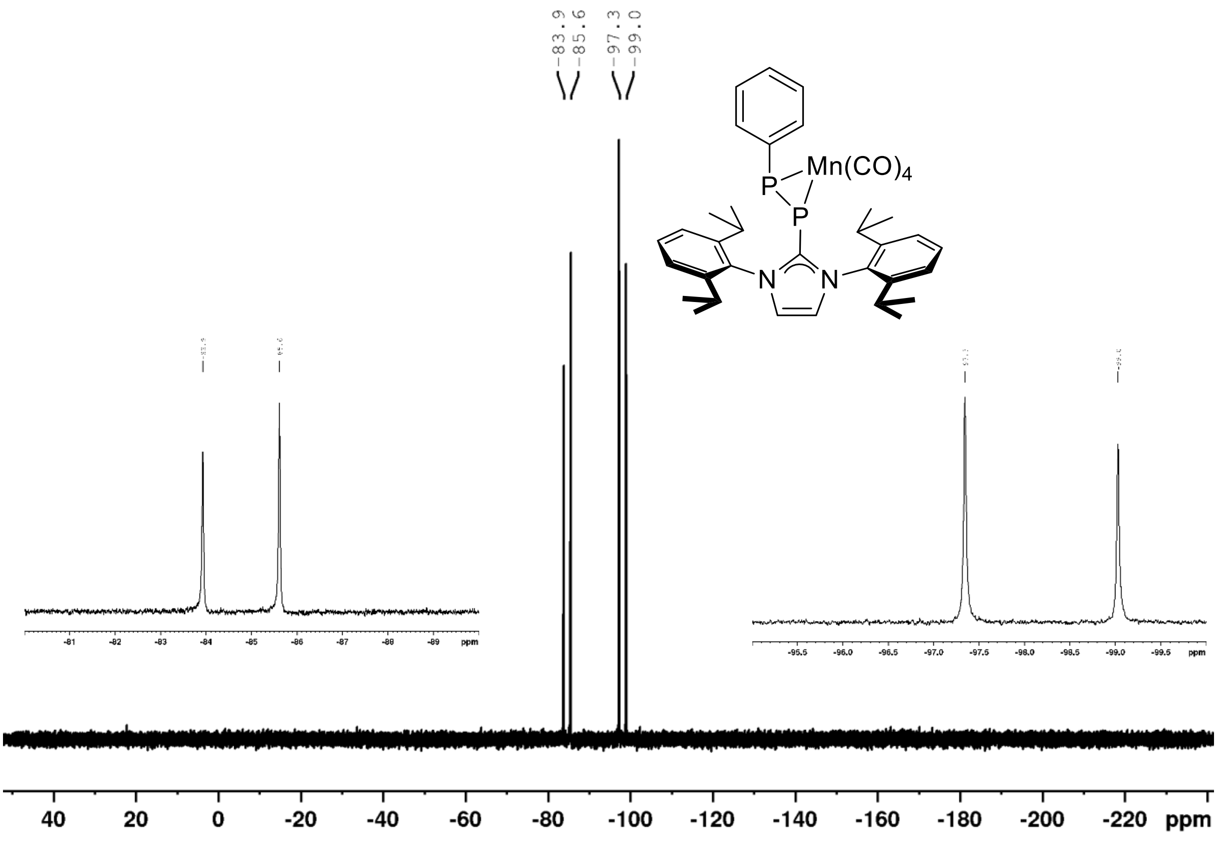

Figure S75: $^{31}P\{^{1}H\}$ NMR spectrum of {(IDipp)P=PPh}Mn(CO)$_4$ (205.5 MHz, C_6D_6, 298 K).

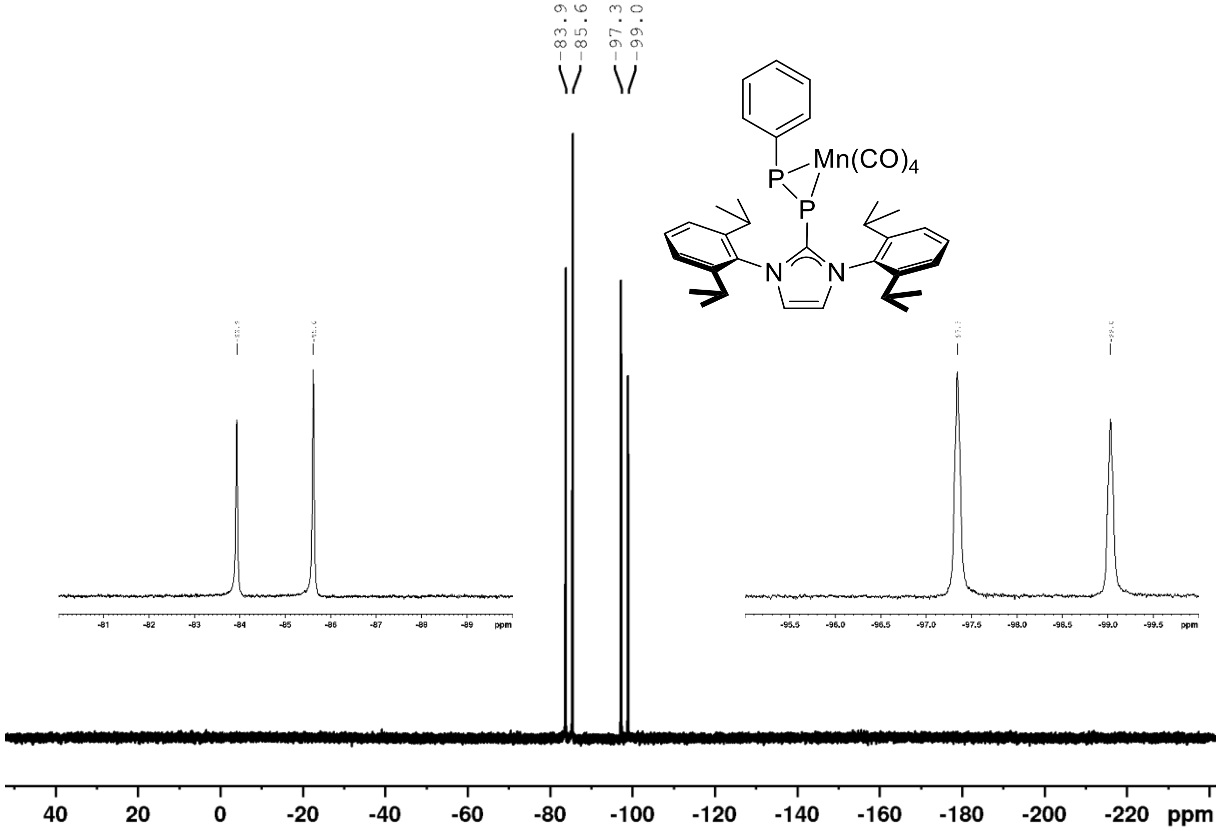

Figure S76: ^{31}P NMR spectrum of {(IDipp)P=PPh}Mn(CO)$_4$ (205.5 MHz, C_6D_6, 298 K).

S3.8. {(IDipp)P=Se}Mn(CO)4

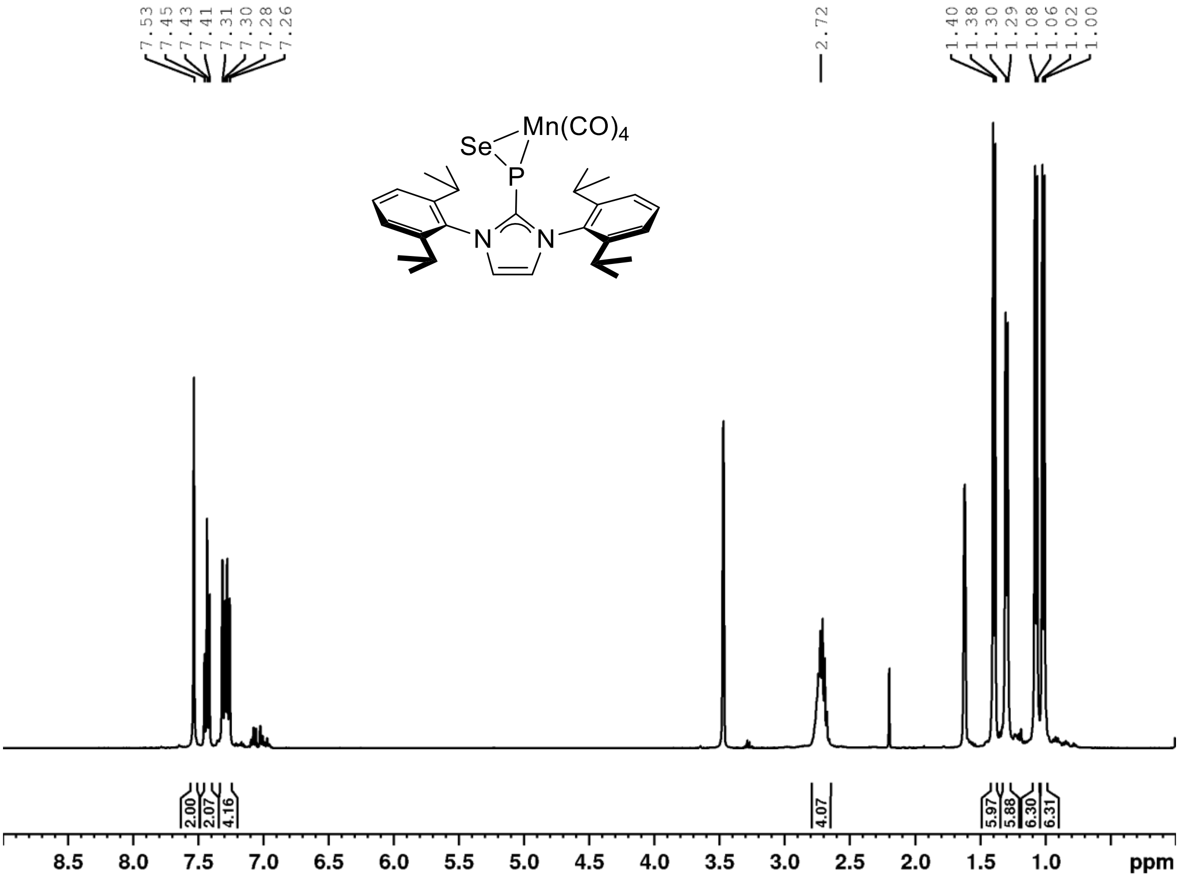

Figure S77: ^{1}H NMR spectrum of {(IDipp)P=Se}Mn(CO)4 (400 MHz, THF-d8, 298 K, overview).

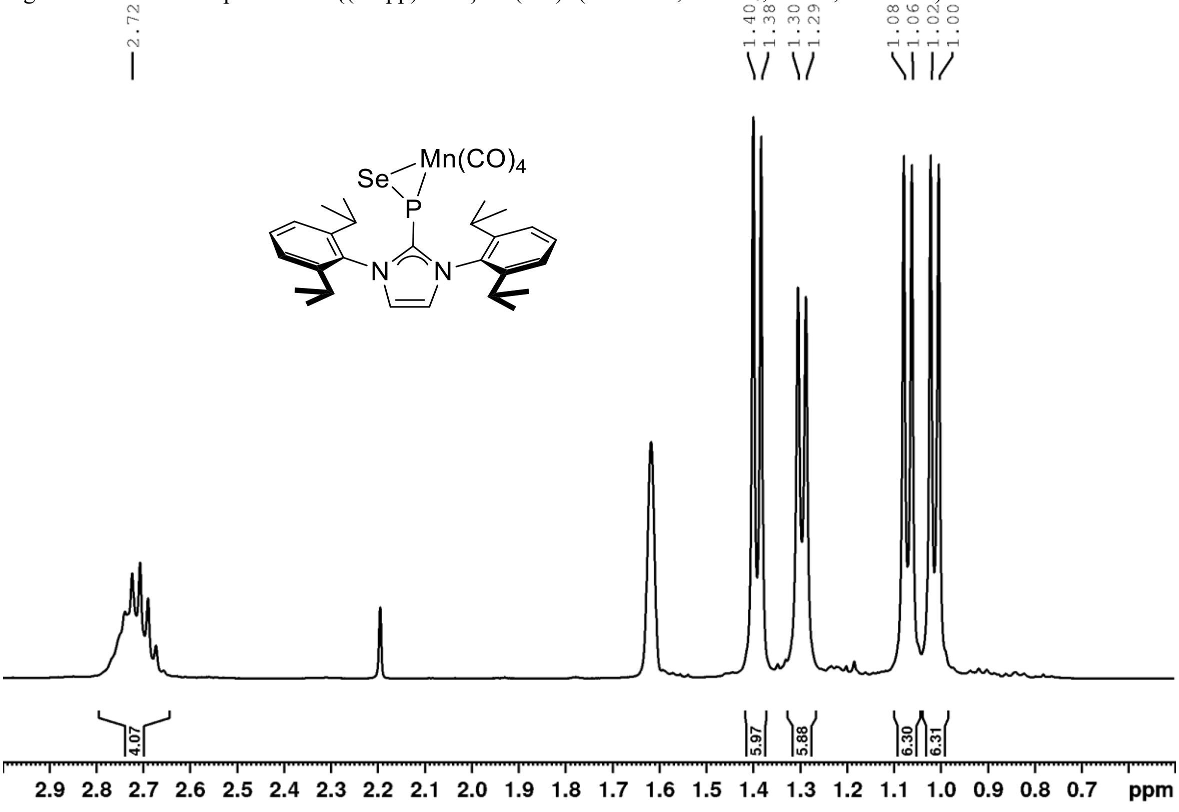

Figure S78: ^{1}H NMR spectrum of {(IDipp)P=Se}Mn(CO)4 (400 MHz, THF-d8, 298 K, 0.5–3.0 ppm).

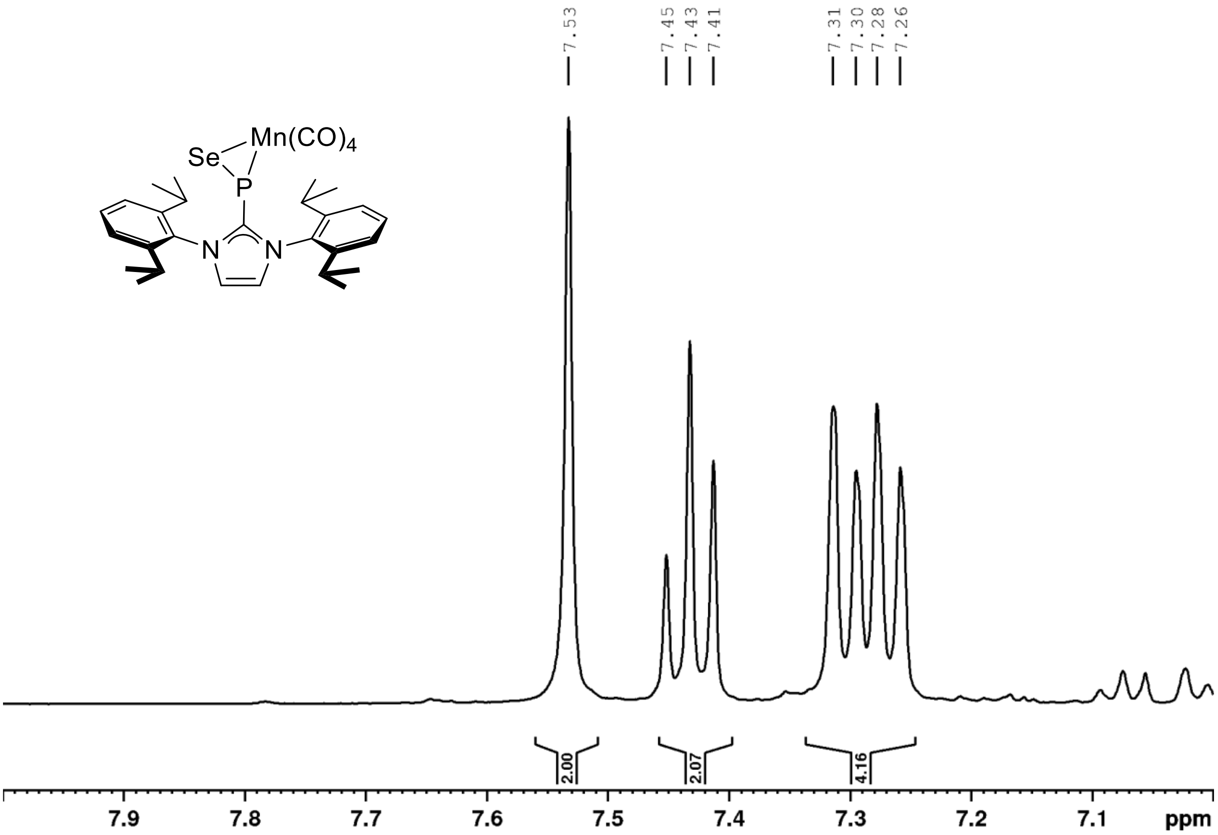

Figure S79: ^{1}H NMR spectrum of {(IDipp)P=Se}Mn(CO)$_4$ (400 MHz, THF-d$_8$, 298 K, 7.0–8.0 ppm).

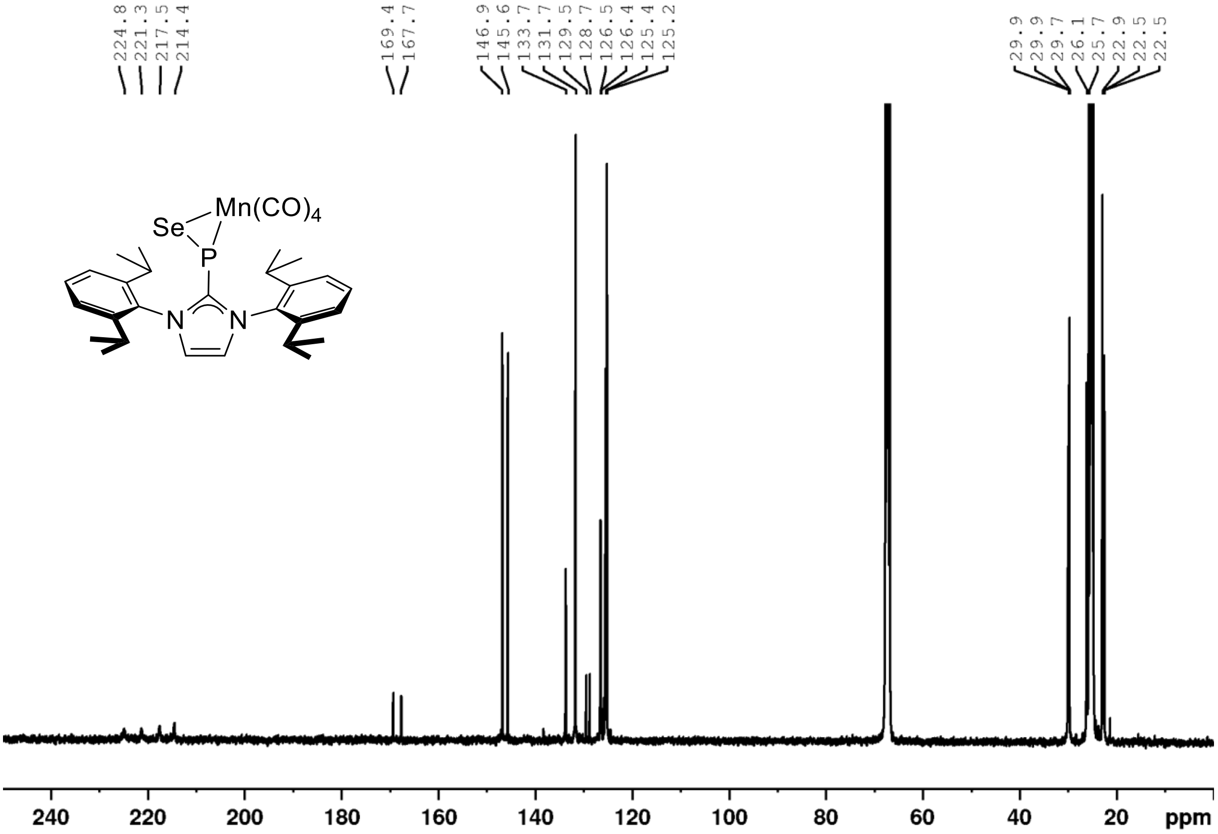

Figure S80: ^{13}C{^{1}H} NMR spectrum of {(IDipp)P=Se}Mn(CO)$_4$ (101 MHz, THF-d$_8$, 298 K, overview).

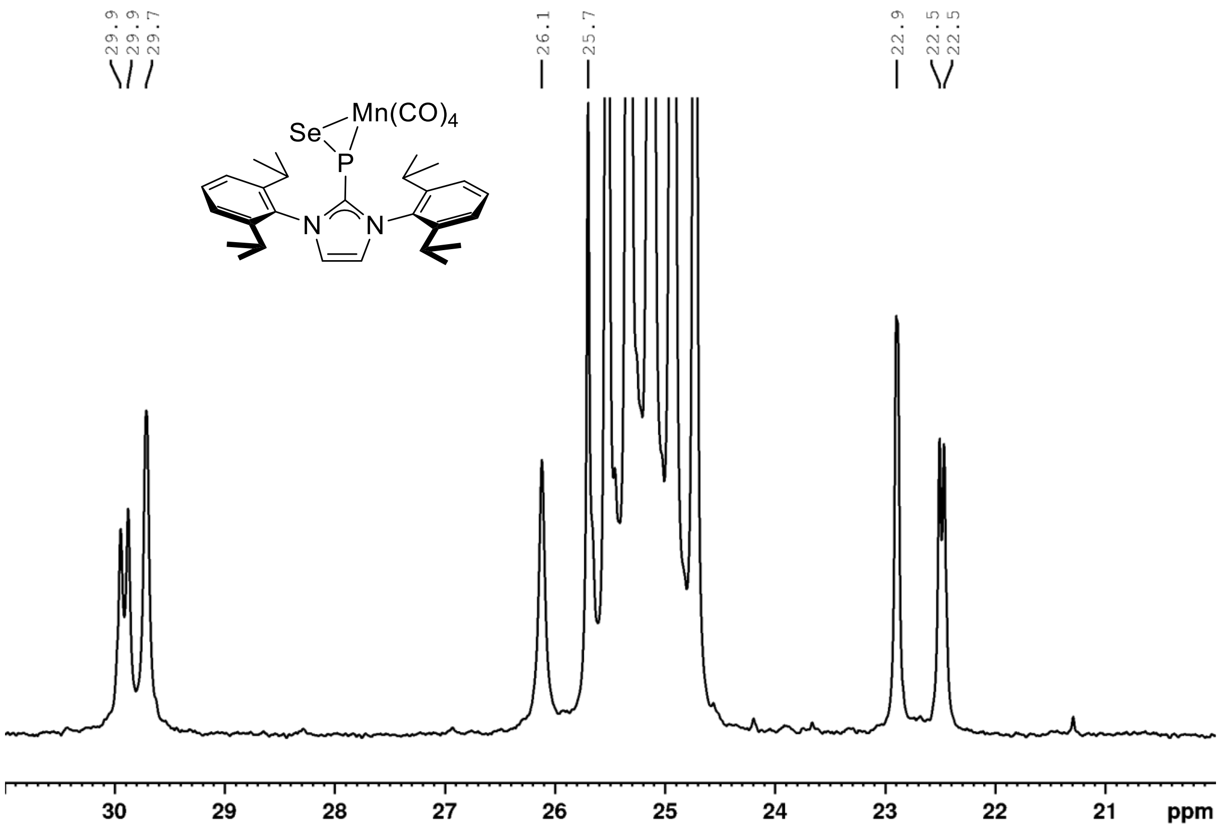

Figure S81: $^{13}C\{^{1}H\}$ NMR spectrum of {(IDipp)P=Se}Mn(CO)$_4$ (101 MHz, THF-d$_8$, 298 K, 20–31 ppm).

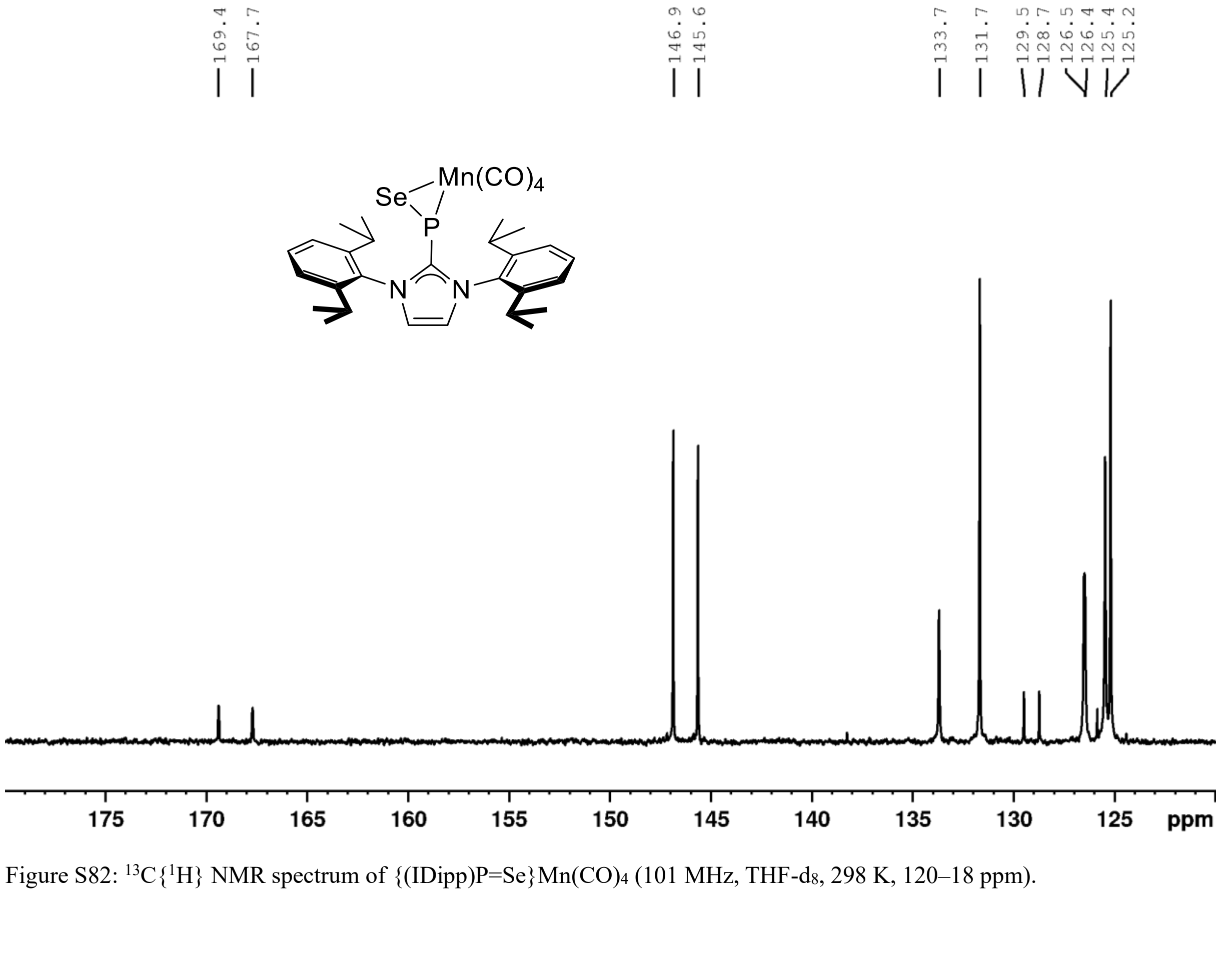

Figure S82: $^{13}C\{^{1}H\}$ NMR spectrum of {(IDipp)P=Se}Mn(CO)$_4$ (101 MHz, THF-d$_8$, 298 K, 120–18 ppm).

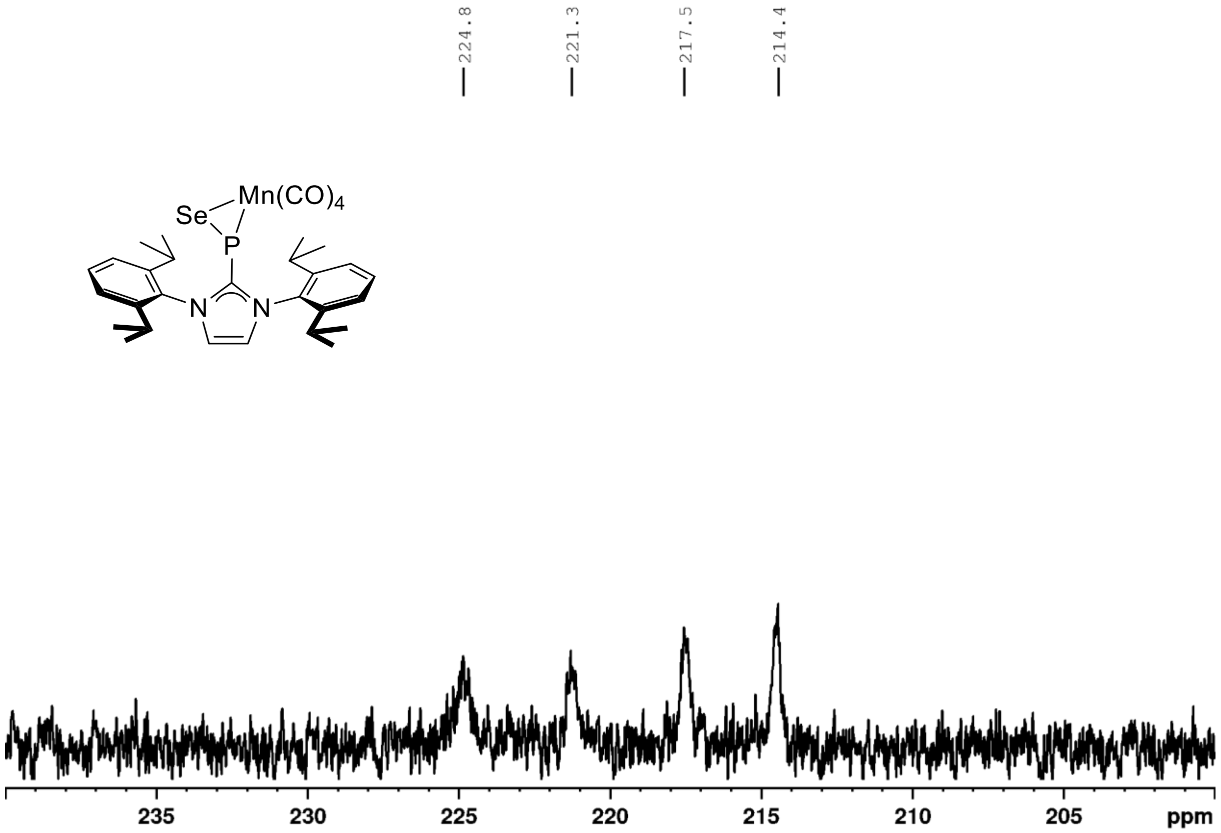

Figure S83: $^{13}C\{^{1}H\}$ NMR spectrum of {(IDipp)P=Se}$Mn(CO)_4$ (101 MHz, THF-d_8, 298 K, 200–240 ppm).

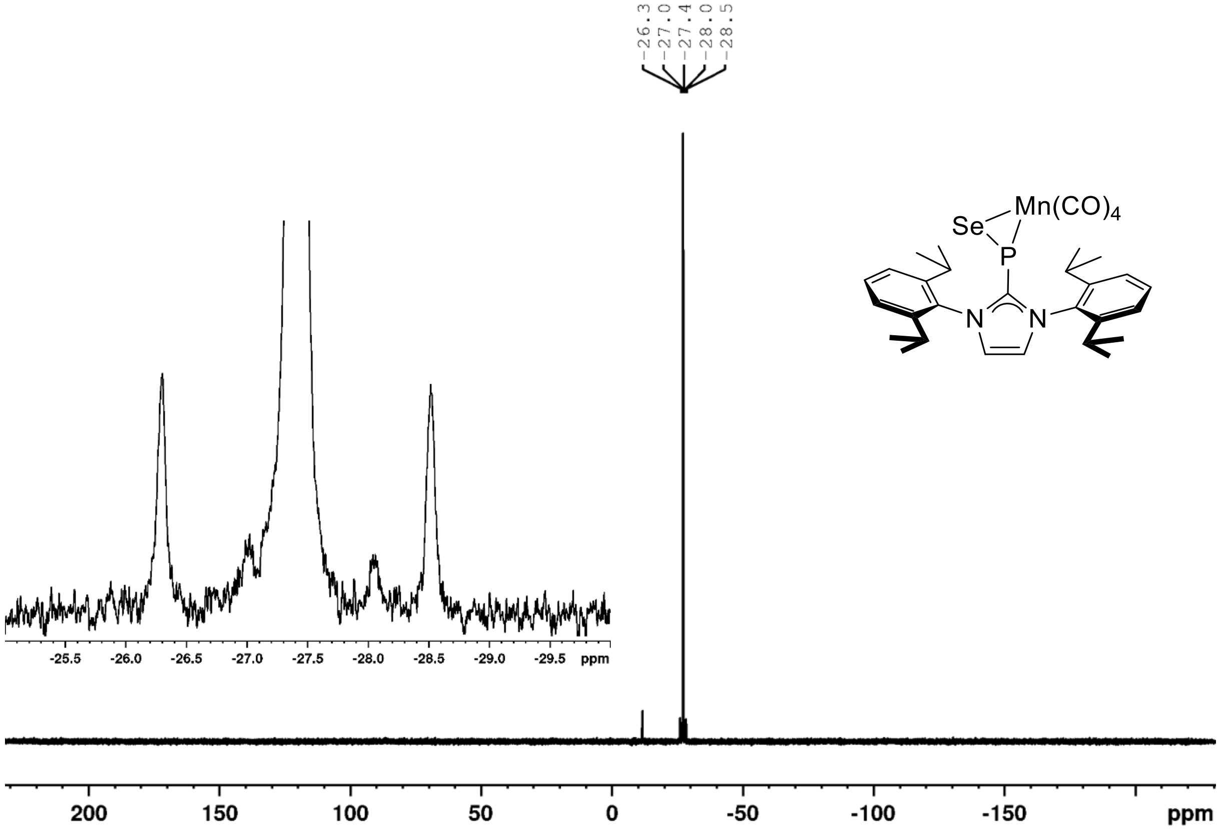

Figure S84: $^{31}P\{^{1}H\}$ NMR spectrum of {(IDipp)P=Se}$Mn(CO)_4$ (162 MHz, THF-d_8, 298 K).

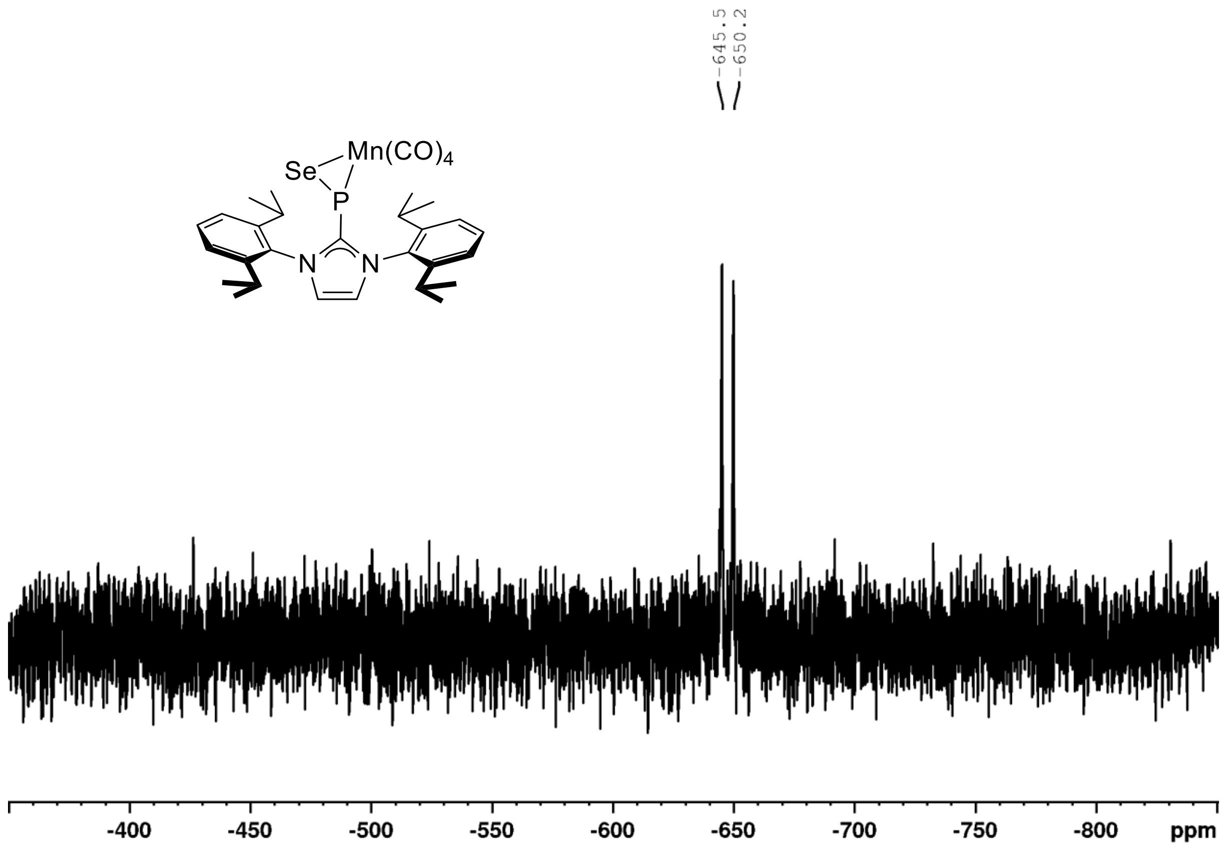

Figure S85: $^{77}Se\{^1H\}$ NMR spectrum of {(IDipp)P=Te}Mn(CO)$_4$ (76 MHz, THF-d_8, 298 K).

S3.9. {(IDipp)P=Te}Mn(CO)4

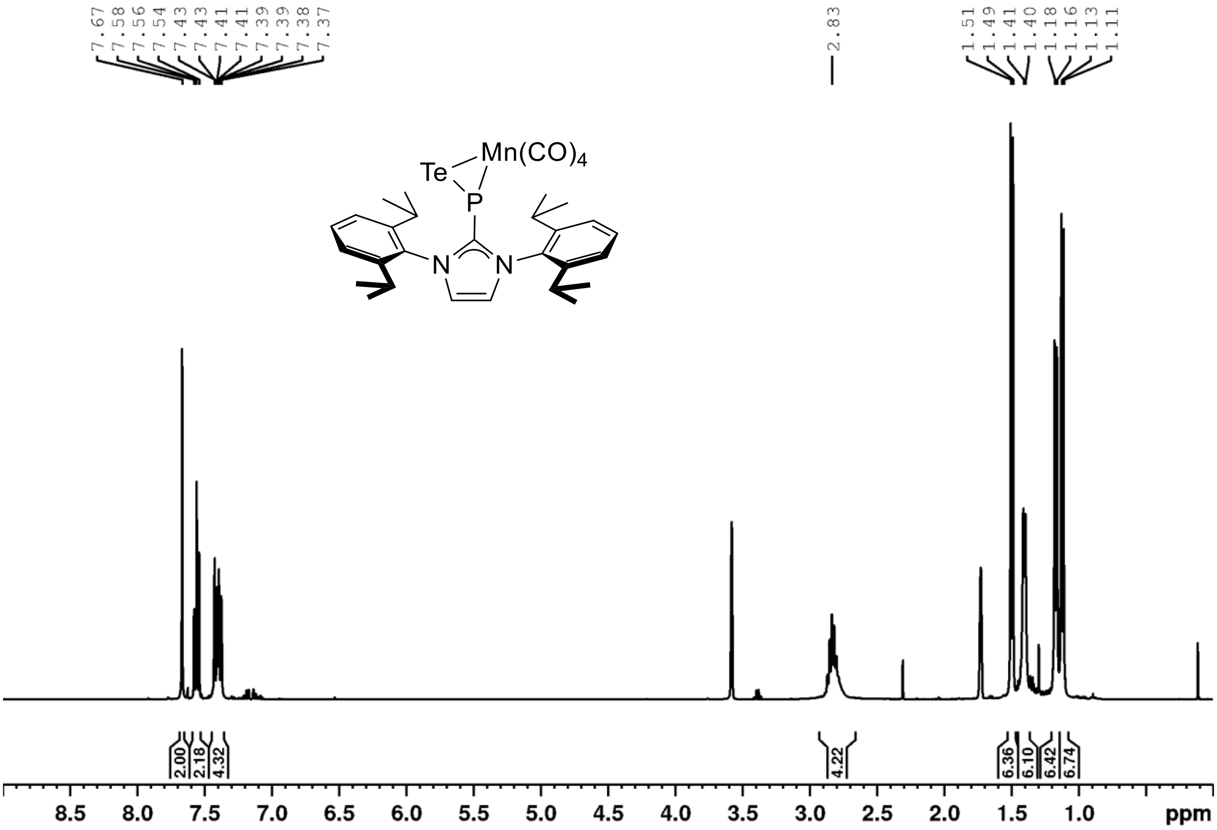

Figure S86: ^{1}H NMR spectrum of {(IDipp)P=Te}Mn(CO)4 (400 MHz, THF-d8, 298 K, overview).

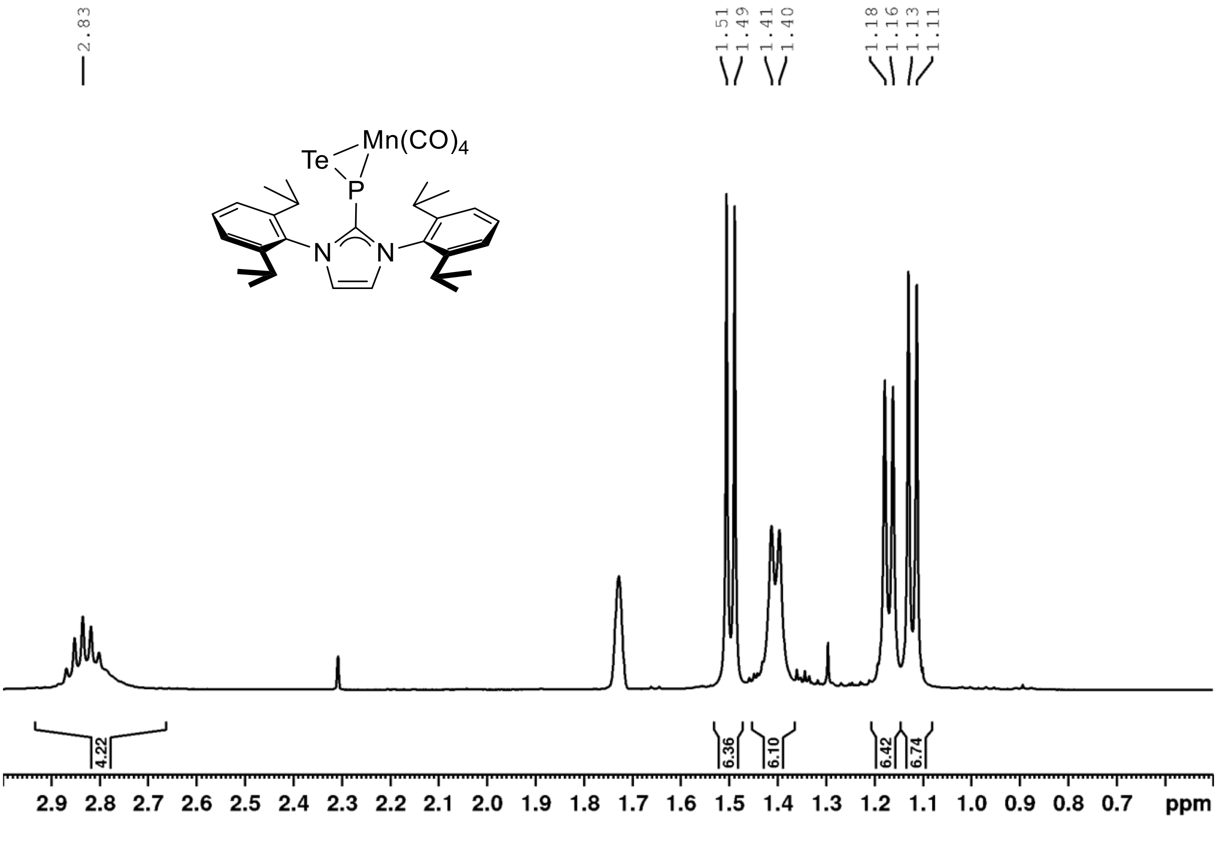

Figure S87: ^{1}H NMR spectrum of {(IDipp)P=Te}Mn(CO)4 (400 MHz, THF-d8, 298 K, 0.5–3.0 ppm).

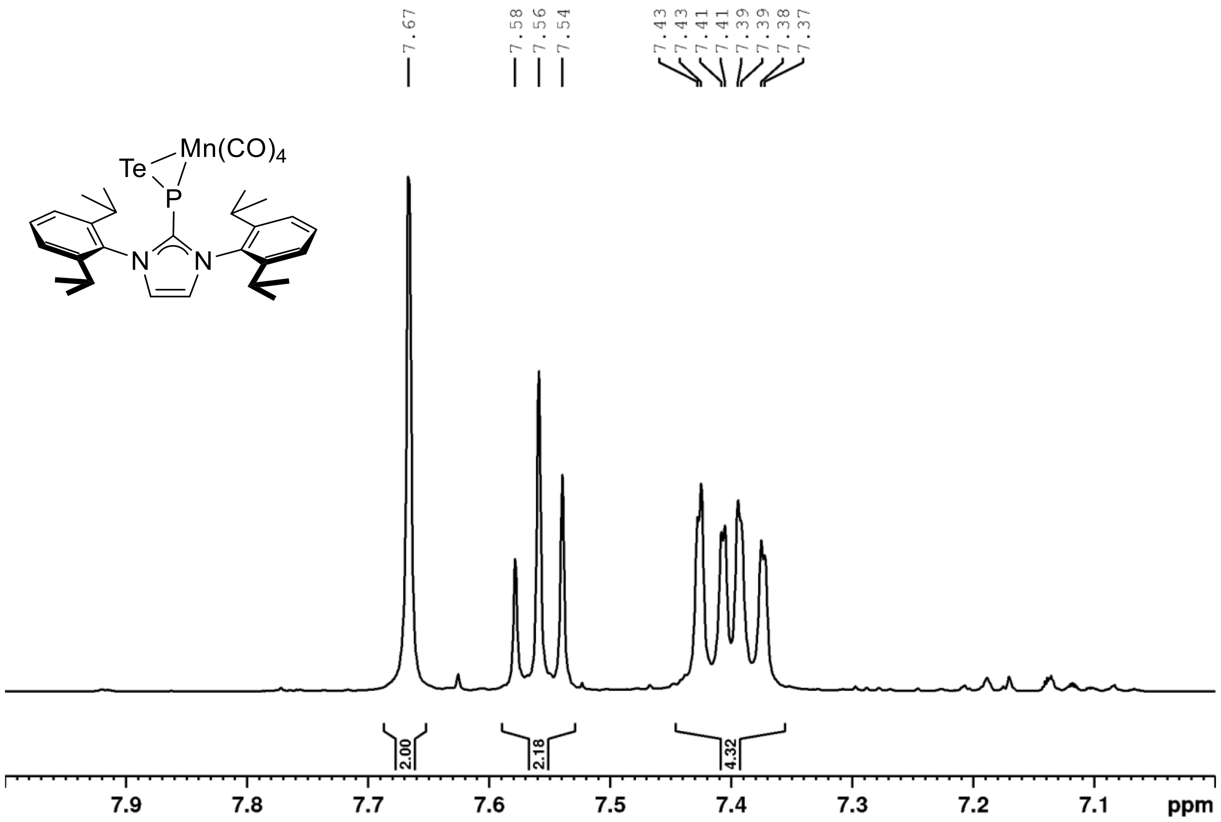

Figure S88: ^{1}H NMR spectrum of {(IDipp)P=Te}Mn(CO)$_4$ (400 MHz, THF-d_8, 298 K, 7.0–8.0 ppm).

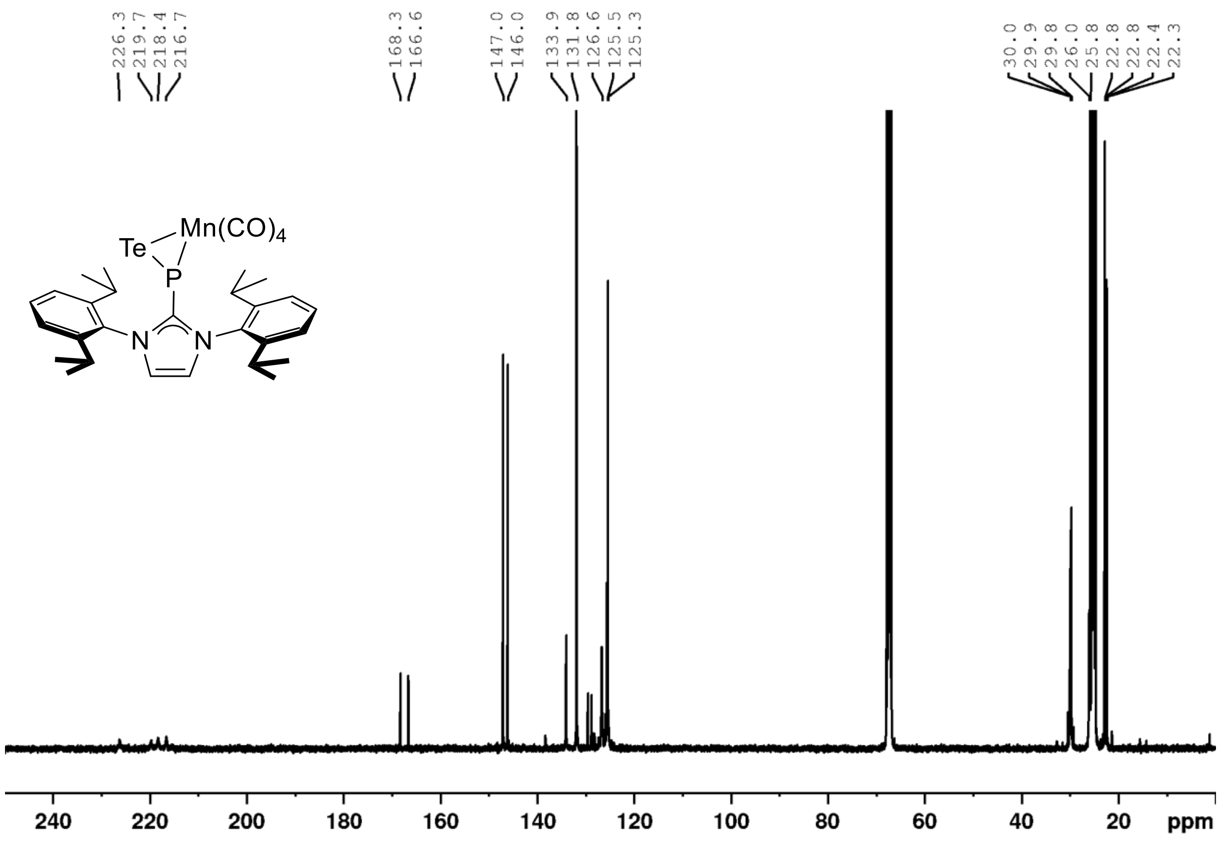

Figure S89: $^{13}C\{^{1}H\}$ NMR spectrum of {(IDipp)P=Te}Mn(CO)$_4$ (101 MHz, THF-d_8, 298 K, overview).

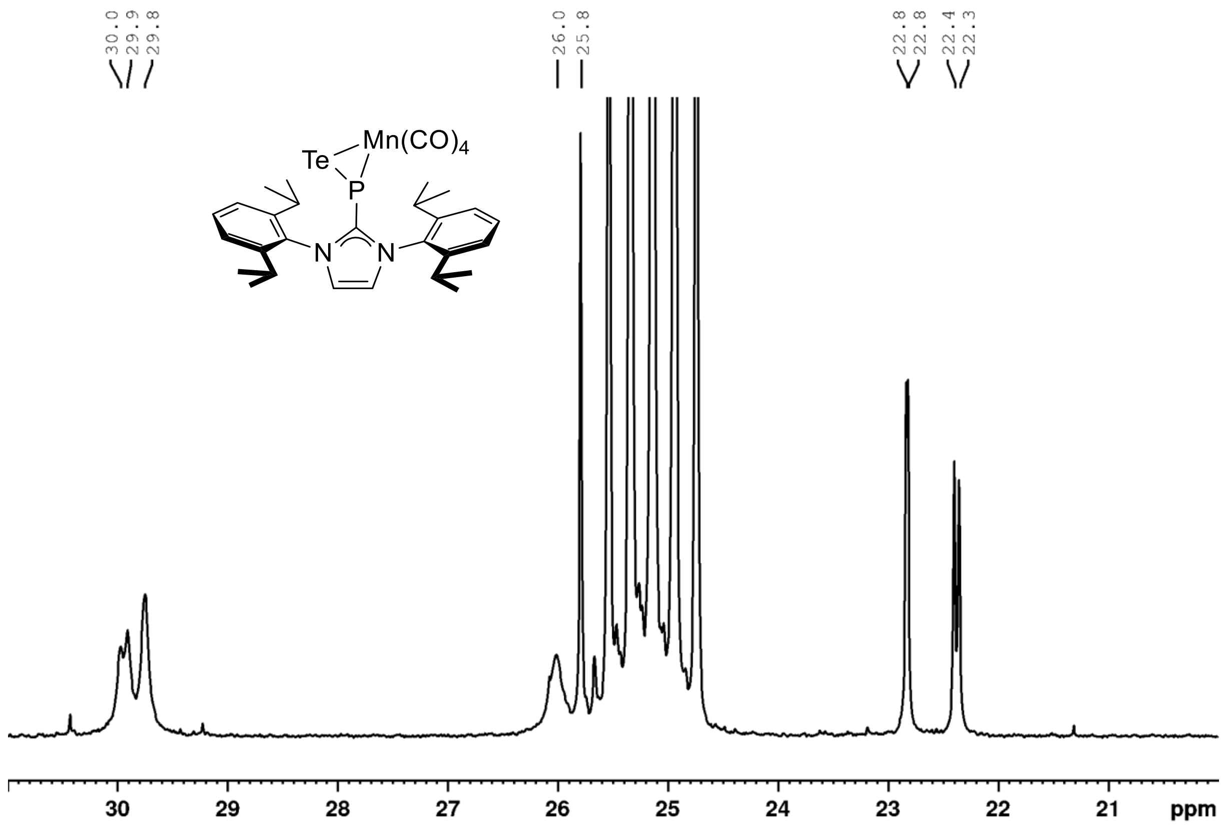

Figure S90: $^{13}C\{^{1}H\}$ NMR spectrum of {(IDipp)P=Te}Mn(CO)$_4$ (101 MHz, THF-d$_8$ 298 K,, 20–31 ppm).

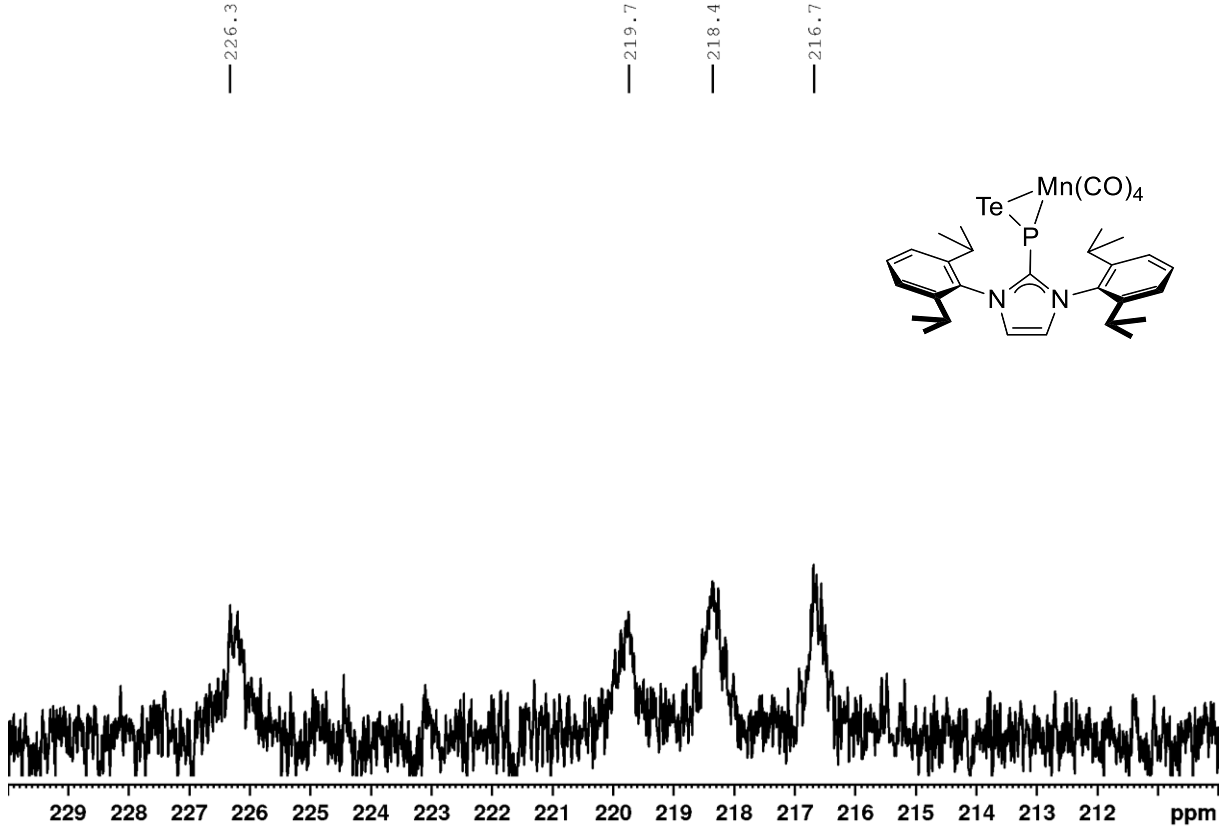

Figure S91: $^{13}C\{^{1}H\}$ NMR spectrum of {(IDipp)P=Te}Mn(CO)$_4$ (101 MHz, THF-d$_8$, 298 K, 210–230 ppm).

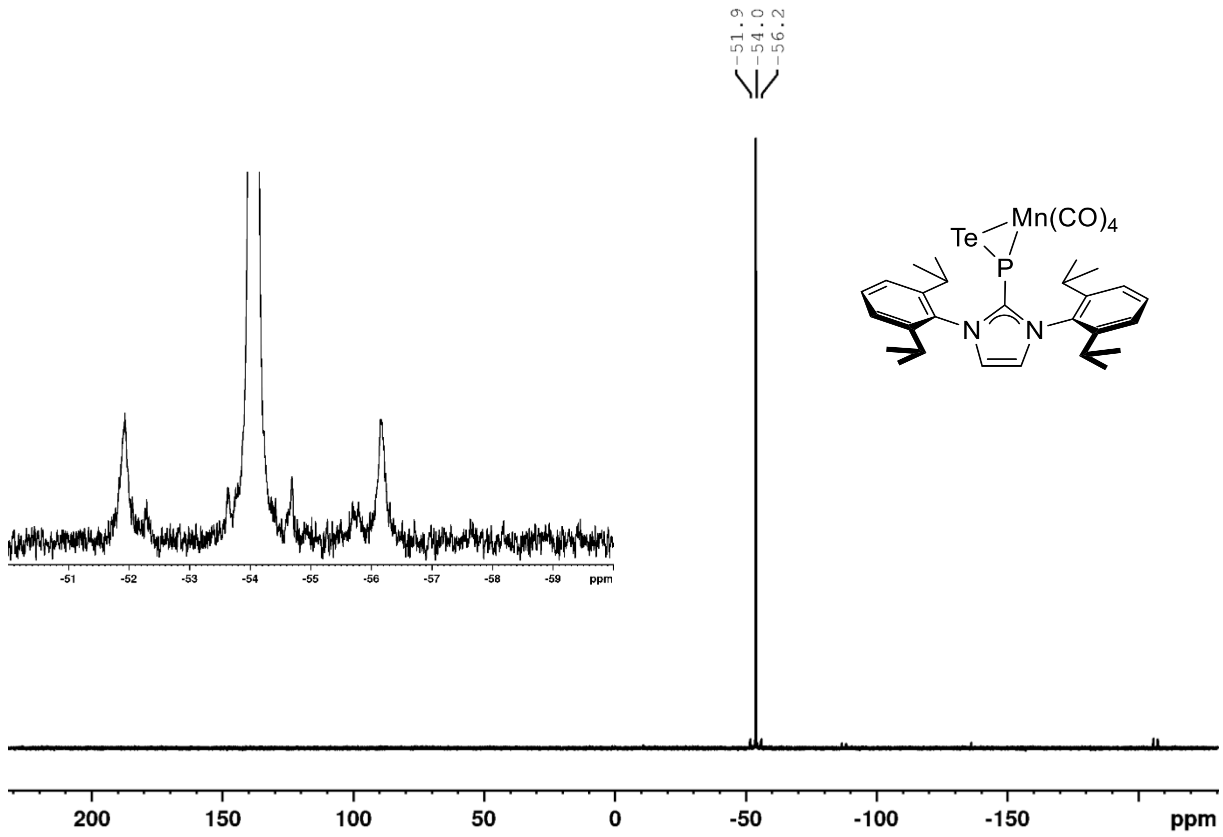

Figure S92: ^{31}P NMR spectrum of {(IDipp)P=Te}Mn(CO)$_4$ (162 MHz, THF-d$_8$, 298 K).

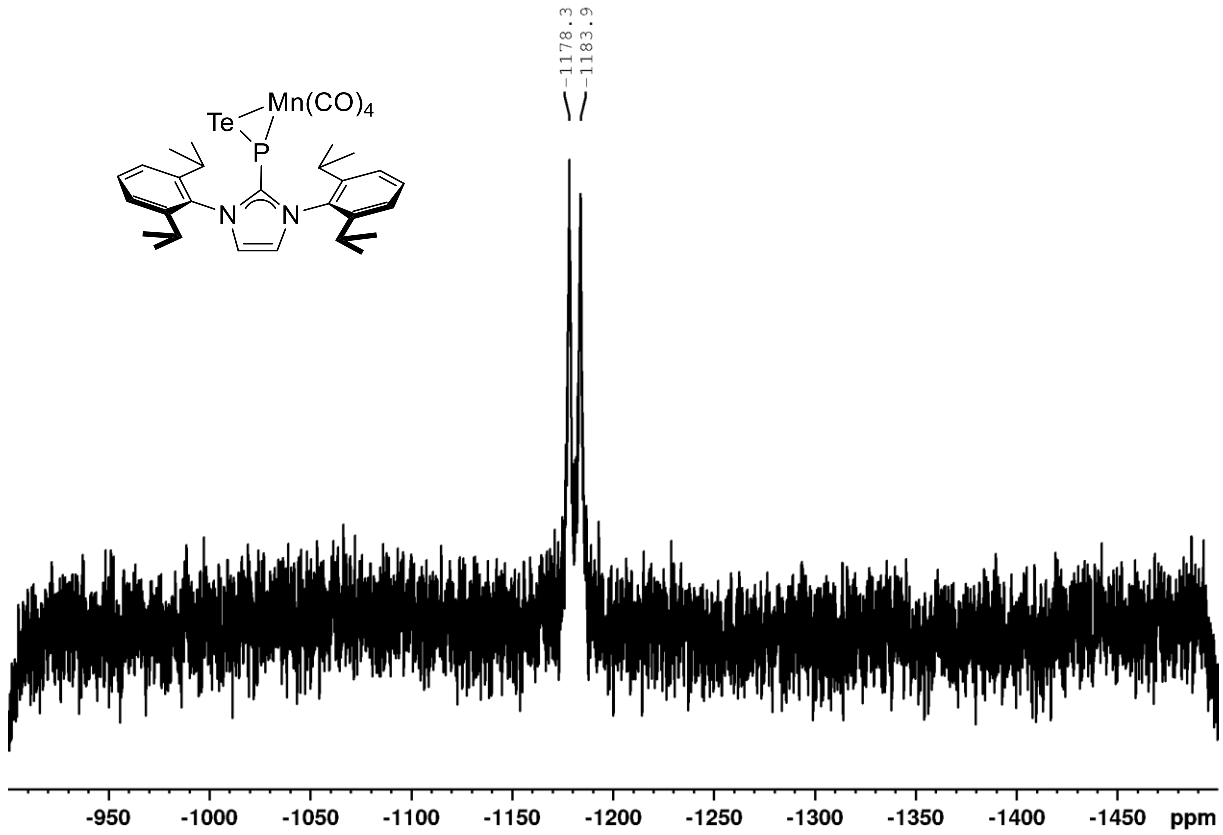

Figure S93: ^{125}Te NMR spectrum of {(IDipp)P=Te}Mn(CO)$_4$ (126 MHz, THF-d$_8$, 298 K).

S4. Preliminary Reactivity Studies with Dihydrogen

In a Schlenk flask, (IDipp)PMn(CO)$_4$ (20 mg, 0.034 mmol) was dissolved in toluene (6 mL). The atmosphere inside the flask was exchanged 15-times with 1 bar H_2 and then sealed. The reaction mixture was stirred for 72 h resulting in an orange solution. Afterwards, the solvent was removed under reduced pressure and an NMR spectrum (C_6D_6) was recorded. Single crystals suitable for x-ray diffraction analysis could be grown by vapor diffusion of *n*-hexane into the NMR solution. From this mixture, single crystals of {(IDipp)P=PH}Mn(CO)$_4$ and [(IDipp)H][{(IDipp)P(μ_2-PH)}$Mn_2(CO)_8$] were measured and their crystals structures determined.

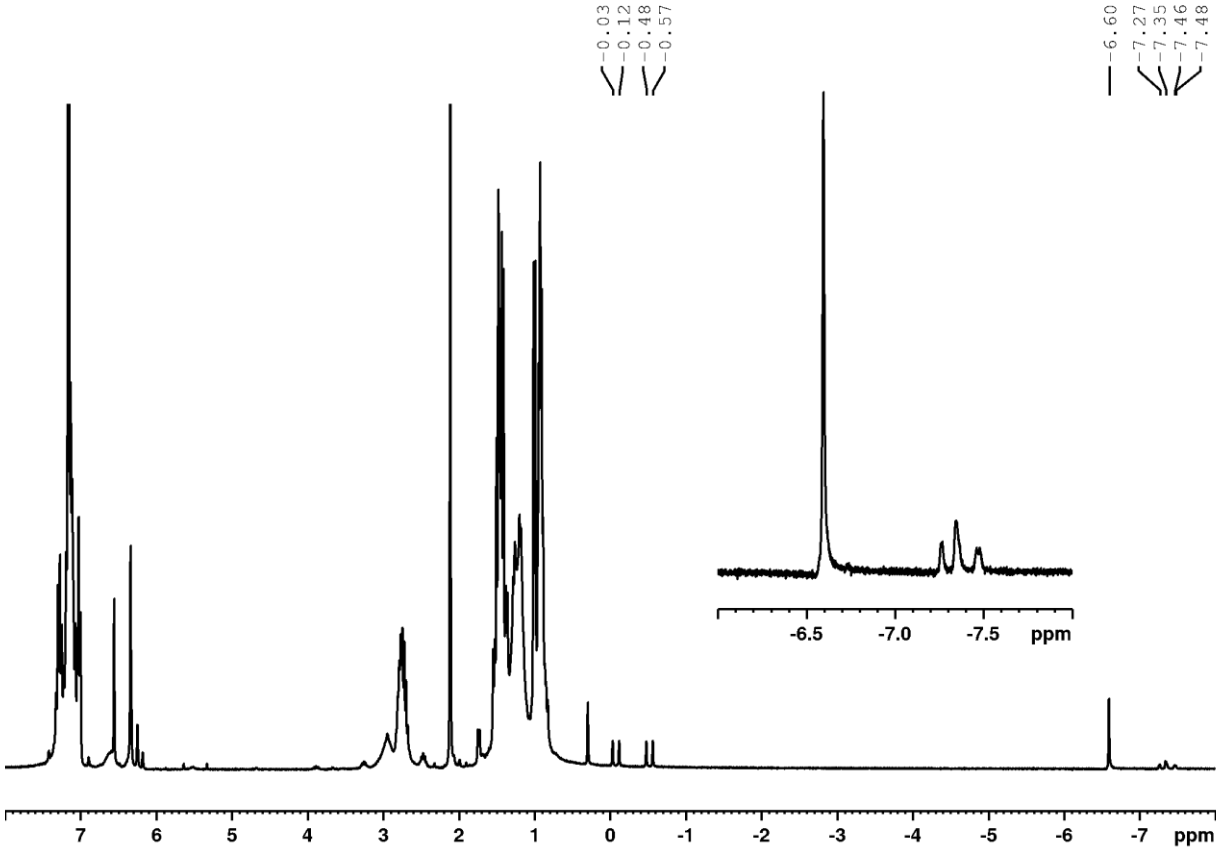

Figure S94: ^{1}H NMR spectrum obtained from the reaction mixture (IDipp)PMn(CO)$_4$ + H_2 (300 MHz, C_6D_6, (−8)–8 ppm).

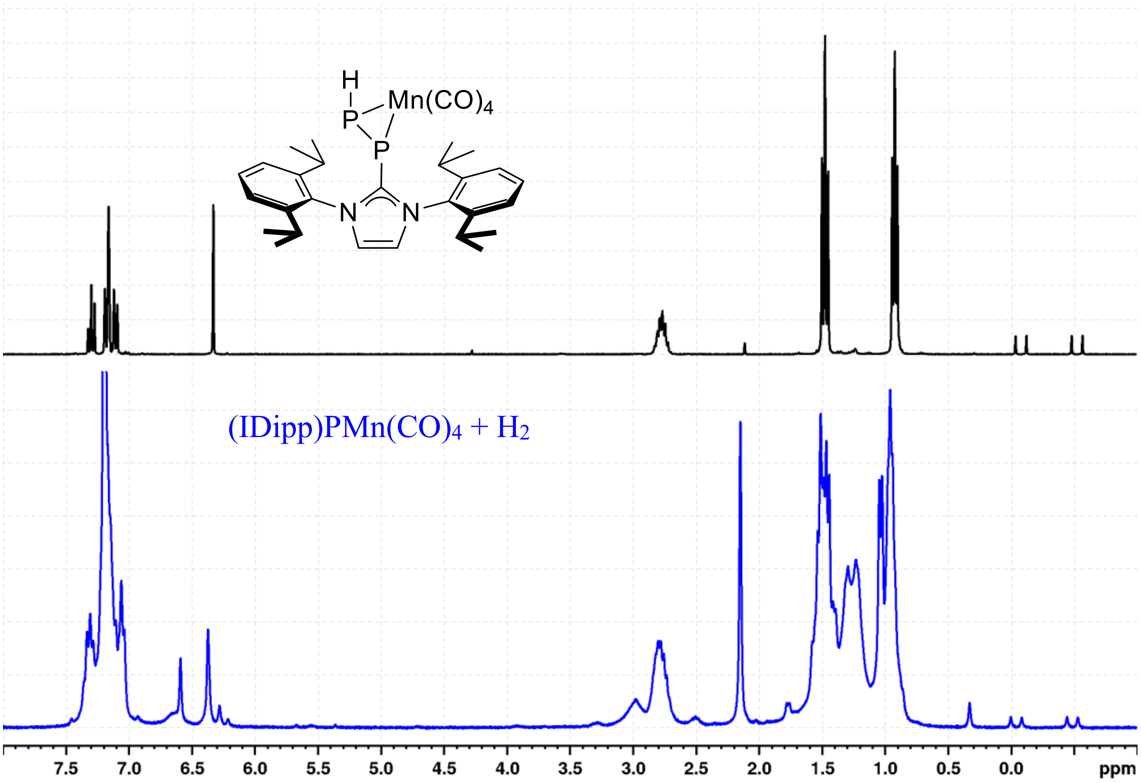

Figure S95: ^{1}H NMR spectrum obtained from the reaction mixture (IDipp)PMn(CO)$_4$ + H_2 (bottom, blue) and (IDipp)PMn(CO)$_4$ (top, black) measured at 300 MHz in C_6D_6).

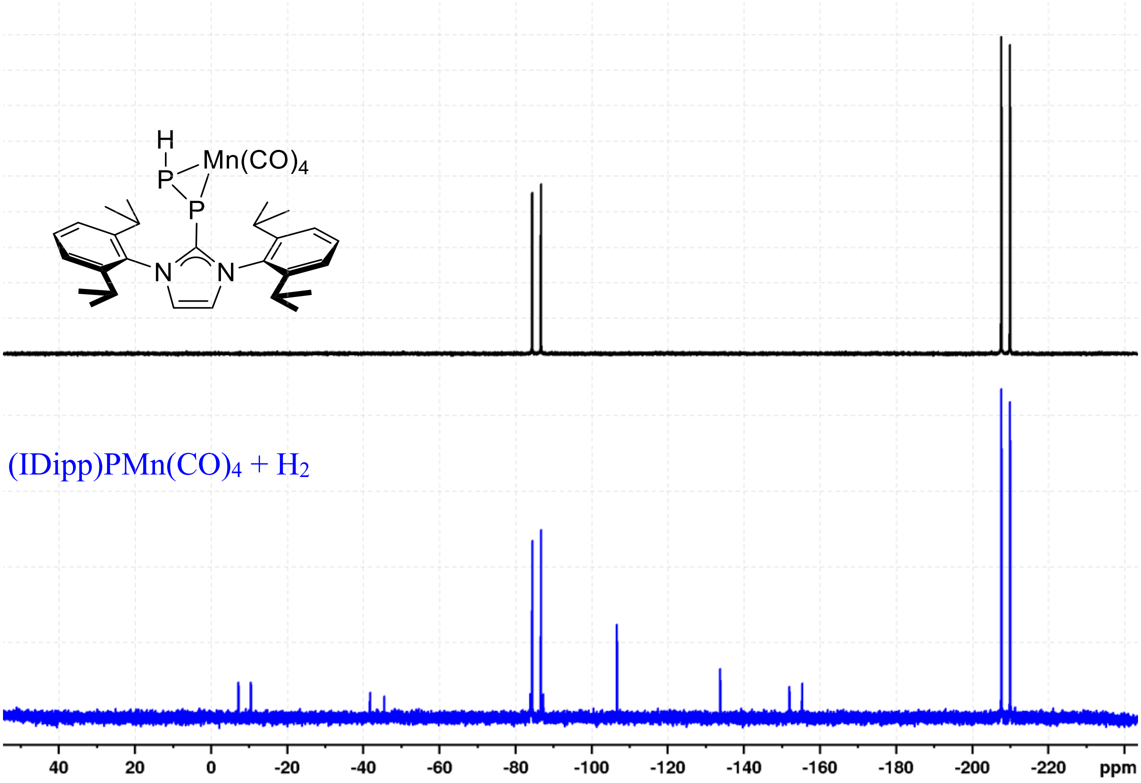

Figure S96: ^{31}P {^{1}H} NMR spectrum obtained from the reaction mixture (IDipp)PMn(CO)$_4$ + H_2 (bottom, blue) and (IDipp)PMn(CO)$_4$ (top, black) measured at 121.5 MHz in C_6D_6).

S5. Investigation of the CO Substitution Reaction with PPh_3 at Variable Temperatures

[(IDipp)PMn(CO)$_4$] (10.0 mg, 0.017 mmol, 1.0 eq.) and PPh_3 (4.5 mg, 0.017 mmol, 1.0 eq.) were dissolved in toluene-d_8 (0.6 mL). The dark green mixture was filtered and NMR spectra were recorded approximately 30 min after start of the reaction.

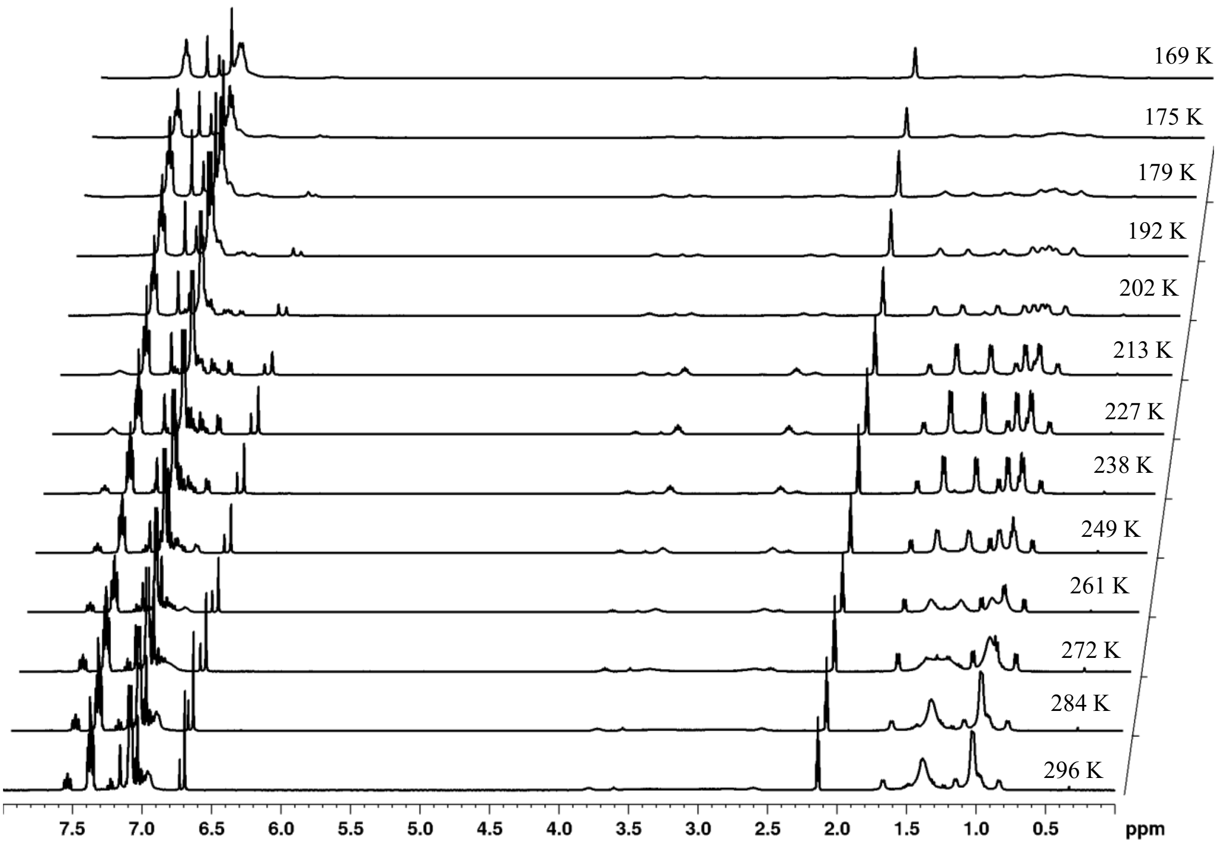

Figure S97: ^{1}H NMR spectrum of the crude reaction mixture of [(IDipp)PMn(CO)$_4$] and PPh_3 (400 MHz, toluene-d_8, 296–169 K, overview).

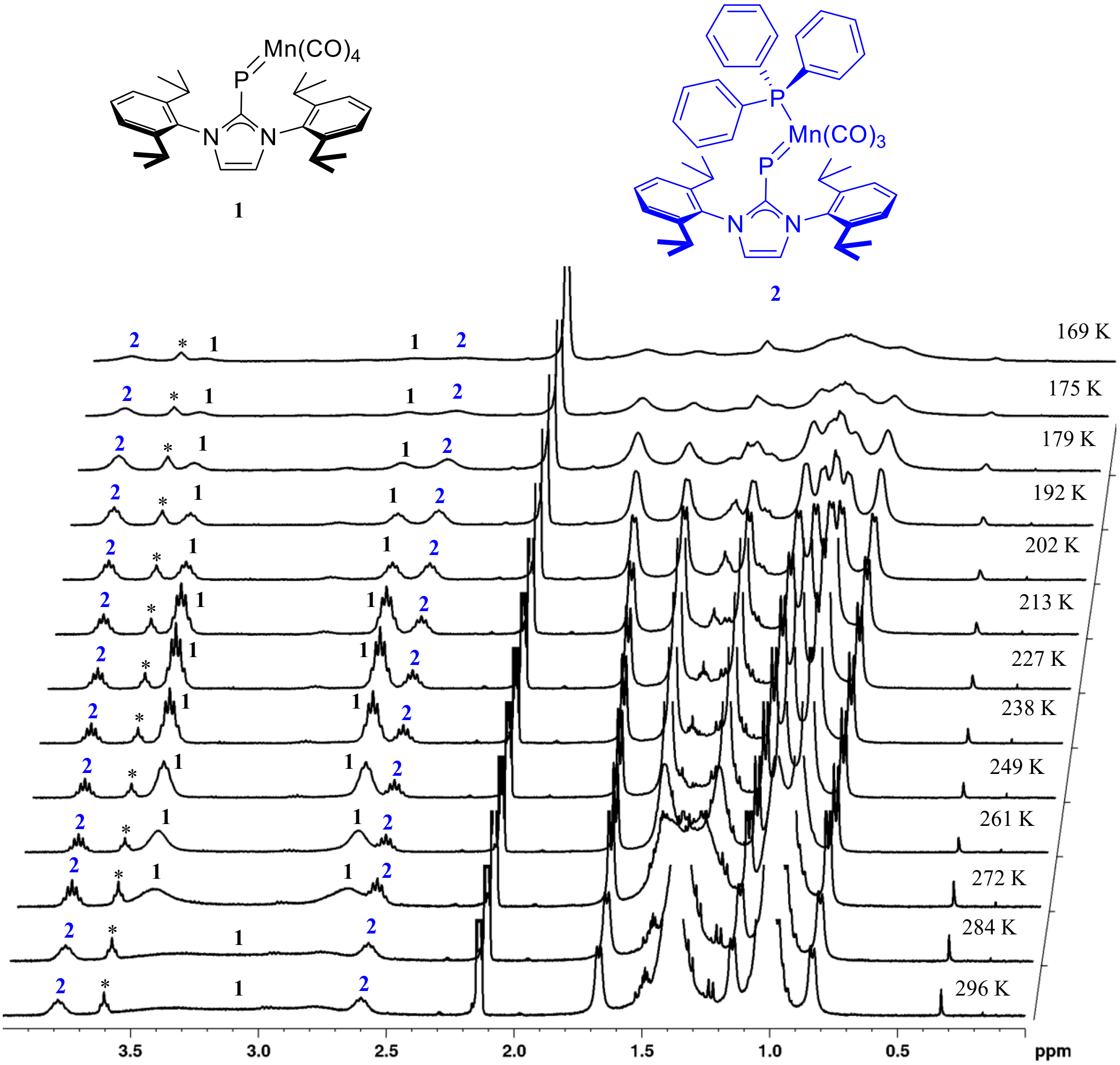

Figure S98: ^{1}H NMR spectrum of the crude reaction mixture of [(IDipp)PMn(CO)4] and PPh3 (400 MHz, toluene-d_8, 296–169 K, 4.0–0.0 ppm, * = residual THF).

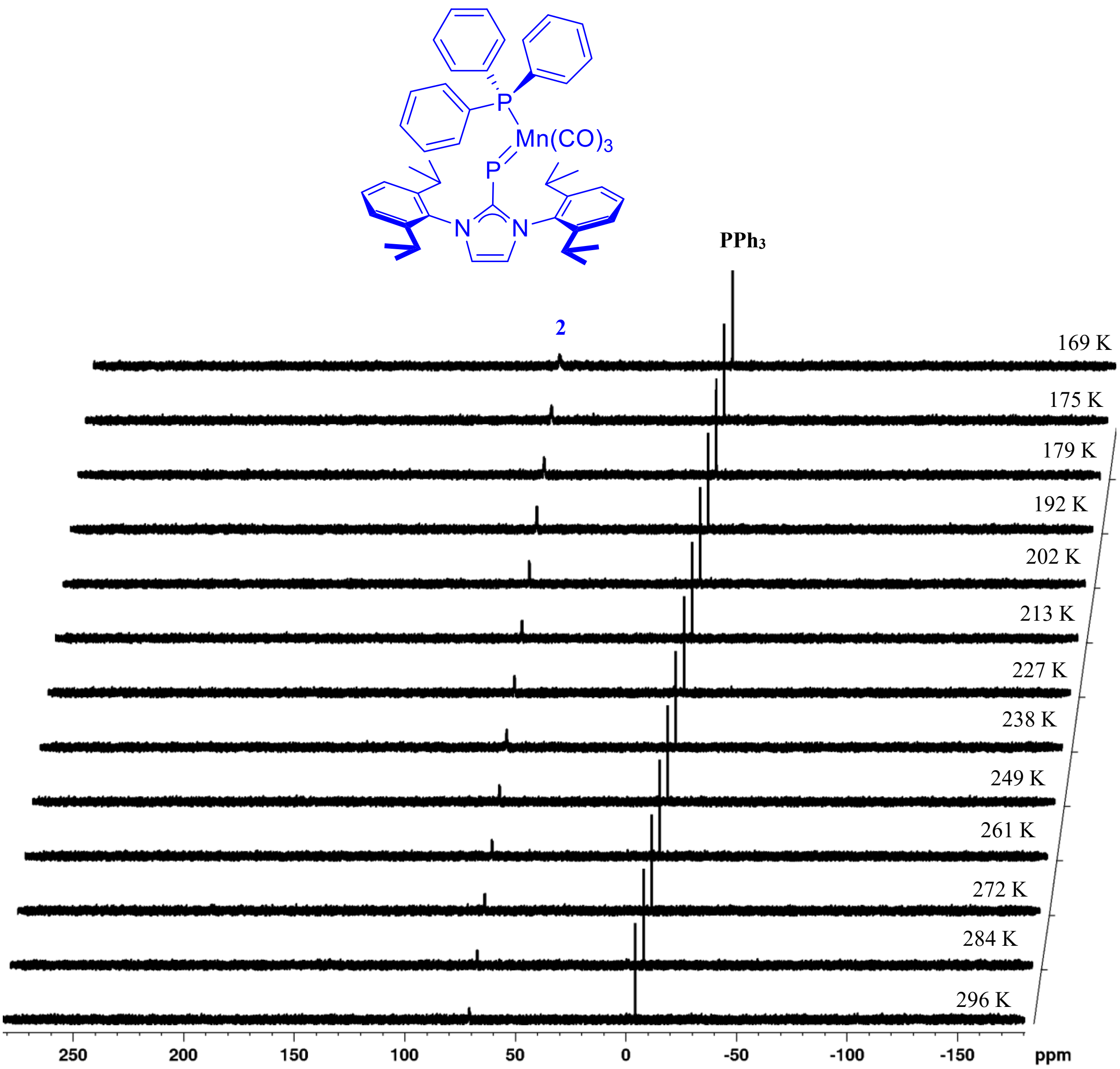

Figure S99: $^{31}P\{^{1}H\}$ NMR spectrum of the crude reaction mixture of [(IDipp)PMn(CO)$_4$] and PPh_3 (162 MHz, toluene-d_8, 296–169 K, (–180)–280 ppm).

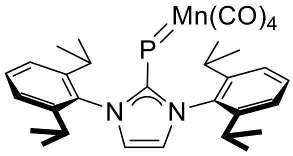

1

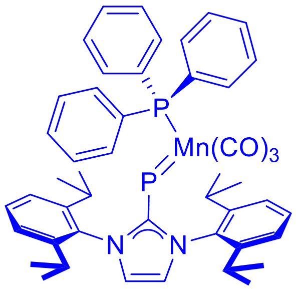

2

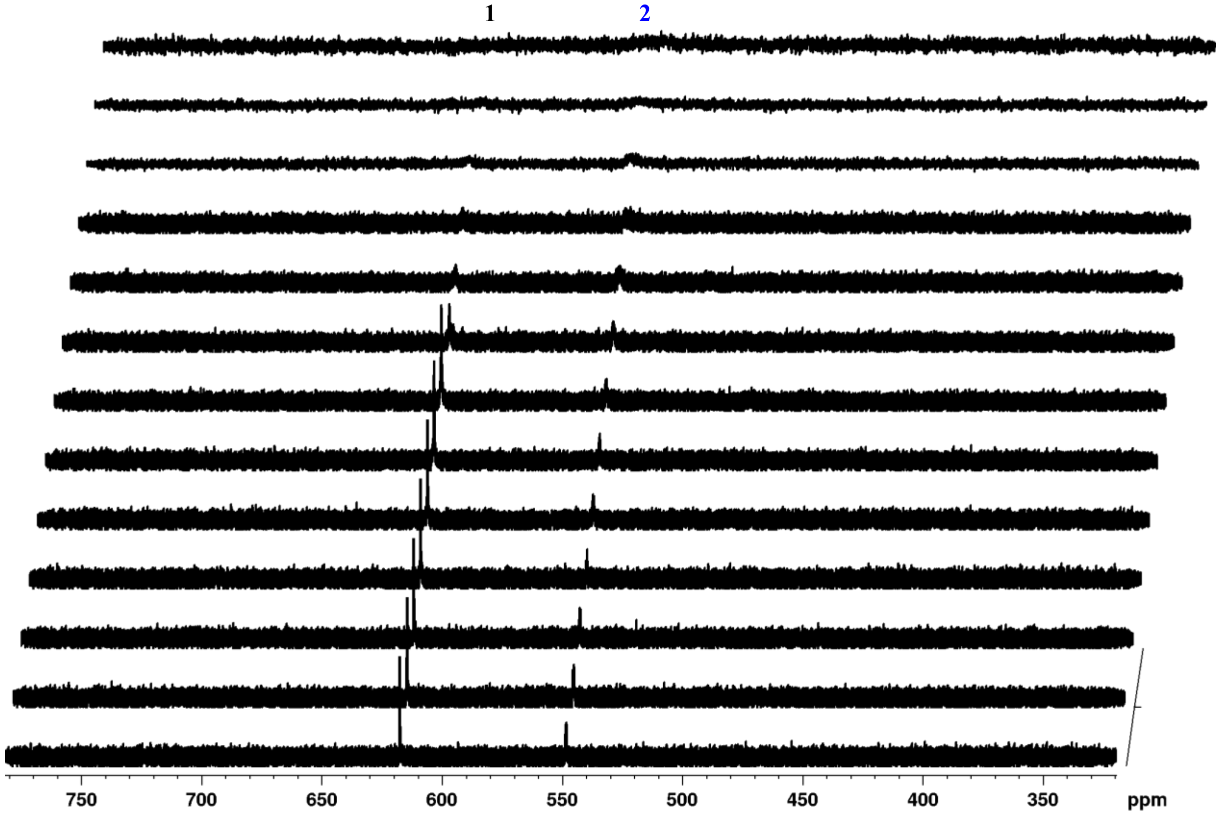

Figure S100: $^{31}P\{^{1}H\}$ NMR spectrum of the crude reaction mixture of [(IDipp)PMn(CO)$_4$] and PPh_3 (162 MHz, toluene-d_8, 296–169 K, 320–780 ppm).

S6. IR Spectra

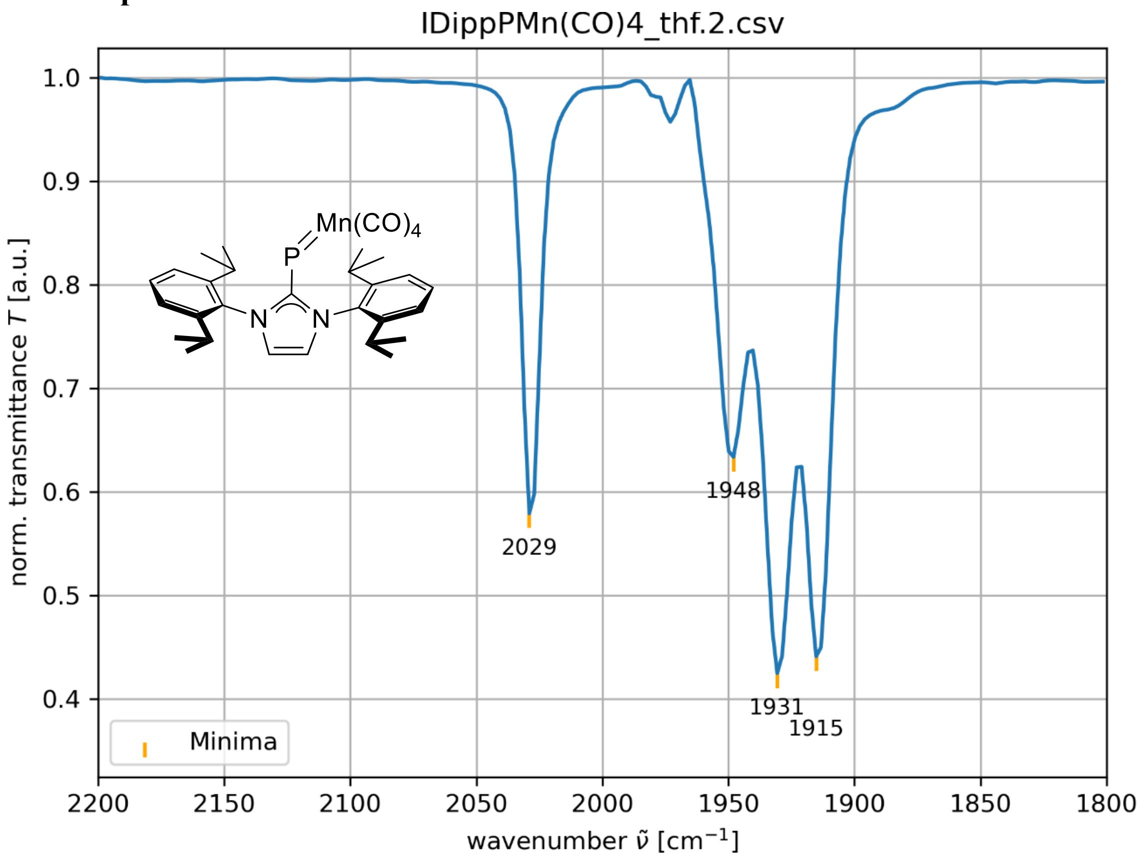

Figure S101: IR spectrum of (IDipp)PMn(CO)$_4$ (THF).

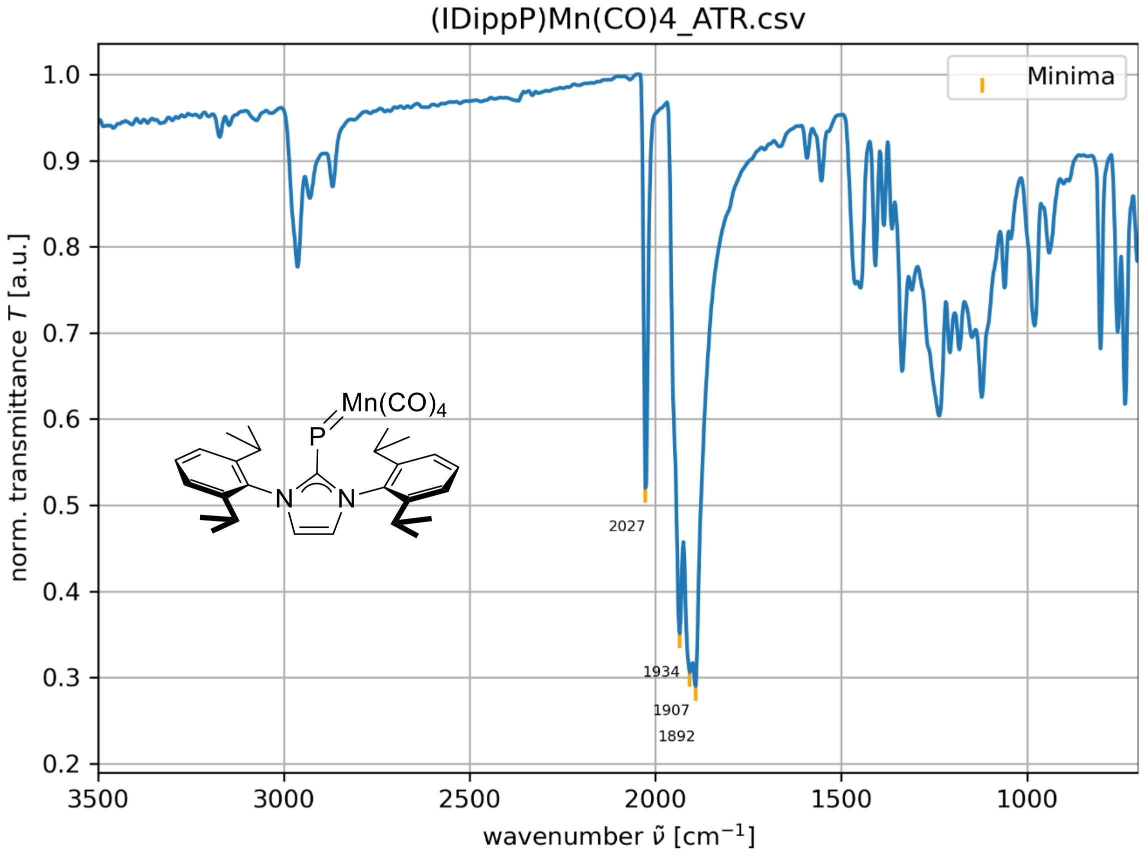

Figure S102: IR spectrum of (IDipp)PMn(CO)$_4$ (ATR).

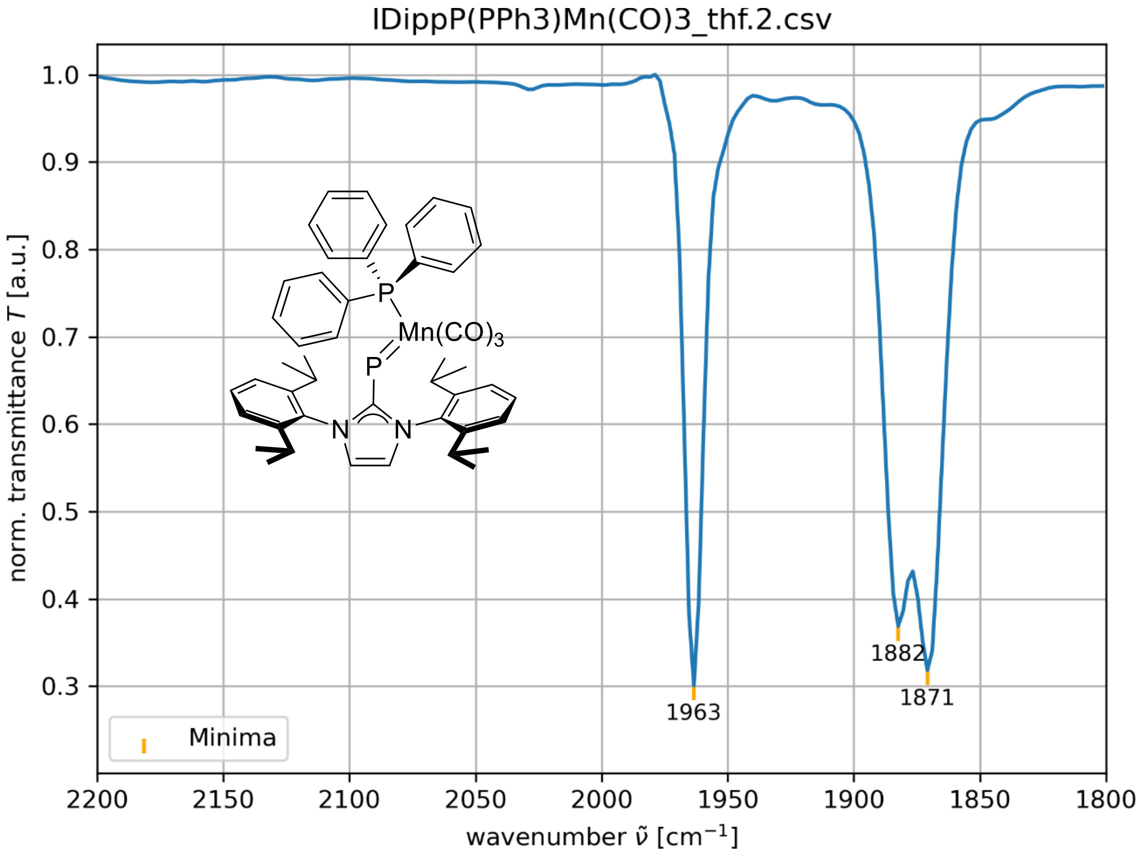

Figure S103: IR spectrum of (IDipp)PMn(PPh_3)$(CO)_3$ (THF).

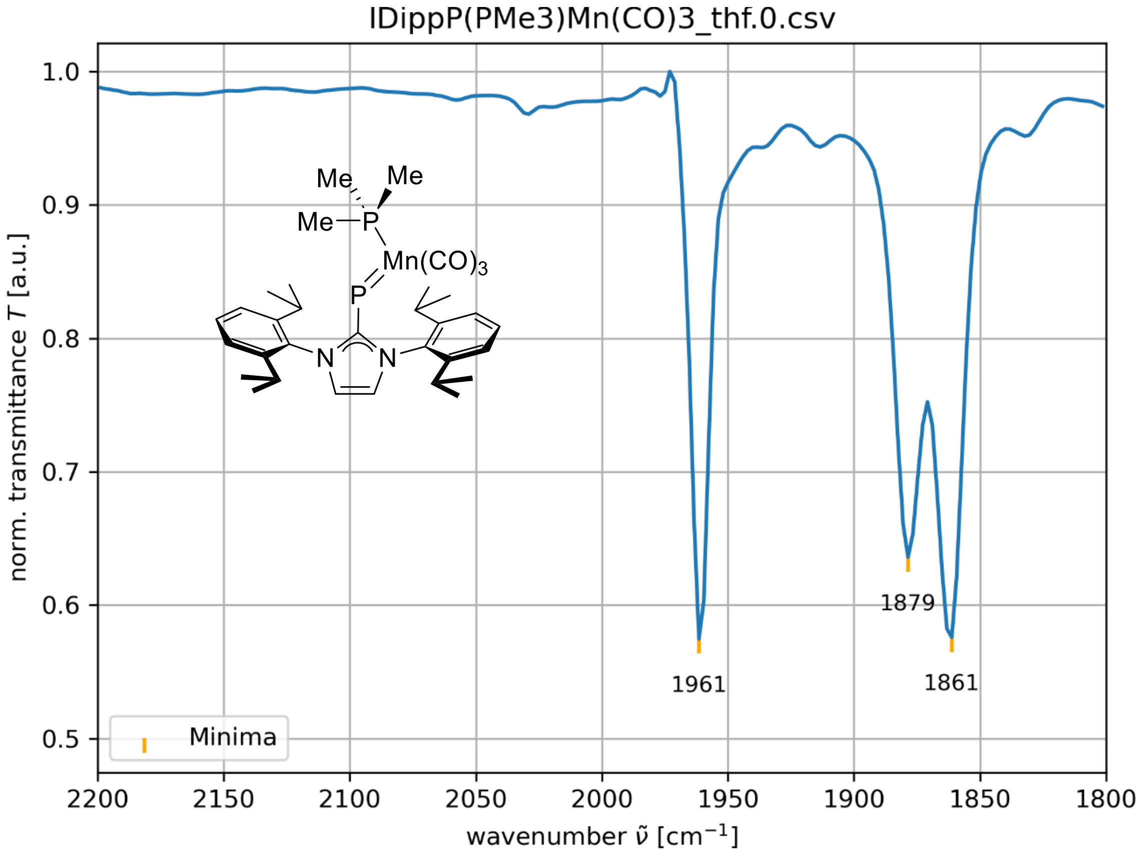

Figure S104: IR spectrum of (IDipp)PMn(PMe_3)$(CO)_3$ (THF).

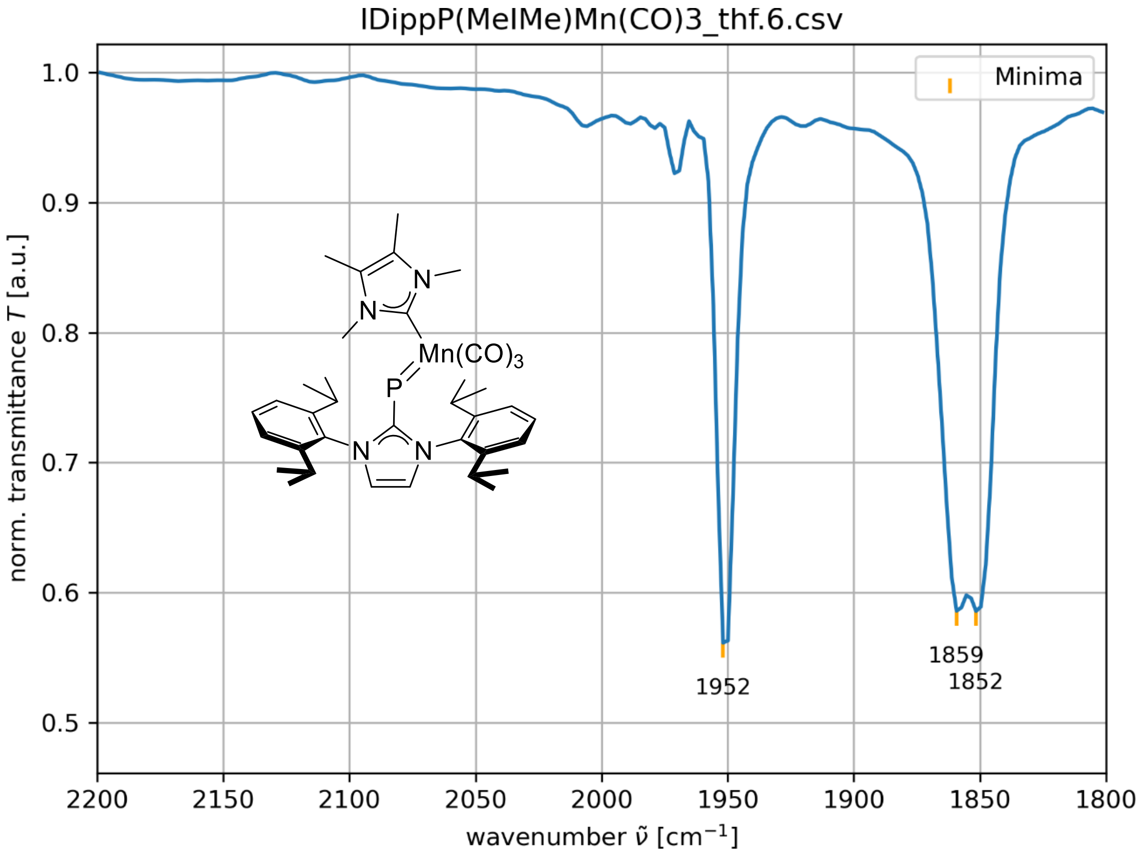

Figure S105: IR spectrum of (IDipp)PMn(IMe)$(CO)_3$ (THF).

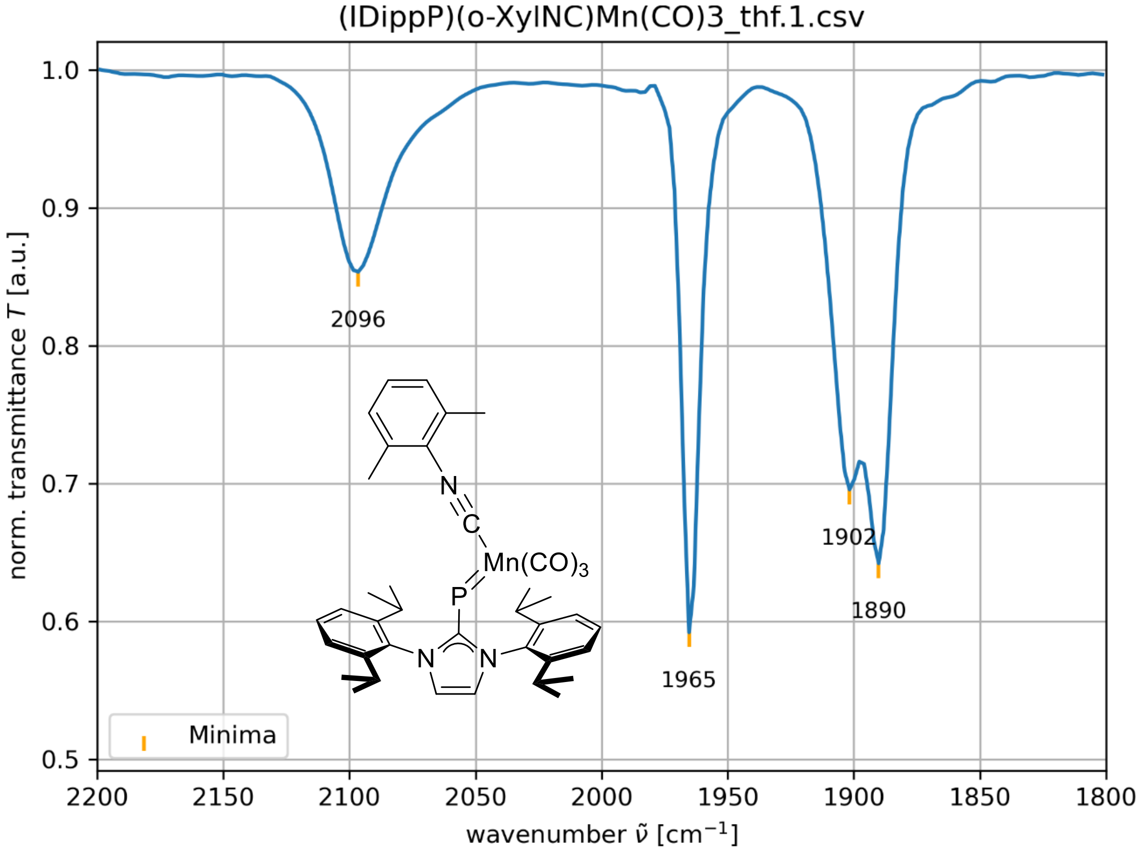

Figure S106: IR spectrum of (IDipp)PMn(XyNC)$(CO)_3$ (THF).

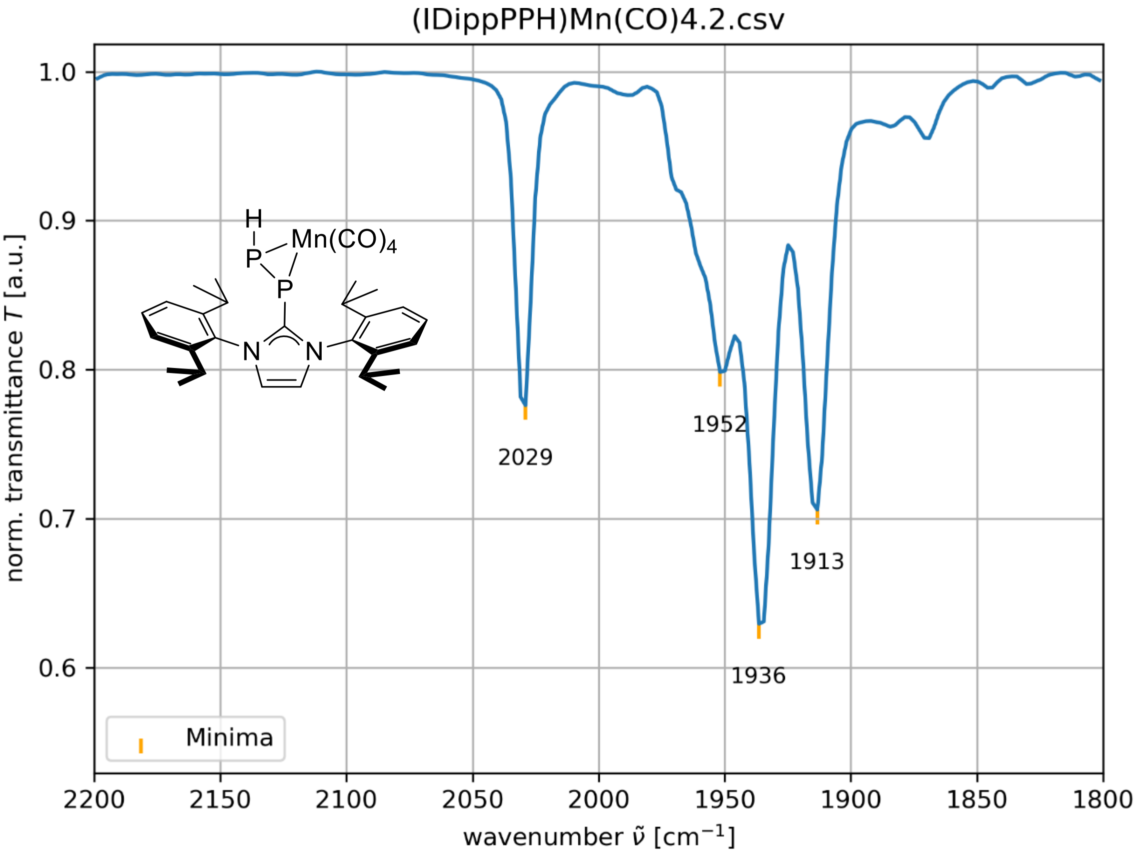

Figure S107: IR spectrum of {(IDipp)P=PH}Mn(CO)₄ (THF).

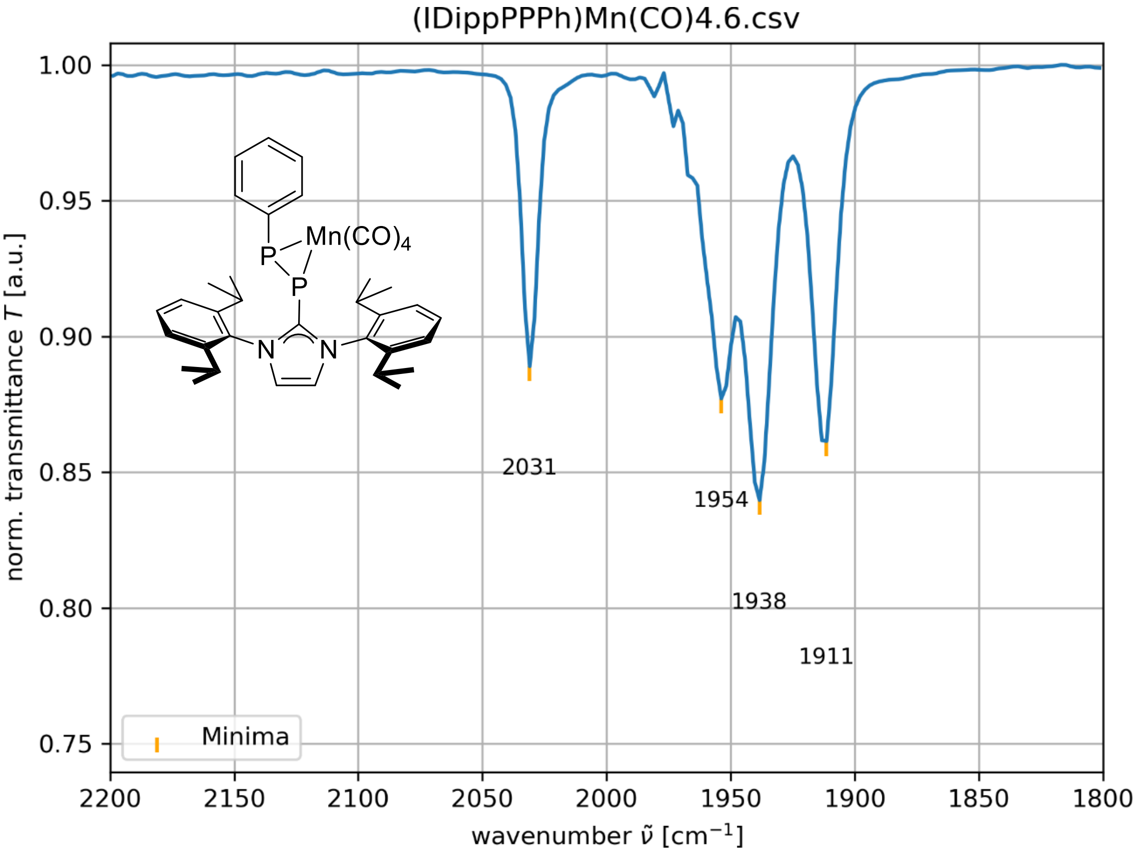

Figure S108: IR spectrum of {(IDipp)P=PPh}Mn(CO)₄ (THF).

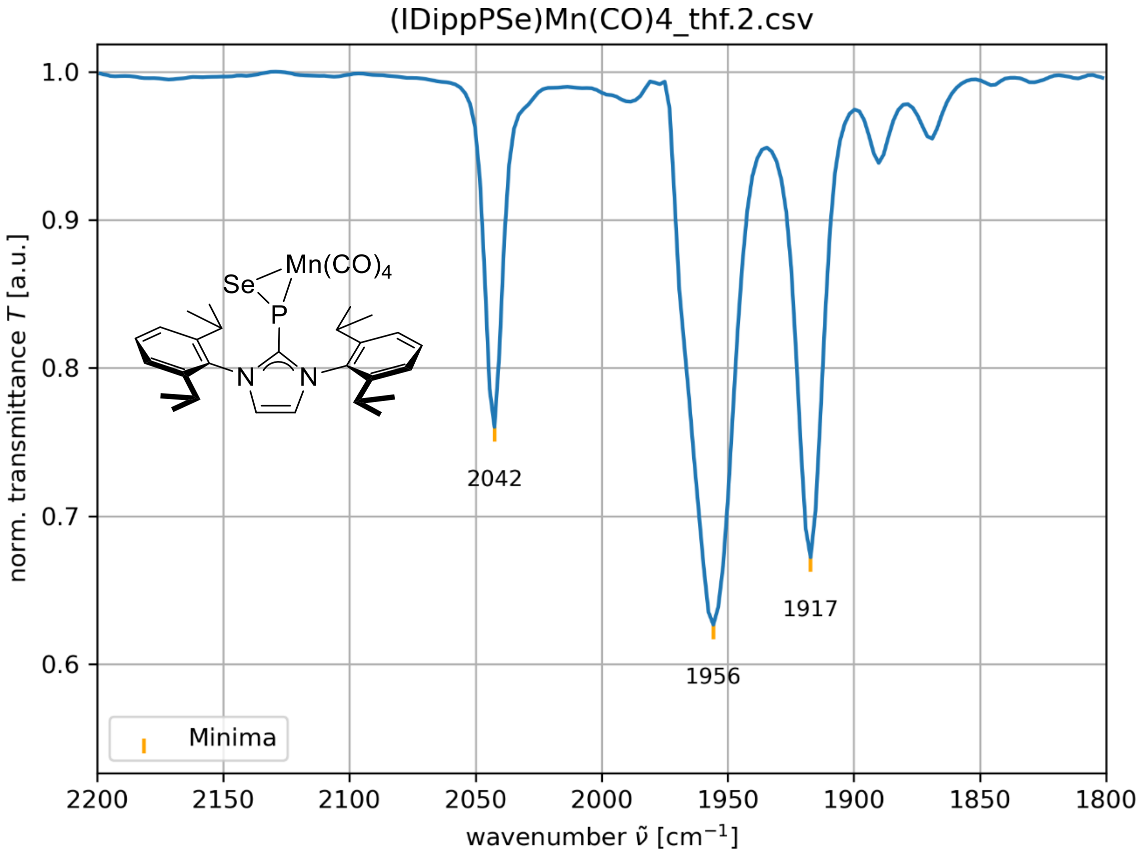

Figure S109: IR spectrum of (IDippPSe)$Mn(CO)_4$ (THF).

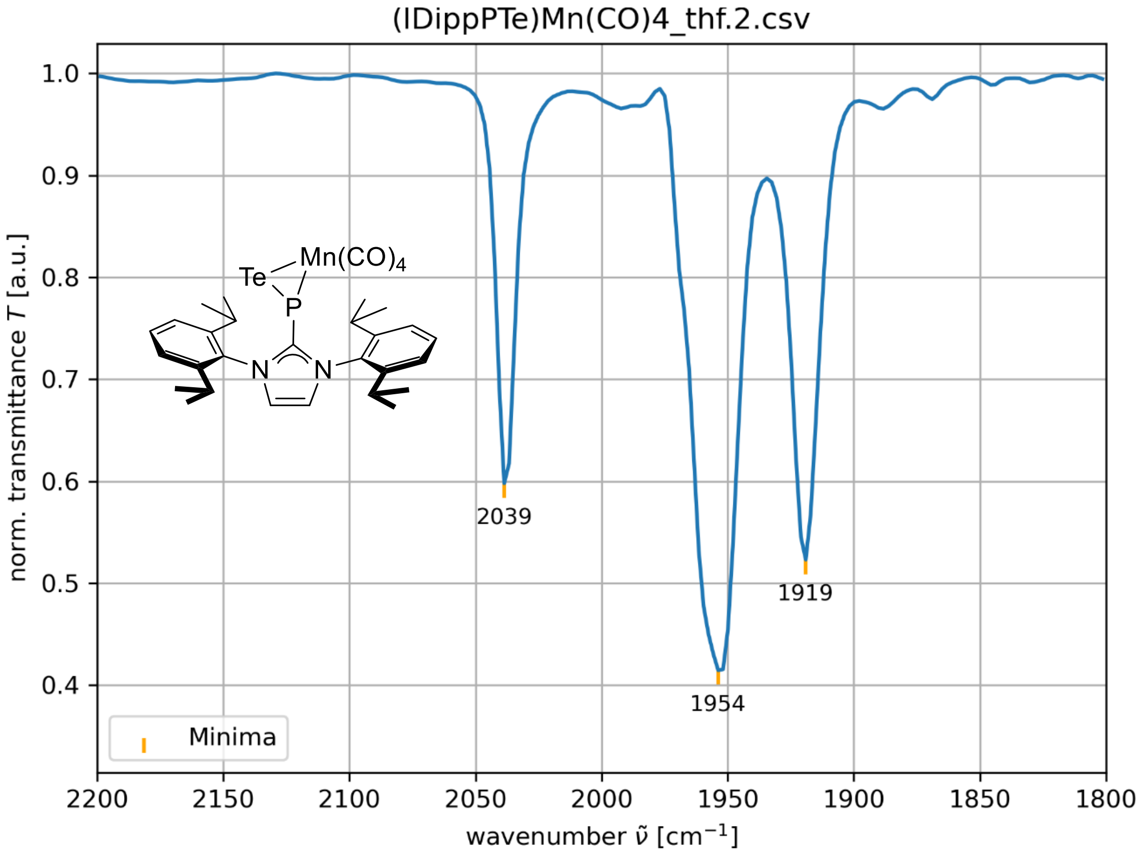

Figure S110: IR spectrum of (IDippPTe)$Mn(CO)_4$ (THF).

S7. UV/Vis Spectra

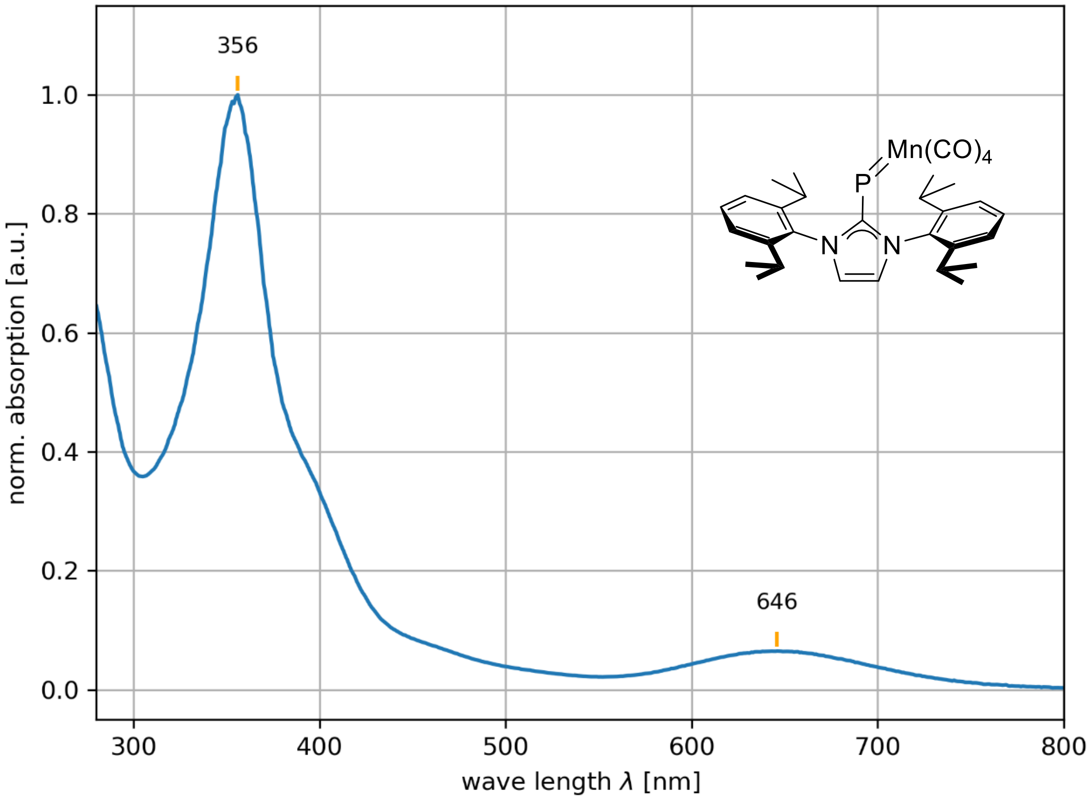

Figure S111: Normalized UV/Vis Spectrum of (IDipp)PMn(CO)$_4$ (THF).

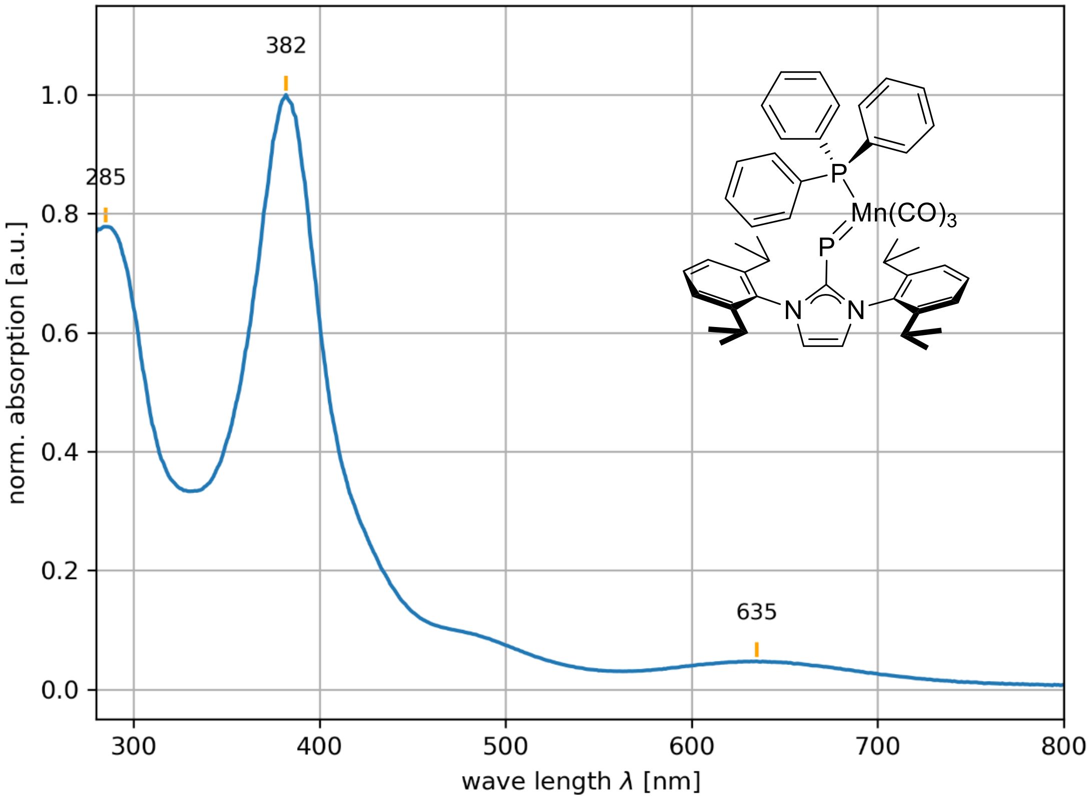

Figure S112: Normalized UV/Vis Spectrum of (IDipp)P(PPh$_3$)Mn(CO)$_3$ (THF).

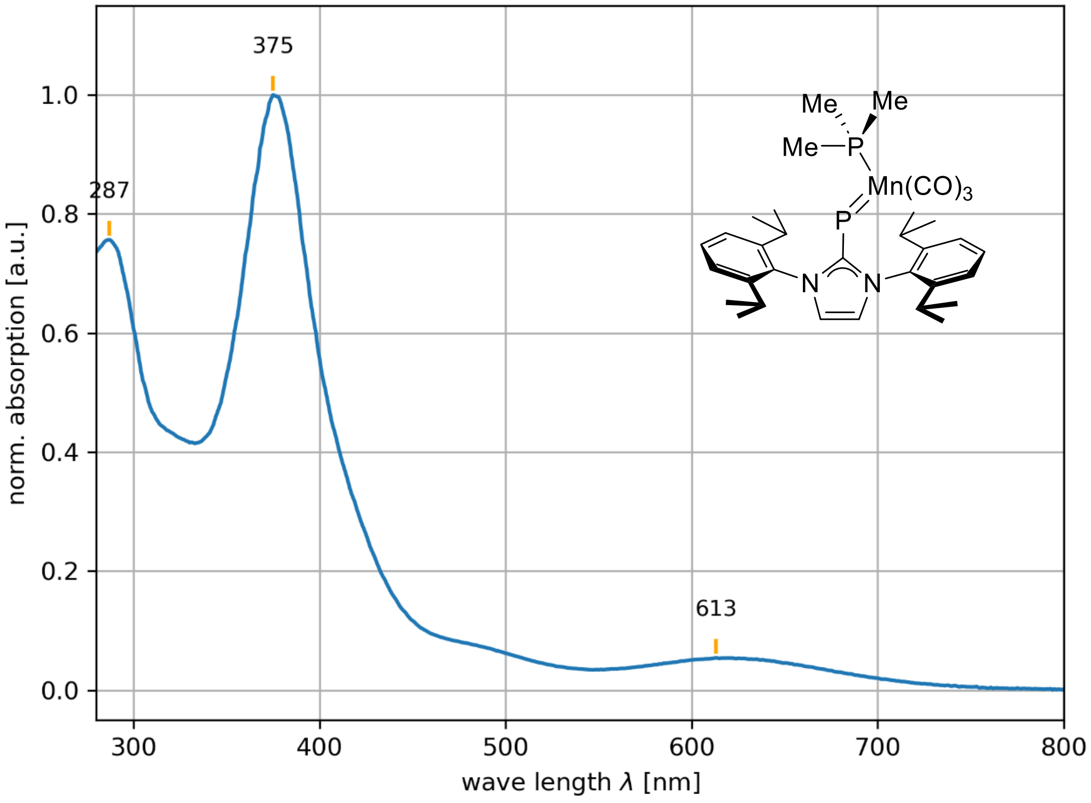

Figure S113: Normalized UV/Vis Spectrum of (IDipp)P(PMe$_3$)Mn(CO)$_3$ (THF).

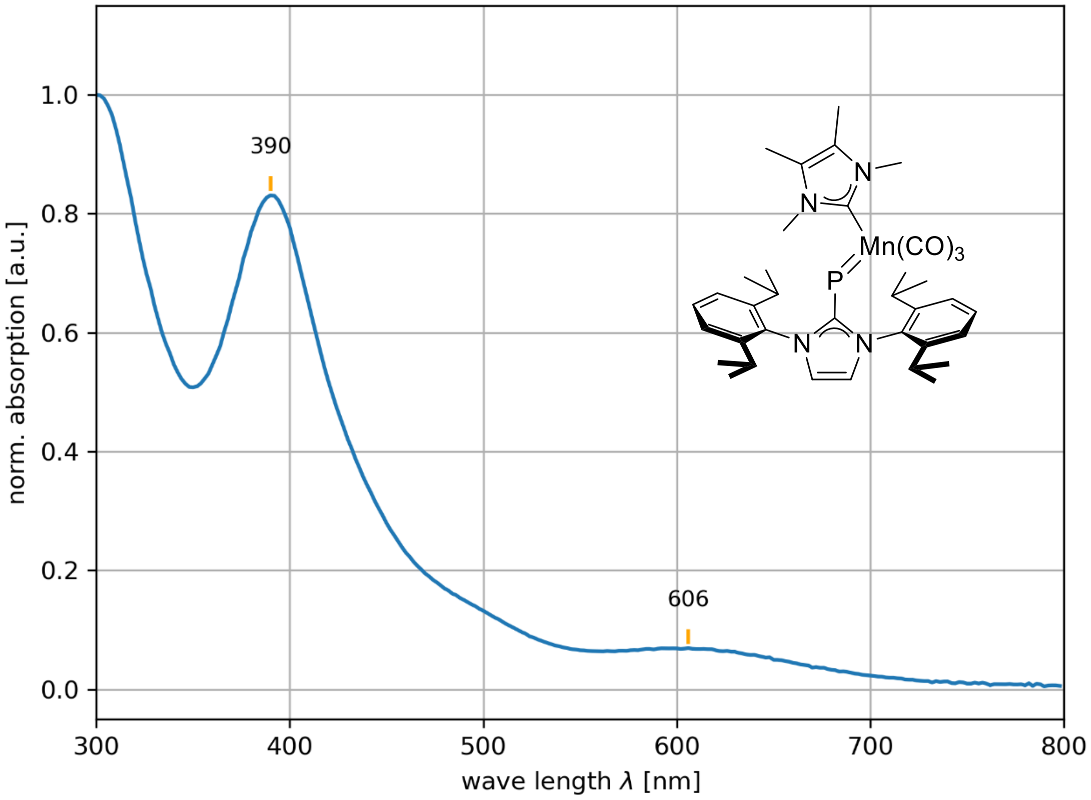

Figure S114: Normalized UV/Vis Spectrum of (IDipp)P(IMe)Mn(CO)$_3$ (THF).

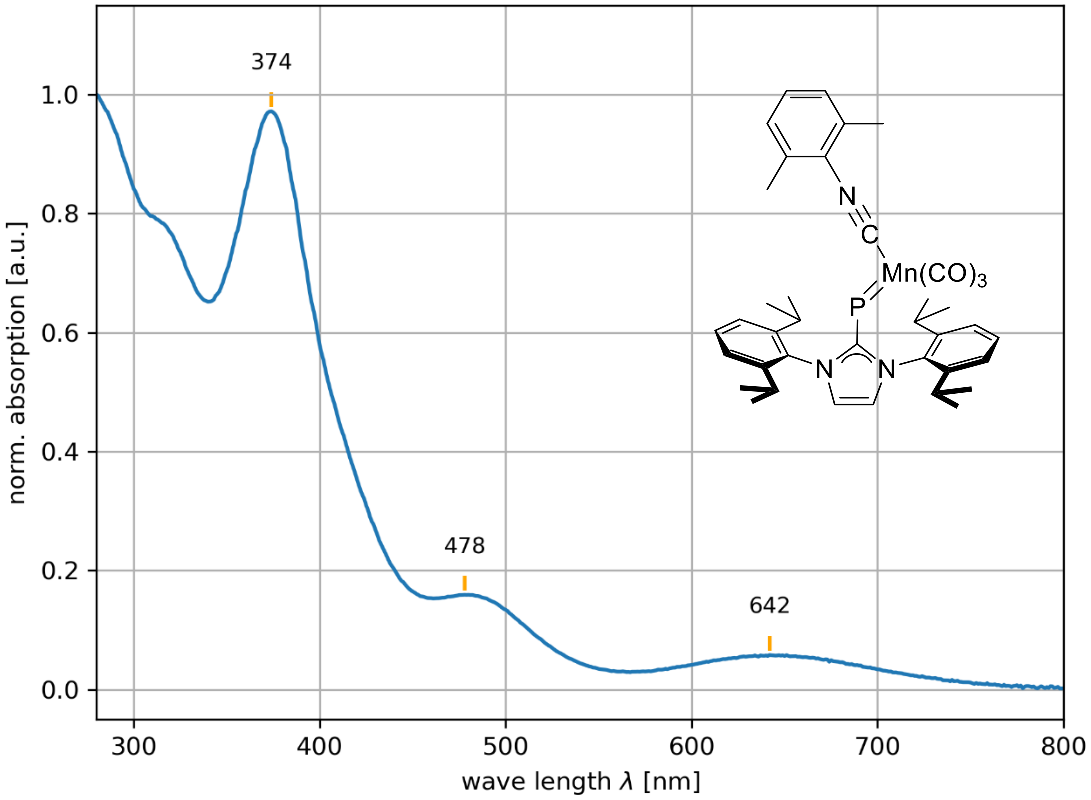

Figure S115: Normalized UV/Vis Spectrum of (IDipp)P(XyNC)Mn(CO)$_3$ (THF).

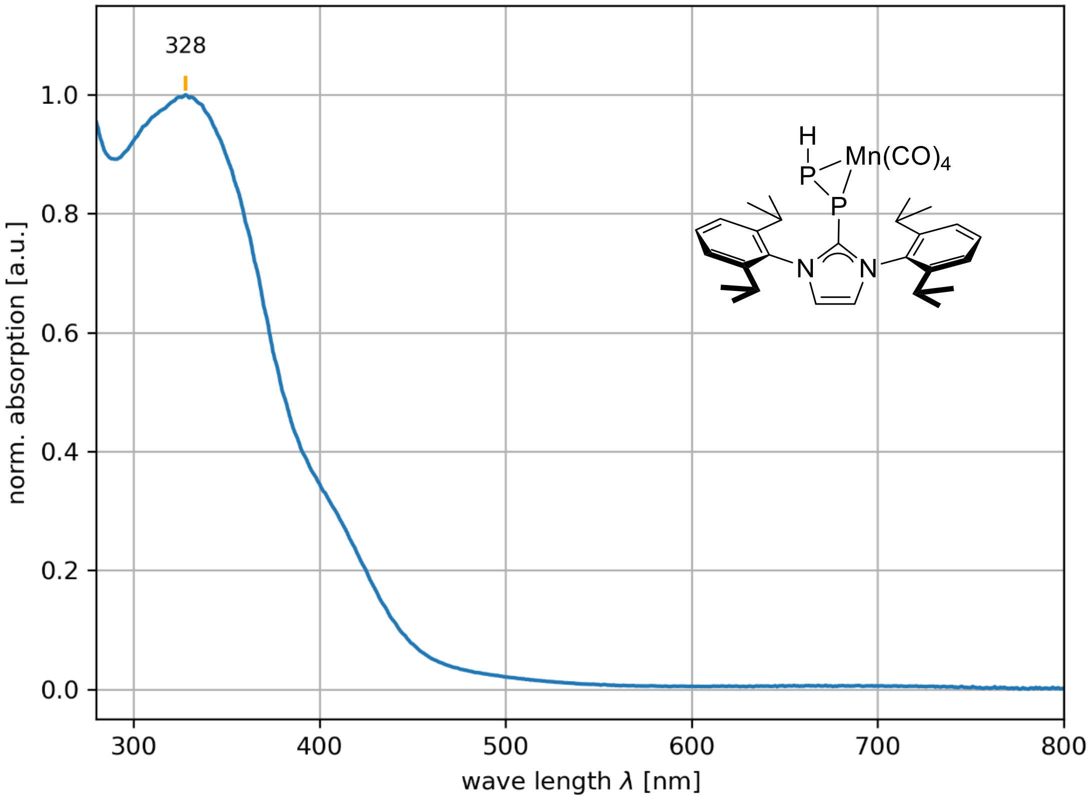

Figure S116: Normalized UV/Vis Spectrum of {(IDipp)P=PH}Mn(CO)$_4$ (THF).

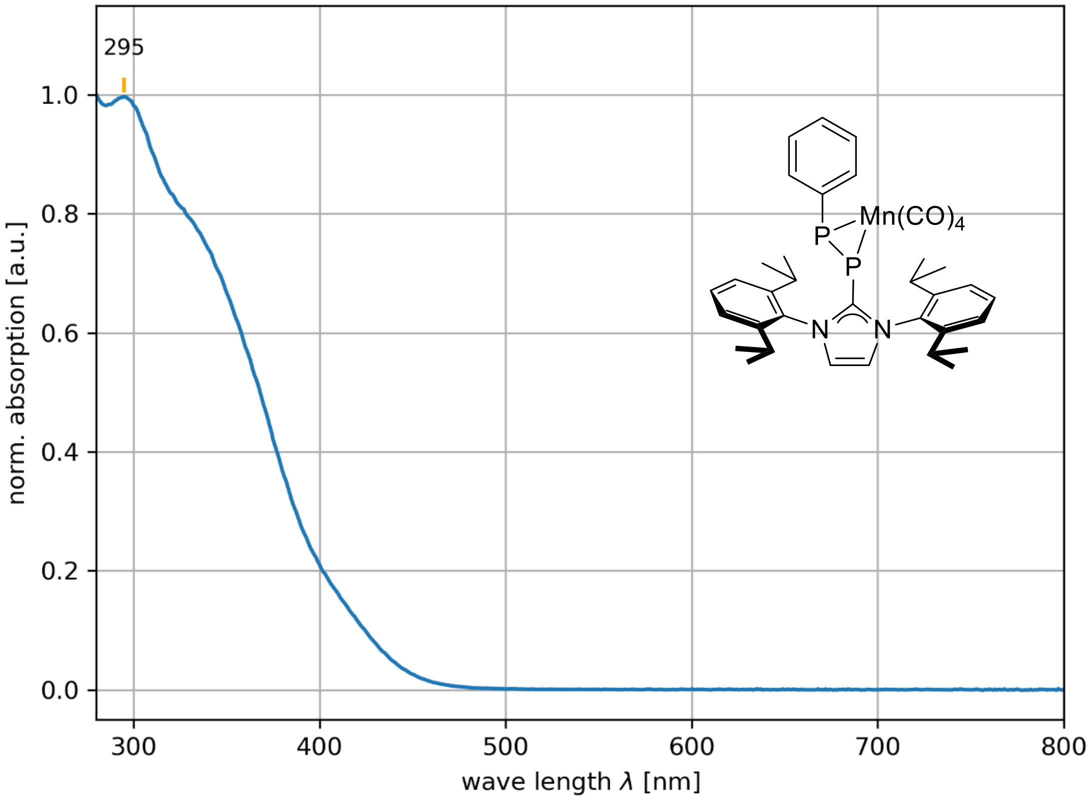

Figure S117: Normalized UV/Vis Spectrum of {(IDipp)P=PPh}Mn(CO)4 (THF).

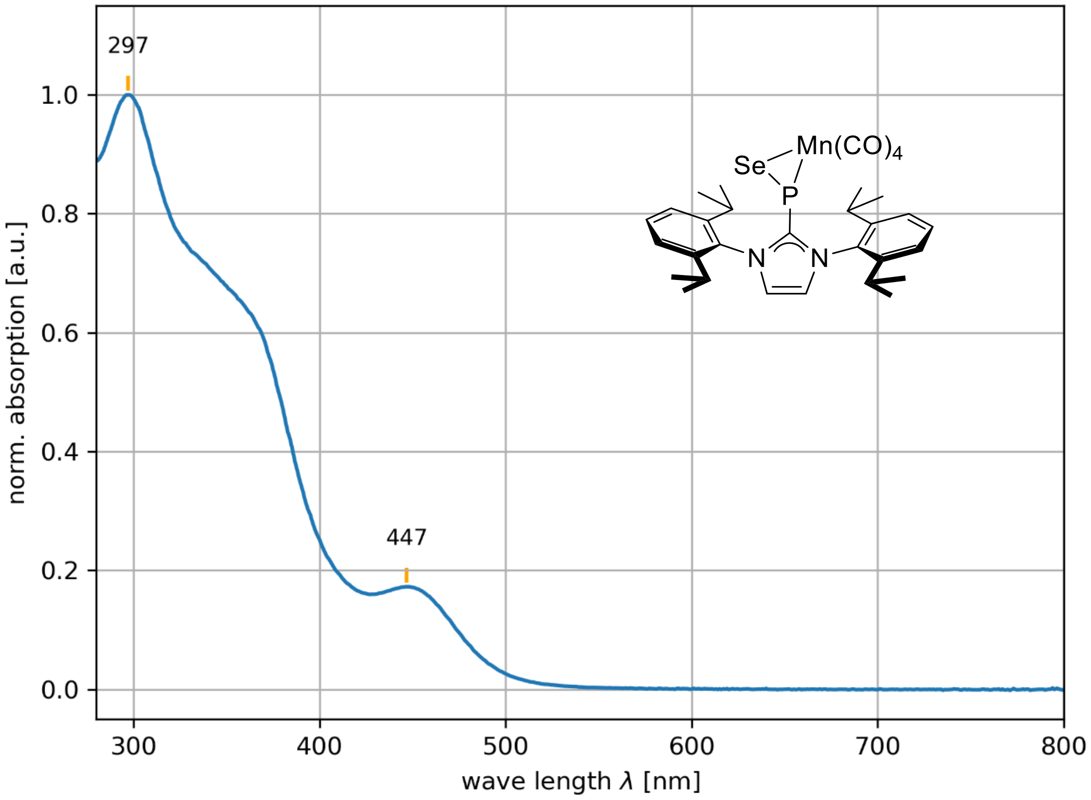

Figure S118: Normalized UV/Vis Spectrum of {(IDipp)P=Se}Mn(CO)4 (THF).

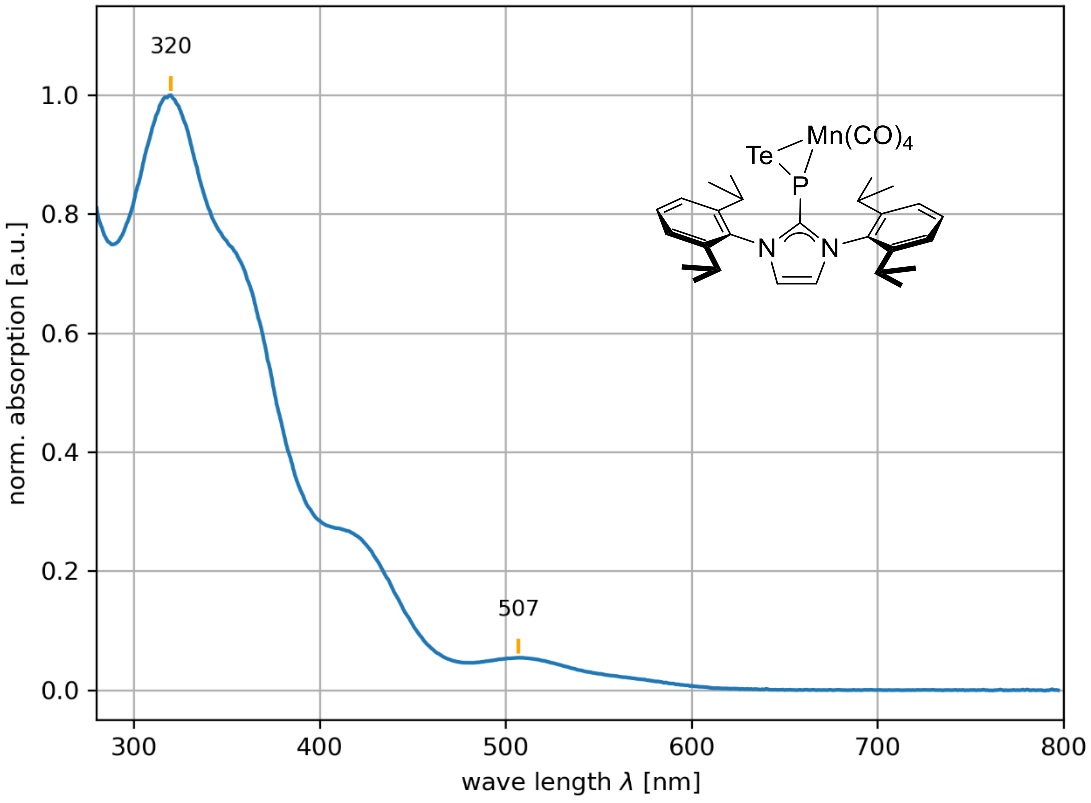

Figure S119: Normalized UV/Vis Spectrum of {(IDipp)P=Te}Mn(CO)$_4$ (THF).

S8. Single Crystal X-Ray Diffraction Analysis Details

S8.1. General

Crystals of (IDipp)PMn$(CO)_4$ were mounted on top of a human hair and all other crystals on a Hampton Research CryoLoop™ each with per-fluorinated inert oil. Data was recorded on Oxford Diffraction Xcalibur EOS diffractometers equipped with either a PhotonJet Cu-microfocus source or a PhotonJet Mo-microfocus source (s. Crystal data and structure refinement tables below) with a HyPix-6000HE detector. Data reduction was performed with CrysAlisPRO.[14] Absorption correction was based on face indexation and integration on a Gaussian grid. The structures were solved by intrinsic phasing with SHELXT-2018[15] and refined on F^2 using the program SHELXL-2018[16] in OLEX2.[17] H atoms were placed in idealized positions and refined using a riding model.

S8.2. Refinement and data handling special details are listed in the crystal data and structure refinement tables (Crystal Data and Structure Refinement Tables

Table S1–Table S10) below.

Complete data have been deposited with the Cambridge Crystallographic Data Centre under the CCDC numbers 2425952–2425961. These data can be obtained free of charge from http://www.ccdc.cam.ac.ak/.

S8.3. Crystal Data and Structure Refinement Tables

Table S1: Crystal data and structure refinement of (IDipp)PMn(CO)$_4$ • *n*-pentane.

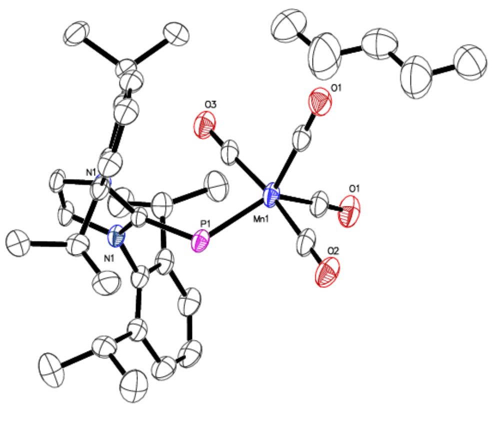	CCDC	2425952
	Temperature	100(2) K
	Wavelength	0.71073 Å
	Crystal system	monoclinic
	Space group (No.)	$P2_1/m$ (11)
	Unit Cell dimensions	
	a = 9.7422(2) Å	α = 90°
	b = 16.2881(4) Å	β = 91.998(3)°
	c = 11.3494(4) Å	γ = 90°
	Volume	1799.85(8) Å^3
	Z	2
Empirical formula	$C_{36}H_{48}MnN_2O_4P$	
Moiety formula	$C_{31}H_{36}MnN_2O_4P$, C_5H_{12}	
Formula weight	658.67	
Density (calculated)	1.215 g cm^{-3}	
Absorption coefficient	0.449 mm^{-1}	
F(000)	700	
Crystal habitus	irregular (brown)	
Crystal size	0.199 × 0.157 × 0.023 mm^3	
Θ range for	2.092 to 30.540°	
Index ranges	$-13 \leq h \leq 13$, $-23 \leq k \leq 23$, $-16 \leq l \leq 16$	
Reflections collected	11146	
Independent reflections	11146	
Completeness to Θ = 25.242°	100.0 %	
Absorption correction	gaussian	
Max. and min. transmission	1.000 and 0.657	
Data / restraints / parameters	11146 / 0 / 224	
Goodness-of-fit on F^2	1.050	
Final R indices [$I > 2\sigma(I)$]	R_1 = 0.0684, wR_2 = 0.1863	
R indices (all data)	R_1 = 0.0848, wR_2 = 0.1950	
Largest diff. peak and hole	1.04 and -0.34 e Å^{-3}	
Crystallization Details	from *n*-pentane/toluene, -40 °C	
Measurement and Refinement Details:<br><br>Refined as a 2-component twin. Component 2 rotated by 179.9657° around [-0.00 0.00 1.00] (reciprocal) or [0.04 0.00 1.00] (direct).		

Table S2: Crystal data and structure refinement of (IDipp)P(PPh_3)Mn$(CO)_3$.

<table>
<tr><td rowspan="11">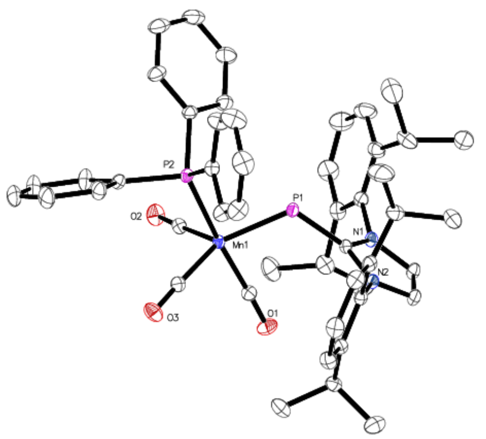
</td><td>CCDC</td><td>2425953</td></tr>
<tr><td>Temperature</td><td>100(2) K</td></tr>
<tr><td>Wavelength</td><td>0.71073 Å</td></tr>
<tr><td>Crystal system</td><td>monoclinic</td></tr>
<tr><td>Space group (No.)</td><td>$P2_1/n$ (14)</td></tr>
<tr><td colspan="2">Unit Cell dimensions</td></tr>
<tr><td>a = 14.8652(4) Å</td><td>α = 90°</td></tr>
<tr><td>b = 18.3691(5) Å</td><td>β = 97.747(2)°</td></tr>
<tr><td>c = 16.2628(5) Å</td><td>γ = 90°</td></tr>
<tr><td>Volume</td><td>4400.2(2) $Å^3$</td></tr>
<tr><td>Z</td><td>4</td></tr>
<tr><td>Empirical formula</td><td colspan="2">$C_{48}H_{51}MnN_2O_3P_2$</td></tr>
<tr><td>Moiety formula</td><td colspan="2">$C_{48}H_{51}MnN_2O_3P_2$</td></tr>
<tr><td>Formula weight</td><td colspan="2">820.78</td></tr>
<tr><td>Density (calculated)</td><td colspan="2">1.239 g cm^{-3}</td></tr>
<tr><td>Absorption coefficient</td><td colspan="2">0.415 mm^{-1}</td></tr>
<tr><td>F(000)</td><td colspan="2">1728</td></tr>
<tr><td>Crystal habitus</td><td colspan="2">block (black)</td></tr>
<tr><td>Crystal size</td><td colspan="2">0.450 × 0.210 × 0.080 mm^3</td></tr>
<tr><td>Θ range for</td><td colspan="2">2.217 to 36.071°</td></tr>
<tr><td>Index ranges</td><td colspan="2">$-22 \leq h \leq 23$, $-30 \leq k \leq 30$, $-25 \leq l \leq 25$</td></tr>
<tr><td>Reflections collected</td><td colspan="2">182357</td></tr>
<tr><td>Independent reflections</td><td colspan="2">19475 [R_{int} = 0.0486]</td></tr>
<tr><td>Completeness to Θ = 25.242°</td><td colspan="2">99.9 %</td></tr>
<tr><td>Absorption correction</td><td colspan="2">gaussian</td></tr>
<tr><td>Max. and min. transmission</td><td colspan="2">1.000 and 0.496</td></tr>
<tr><td>Data / restraints / parameters</td><td colspan="2">19475 / 0 / 513</td></tr>
<tr><td>Goodness-of-fit on F^2</td><td colspan="2">1.045</td></tr>
<tr><td>Final R indices [$I > 2\sigma(I)$]</td><td colspan="2">R_1 = 0.0330, wR_2 = 0.0874</td></tr>
<tr><td>R indices (all data)</td><td colspan="2">R_1 = 0.0421, wR_2 = 0.0909</td></tr>
<tr><td>Largest diff. peak and hole</td><td colspan="2">0.54 and -0.38 e $Å^{-3}$</td></tr>
<tr><td>Crystallization Details</td><td colspan="2">from C_6D_6/n-hexane, vapor diffusion, r.t.</td></tr>
<tr><td colspan="3">Measurement and Refinement Details: -</td></tr>
</table>

Table S3: Crystal data and structure refinement of (IDipp)P(PMe$_3$)Mn(CO)$_3$ • C_6D_6.

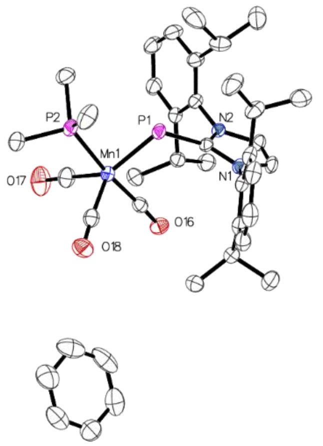

CCDC	2425954	
Temperature	100(2) K	
Wavelength	1.54184 Å	
Crystal system	monoclinic	
Space group (No.)	$P2_1/c$ (14)	
Unit Cell dimensions		
a = 12.0984(3) Å	α = 90°	
b = 14.7711(4) Å	β = 96.468(3)°	
c = 22.1799(6) Å	γ = 90°	
Volume	3938.47(18) Å^3	
Z	4	
Empirical formula	$C_{39}H_{45}D_6MnN_2O_3P_2$	
Moiety formula	$C_{33}H_{45}MnN_2O_3P_2$, C_6D_6	
Formula weight	718.73	
Density (calculated)	1.212 g cm^{-3}	
Absorption coefficient	3.774 mm^{-1}	
F(000)	1512	
Crystal habitus	irregular (black)	
Crystal size	0.070 × 0.060 × 0.020 mm^3	
Θ range for	3.602 to 80.491°	
Index ranges	$-15 \leq h \leq 15$, $-18 \leq k \leq 18$, $-28 \leq l \leq 25$	
Reflections collected	87622	
Independent reflections	8509 [R_{int} = 0.0736]	
Completeness to Θ = 67.684°	100.0 %	
Absorption correction	gaussian	
Max. and min. transmission	0.972 and 0.836	
Data / restraints / parameters	8509 / 0 / 435	
Goodness-of-fit on F^2	1.063	
Final R indices [$I > 2\sigma(I)$]	R_1 = 0.0393, wR_2 = 0.1062	
R indices (all data)	R_1 = 0.0436, wR_2 = 0.1092	
Largest diff. peak and hole	0.68 and -0.45 e Å^{-3}	
Crystallization Details	from C_6D_6/n-hexane, vapor diffusion, r.t.	
Measurement and Refinement Details: -		

Table S4: Crystal data and structure refinement of (IDipp)P(IMe)Mn(CO)$_3$ • C_6D_6.

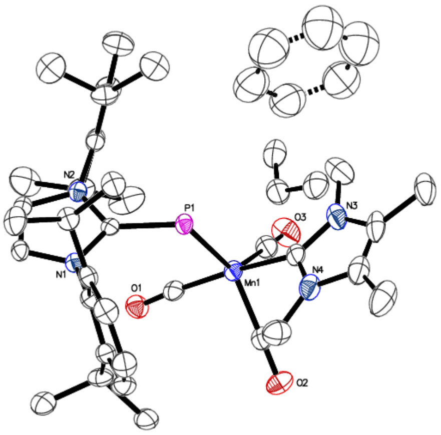

CCDC	2425955
Temperature	100(2) K
Wavelength	1.54184 Å
Crystal system	monoclinic
Space group (No.)	$C2/c$ (15)
Unit Cell dimensions	
a = 36.5788(14) Å	α = 90°
b = 11.0471(4) Å	β = 106.472(4)°
c = 20.947(2) Å	γ = 90°
Volume	8117.1(9) Å^3
Z	8
Empirical formula	$C_{43}H_{48}D_6MnN_4O_3P$
Moiety formula	$C_{37}H_{48}MnN_4O_3P$, C_6D_6
Formula weight	766.85
Density (calculated)	1.255 g cm^{-3}
Absorption coefficient	3.349 mm^{-1}
F(000)	3232
Crystal habitus	irregular (orange)
Crystal size	0.070 × 0.040 × 0.030 mm^3
Θ range for	2.519 to 76.757°
Index ranges	$-45 \leq h \leq 45$, $-12 \leq k \leq 13$, $-26 \leq l \leq 26$
Reflections collected	43492
Independent reflections	8181 [R_{int} = 0.0834]
Completeness to Θ = 67.684°	99.6 %
Absorption correction	gaussian
Max. and min. transmission	0.989 and 0.866
Data / restraints / parameters	8181 / 72 / 496
Goodness-of-fit on F^2	1.044
Final R indices [$I > 2\sigma(I)$]	R_1 = 0.0580, wR_2 = 0.1470
R indices (all data)	R_1 = 0.0846, wR_2 = 0.1623
Largest diff. peak and hole	0.82 and -0.66 e Å^{-3}
Crystallization Details	from C_6D_6/n-hexane, vapor diffusion, r.t.

Measurement and Refinement Details:

A solvent molecule C_6D_6 was found disordered over an inversion center and refined as a rigid group.

Table S5: Crystal data and structure refinement of (IDipp)P(XyNC)Mn(CO)$_3$.

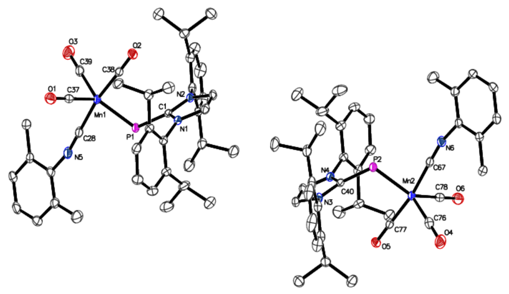

CCDC	2425956
Temperature	100(1) K
Wavelength	0.71073 Å
Crystal system	orthorhombic
Space group (No.)	$Pca2_1$ (29)
Unit Cell dimensions	
a = 19.8475(3) Å	α = 90°
b = 18.8501(2) Å	β = 90°
c = 19.5699(2) Å	γ = 90°
Volume	7321.61(16) Å^3
Z	8
Empirical formula	$C_{39}H_{45}MnN_3O_3P$
Moiety formula	$C_{39}H_{45}MnN_3O_3P$
Formula weight	689.69
Density (calculated)	1.251 g cm^{-3}
Absorption coefficient	0.444 mm^{-1}
F(000)	2912
Crystal habitus	cube (black)
Crystal size	0.220 × 0.170 × 0.110 mm^3
Θ range for	2.301 to 36.335°
Index ranges	$-29 \leq h \leq 29$, $-31 \leq k \leq 31$, $-30 \leq l \leq 31$
Reflections collected	180323
Independent reflections	30657 [R_{int} = 0.0784]
Completeness to Θ = 25.242°	99.9 %
Absorption correction	gaussian
Max. and min. transmission	1.000 and 0.626
Data / restraints / parameters	30657 / 1 / 868
Goodness-of-fit on F^2	1.066
Final R indices [$I > 2\sigma(I)$]	R_1 = 0.0435, wR_2 = 0.1117
R indices (all data)	R_1 = 0.0572, wR_2 = 0.1175
Largest diff. peak and hole	0.59 and -0.42 e Å^{-3}
Flack X parameter	0.269(12)
Crystallization Details	from benzene/n-hexane, r.t.

Measurement and Refinement Details:

Refined as a 2-component inversion twin.
The structure could also be solved in the centrosymmetric space group $Pbca$. In this case the GooF and R values increase significantly. Therefore, the non-centrosymmetric solution ($Pca2_1$) was chosen. Additionally, the structure is proven to be not strictly centrosymmetric.

Table S6: Crystal data and structure refinement of {(IDipp)P=PH}Mn(CO)$_4$.

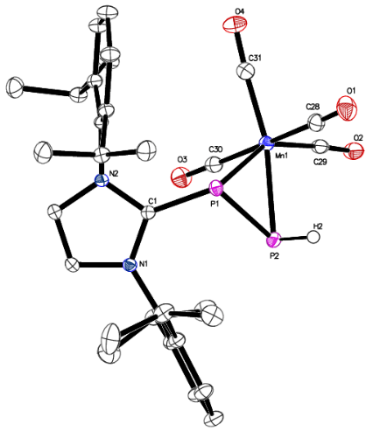	CCDC	2425957
	Temperature	100.0(9) K
	Wavelength	0.71073 Å
	Crystal system	monoclinic
	Space group (No.)	$P2_1/n$ (14)
	Unit Cell dimensions	
	a = 10.7059(3) Å	α = 90°
	b = 22.7430(5) Å	β = 95.469(2)°
	c = 13.1773(4) Å	γ = 90°
	Volume	3193.86(15) Å^3
	Z	4
Empirical formula	$C_{31}H_{37}MnN_2O_4P_2$	
Moiety formula	$C_{31}H_{37}MnN_2O_4P_2$	
Formula weight	618.50	
Density (calculated)	1.286 g cm^{-3}	
Absorption coefficient	0.550 mm^{-1}	
F(000)	1296	
Crystal habitus	block (yellow)	
Crystal size	0.430 × 0.130 × 0.060 mm^3	
Θ range for	2.110 to 38.348°	
Index ranges	$-18 \leq h \leq 18$, $-39 \leq k \leq 38$, $-22 \leq l \leq 22$	
Reflections collected	163388	
Independent reflections	17032 [R_{int} = 0.0577]	
Completeness to Θ = 25.242°	100.0 %	
Absorption correction	gaussian	
Max. and min. transmission	1.000 and 0.465	
Data / restraints / parameters	17032 / 0 / 373	
Goodness-of-fit on F^2	1.038	
Final R indices [$I > 2\sigma(I)$]	R_1 = 0.0435, wR_2 = 0.0924	
R indices (all data)	R_1 = 0.0665, wR_2 = 0.0998	
Largest diff. peak and hole	0.70 and -0.36 e Å^{-3}	
Crystallization Details	from C_6D_6/n-hexane, vapor diffusion, r.t.	
Measurement and Refinement Details:<br><br>H2 (bound to P2) was refined freely.		

Table S7: Crystal data and structure refinement of {(IDipp)P=PPh}Mn$(CO)_4$ • 0.5 C_6D_6.

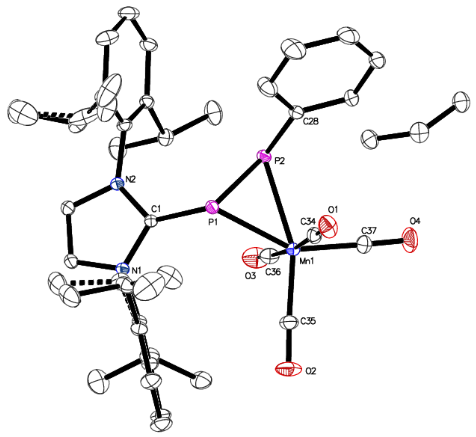	CCDC	2425958
	Temperature	99.9(8) K
	Wavelength	0.71073 Å
	Crystal system	triclinic
	Space group (No.)	$P\overline{1}$ (2)
	Unit Cell dimensions	
	a = 10.1992(4) Å	α = 85.039(2)°
	b = 10.7734(3) Å	β = 77.453(2)°
	c = 19.4589(2) Å	γ = 69.843(3)°
	Volume	1959.10(10) Å^3
	Z	2

Empirical formula	$C_{40}H_{41}D_3MnN_2O_4P_2$
Moiety formula	$C_{37}H_{41}MnN_2O_4P_2$, 0.5(C_6D_6)
Formula weight	736.67
Density (calculated)	1.249 g cm^{-3}
Absorption coefficient	0.459 mm^{-1}
F(000)	770
Crystal habitus	irregular (orange)
Crystal size	2.110 × 0.890 × 0.700 mm^3
Θ range for	4.507 to 53.894°
Index ranges	$-23 \le h \le 23$, $-24 \le k \le 24$, $-44 \le l \le 44$
Reflections collected	367825
Independent reflections	47679 [R_{int} = 0.0693]
Completeness to Θ = 25.242°	99.3 %
Absorption correction	gaussian
Max. and min. transmission	0.71971 and 0.70561
Data / restraints / parameters	47679 / 0 / 510
Goodness-of-fit on F^2	1.045
Final R indices [$I > 2\sigma(I)$]	R_1 = 0.0374, wR_2 = 0.0978
R indices (all data)	R_1 = 0.0478, wR_2 = 0.1021
Largest diff. peak and hole	0.91 and -0.74 e Å^{-3}
Crystallization Details	from C_6D_6/n-hexane, vapor diffusion, r.t.

Measurement and Refinement Details:

Two isopropyl groups were found disordered and refined accordingly.
During data reduction, frames that showed strong rings were ommitted.

Table S8: Crystal data and structure refinement of {(IDipp)P=Se}Mn(CO)$_4$.

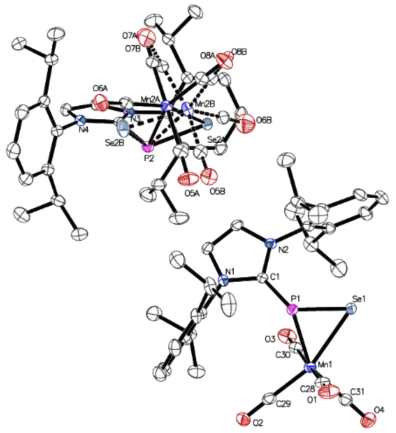

CCDC	2425959	
Temperature	100.0(6) K	
Wavelength	0.71073 Å	
Crystal system	monoclinic	
Space group (No.)	$P2_1/n$ (14)	
Unit Cell dimensions		
a = 15.0568(3) Å	α = 90°	
b = 11.0597(3) Å	β = 91.324(2)°	
c = 38.4327(9) Å	γ = 90°	
Volume	6398.2(3) Å^3	
Z	8	

Empirical formula	$C_{31}H_{36}MnN_2O_4PSe$
Moiety formula	$C_{31}H_{36}MnN_2O_4PSe$
Formula weight	665.49
Density (calculated)	1.382 g cm^{-3}
Absorption coefficient	1.636 mm^{-1}
F(000)	2736
Crystal habitus	irregular (orange)
Crystal size	0.350 × 0.230 × 0.220 mm^3
Θ range for	2.111 to 38.360°
Index ranges	$-26 \leq h \leq 26$, $-19 \leq k \leq 17$, $-66 \leq l \leq 66$
Reflections collected	338263
Independent reflections	34006 [R_{int} = 0.0871]
Completeness to Θ = 25.242°	100.0 %
Absorption correction	gaussian
Max. and min. transmission	1.000 and 0.289
Data / restraints / parameters	34006 / 128 / 828
Goodness-of-fit on F^2	1.022
Final R indices [$I > 2\sigma(I)$]	R_1 = 0.0466, wR_2 = 0.0910
R indices (all data)	R_1 = 0.0834, wR_2 = 0.1012
Largest diff. peak and hole	0.75 and -0.33 e Å^{-3}
Crystallization Details	from C_6D_6/n-hexane, vapor diffusion, r.t.

Measurement and Refinement Details:

For one of the two molecule of the asymmetric unit the Se-Mn(CO)$_4$ moiety was found disordered over two positions and refined accordingly, restraining all Se-Mn, Mn-C, Mn-O and C-O distances to be equal. The second disordered position, which is only occupied by approximately 7%, corresponds to the other enantiomer.

Table S9: Crystal data and structure refinement of {(IDipp)P=Te}Mn(CO)$_4$.

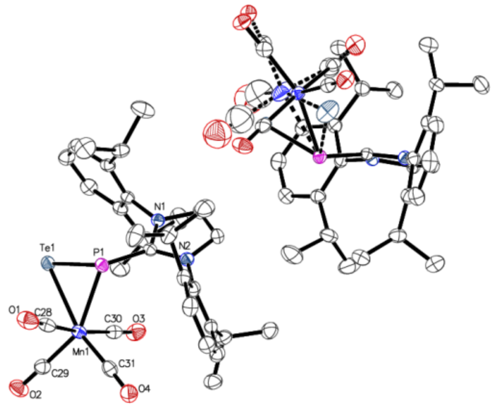

	CCDC	2425960
	Temperature	100.00(10) K
	Wavelength	0.71073 Å
	Crystal system	monoclinic
	Space group (No.)	$P2_1/n$ (14)
	Unit Cell dimensions	
	a = 15.3254(2) Å	α = 90°
	b = 11.11930(10) Å	β = 90.2890(10)°
	c = 38.2883(4) Å	γ = 90°
	Volume	6524.54(12) Å^3
	Z	8
Empirical formula	$C_{31}H_{36}MnN_2O_4PTe$	
Moiety formula	$C_{31}H_{36}MnN_2O_4PTe$	
Formula weight	714.13	
Density (calculated)	1.454 g cm^{-3}	
Absorption coefficient	1.366 mm^{-1}	
F(000)	2880	
Crystal habitus	irregular (red)	
Crystal size	0.140 × 0.100 × 0.090 mm^3	
Θ range for	2.071 to 30.567°	
Index ranges	$-21 \leq h \leq 21$, $-15 \leq k \leq 15$, $-54 \leq l \leq 54$	
Reflections collected	207791	
Independent reflections	19953 [R_{int} = 0.0970]	
Completeness to Θ = 25.242°	100.0 %	
Absorption correction	gaussian	
Max. and min. transmission	1.000 and 0.776	
Data / restraints / parameters	19953 / 89 / 828	
Goodness-of-fit on F^2	1.072	
Final R indices [$I > 2\sigma(I)$]	R_1 = 0.0471, wR_2 = 0.1109	
R indices (all data)	R_1 = 0.0643, wR_2 = 0.1175	
Largest diff. peak and hole	1.70 and -0.80 e Å^{-3}	
Crystallization Details	from C_6D_6/n-hexane, vapor diffusion, r.t.	

Measurement and Refinement Details:

For one of the two molecule of the asymmetric unit the Te-Mn(CO)$_4$ moiety was found disordered over two positions and refined accordingly, restraining all Te-Mn, Mn-C, Mn-O and C-O distances to be equal. The second disordered position, which is only occupied by approximately 11%, corresponds to the other enantiomer.

Table S10: Crystal data and structure refinement of [(IDipp)H][{(IDipp)P(μ_2-PH)}$Mn_2(CO)_8$].

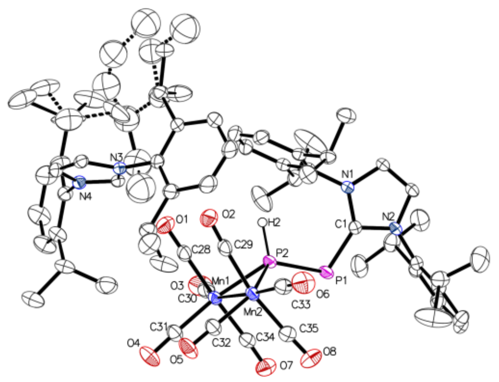	CCDC	2425961
	Temperature	99.9(7) K
	Wavelength	0.71073 Å
	Crystal system	monoclinic
	Space group (No.)	$C2/c$ (15)
	Unit Cell dimensions	
	a = 42.5367(12) Å	α = 90°
	b = 12.4663(4) Å	β = 95.701(3)°
	c = 25.9108(8) Å	γ = 90°
	Volume	13671.9(7) Å^3
	Z	8

Empirical formula	$C_{68}H_{88}Mn_2N_4O_8P_2$
Moiety formula	$C_{35}H_{37}Mn_2N_2O_8P_2$,$C_{27}H_{36}N_2$,$C_6H_{14}$
Formula weight	1261.24
Density (calculated)	1.225 g cm^{-3}
Absorption coefficient	0.470 mm^{-1}
F(000)	5344
Crystal habitus	irregular (orange)
Crystal size	0.250 × 0.133 × 0.057 mm^3
Θ range for	2.180 to 30.537°
Index ranges	$-60 \leq h \leq 60$, $-17 \leq k \leq 17$, $-37 \leq l \leq 37$
Reflections collected	221693
Independent reflections	20901 [R_{int} = 0.0900]
Completeness to Θ = 25.242°	99.9 %
Absorption correction	gaussian
Max. and min. transmission	1.000 and 0.599
Data / restraints / parameters	20901 / 117 / 838
Goodness-of-fit on F^2	1.105
Final R indices [$I > 2\sigma(I)$]	R_1 = 0.0740, wR_2 = 0.1876
R indices (all data)	R_1 = 0.0997, wR_2 = 0.1995
Largest diff. peak and hole	1.00 and -0.71 e Å^{-3}
Crystallization Details	from C_6D_6/n-hexane, vapor diffusion, rt
Measurement and Refinement Details:<br><br>One isopropyl group and the molecule n-hexane were each found to be disorderd over two positions and refined accordingly. H2 (bound to P2) was refined freely.	

S8.4. Molecular Structures and Structural Details

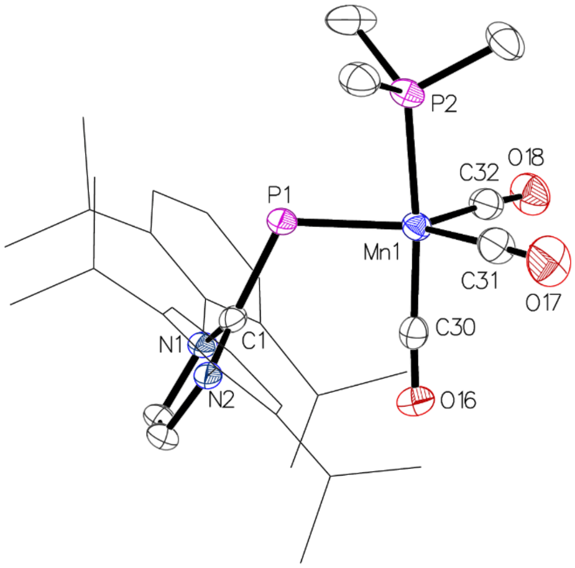

Figure S120: Molecular structure of (IDipp)P(PMe$_3$)Mn(CO)$_3$ • C_6D_6 with thermal displacement parameters drawn at the 50% probability level. Hydrogen atoms and the solvent molecule were omitted for clarity. Dipp groups are displayed as wireframe. Selected interatomic distances and angles are displayed in Table 1 and Table S10.

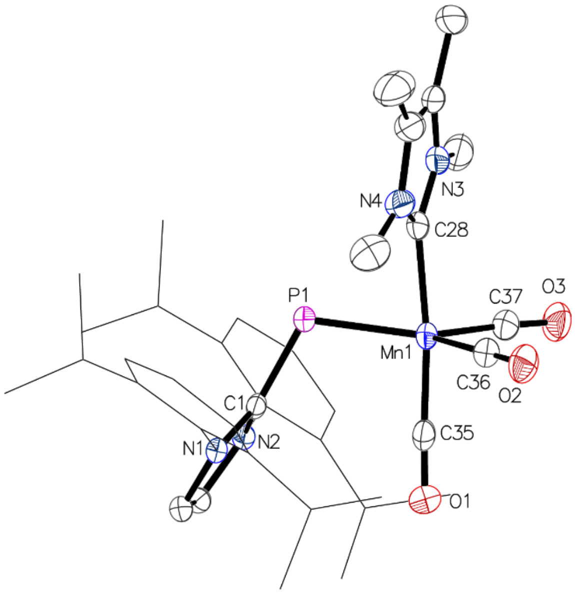

Figure S121: Molecular structure of (IDipp)P(IMe)Mn(CO)$_3$ • C_6D_6 with thermal displacement parameters drawn at the 50% probability level. Hydrogen atoms and the solvent molecule were omitted for clarity. Dipp groups are displayed as wireframes. Selected interatomic distances and angles are displayed in Table 1 and Table S10.

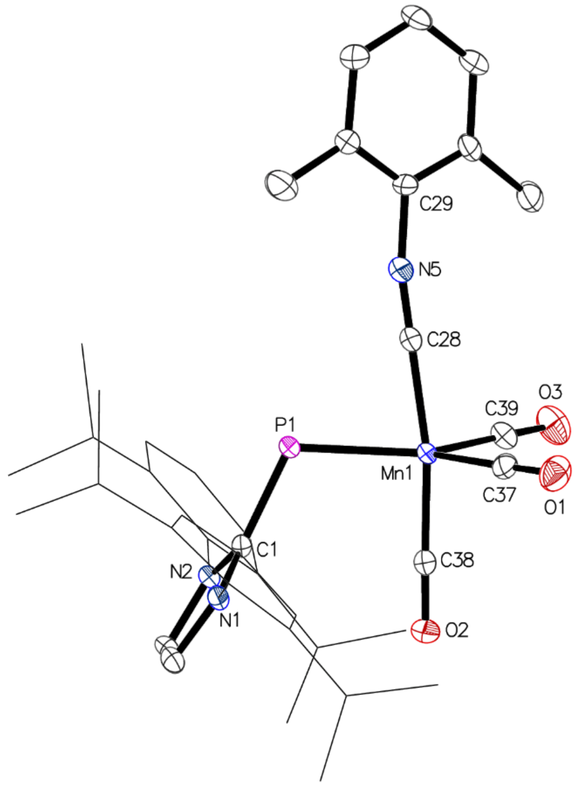

Figure S122: Molecular structure of (IDipp)P(XyNC)Mn(CO)3 with thermal displacement parameters drawn at the 50% probability level. Hydrogen atoms and the second molecule in the asymmetric unit were omitted for clarity. Dipp groups are displayed as wireframes. Selected interatomic distances and angles are displayed in Table 1 and Table S10.

Table S11: Comparison of selected interatomic distances and angles in the molecular structures of (IDipp)(L)Mn(CO)3 • solv (L = CO (solv = *n*-pentane), PPh3, PMe3 (solv = C6D6), IMe (solv = C6D6), XyNC).

***d* [Å] / ∡ [°]**	**L = CO[a]**	**L = PPh3**	**L = PMe3**	**L = IMe**	**L = XyNC[b]**
Mn–P	2.1904(11)	2.1805(3)	2.1939(5)	2.2136(8)	2.1679(9)/2.1673(9)
Mn–L	1.848(4)	2.2873(2)	2.2965(5)	2.064(3)	1.861(3)/1.874(3)
Mn–CO(eq1)	1.801(3)	1.7877(8)	1.783(2)	1.782(3)	1.831(3)/1.818(3)
Mn–CO(eq2)	1.801(3)	1.7898(8)	1.792(2)	1.787(4)	1.829(3)/1.838(3)
Mn–CO(ax)	1.820(4)	1.7935(9)	1.7907(18)	1.792(3)	1.757(2)/1.755(2)
C–O(eq1)	1.156(4)	1.1594(10)	1.159(3)	1.160(4)	1.177(3)/1.179(3)
C–O(eq2)	1.156(4)	1.1591(10)	1.155(2)	1.163(4)	1.164(3)/1.179(3)
C–O(ax)	1.155(5)	1.1512(11)	1.156(2)	1.154(4)	1.110(3)/1.128(3)
C–O(= L)	1.151(5)	–	–	–	–
C–N	–	–	–	–	1.149(3)/1.137(3)
P–C1[c]	1.822(3)	1.8123(8)	1.8169(17)	1.815(3)	1.743(2)/1.740(2)
C1–P–Mn [c]	114.05(12)	113.47(3)	113.83(5)	112.81(9)	112.93(9)/112.75(9)
P–Mn–L	84.16(15)	84.777(8)	83.932(17)	81.75(9)	79.50(8)/79.56(8)
P–Mn–CO(ax)	88.85(14)	90.71(3)	90.79(5)	93.92(10)	91.66(8)/91.95(8)
L–Mn–CO(ax)	173.0(2)	175.12(3)	173.60(6)	170.67(16)	171.09(11)/171.49(11)

CO(eq1)–Mn–CO(eq2)	98.3(2)	100.24(4)	93.88(9)	92.45(15)	96.34(12)/96.12(12)
P–Mn–CO(eq1)	130.84(10)	128.36(3)	136.52(7)	137.46(11)	131.62(8)/131.53(9)
P–Mn–CO(eq2)	130.84(10)	131.07(3)	129.51(6)	129.76(11)	131.74(9)/131.93(8)
Σ(∡$_{eq}$)	360.0(3)	359.67(6)	359.91(13)	359.7(2)	359.70(16)/359.58(16)

[a] Symmetrically equivalent atoms were generated using the following transformation: (x, ½-y, z). [b] Two crystallographically independent molecules in the asymmetric unit. [c] C1 = C(carbene)

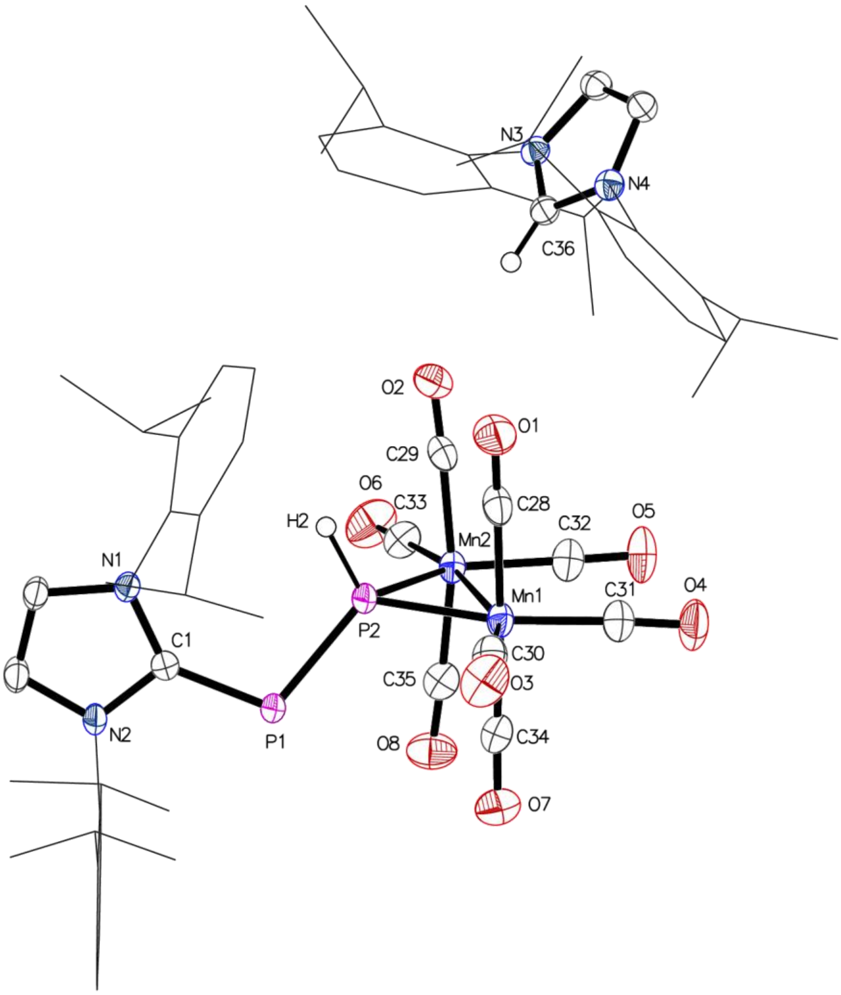

Figure S123: Molecular structure of [(IDipp)H][μ2-{(IDipp)PPH}Mn2(CO)8] with thermal displacement parameters drawn at the 50% probability level. Hydrogen atoms except for the ones bound to P2 (H2) and to C36 were omitted for clarity. Dipp groups are displayed as wireframes. Selected interatomic distances [Å] and angles [°] are listed below: P1–P2 2.1848(10), P2–Mn1 2.2745(8), P2–Mn2 2.2753(8), Mn1–Mn2 2.8584(6), P1–C1 1.775(3), P2–H2 1.33(4), C1–P1–P2 108.20(9), P1–P2–Mn1 117.66(4), P1–P2–Mn2 123.62(2), Mn1–P2–Mn2 77.84(3).

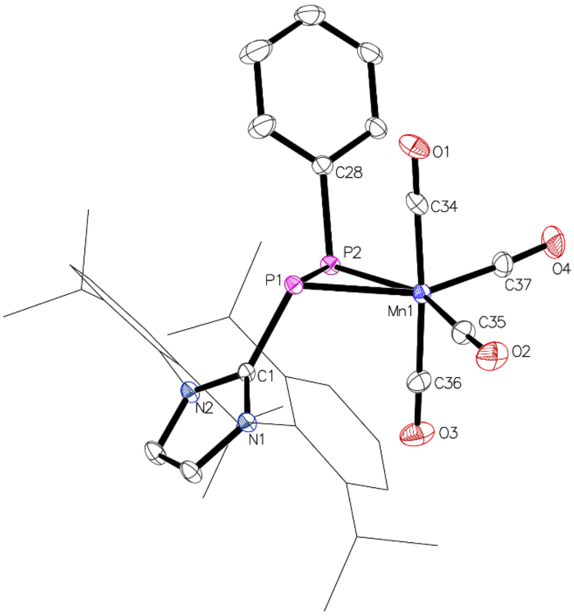

Figure S124: Molecular structure of {(IDipp)P=PPh}Mn(CO)$_4$ • 0.5 C_6D_6 with thermal displacement parameters drawn at the 50% probability level. Hydrogen atoms, disordered isopropyl groups and the solvent molecule were omitted for clarity. Dipp groups are displayed as wireframe. Selected interatomic distances and angles are displayed in Table 2 and Table S11.

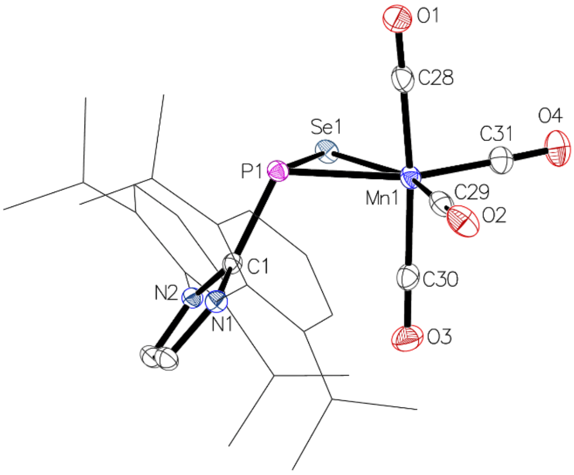

Figure S125: Molecular structure of {(IDipp)P=Se}Mn(CO)$_4$ with thermal displacement parameters drawn at the 50% probability level. Hydrogen atoms and second molecule in the asymmetric unit were omitted for clarity. Dipp groups are displayed as wireframe. Selected interatomic distances and angles are displayed in Table 2 and Table S11.

Table S12: Comparison of selected interatomic distances and angles in the molecular structures of (IDippP=E)Mn(CO)$_4$ • solv (E = PR (R = H, Ph (solv = 0.5 C_6D_6)), Se, Te). CO(aE1) occupies the opposite site compared to NHC; CO(eq1) is the carbonyl ligand that is closest to P1; CO(eq2) is closest to E.

d **[Å] / ∡ [°]**	**E = PH**	**E = PPh**	**E = Se[c]**	**E = Te[c]**
Mn–P1[a]	2.3806(3)	2.37793(18)	2.3691(4)	2.3844(9)
Mn–E	2.4148(3)	2.4111(2)	2.5103(3)	2.6843(4)
P1–E[a]	2.1612(4)	2.1486(2)	2.2094(4)	2.4311(8)
E–R	1.328(9)	1.8369(4)	–	–
P1–C1[a,b]	1.8323(10)	1.8274(4)	1.8433(12)	1.841(3)
Mn–CO(eq1)	1.8106(12)	1.8169(4)	1.7980(16)	1.794(3)
Mn–CO(eq2)	1.8001(11)	1.8012(5)	1.8210(15)	1.819(3)
Mn–CO(ax1)	1.8464(12)	1.8303(5)	1.8484(15)	1.845(4)
Mn–CO(ax2)	1.8396(11)	1.8505(5)	1.8557(15)	1.864(3)
C–O(eq1)	1.1520(14)	1.1504(5)	1.1501(19)	1.152(4)
C–O(eq2)	1.1550(13)	1.1515(6)	1.1479(18)	1.143(4)
C–O(ax1)	1.1434(15)	1.1477(6)	1.1373(18)	1.141(4)
C–O(ax2)	1.1471(14)	1.1441(7)	1.1408(18)	1.134(4)
C1–P1–Mn [a,b]	110.96(3)	114.084(14)	113.35(4)	114.42(10)
P1–Mn–E	53.567(10)	53.309(6)	53.754(11)	56.95(2)
Mn–P1–E	66.022(16)	64.136(13)	66.39(2)	67.75(3)
Mn–E–P1	62.412(16)	62.555(14)	59.86(2)	55.30(3)
C1–P1–E[a,b]	102.67(3)	106.02(2)	107.46(4)	108.58(9)
CO(ax1)–Mn–CO(ax2)	176.65(5)	173.99(2)	174.53(7)	172.70(14)
CO(eq1)–Mn–CO(eq2)	101.38(5)	99.86(3)	99.36(7)	98.5(15)
P1–Mn–CO(eq1)[a]	109.49(4)	108.49(2)	108.14(5)	108.59(11)
E–Mn–CO(eq2)	95.58(3)	99.95(2)	99.04(5)	96.25(10)

[a] P1 = *P*–C(carbene), [b] C1 = *C*(carbene), [c] only parameters of the component which does not show severe disorder are listed

S9. Kinetic Studies

S9.1. Theory

The following reaction was studied kinetically

$$(IDipp)PMn(CO)_4 + PPh_3 \rightarrow (IDipp)PMn(PPh_3)(CO)_3 + CO$$

where [Mn] = *c*((IDipp)PMn($CO)_4$), [L] = *c*(PPh_3) and [MnL] = *c*((IDipp)P(PPh_3)Mn($CO)_3$) describe the respective concentrations in solution. Assuming this process is a second order kinetic, the rate *r* of the reaction is given by

$$r = -\frac{d[\mathrm{Mn}]}{dt} = \frac{d[\mathrm{MnL}]}{dt} = k[\mathrm{Mn}][\mathrm{L}] \tag{1}$$

where *m* and *n* are the reaction orders in respect to the corresponding reactant.

When a large excess of PPh_3 is used ([L] >> [Mn]), the concentration of the ligand can be assumed to be constant ([L] ≈ const.) and equation (1) can be simplified to

$$-\frac{d[\mathrm{Mn}]}{dt} = k'[\mathrm{Mn}] \tag{2}$$

with

$$k' = k\,[L] \tag{3}$$

For a (pseudo) first order reaction, equation (2 can be solved and the integrated rate law can be stated as follows

$$\int_{[\mathrm{Mn}]_0}^{[\mathrm{Mn}]_t} \frac{d[\mathrm{Mn}]}{[\mathrm{Mn}]} = -\int_{t=0}^{t} k'\,dt \tag{4}$$

$$\ln([\mathrm{Mn}]_t) - ln([\mathrm{Mn}]_0) = -k't \tag{5}$$

The concentration [MnL] can be described by the physical property absorbance *A* as described below[18]

$$[\mathrm{MnL}]_t = [\mathrm{Mn}]_0(A_t - A_0)/(A_\infty - A_0) \tag{6}$$

expression of concentration[18] in terms of physical properties leads to

$$\ln(A_\infty - A_t) = -k't + \ln(A_\infty - A_0) \tag{7}$$

Accordingly, k' can be determined by a linear fit of $\ln(|A_\infty - A_t|)$ vs. time *t*.

For a second order reaction equation (3 must hold true. Therefore, a plot of k' vs. [L] should result in a line with the slope k.

The conversion *X*(Mn) is given by

$$X(\mathrm{Mn}) = \frac{X(\mathrm{Mn})_t}{X(\mathrm{Mn})_0} \quad (8)$$

with $X(\mathrm{Mn})_0 \approx X(\mathrm{Mn})_{t1}$ (t1 = first measurement).

S9.2. Experiments

Kinetic studies were conducted IR-spectroscopically under pseudo first order conditions ([L] ≥ 10 eq.) using a cuvette of an approximately 1 mm optical path with NaCl windows (V = 0.26 mL). Kinetic runs were repeated at least three times (s. Table S13: Measurement parameters of kinetic runs. for measurement parameters). THF was used as the solvent for the experiments because no interference or measurement artifacts from aromatic overtones were observed this way (which happened when using toluene as a solvent). Backgrounds were measured with a solution of PPh_3 in THF with the respective ligand concentration. Methodology was similar to what has been reported by *Basolo et al.*[19]

Sample Preparation: Inside a glove box, 80 µL of a stock solution of (IDipp)PMn$(CO)_4$ (0.0125 M) were diluted with the respective amount of solvent. Afterwards, the according volume of a stock solution of the ligand was added in one portion. The reaction mixture was transferred to the cuvette, which was removed from the glove box and placed into the spectrometer. The measurement was started and a number of spectra ("Number of measurements") was recorded each after a waiting period ("Time between measurements"). The time between mixing of the reagents and start of the spectroscopic measurement was recorded as Δt_0. Assignment and comparison of IR bands of both product and reactant is shown in Figure S126.

The rate of the reaction $r = \frac{d[\mathrm{Mn}]}{dt}$ was monitored by observation of the maximum (determined with *NumPy*)[8] absorbance A_t between 2040 and 2030 nm (carbonyl band of (IDipp)PMn$(CO)_4$) (Absorbance over time data are shown in

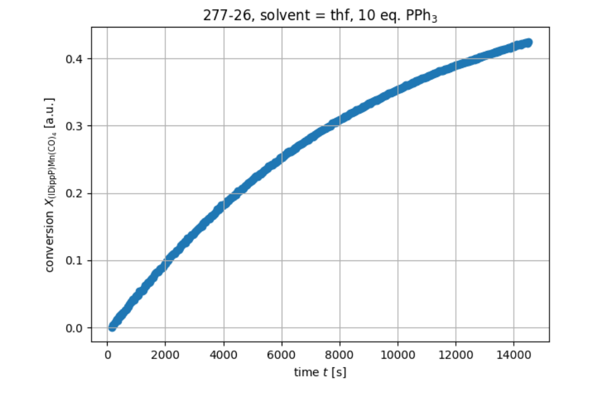
277-26, solvent = thf, 10 eq. PPh_3
conversion $X_{(IDippP)Mn(CO)_4}$ [a.u.]
time t [s]

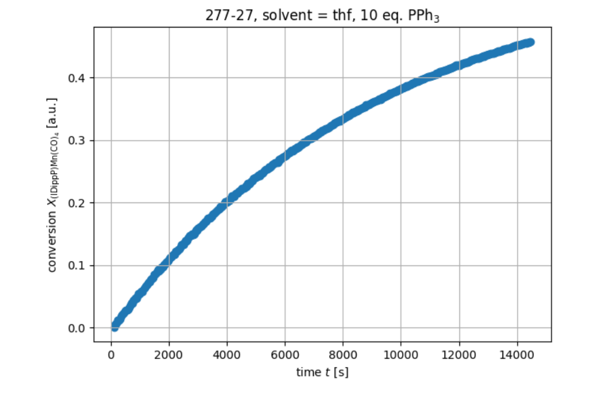
277-27, solvent = thf, 10 eq. PPh_3
conversion $X_{(IDippP)Mn(CO)_4}$ [a.u.]
time t [s]

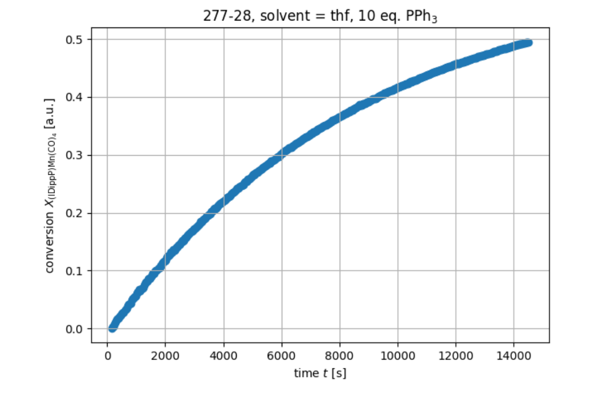
277-28, solvent = thf, 10 eq. PPh_3
conversion $X_{(IDippP)Mn(CO)_4}$ [a.u.]
time t [s]

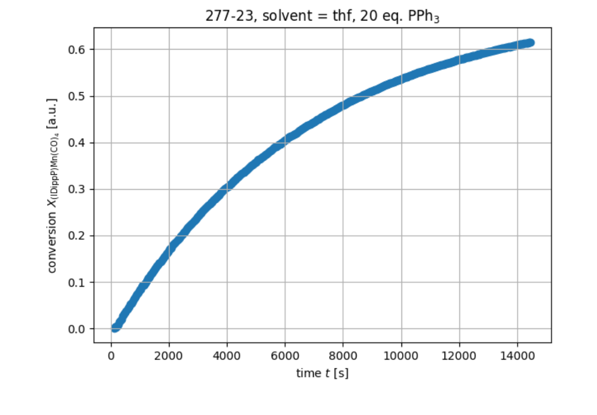
277-23, solvent = thf, 20 eq. PPh_3
conversion $X_{(IDippP)Mn(CO)_4}$ [a.u.]
time t [s]

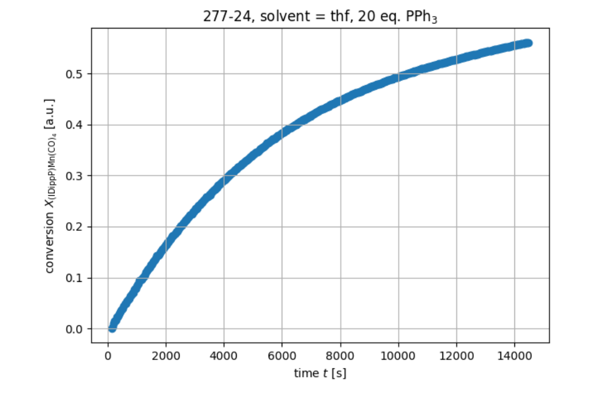
277-24, solvent = thf, 20 eq. PPh_3
conversion $X_{(IDippP)Mn(CO)_4}$ [a.u.]
time t [s]

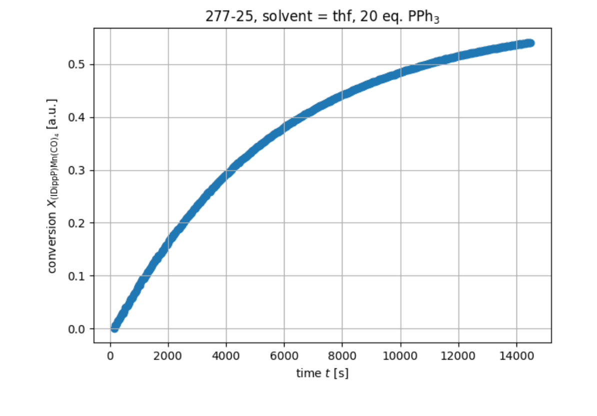
277-25, solvent = thf, 20 eq. PPh_3
conversion $X_{(IDippP)Mn(CO)_4}$ [a.u.]
time t [s]

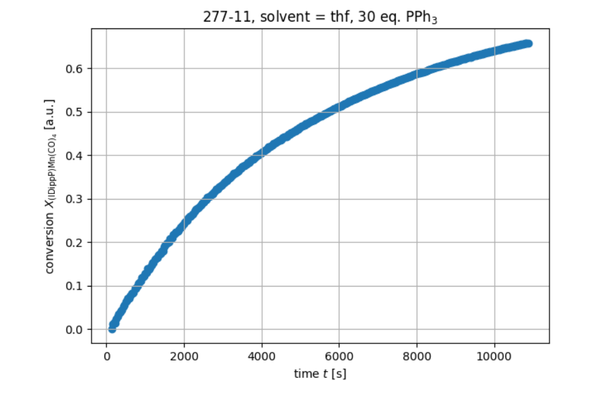
277-11, solvent = thf, 30 eq. PPh_3
conversion $X_{(IDippP)Mn(CO)_4}$ [a.u.]
time t [s]

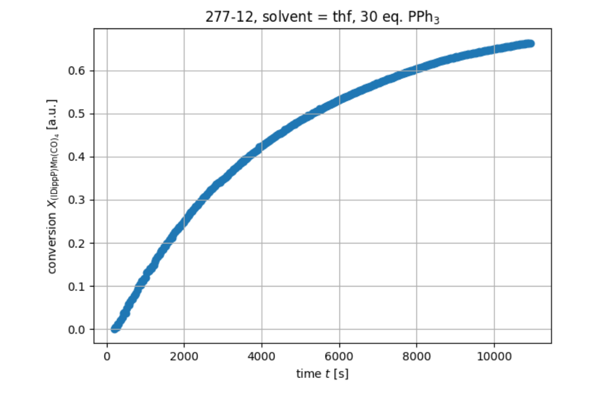
277-12, solvent = thf, 30 eq. PPh_3
conversion $X_{(IDippP)Mn(CO)_4}$ [a.u.]
time t [s]

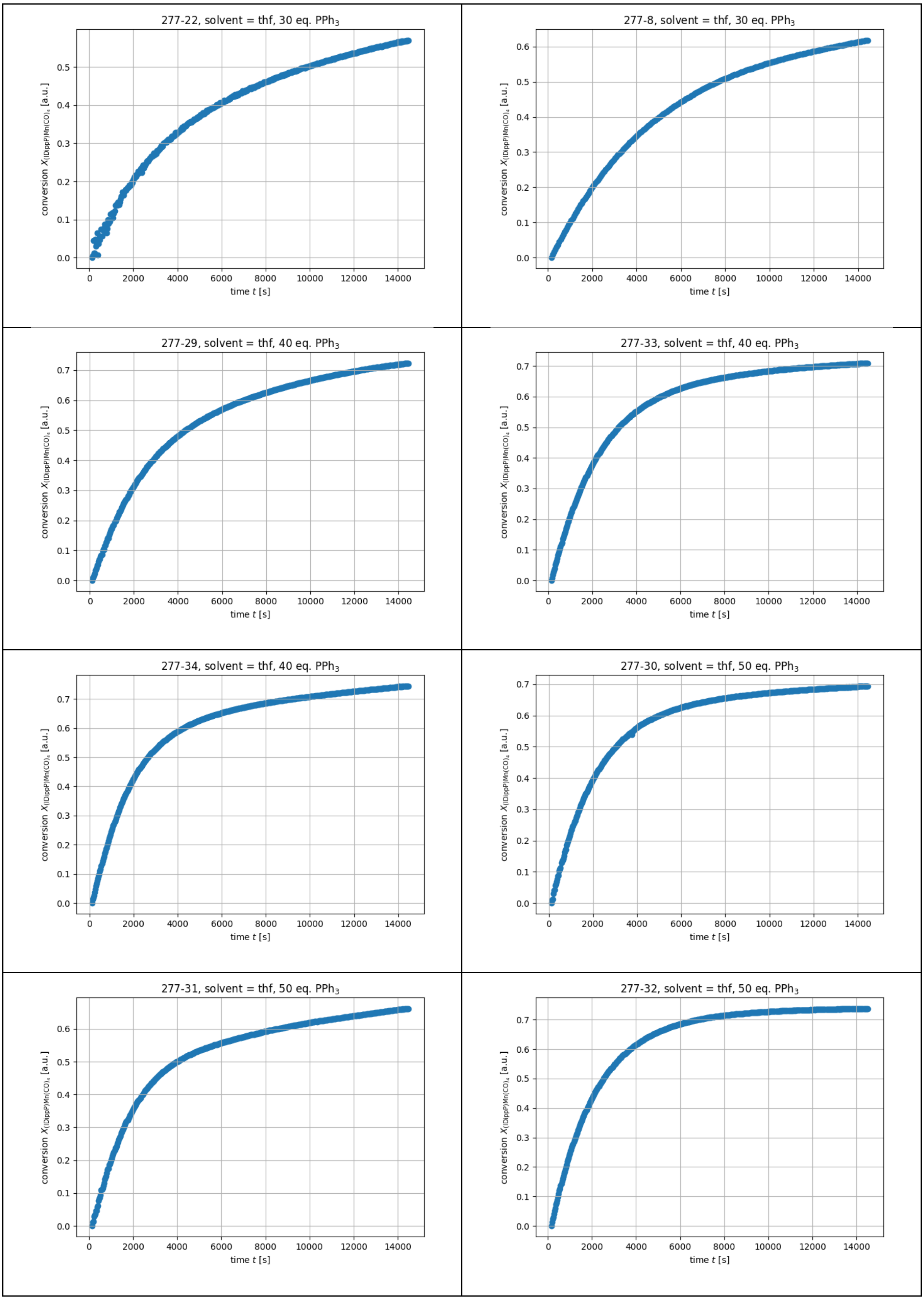
277-22, solvent = thf, 30 eq. PPh$_3$
277-8, solvent = thf, 30 eq. PPh$_3$
277-29, solvent = thf, 40 eq. PPh$_3$
277-33, solvent = thf, 40 eq. PPh$_3$
277-34, solvent = thf, 40 eq. PPh$_3$
277-30, solvent = thf, 50 eq. PPh$_3$
277-31, solvent = thf, 50 eq. PPh$_3$
277-32, solvent = thf, 50 eq. PPh$_3$
conversion $X_{(IDippP)Mn(CO)_4}$ [a.u.]
time t [s]

Table S15, conversion graphs are shown in Table S14). A_∞ was determined as the last recorded datapoint. $\ln(|A_\infty - A_t|)$ was plotted vs. time and the data was approximated by appliance of a linear fit from 0 to 9000 s using the Stats module from the SciPy library (s. Table S16).[11] They all gave good linear regression results with $R^2 > 0.97$. The mean value of each this way determined apparent rate constants k' (s. Table S17) with their respective standard deviations $\sigma(k')$ (determined with the *mean* and *std* functions from the *Numpy* library[8]) were plotted vs. the respective concentration of PPh_3 (s. Figure S127). A regression proved linear correlation with $R^2 > 0.99$. The deviation of the ligand concentration was evaluated by introduction of a systematic error of +0.5 mol/L and +1 mol/L. The resulting data points and linear regression are shown in Figure S128. With increasing concentration error, the y-intercept approaches zero. This has no effect on the slope (= rate constant k) of the fit. The non-zero y-intercept can therefore be regarded as an artifact of the concentration determination error.

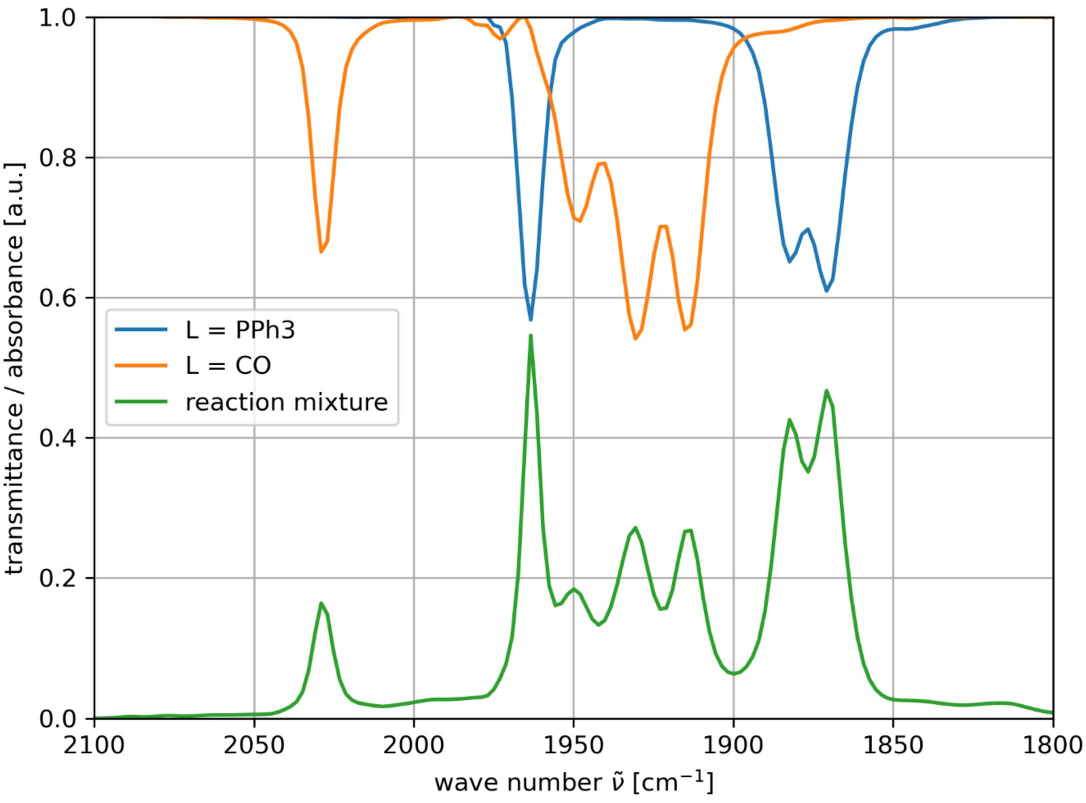

Figure S126: Comparison between reactant (L = CO) and product (L = PPh_3) IR spectra of (IDipp)P(L)$Mn(CO)_4$ (top) as well as the reaction mixture (bottom).

Table S13: Measurement parameters of kinetic runs.

Experiment number	Equivalents PPh_3	µL stock solution PPh_3 (0.5 M)	µL THF	µL stock solution [(IDipp)PMn$(CO)_4$] (0.0125 M)	Δt_0 [s]	Number of measurements	Time between measurements [s]
277-26	10	20	140	80	156	480	30
277-27	10	20	140	80	118	480	30
277-28	10	20	140	80	155	480	30
277-23	20	40	120	80	114	480	30

277-24	20	40	120	80	137	480	30
277-25	20	40	120	80	141	480	30
277-11	30	60	100	80	135	360	30
277-12	30	60	100	80	195	360	30
277-22	30	60	100	80	130	480	30
277-8	30	60	100	80	150	240	60
277-29	40	80	80	80	105	480	30
277-33	40	80	80	80	146	480	30
277-34	40	80	80	80	119	480	30
277-30	50	100	60	80	112	480	30
277-31	50	100	60	80	138	480	30
277-32	50	100	60	80	163	480	30

Table S14: Conversion plots for the different kinetic runs.

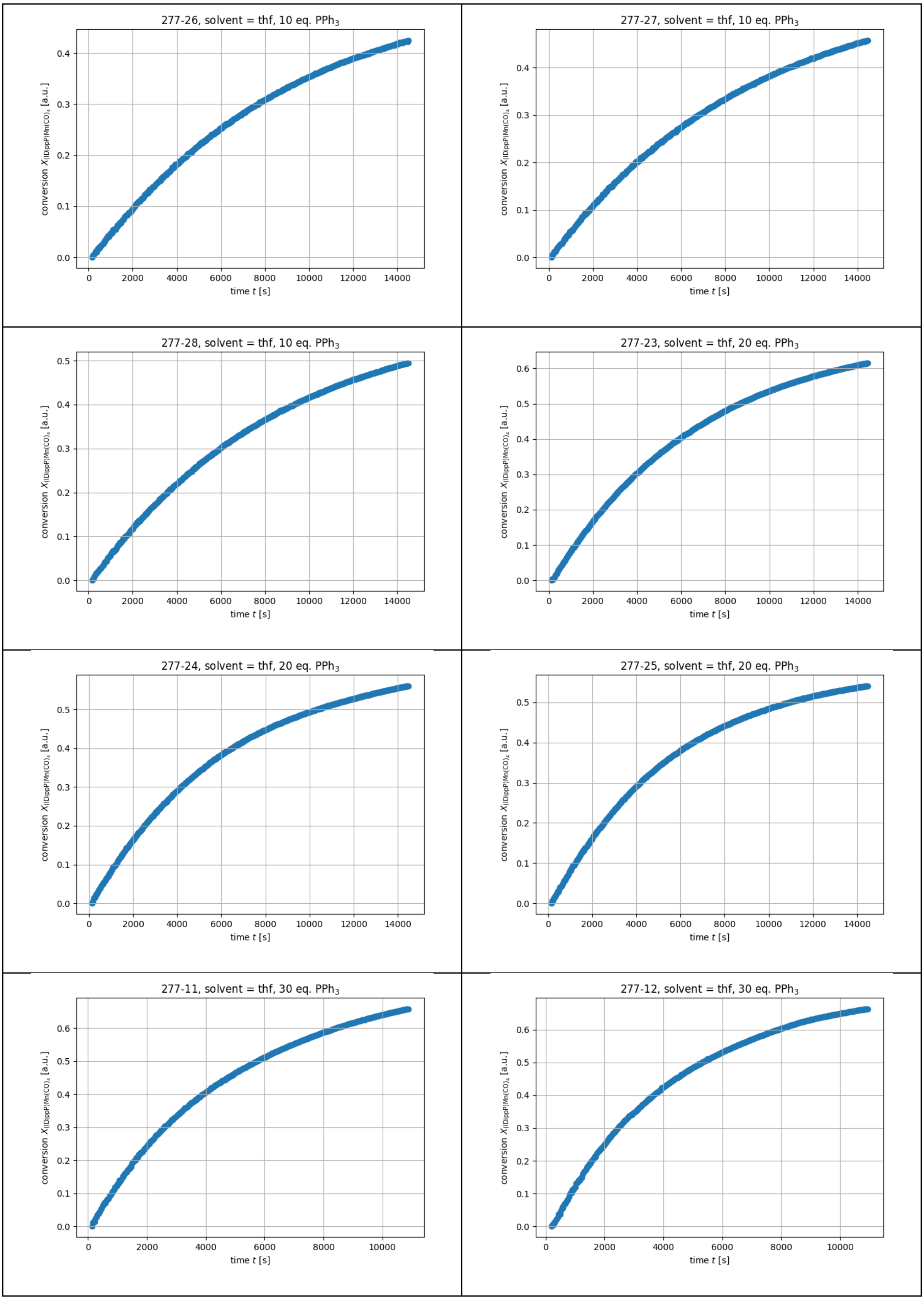

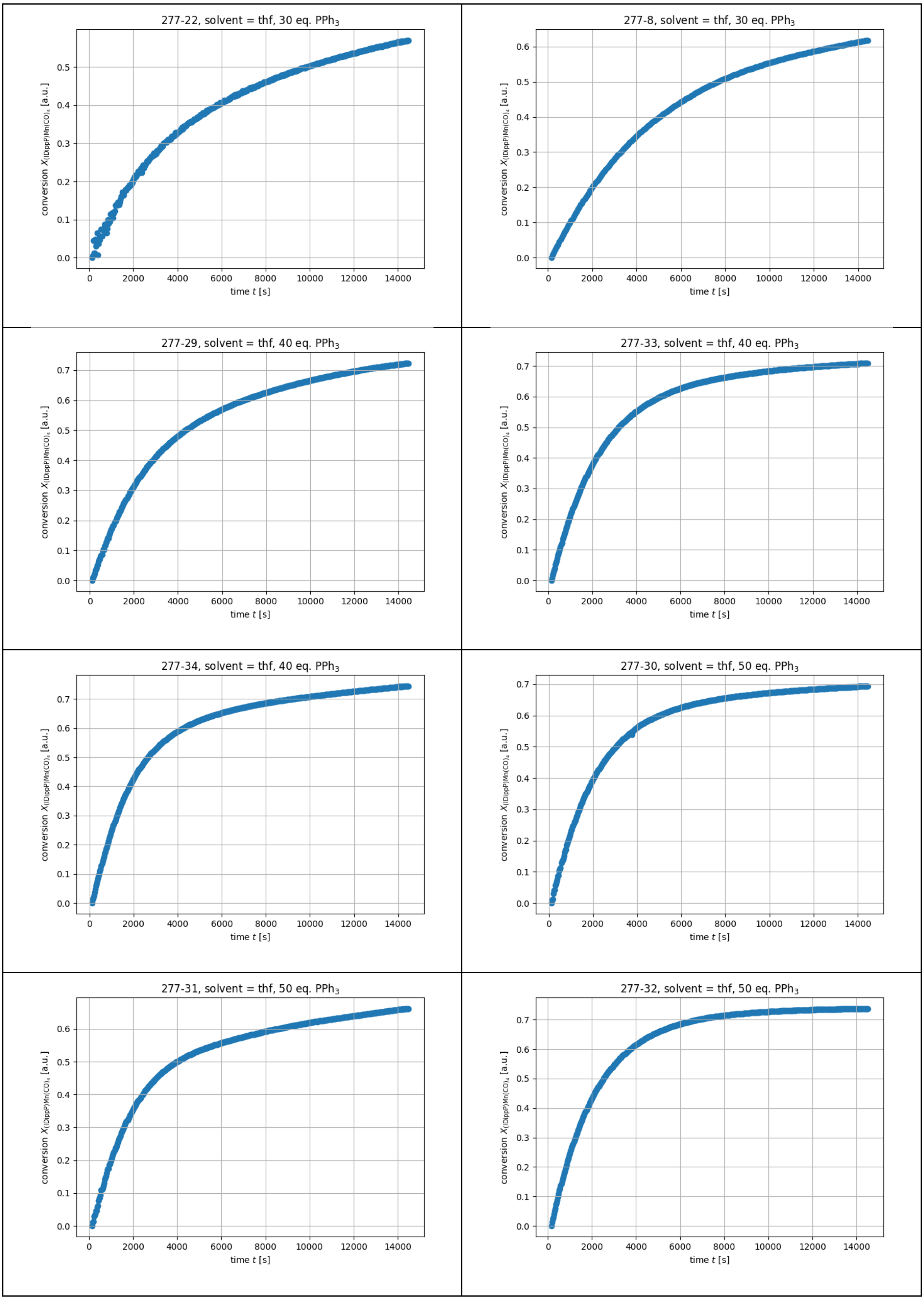
277-22, solvent = thf, 30 eq. PPh3
277-8, solvent = thf, 30 eq. PPh3
277-29, solvent = thf, 40 eq. PPh3
277-33, solvent = thf, 40 eq. PPh3
277-34, solvent = thf, 40 eq. PPh3
277-30, solvent = thf, 50 eq. PPh3
277-31, solvent = thf, 50 eq. PPh3
277-32, solvent = thf, 50 eq. PPh3
conversion X(IDippP)Mn(CO)4 [a.u.]
time t [s]

Table S15: Absorbance over time plots for the different kinetic runs.

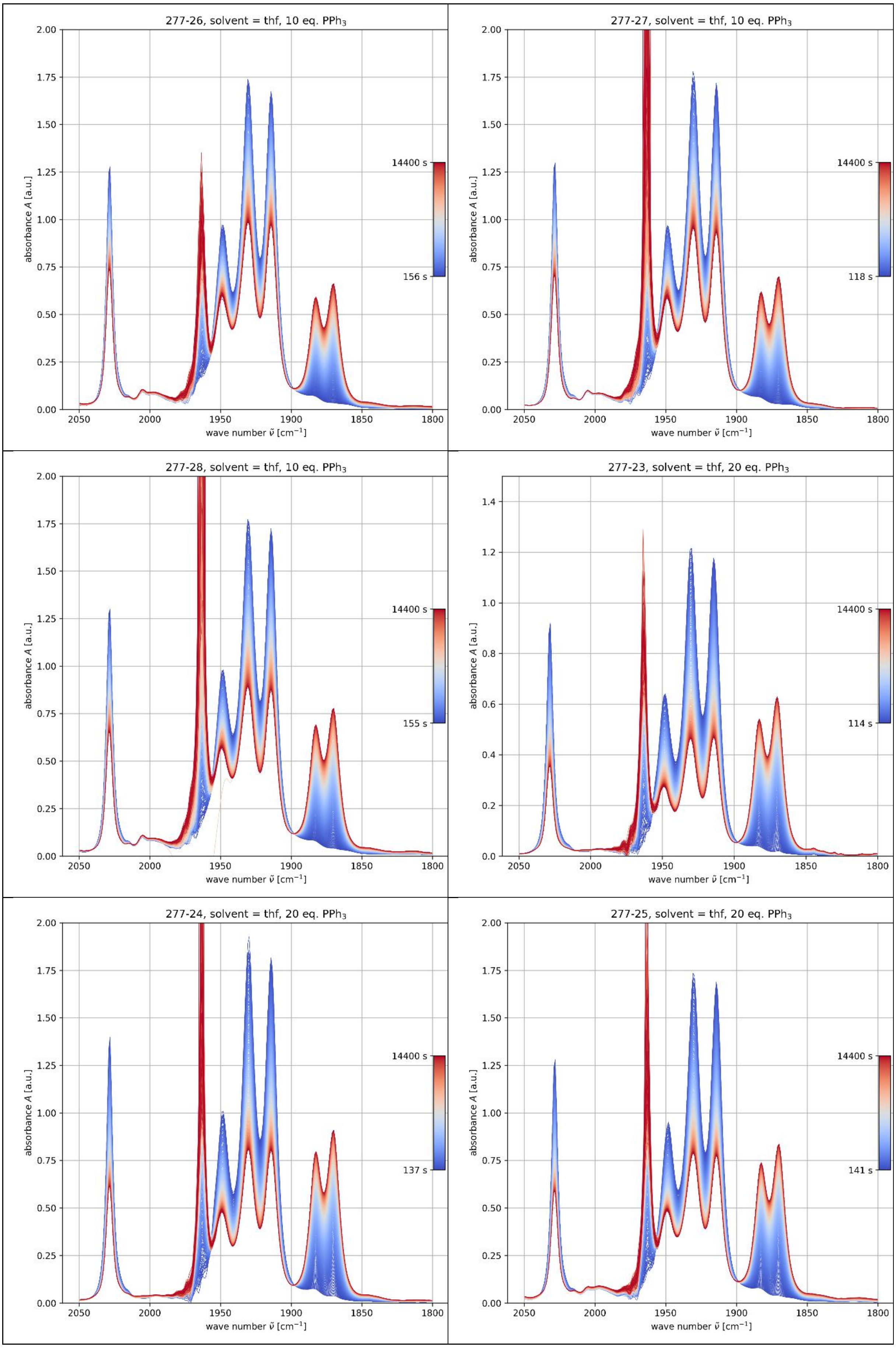

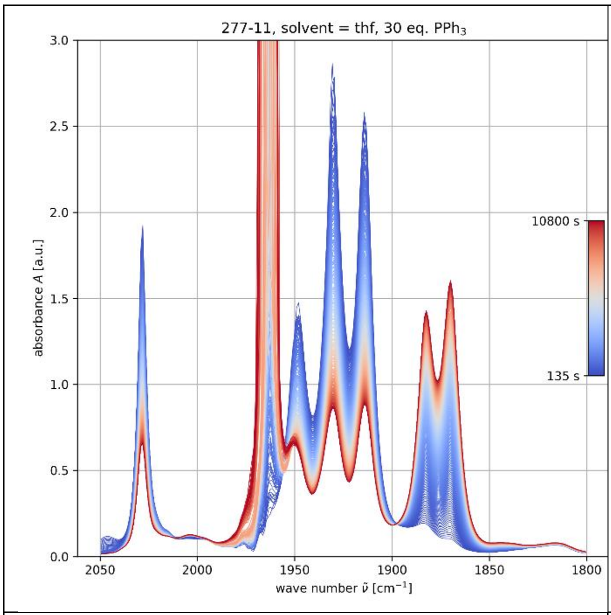
277-11, solvent = thf, 30 eq. PPh3
absorbance A [a.u.]
wave number ṽ [cm−1]
10800 s
135 s

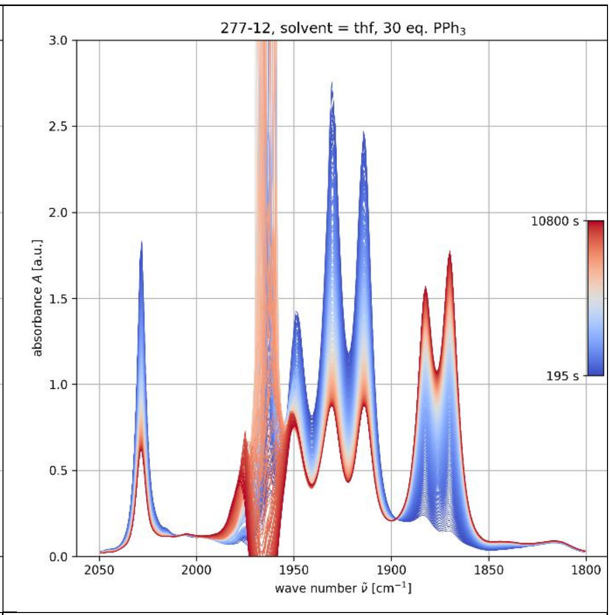
277-12, solvent = thf, 30 eq. PPh3
absorbance A [a.u.]
wave number ṽ [cm−1]
10800 s
195 s

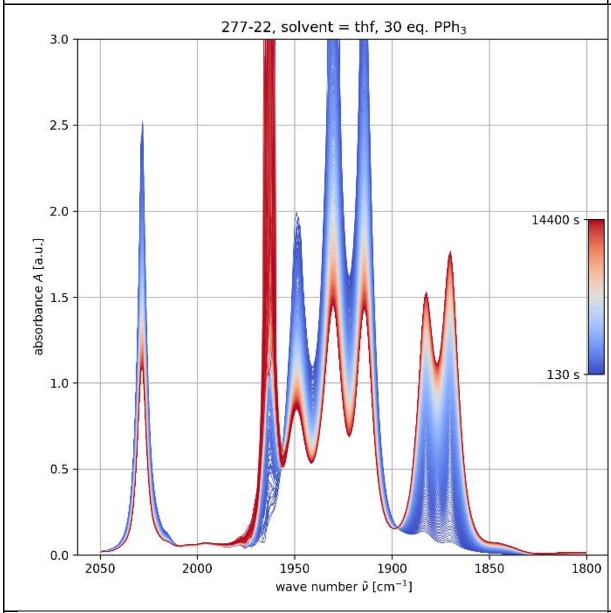
277-22, solvent = thf, 30 eq. PPh3
absorbance A [a.u.]
wave number ṽ [cm−1]
14400 s
130 s

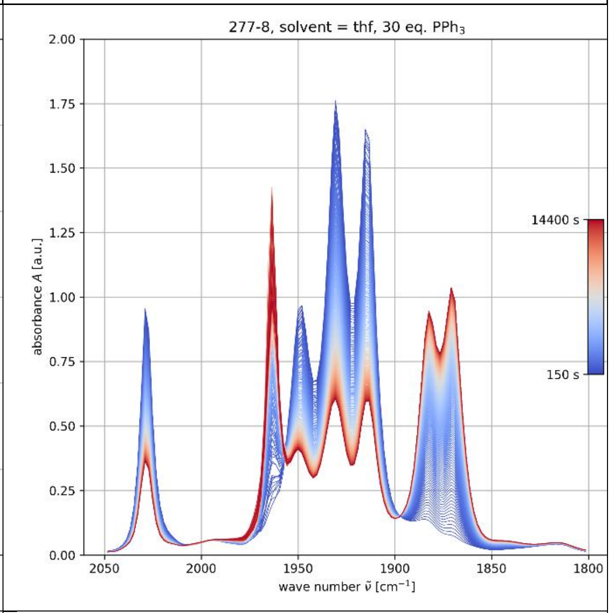
277-8, solvent = thf, 30 eq. PPh3
absorbance A [a.u.]
wave number ṽ [cm−1]
14400 s
150 s

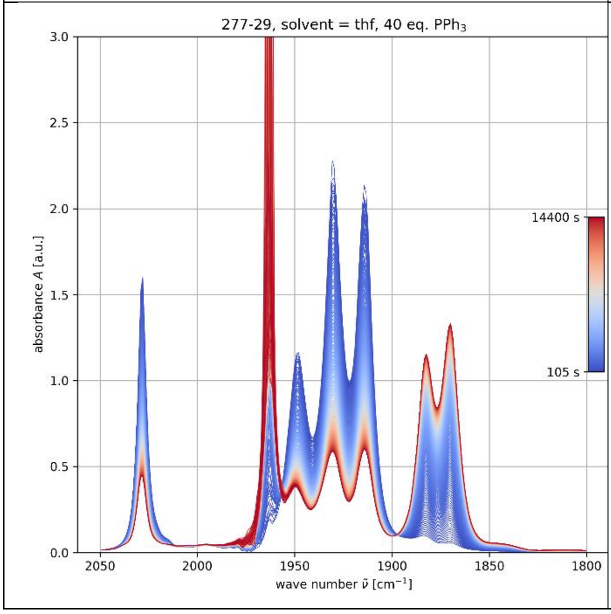
277-29, solvent = thf, 40 eq. PPh3
absorbance A [a.u.]
wave number ṽ [cm−1]
14400 s
105 s

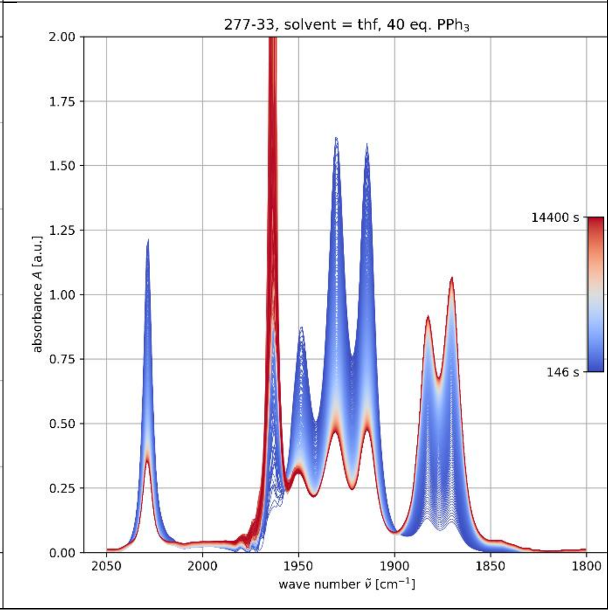
277-33, solvent = thf, 40 eq. PPh3
absorbance A [a.u.]
wave number ṽ [cm−1]
14400 s
146 s

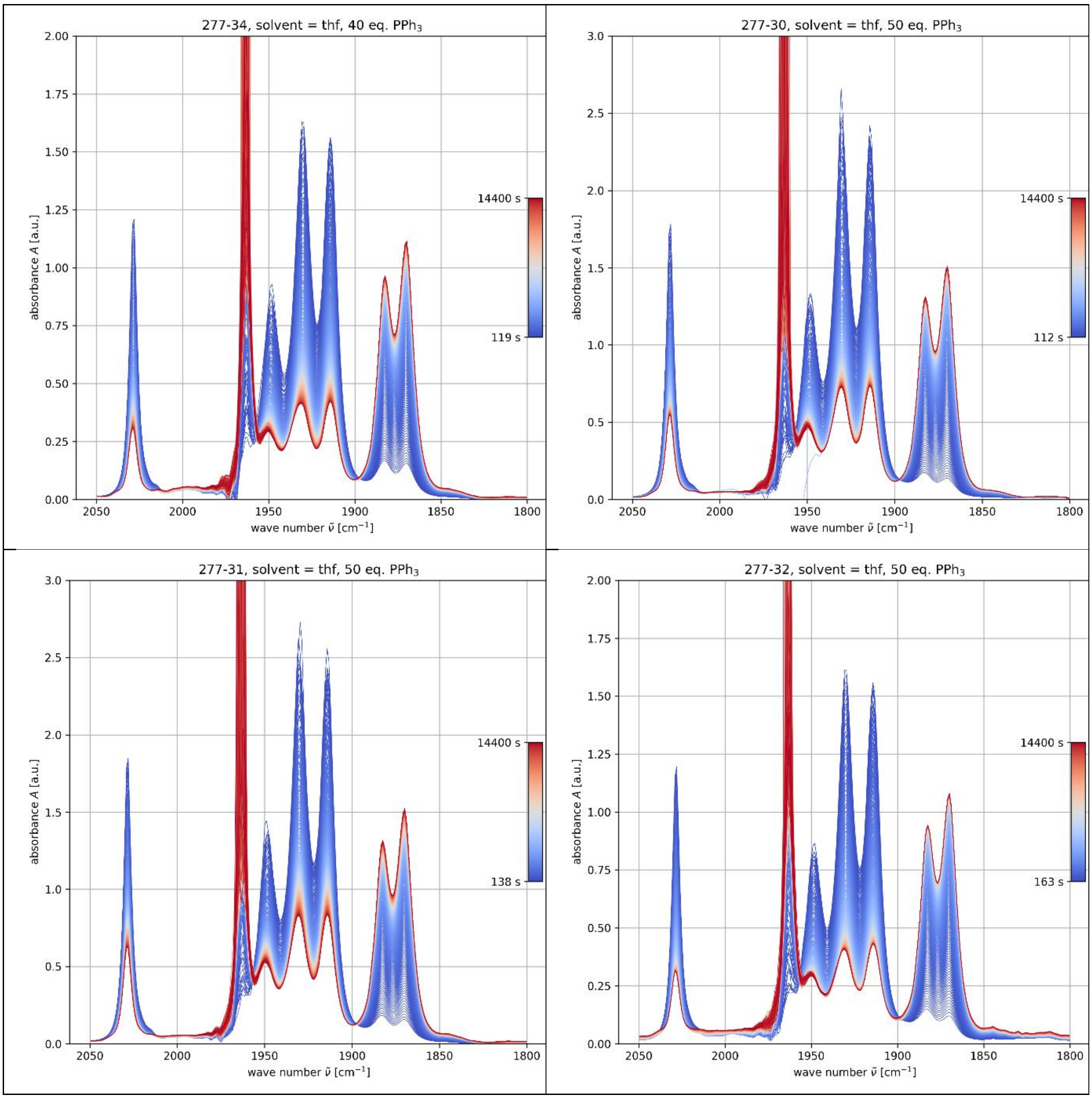

Table S16: $\ln(|A_\infty - A_t|)$ vs. time plots and linear fits including fit parameters for the different kinetic runs.

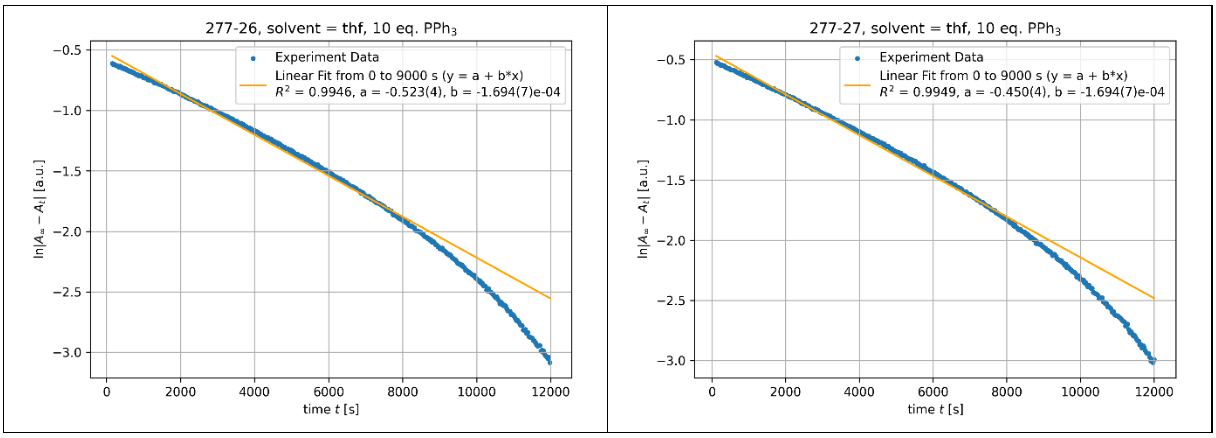

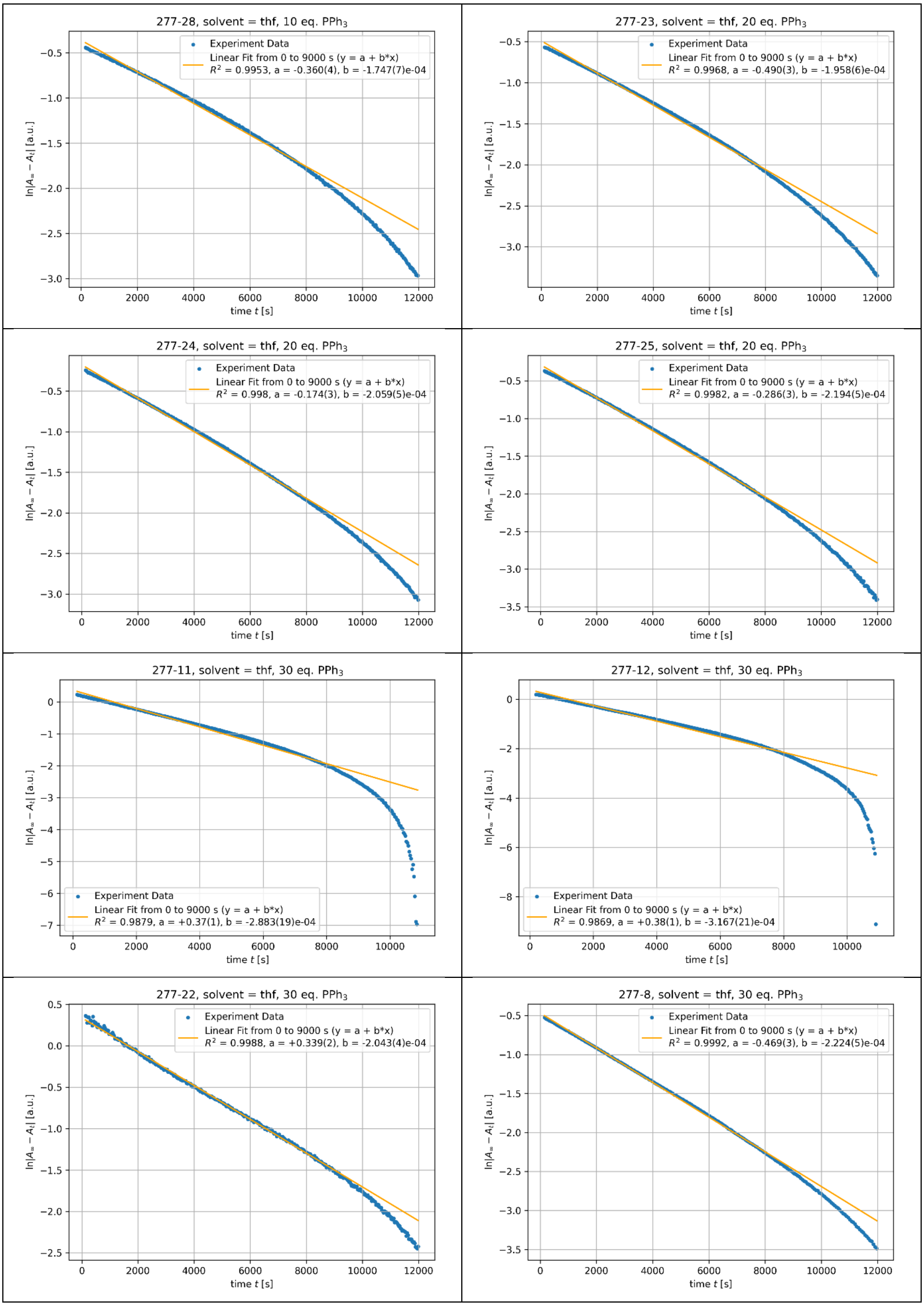
277-28, solvent = thf, 10 eq. PPh3
Experiment Data
Linear Fit from 0 to 9000 s (y = a + b*x)
R2 = 0.9953, a = -0.360(4), b = -1.747(7)e-04
277-23, solvent = thf, 20 eq. PPh3
Experiment Data
Linear Fit from 0 to 9000 s (y = a + b*x)
R2 = 0.9968, a = -0.490(3), b = -1.958(6)e-04
277-24, solvent = thf, 20 eq. PPh3
Experiment Data
Linear Fit from 0 to 9000 s (y = a + b*x)
R2 = 0.998, a = -0.174(3), b = -2.059(5)e-04
277-25, solvent = thf, 20 eq. PPh3
Experiment Data
Linear Fit from 0 to 9000 s (y = a + b*x)
R2 = 0.9982, a = -0.286(3), b = -2.194(5)e-04
277-11, solvent = thf, 30 eq. PPh3
Experiment Data
Linear Fit from 0 to 9000 s (y = a + b*x)
R2 = 0.9879, a = +0.37(1), b = -2.883(19)e-04
277-12, solvent = thf, 30 eq. PPh3
Experiment Data
Linear Fit from 0 to 9000 s (y = a + b*x)
R2 = 0.9869, a = +0.38(1), b = -3.167(21)e-04
277-22, solvent = thf, 30 eq. PPh3
Experiment Data
Linear Fit from 0 to 9000 s (y = a + b*x)
R2 = 0.9988, a = +0.339(2), b = -2.043(4)e-04
277-8, solvent = thf, 30 eq. PPh3
Experiment Data
Linear Fit from 0 to 9000 s (y = a + b*x)
R2 = 0.9992, a = -0.469(3), b = -2.224(5)e-04
ln|A∞ − At| [a.u.]
time t [s]

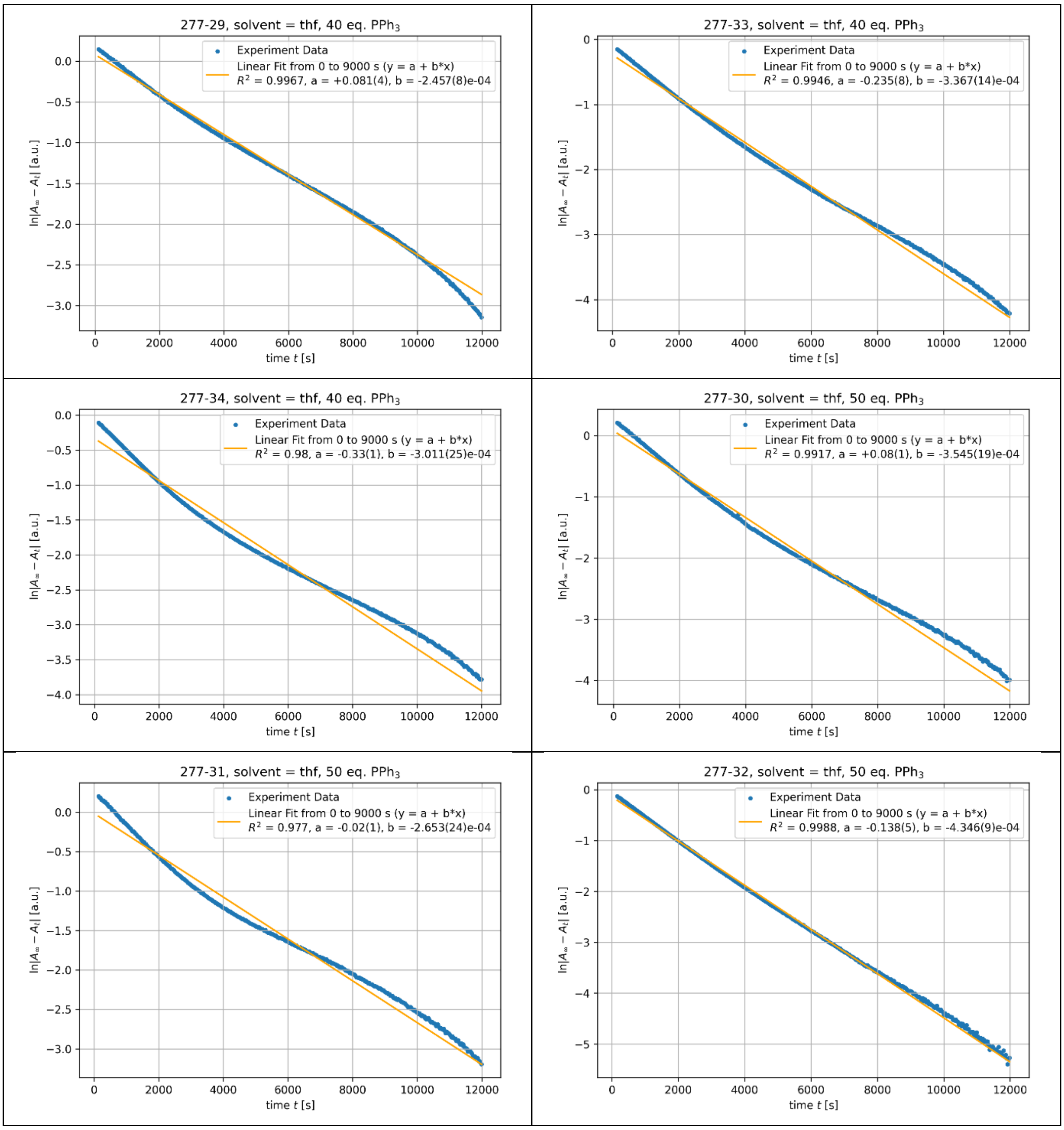

Table S17: Mean apparent rate constants k' for different PPh_3 concentrations.

**Equivalents PPh_3**	**$c(PPh_3)$ [mol L^{-1}]**	**Mean k' [s^{-1}]**	**$\sigma(k')$ [s^{-1}]**
**10**	0.385	$1.71 \cdot 10^{-4}$	$0.03 \cdot 10^{-4}$
**20**	0.769	$2.1 \cdot 10^{-4}$	$0.1 \cdot 10^{-4}$
**30**	1.154	$2.6 \cdot 10^{-4}$	$0.5 \cdot 10^{-4}$
**40**	1.538	$3.0 \cdot 10^{-4}$	$0.4 \cdot 10^{-4}$
**50**	1.923	$3.5 \cdot 10^{-4}$	$0.7 \cdot 10^{-4}$

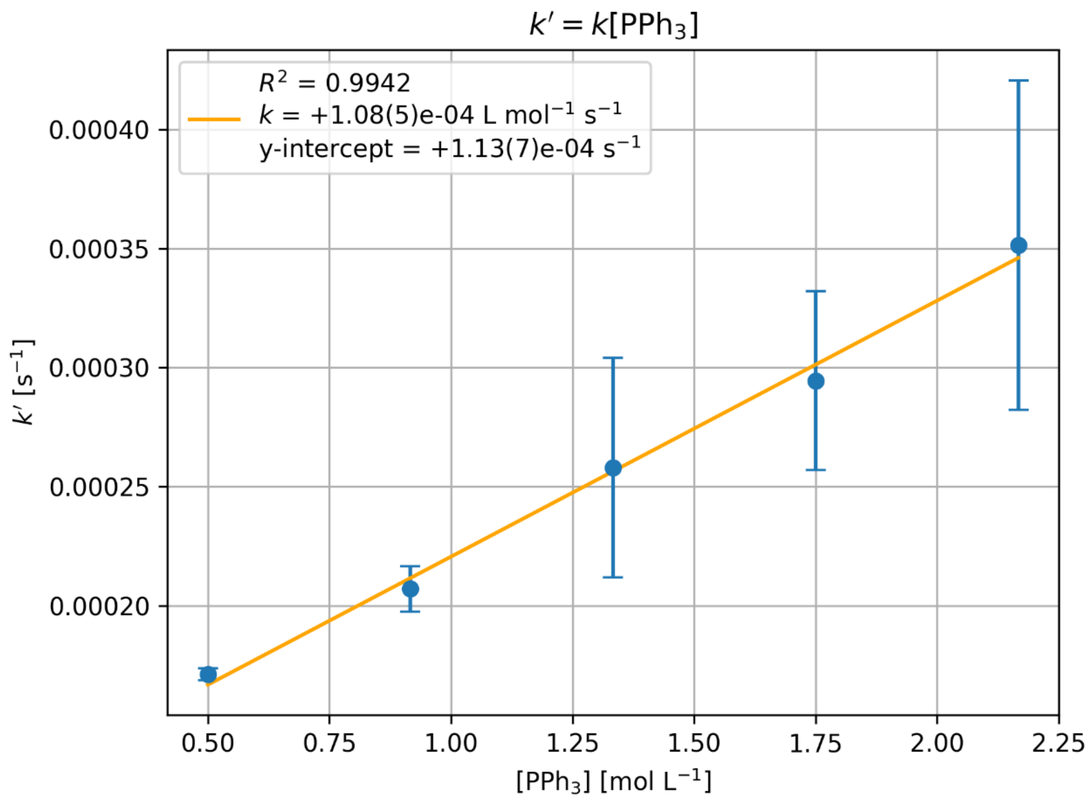

Figure S127: Plot of k' vs. concentration of PPh_3 and linear regression including fit parameters.

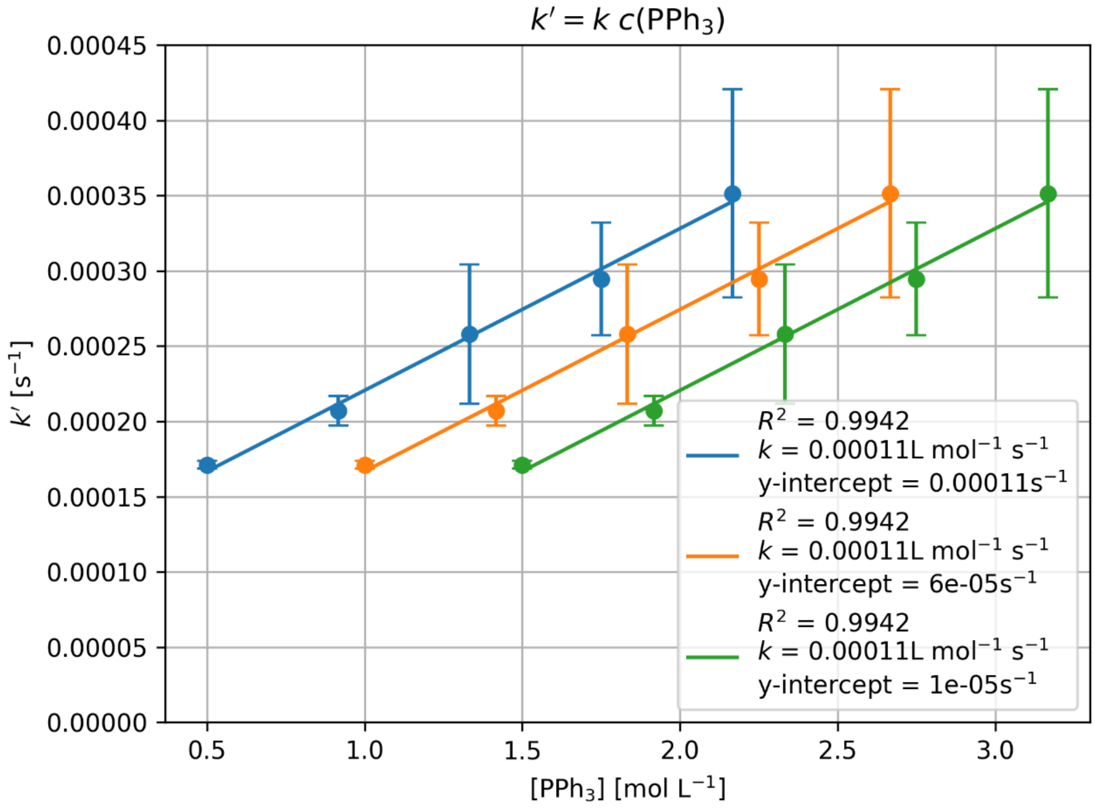

Figure S128: Plot of k' vs. concentration of PPh_3 and linear regression including fit parameters for [PPh_3] (blue), [PPh_3]+0.5 mol/L (orange) and [PPh_3]+1.0 mol/L (green) to evaluate the effect of the error of concentration determination.

The free activation energy $\Delta G^{\#}$ was estimated using the Eyering equation (Equation 9)[20] (k_b = 1.38 · 10^{-23} J K^{-1}, R = 8.1345 J K^{-1} mol^{-1}, h = 6.62 · 10^{-23} J s, T = 293.15 K) and determined to be 94.0 kJ mol^{-1} (22.5 kcal mol^{-1}). With this, the rate constant for the reaction at 50 °C k_{50} = 4.28 · 10^{-3} s^{-1} can be approximated (T = 323.15 K). Similarly, $\Delta G^{\#}$ was estimated for the substitution reaction at [(NO)Mn(CO)$_4$] reported by Basolo[21] (k = 2.2 · 10^{-3} s^{-1}, k_b = 1.38 · 10^{-23} J K^{-1}, R = 8.1345 J K^{-1} mol^{-1}, h = 6.62 · 10^{-23} J s, T = 323.15 K) and determined to be 95.8 kJ mol^{-1} (22.9 kcal mol^{-1}), as well as used for the estimation of $k_{T = 293.15\,K}$ = 2.72 · 10^{-6} s^{-1}. Calculated and experimentally determined kinetic parameters are summarized Table S18.

$$k = \frac{\mathrm{k_B}T}{\mathrm{h}}\,\mathrm{e}^{-\Delta G^{\#}/\mathrm{R}T} \Leftrightarrow \Delta G^{\#} = -\ln\left(\frac{k\mathrm{h}}{\mathrm{k_B}T}\right)\mathrm{R}T \qquad (10)$$

Table S18: Comparison of calculated and experimentally determined kinetic parameters.

Compound	$k_{T = 293.15\,K}$ [mol L^{-1} s^{-1}]	$k_{T = 318.15\,K}$ [s^{-1}]	$k_{T = 323.15\,K}$ [mol L^{-1} s^{-1}]	$\Delta G^{\#}$ [kJ mol^{-1}]	$\Delta G^{\#}$ [kcal mol^{-1}]
(IDipp)PMn(CO)$_4$	1.08(5) · 10^{-4} a)	–	4.28 · 10^{-3}	94.0	22.5
(NO)Mn(CO)$_4$	2.72 · 10^{-6}	–	2.2 · 10^{-3} b)	95.8	22.9
(η^3-allyl)Mn(CO)$_4$	–	2.8 · 10^{-4} c)	–	99.7	23.8

a) determined experimentally b) determined experimentally by Basolo[21] c) determined experimentally by Basolo[22]

S10. Computational Details

S10.1. General Information and Energies of Optimized Structures

All calculations were performed using the density functional method B97-D,[23] as implemented in the program Gaussian16.[24] For all main group elements (C, H, N, O and P) the People type all-electron triple-ζ basis set was used, extended by a set of polarisation functions 6-311G(d,p), along with a fully relativistic effective core potential – Stuttgart RSC 1997 ECP – for the 3d transition metal Manganese (ECP10MDF),[25,26] which was obtained from the "Basis Set Exchange" website [https://www.basissetexchange.org/].[27] Solvent effects were included by a continuum using a universal solvent model (SMD) for THF.[28] Harmonic vibrational frequencies are calculated to characterise the respective minima (without imaginary frequency) and transition state structures (with imaginary frequency). In addition, an Intrinsic Reaction Coordinate (IRC) calculation has been carried out on each true transition state structure using the same level of theory to determine the reaction intermediates. Energy profiles are depicted in Figures S129 (PMe_3), S130 (PPh_3) and S131 (PPh_3, THF).

Table S19: Energies for all optimized structure in the gas phase.

Compound		E_{0K}[a] / [Ha]	E_{298K}[b] / [Ha]	H_{298K}[b] / [Ha]	G_{298K}[b] / [Ha]
(IDipp)PMn(CO)$_4$	(**1**)	-2058.174403	-2058.131570	-2058.130626	-2058.250994
PPh$_3$		-1035.676263	-1035.659958	-1035.659014	-1035.723145
PMe$_3$		-460.949808	-460.942914	-460.941970	-460.978278
CO		-113.270155	-113.267795	-113.266851	-113.289291
(IDipp)PMn(IMe)(CO)$_3$	(**4**)	-2328.010129	-2327.958359	-2327.957415	-2328.096199
(IDipp)PMn(XyNC)(CO)$_3$	(**5**)	-2347.656331	-2347.604800	-2347.603855	-2347.745847
**PPh$_3$ substitution**	IN1	-3093.873336	-3093.812992	-3093.812048	-3093.970631
	TS1	-3093.858691	-3093.797898	-3093.796954	-3093.958461
	IN2	-3093.873338	-3093.812993	-3093.812049	-3093.970644
	TS2	-3093.838660	-3093.777550	-3093.776606	-3093.938825
	IN3	-3093.864904	-3093.802573	-3093.801629	-3093.969626
(IDipp)PMn(PPh$_3$)(CO)$_3$	(**2**)	-2980.590562	-2980.532216	-2980.531272	-2980.687365
**PMe$_3$ substitution**	IN1	-2519.135089	-2519.083199	-2519.082254	-2519.225037
	TS1	-2519.137261	-2519.086560	-2519.085616	-2519.222734
	IN2	-2519.146845	-2519.095979	-2519.095035	-2519.231862
	TS2	-2519.110662	-2519.059130	-2519.058186	-2519.197545
	IN3	-2519.110924	-2519.058519	-2519.057575	-2519.199916
(IDipp)PMn(PMe$_3$)(CO)$_3$	(**3**)	-2405.856513	-2405.807864	-2405.806920	-2405.940594

[a] DFT energy incl. ZPE.
[b] standard conditions T = 298.15 K and p = 1 atm.

Table S20: Energies for all optimized structures in THF solution (SMD model).

Compound		E_{0K}[a] / [Ha]	E_{298K}[b] / [Ha]	H_{298K}[b] / [Ha]	G_{298K}[b] / [Ha]
(IDipp)PMn(CO)$_4$	(**1**)	-2058.203164	-2058.160576	-2058.159632	-2058.279165
PPh$_3$		-1035.694230	-1035.677876	-1035.676932	-1035.741627
CO		-113.270155	-113.267794	-113.266850	-113.289291
**PPh$_3$ substitution in THF**	IN1	-3093.916244	-3093.856043	-3093.855098	-3094.013858
	TS1	-3093.907380	-3093.847022	-3093.846078	-3094.007345
	IN2	-3093.916125	-3093.856019	-3093.855074	-3094.012798
	TS2	-3093.881716	-3093.820890	-3093.819946	-3093.980919
	IN3	-3093.904607	-3093.842904	-3093.841960	-3094.005245
(IDipp)PMn(PPh$_3$)(CO)$_3$	(**2**)	-2980.634980	-2980.576821	-2980.575877	-2980.731018

[a] DFT energy incl. ZPE.
[b] standard conditions T = 298.15 K and p = 1 atm.

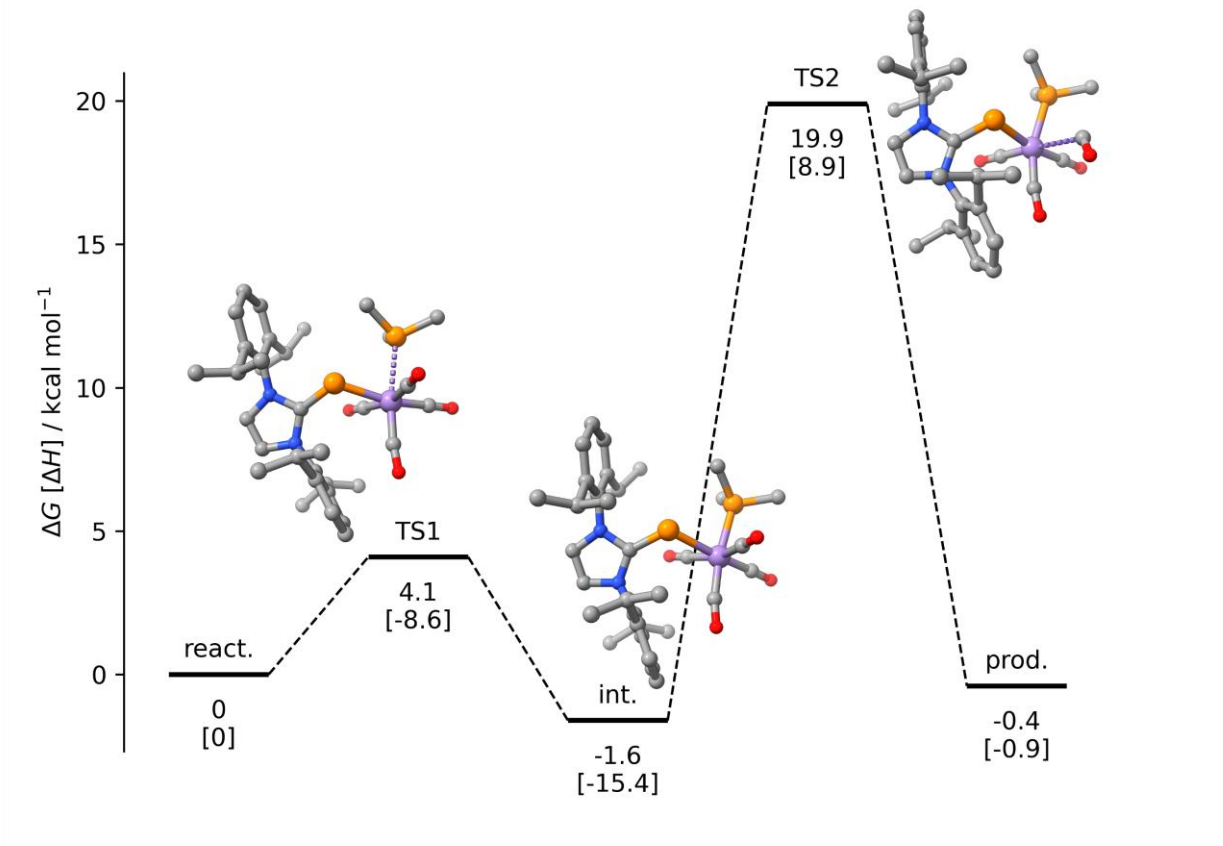

Figure S132: Calculated energy profile for the carbonyl substitution reaction of (IDipp)PMn(CO)$_4$ with PMe$_3$ (G16: B97-D // Mn: SSD/6-311G(d,p)).

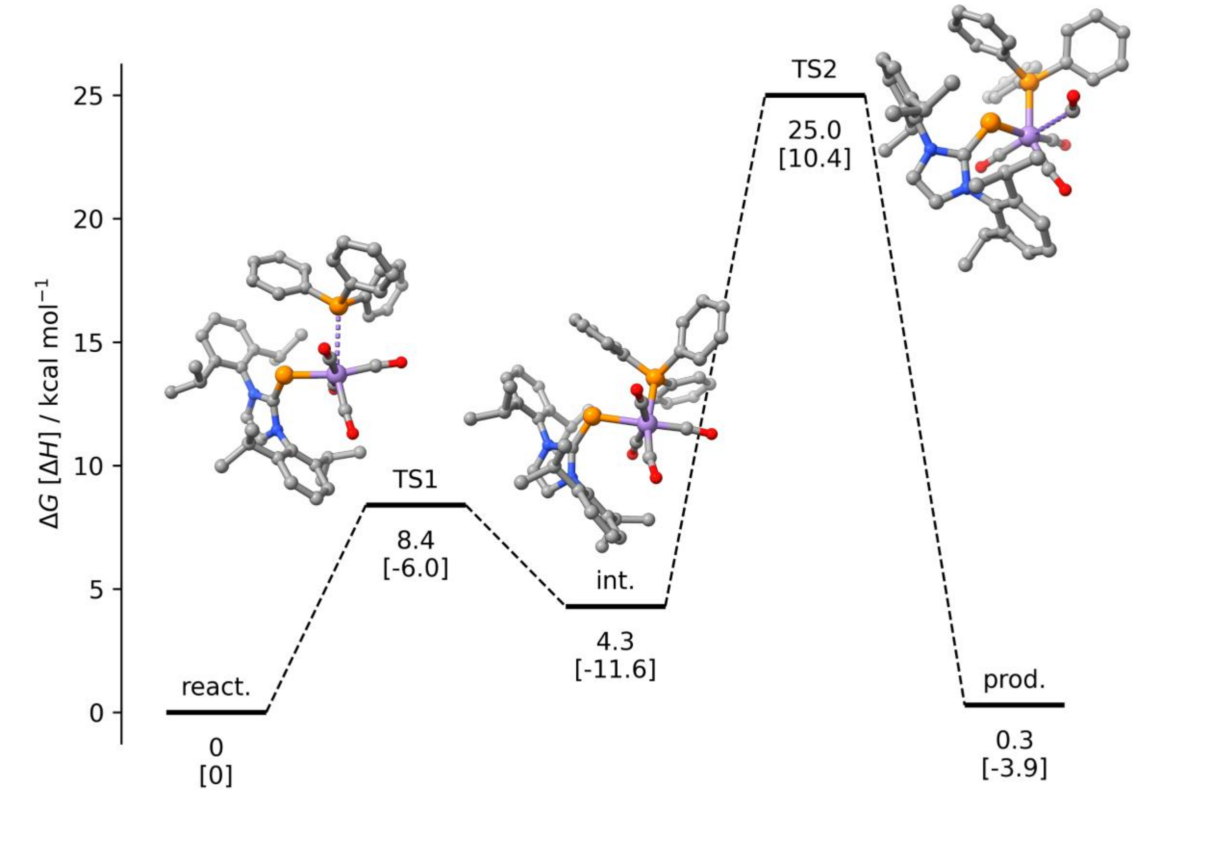

Figure S133: Calculated energy profile for the carbonyl substitution reaction of (IDipp)PMn(CO)$_4$ with PPh$_3$ (G16: B97-D // Mn: SSD/6-311G(d,p) // SMD: THF).

S10.2. NBO Analysis

Natural Bond Orbital (NBO) analysis was accomplished using NBO version 3.1[29–33] which is included in Gaussian16. AIM charges were calculated using the Multiwfn program.[34,35] A comparison of selected computational data can be found in Table S26.

Table S21: NBO analysis of (IDipp)PMn$(CO)_4$ (**1**) at the B97-D/6-311G(d,p) level of theory in vacuum.

**(IDipp)PMn$(CO)_4$**				
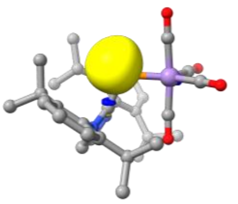	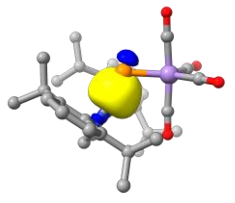	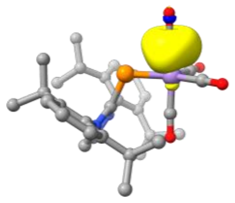	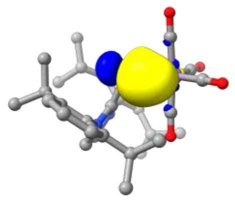	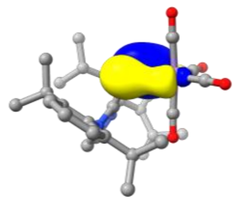
**NBO #144**	**NBO #7**	**NBO #4**	**NBO #2**	**NBO #1**

**NBO no.**	**type**	**element**	**NBO charge**	**WBI**	**coefficent**	**localization [%]**	**AO contribution [%] s**	**p**	**d**
144	LP(P)	P	0.41		1.00	100	67	33	0
7	s(P-C_{NHC})	P		1.03	0.55	30	13	86	1
		C	0.15		0.84	70	43	57	0
4	s(Mn-CO_{cis})	Mn	-2.21	1.07	0.58	33	20	52	28
		C	0.82		0.82	67	68	32	0
2	s(Mn-P)	Mn		1.36	0.62	38	18	52	30
		P			0.79	62	17	83	0
1	p(Mn-P)	Mn			0.64	41	3	43	54
		P			0.77	59	4	96	0

Table S22: NBO analysis of (IDipp)P(PPh_3)Mn$(CO)_3$ (**2**) at the B97-D/6-311G(d,p) level of theory in vacuum.

(IDipp)PMn(PPh_3)$(CO)_3$

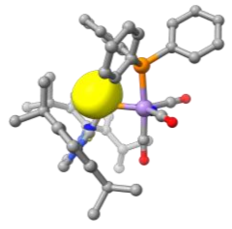	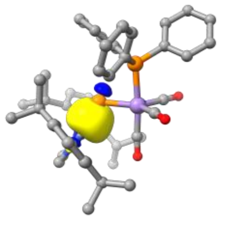	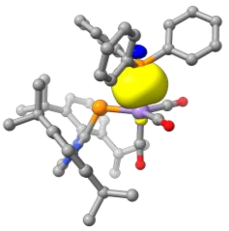	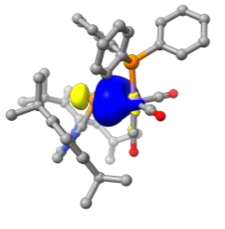	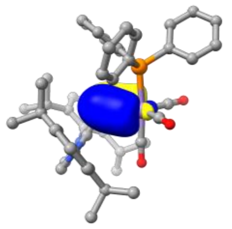
**NBO #208**	**NBO #7**	**NBO #6**	**NBO #2**	**NBO #1**

NBO no.	type	element	NBO charge	WBI	coefficent	localization [%]	AO contribution [%] s	p	d
208	LP(P)	P	0.34		1.00	100	65	35	0
7	s($P-C_{NHC}$)	P		1.07	0.55	30	13	86	1
		C	0.14		0.84	70	44	56	0
6	s(Mn-PPh_3)	Mn	-2.18	0.72	0.57	32	17	56	27
		P	1.36		0.82	68	36	64	0
2	s(Mn-P)	Mn		1.37	0.61	37	21	54	25
		P			0.79	63	22	78	0
1	p(Mn-P)	Mn			0.61	38	0	43	57
		P			0.79	62	0	99	0

Table S23: NBO analysis of (IDipp)P(PMe$_3$)Mn(CO)$_3$ (**3**) at the B97-D/6-311G(d,p) level of theory in vacuum.

**(IDipp)PMn(PMe$_3$)(CO)$_3$**				
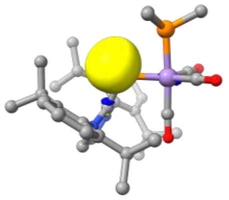	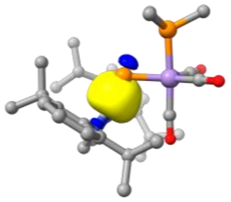	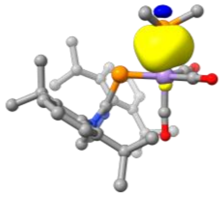	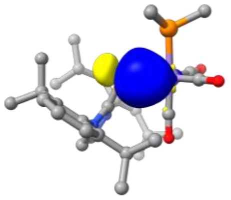	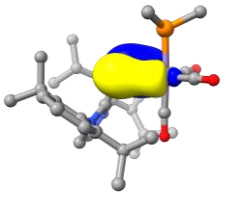
**NBO #160**	**NBO #7**	**NBO #6**	**NBO #2**	**NBO #1**

**NBO no.**	**type**	**element**	**NBO charge**	**WBI**	**coefficent**	**localization [%]**	**AO contribution [%] s**	**p**	**d**
160	LP(P)	P	0.27		1.00	100	64	36	0
7	s(P-C$_{NHC}$)	P		1.07	0.55	31	14	85	1
		C	0.15		0.83	69	44	56	0
6	s(Mn-PMe$_3$)	Mn	-2.18	0.75	0.58	34	17	55	28
		P	1.34		0.81	66	38	62	0
2	s(Mn-P)	Mn		1.38	0.60	36	19	54	27
		P			0.80	64	20	80	0
1	p(Mn-P)	Mn			0.61	38	2	43	55
		P			0.79	62	2	97	0

Table S24: NBO analysis of (IDipp)P(IMe)Mn$(CO)_3$ (**4**) at the B97-D/6-311G(d,p) level of theory in vacuum.

**(IDipp)PMn(IMe)$(CO)_3$**				
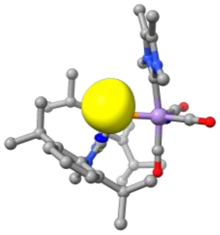	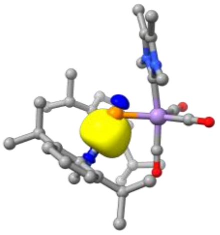	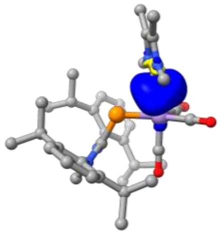	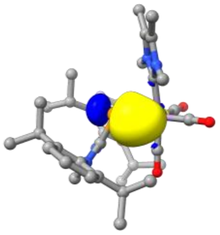	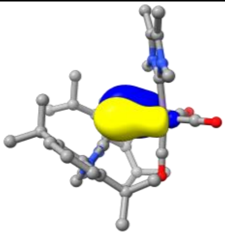
**NBO #172**	**NBO #7**	**NBO #6**	**NBO #2**	**NBO #1**

**NBO no.**	**type**	**element**	**NBO charge**	**WBI**	**coefficent**	**localization [%]**	**AO contribution [%]**		
							**s**	**p**	**d**
172	LP(P)	P	0.26		1.00	100	65	35	0
7	s(P-C_{NHC})	P		1.08	0.55	30	14	86	1
		C	0.14		0.84	70	44	56	0
6	s(Mn-IMe)	Mn	-1.99	0.71	0.54	29	17	51	31
		C	0.44		0.84	71	47	53	0
2	s(Mn-P)	Mn		1.32	0.59	35	19	52	28
		P			0.80	65	19	81	0
1	p(Mn-P)	Mn			0.59	35	2	45	52
		P			0.80	65	3	97	0

Table S25: NBO analysis of (IDipp)P(XyNC)Mn(CO)$_3$ (**5**) at the B97-D/6-311G(d,p) level of theory in vacuum.

(IDipp)PMn(XyNC)(CO)$_3$

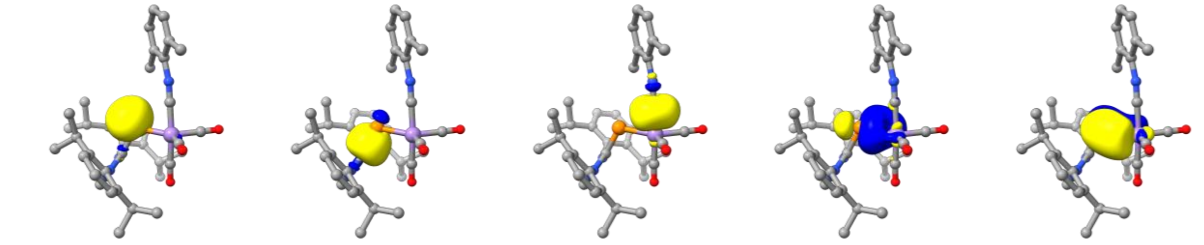

NBO #172	NBO #7	NBO #3	NBO #2	NBO #1

NBO no.	type	element	NBO charge	WBI	coefficent	localization [%]	AO contribution [%] s	p	d
174	LP(P)	P	0.34		1.00	100	66	34	0
7	s(P-C$_{NHC}$)	P		1.04	0.55	30	13	86	1
		C	0.15		0.84	70	43	57	0
3	s(Mn-CNXy)	Mn	-2.10	0.97	0.57	32	20	52	29
		C	0.60		0.82	68	65	35	0
2	s(Mn-P)	Mn		1.32	0.61	37	18	52	30
		P			0.80	63	18	82	0
1	p(Mn-P)	Mn			0.62	39	2	43	55
		P			0.78	61	3	97	0

Table S26: Comparison of selected computational data for Mn carbonyls [(IDipp)P(L)Mn(CO)$_3$)] **1**–**5.**

Compound	L	Mn–P [Å] (WBI)	Mn–L [Å] (WBI)	P–C1 [Å] (WBI)	NBO charges Mn	P	C1	L	AIM Charges Mn	P	C1	L
**1**	CO	2.220 (1.36)	1.855 (1.07)	1.830 (1.03)	−2.21	0.41	0.15	0.82	0.79	0.21	0.37	0.87
**2**	PPh$_3$	2.212 (1.37)	2.348 (0.72)	1.810 (1.07)	−2.18	0.27	0.15	1.34	0.72	0.15	0.34	1.55
**3**	PMe$_3$	2.221 (1.38)	2.326 (0.75)	1.813 (1.07)	−2.18	0.34	0.14	1.36	0.72	0.11	0.35	1.55
**4**	IMe	2.239 (1.32)	2.046 (0.71)	1.814 (1.08)	−1.99	0.26	0.14	0.44	0.81	0.11	0.35	0.70
**5**	XyNC	2.228 (1.32)	1.887 (0.97)	1.824 (1.04)	−2.10	0.34	0.15	0.60	0.81	0.18	0.37	0.70

S11. References

[1] B. E. Mann, R. A. Motterlini, D. A. Scapens, *Therapeutic Delivery of Carbon Monoxide*, **2008**, WO2008003953A2.

[2] A. Doddi, D. Bockfeld, T. Bannenberg, P. G. Jones, M. Tamm, *Angew. Chem. Int. Ed.* **2014**, *53*, 13568–13572.

[3] A. J. Arduengo, H. V. R. Dias, J. C. Calabrese, *Chem. Lett.* **1997**, *26*, 143–144.

[4] W. Wolfsberger, H. Schmidbaur, *Synth. React. Inorg. Met.-Org. Chem.* **1974**, DOI 10.1080/00945717408069645.

[5] M. L. Luetkens, A. P. Sattelberger, H. H. Murray, J. D. Basil, J. P. Fackler, R. A. Jones, D. E. Heaton, *Inorganic Syntheses, Volume 28* **2007**, 305–310.

[6] N. Kuhn, T. Kratz, *Synthesis* **1993**, *1993*, 561–562.

[7] Bruker, TopSpin 4.4.0, Bruker Corporation, Billerica, MA, USA, **2024**.

[8] C. R. Harris, K. J. Millman, S. J. van der Walt, R. Gommers, P. Virtanen, D. Cournapeau, E. Wieser, J. Taylor, S. Berg, N. J. Smith, R. Kern, M. Picus, S. Hoyer, M. H. van Kerkwijk, M. Brett, A. Haldane, J. F. del Río, M. Wiebe, P. Peterson, P. Gérard-Marchant, K. Sheppard, T. Reddy, W. Weckesser, H. Abbasi, C. Gohlke, T. E. Oliphant, *Nature* **2020**, *585*, 357–362.

[9] The pandas development team, **2020**, DOI 10.5281/zenodo.3509134.

[10] W. McKinney, in *Proc. 9th Python Sci. Conf.* (Eds.: S. van der Walt, J. Millman), **2010**, pp. 56–61.

[11] P. Virtanen, R. Gommers, T. E. Oliphant, M. Haberland, T. Reddy, D. Cournapeau, E. Burovski, P. Peterson, W. Weckesser, J. Bright, S. J. van der Walt, M. Brett, J. Wilson, K. J. Millman, N. Mayorov, A. R. J. Nelson, E. Jones, R. Kern, E. Larson, C. J. Carey, İ. Polat, Y. Feng, E. W. Moore, J. VanderPlas, D. Laxalde, J. Perktold, R. Cimrman, I. Henriksen, E. A. Quintero, C. R. Harris, A. M. Archibald, A. H. Ribeiro, F. Pedregosa, P. van Mulbregt, SciPy 1.0 Contributors, *Nat. Methods* **2020**, *17*, 261–272.

[12] E. O. Lebigot, "Uncertainties: a Python package for calculations with uncertainties," can be found under https://pythonhosted.org/uncertainties, **n.d.**

[13] J. D. Hunter, *Comput. Sci. Eng.* **2007**, *9*, 90–95.

[14] Rigaku Oxford Diffraction, CrysAlisPRO Software System, Versions 1.171.42.56a (2022), 1.171.42.64a (2022) And 1.171.43.120a (2024), Oxford Diffraction, Oxford, UK, **2024**.

[15] G. M. Sheldrick, *Acta Cryst.* **2015**, *A71*, 3–8.

[16] G. M. Sheldrick, *Acta Cryst.* **2015**, *C71*, 3–8.

[17] O. V. Dolomanov, L. J. Bourhis, R. J. Gildea, J. A. K. Howard, H. Puschmann, *J. Appl. Crystallogr.* **2009**, *42*, 339–341.

[18] J. R. Chipperfield, *J. Organomet. Chem.* **1989**, *363*, 253–263.

[19] Q. Z. Shi, T. G. Richmond, W. C. Trogler, F. Basolo, *J. Am. Chem. Soc.* **1984**, *106*, 71–76.

[20] Keith J. Laldler, M. Christine King, *J. Phys. Chem.* **1983**, *87*, 2657–2664.

[21] Henning. Wawersik, Fred. Basolo, *J. Am. Chem. Soc.* **1967**, *89*, 4626–4630.

[22] G. T. Palmer, F. Basolo, *J. Am. Chem. Soc.* **1985**, *107*, 3122–3129.

[23] S. Grimme, *J. Comput. Chem.* **2006**, *27*, 1787–1799.

[24] M. J. Frisch, G. W. Trucks, H. B. Schlegel, G. E. Scuseria, M. A. Robb, J. R. Cheeseman, G. Scalmani, V. Barone, G. A. Petersson, H. Nakatsuji, X. Li, M. Caricato, A. V. Marenich, J. Bloino, B. G. Janesko, R. Gomperts, B. Mennucci, H. P. Hratchian, J. V. Ortiz, A. F. Izmaylov, J. L. Sonnenberg, D. Williams-Young, F. Ding, F. Lipparini, F. Egidi, J. Goings, B. Peng, A. Petrone, T. Henderson, D. Ranasinghe, V. G. Zakrzewski, J. Gao, N. Rega, G. Zheng, W. Liang, M. Hada, M. Ehara, K. Toyota, R. Fukuda, J. Hasegawa, M. Ishida, T. Nakajima, Y. Honda, O. Kitao, H. Nakai, T. Vreven, K. Throssell, J. A. Montgomery Jr., J. E. Peralta, F. Ogliaro, M. J. Bearpark, J. J. Heyd, E. N. Brothers, K. N. Kudin, V. N.

Staroverov, T. A. Keith, R. Kobayashi, J. Normand, K. Raghavachari, A. P. Rendell, J. C. Burant, S. S. Iyengar, J. Tomasi, M. Cossi, J. M. Millam, M. Klene, C. Adamo, R. Cammi, J. W. Ochterski, R. L. Martin, K. Morokuma, O. Farkas, J. B. Foresman, D. J. Fox, **2016**.
[25] M. Dolg, U. Wedig, H. Stoll, H. Preuss, *J. Chem. Phys.* **1987**, *86*, 866–872.
[26] J. M. L. Martin, A. Sundermann, *J. Chem. Phys.* **2001**, *114*, 3408–3420.
[27] B. P. Pritchard, D. Altarawy, B. Didier, T. D. Gibson, T. L. Windus, *J. Chem. Inf. Model.* **2019**, *59*, 4814–4820.
[28] A. V. Marenich, C. J. Cramer, D. G. Truhlar, *J. Phys. Chem. B* **2009**, *113*, 6378–6396.
[29] E. D. Glendening, A. E. Reed, J. E. Carpenter, F. Weinhold, *NBO Version 3.1*, **1980**.
[30] J. P. Foster, F. Weinhold, *J. Am. Chem. Soc.* **1980**, *102*, 7211–7218.
[31] A. E. Reed, F. Weinhold, *J. Chem. Phys.* **1983**, *78*, 4066–4073.
[32] A. E. Reed, R. B. Weinstock, F. Weinhold, *J. Chem. Phys.* **1985**, *83*, 735–746.
[33] A. E. Reed, F. Weinhold, *J. Chem. Phys.* **1985**, *83*, 1736–1740.
[34] T. Lu, F. Chen, *J. Comput. Chem.* **2012**, *33*, 580–592.
[35] T. Lu, *J. Chem. Phys.* **2024**, *161*, 082503.
